# Investigating the causal impact of gut microbiota on glioblastoma: a bidirectional Mendelian randomization study

**DOI:** 10.1186/s12864-023-09885-2

**Published:** 2023-12-18

**Authors:** Chuan Zeng, Chaolong Zhang, Chunming He, Haimin Song

**Affiliations:** 1 Department of Neurosurgery, First Affiliated Hospital of Gannan Medical University, Qingnian Road, Ganzhou City, 341000 Jiangxi Province China; 2 The First Clinical Medical College of Gannan Medical University, Ganzhou City, 341000 Jiangxi Province China

**Keywords:** Glioblastoma, Gut microbiota, Mendelian randomization, Causality, Gut-brain axis

## Abstract

**Background:**

Currently, the influence of microbiota on the occurrence, progression, and treatment of cancer is a topic of considerable research interest. Therefore, based on the theory of the gut-brain axis proved by previous studies, our objective was to uncover the causal relationship between glioblastoma and the gut microbiome using Mendelian randomization analysis.

**Methods:**

We conducted a bidirectional Mendelian randomization study using summary statistics of gut microbiota derived from the MiBioGen consortium, the largest database of gut microbiota. Summary statistics for glioblastoma were obtained from IEU OpenGWAS project, which included 91 cases and 218,701 controls. We assessed the presence of heterogeneity and horizontal pleiotropy in the analyzed data. We primarily employed the inverse variance weighting method to investigate the causal relationship between gut microbiota and glioblastoma after excluding cases of horizontal pleiotropy. Four other analysis methods were employed as supplementary. Excluding abnormal results based on leave-one-out sensitivity analysis. Finally, reverse Mendelian randomization analysis was performed.

**Results:**

Four genus-level taxa and one family-level taxa exhibited causal associations with glioblastoma. And these results of reverse Mendelian randomization analysis shown glioblastoma exhibited causal associations with three genus-level taxa and one family-level taxa. However, the Prevotella7(Forward, *P*=0.006, OR=0.34, 95%CI:0.158-0.732; Reverse, *P*=0.004, OR=0.972, 95%CI:0.953-0.991) shown the causal associations with glioblastoma in the bidirectional Mendelian randomization.

**Conclusions:**

In this bidirectional Mendelian randomization study, we identified five gut microbiota species with causal associations to glioblastoma. However, additional randomized controlled trials are required to clarify the impact of gut microbiota on glioblastoma and to reveal its precise mechanisms.

**Supplementary Information:**

The online version contains supplementary material available at 10.1186/s12864-023-09885-2.

## Background

Glioblastoma (GBM) stands out as one of the most malignant primary brain tumors, characterized by its exceptionally high fatality rate. The rapid growth and heterogeneity of this tumor are significant contributors to its aggressive progression, manifesting in symptoms such as neurological impairment and cognitive decline. The current standard treatment for newly diagnosed cases entails a combination of post-surgical radiotherapy and temozolomide, followed by adjuvant temozolomide therapy [1]. Nevertheless, the tumor's aggressiveness and its deep-seated location within brain tissue pose formidable challenges to achieving complete removal. Furthermore, post-successful surgery, the presence of residual tumor cells can lead to recurrence [2]. Moreover, glioblastoma frequently displays resistance to conventional radiotherapy and chemotherapy. The presence of the blood-brain barrier further hinders the delivery of therapeutic agents to tumor tissue, presenting a formidable therapeutic obstacle [3]. Furthermore, the genetic heterogeneity of tumor cells at different sites can result in diverse phenotypes and gene expression patterns, creating an additional therapeutic challenge. At present, the treatment of glioblastoma remains a pressing concern. Consequently, researchers are exploring innovative therapeutic approaches, including immunotherapy, gene therapy, and targeted therapy. Immunotherapy involves the stimulation of the patient's immune system to selectively target and attack tumor cells [4–6]. Therefore, given the unique nature of glioblastoma, the significance of prevention and early diagnosis becomes even more pronounced. Nevertheless, despite substantial progress in clinical and basic research over the years, the precise etiology of GBM remains elusive.

The brain was historically considered an “immune-privileged” organ due to the blood-brain barrier. However, the discovery of a functional lymphatic system and the presence of peripheral immune cells have substantiated the existence of an immune system in the brain [7]. Glioblastoma , characterized as cold tumors, inhibits the immune response to cancer, leading to immunotherapy failures [8, 9]. Recent studies have highlighted the multifaceted roles of the gut microbiota, encompassing regulation of nutrient absorption, synthesis of vitamins, metabolism of bile and hormones, and fermentation of carbohydrates [10, 11]. Moreover, the gut microbiota exerts systemic effects on immunity, inflammation, and metabolism [12–14]. Emerging evidence suggests that the gut microbiota can indirectly influence brain tumor metabolism and the brain's immune environment through the production of metabolites [15, 16]. This interaction can either promote or inhibit the malignant progression of GBM. As a result, researchers are increasingly focusing on the well-established gut-brain axis, a bidirectional link between the brain and the gut [17, 18].

However, owing to the absence of evidence from randomized controlled trials, the existence of a definitive causal link between gut flora and glioblastoma remains uncertain. While randomized controlled trials serve as the gold standard for establishing causality in epidemiological investigations, conducting them can be challenging due to ethical constraints and substantial costs. To explore the potential association between the gut microbiota and GBM, we utilized Mendelian Randomization (MR) Analysis, a systematic method for assessing causality. MR employs genetic variation as an instrumental variable to model interventions, enhancing our ability to make more confident inferences regarding the influence of a factor on disease occurrence [19, 20]. In this study, we will employ MR methods to examine the potential causal connection between gut microbiota and GBM.

The objective of this study is to elucidate whether the gut microbiota's composition is linked to the risk of GBM and to delve deeper into potential underlying biological mechanisms. We anticipate that this study will offer novel insights and strategies for the future prevention and treatment of GBM. This endeavor will not only enhance our comprehension of GBM's etiology but may also offer substantial backing for the formulation of personalized therapeutic protocols, with the potential to enhance both patient survival and quality of life.

## Methods

### Study design

**Fig. 1:**
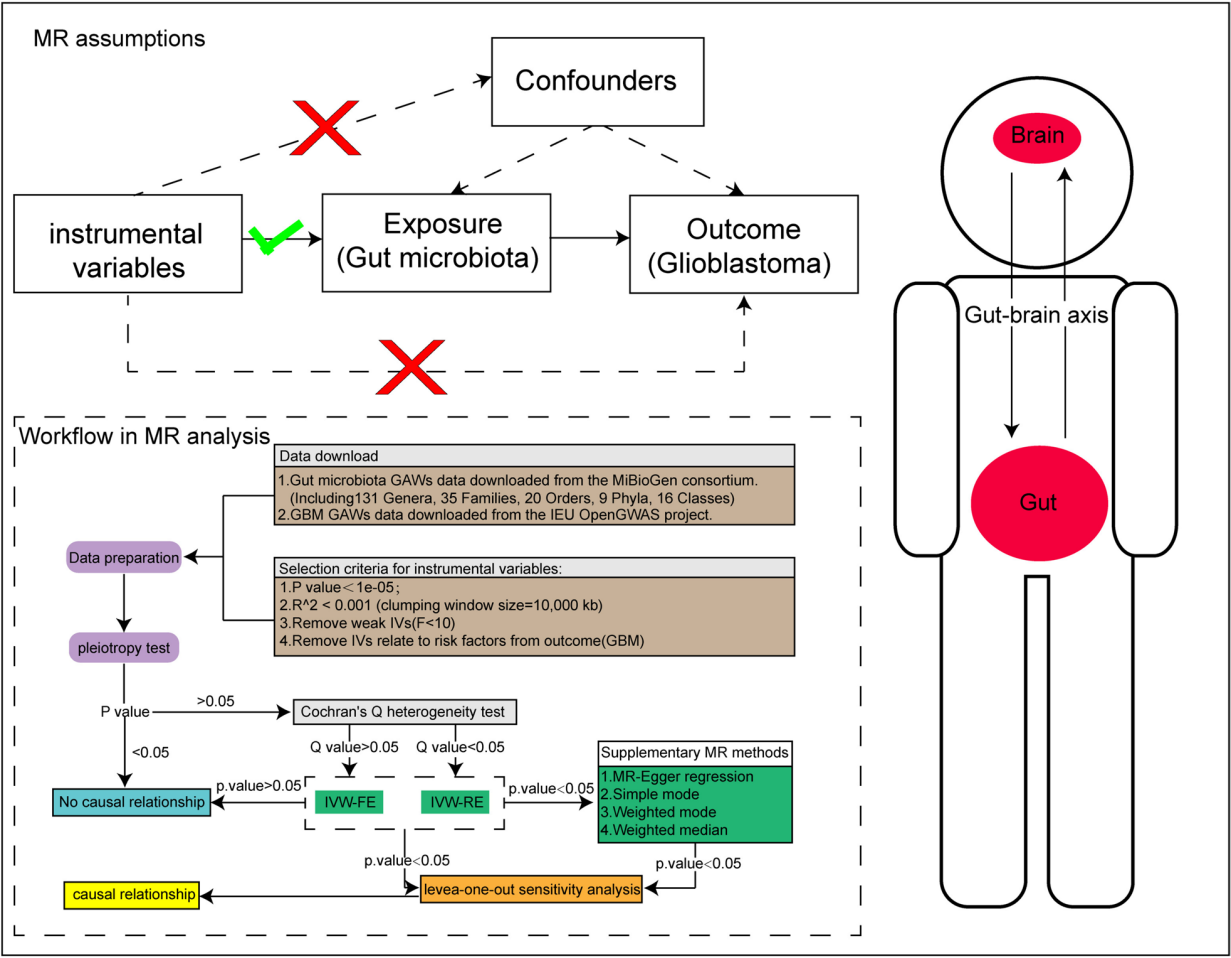
The whole study design

The entire study design is displayed in Fig. 1. MR was employed to analyze the causal relationship between the gut microbiota and GBM. We adhered to the three core principles of MR analysis: (1) Strong link between genetic variation and exposure factors [21]; (2) ensuring no correlation between genetic variation and confounders [22]; (3) affirming that genetic variation influences the outcomes solely through exposure factors, with no involvement of other pathways [23]. Concurrently, we conducted a reverse MR analysis utilizing the statistically significant findings from the initial MR analysis to obtain more robust results.

### Data source and preparation

We sourced summary statistics of gut microbiota composition from the most extensive genome-wide meta-analysis to date, conducted by the MiBioGen consortium (https://mibiogen.gcc.rug.nl.) [24]. This analysis encompassed 18,340 participants of European ethnicity from 11 countries and included 122,110 loci of genetic variation. The summary statistics for the genome-wide association study (GWAS) related to GBM were acquired from the Medical Research Council Integrative Epidemiology Unit (IEU) Open GWAS project (https://gwas.mrcieu.ac.uk/datasets/finn-b-C3_GBM/.) [25](updated to 2021.04.06, ncase=91, ncontrol=218,701, number of SNPs=16,380,466).

The selection criteria for instrumental variables (IVs) included the following steps: (1) Identification of single nucleotide polymorphisms (SNPs) associated with each genus at the locus-wide significance threshold (*P*<1.0 × 10-5) as potential IVs [18, 26]; (2) Conducting a linkage disequilibrium (LD) window analysis for all IVs (r2 < 0.001, clumping window size=10,000 kb); (3) Removal of SNPs related to exposure but lacking corresponding matches in the GWAS outcome statistics, calculated using the formula F=beta^2^_exposure_ /SE ^2^_exposure_ [23] ; (4) Exclusion of SNPs with a minor allele frequency (MAF) ≤ 0.01; and (5) In cases of palindromic SNPs, determination of forward strand alleles based on allele frequency information [27].

### Statistical analysis

MR is employed to investigate causal relationships between bacterial taxa and GBM. Before conducting the analysis, we conducted a test for horizontal pleiotropy to eliminate statistics affected by horizontal pleiotropy. This ensures that the inverse variance weighting (IVW) method can serve as the primary approach for causality assessment in MR analysis [28]. Furthermore, we employed Cochrane's Q test to evaluate heterogeneity among IVs. In cases where heterogeneity was detected (*P*<0.05), we adopted a random-effects IVW (IVW-RE) model, which offers more conservative estimates. Conversely, in the absence of heterogeneity, we utilized a fixed-effects IVW (IVW-FE) model [18]. In case the IVW results yielded statistical significance (*p*<0.05), we introduced several additional MR methods, including MR-Egger regression, simple mode, weighted median, and weighted mode. Notably, weighted median (WM) and MR-Egger regression serve to complement the IVW method and offer broader CIs [29].

Finally, we performed a leave-one-out sensitivity analysis of statistically significant causal relationships to arrive at our final results. Subsequently, to enhance result credibility, we conducted an inverse MR analysis using the GWAS summary statistics from flora causally associated with GBM as the outcome and those from GBM as the exposure, applying the same MR analysis methods as previously described. All of the aforementioned analyses were conducted using the R programming language (R version 4.3.0) and the "TwoSampleMR" package in R [30, 31].

## Results

We utilized gut microbiota GAWs data obtained from the MiBioGen consortium, which encompassed 131 genus-level taxa, 35 family-level taxa, 20 order-level taxa, 9 phylum-level taxa, 16 class-level taxa, and a total of 2,620 SNPs, as instrumental variables. Detailed information regarding these SNPs can be found in Online Resource 1: Table S1.

**Fig. 2:**
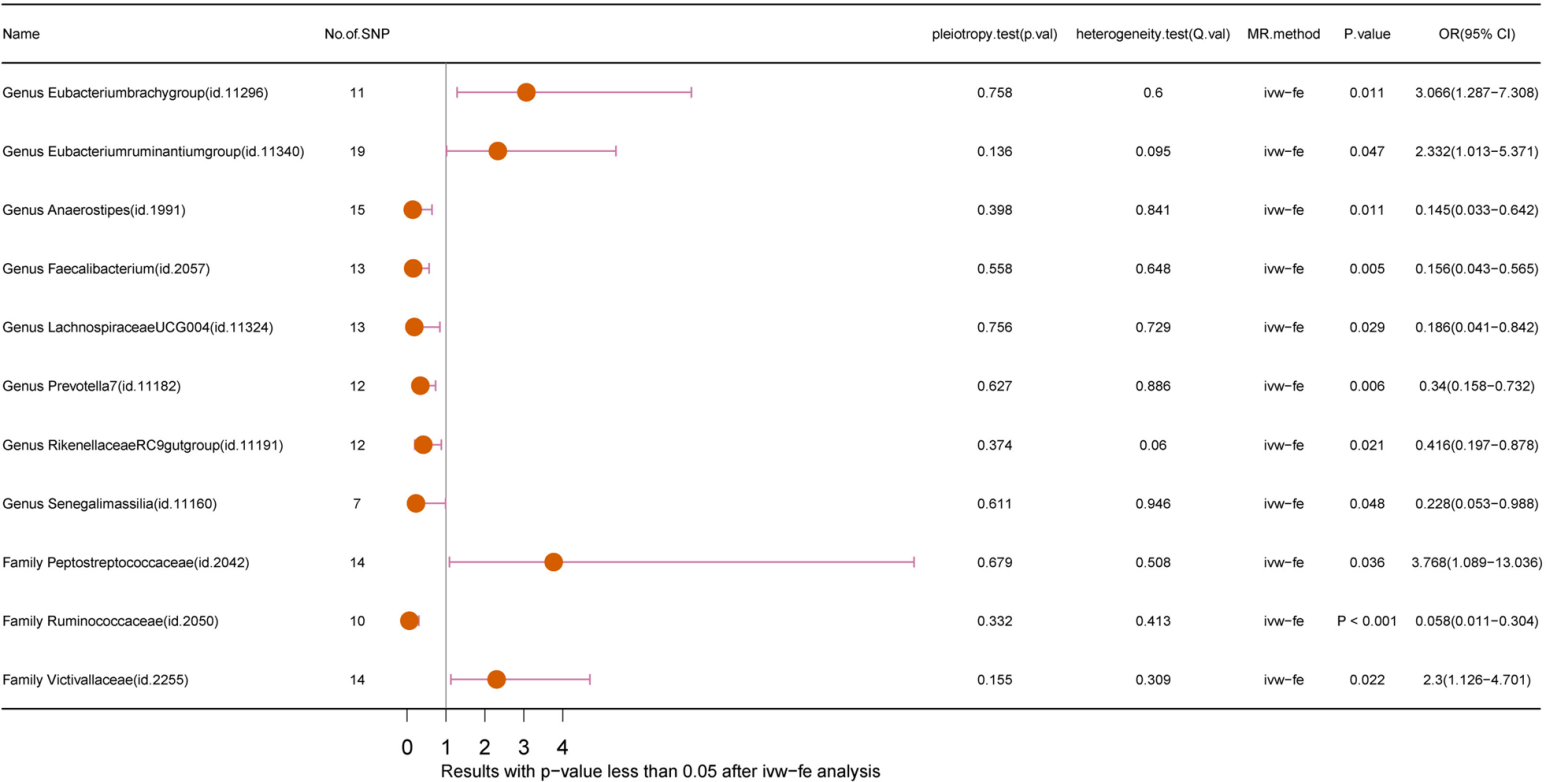
Forest plot of GM taxa associated with GBM (*P*<0.05) identified by IVW-FE method

Following the aforementioned steps, we conducted a horizontal pleiotropy test to exclude certain statistics influenced by horizontal pleiotropy. Subsequently, we employed different IVW analysis methods based on the Q-value obtained from the heterogeneity test. Notably, the Q-value for the heterogeneity test exceeded 0.05 in nearly all groups, suggesting the absence of statistical heterogeneity. As illustrated in Fig. 2**,** the IVW analysis method revealed that 8 genus-level flora (*Eubacteriumbrachygroup, Eubacteriumruminantiumgroup, Anaerostipes, Faecalibacterium, LachnospiraceaeUCG004, Prevotella7, RikenellaceaeRC9gutgroup, Senegalimassilia*) and 3 family-level flora (*Bacteroidaceae, Peptostreptococcaceae, Ruminococcaceae, Victivallaceae*) exhibited associations with GBM. Notably, *Eubacteriumbrachygroup* (Weighted median, *P*=0.007, OR=1.554, 95% CI: 1.554-15.890), *Eubacteriumruminantiumgroup* (Weighted median, *P*=0.036, OR=3.673, 95% CI: 1.087-12.411), *Prevotella7* (Weighted median, *P*=0.034, OR=0.326, 95% CI: 0.116-0.917), and *Peptostreptococcaceae* (Weighted median, *P*=0.040, OR=6.121, 95% CI: 1.089-34.402) were confirmed in two MR methods to exhibit causality with GBM (IVW and weighted median). Additionally, *Ruminococcaceae* (MR-Egger regression, *P*=0.048, OR=0.009, 95% CI: 0.000-0.468; Weighted median, *P*=0.040, OR=0.094, 95% CI: 0.010-0.897) demonstrated causality with GBM in three distinct methods (IVW, MR-Egger regression, and weighted median).

**Fig. 3:**
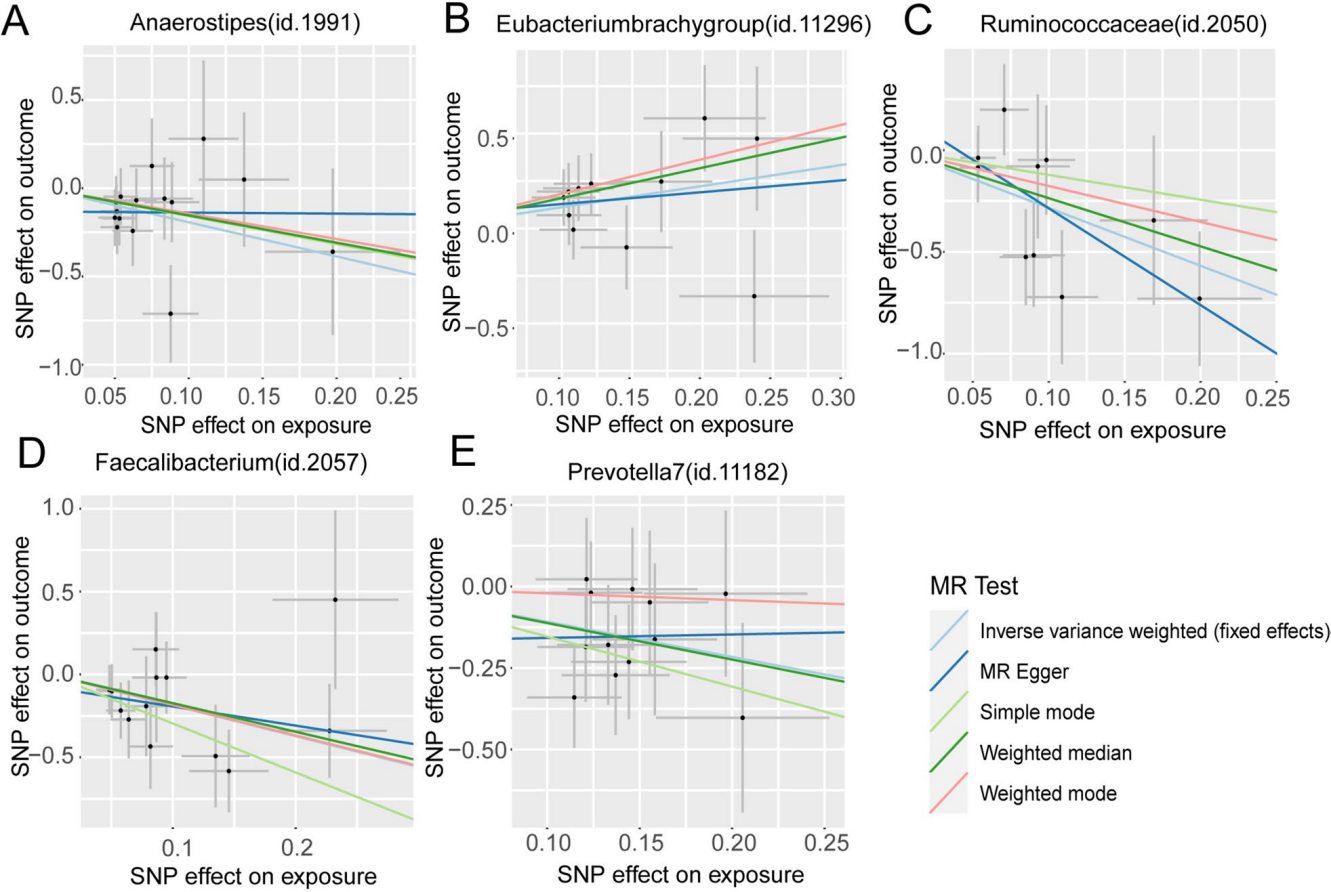
Scatter plots for the causal association between gut microbiota and GBM identified by IVW-FE method

**Fig. 4:**
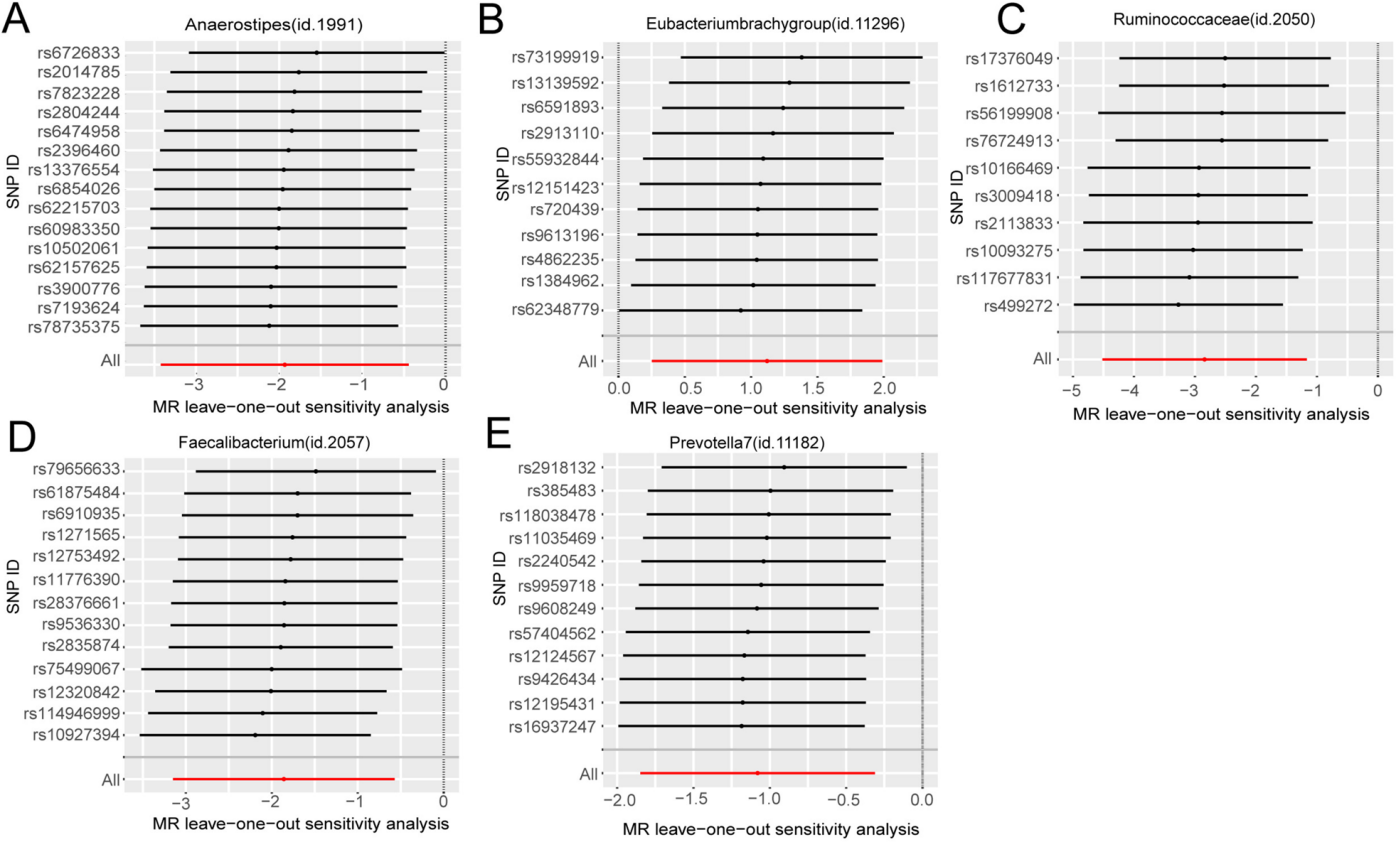
Leave-one-out plots for the causal association between gut microbiota and GBM identified by IVW-FE method

In addition, we conducted a leave-one-out sensitivity analysis for these 11 groups and presented the final results in Table 1, Figs. 3 and 4. And the details of SNPs were shown in Table 2. According to both the Inverse Variance Weighting and weighted median estimates, *Eubacteriumbrachygroup* exhibited a risk factor associated with GBM (IVW, *P*=0.011, OR=3.066, 95%CI=1.287-7.308; Weighted median, *P*=0.007, OR=4.969, 95%CI: 1.554-15.890). Furthermore, the IVW results for *Anaerostipes* (IVW, *P*=0.011, OR=0.145, 95%CI:0.033-0.642) , *Faecalibacterium* (IVW, *P*=0.005, OR=0.156, 95%CI=0.043-0.565), Prevotella7 (IVW, *P*=0.006, OR=0.340, 95%CI=0.158-0.732), and *Ruminococcaceae* (IVW, *P*=0.001, OR=0.058, 95%CI=0.011-0.304) with GBM remained causal associations even after leave-one-out sensitivity analysis, signifying a protective effect on GBM for all four.

**Table 1:** Results of all MR analyses with causality

Name (id)	No.of SNP	pleiotropy test (*p*. value)	Cochrane's Q heterogeneity test(Q_pval)	MR method	p.val	OR	or_lci95	or_uci95
Genus *Eubacteriumbrachygroup*(id.11296)	11	0.758	0.600	ivw-fe	0.011	3.066	1.287	7.308
				MR Egger	0.707	1.873	0.079	44.533
				Simple mode	0.104	6.224	0.840	46.133
				Weighted mode	0.106	6.224	0.826	46.931
				Weighted median	0.007	4.969	1.554	15.890
Genus *Anaerostipes*(id.1991)	15	0.398	0.841	ivw-fe	0.011	0.145	0.033	0.642
				MR Egger	0.980	0.942	0.011	80.970
				Simple mode	0.361	0.205	0.008	5.490
				Weighted mode	0.364	0.237	0.012	4.809
				Weighted median	0.122	0.214	0.030	1.511
Genus *Faecalibacterium*(id.2057)	13	0.558	0.648	ivw-fe	0.005	0.156	0.043	0.565
				MR Egger	0.408	0.316	0.023	4.380
				Simple mode	0.090	0.052	0.002	1.207
				Weighted mode	0.150	0.159	0.015	1.658
				Weighted median	0.096	0.178	0.023	1.355
Genus *Prevotella7*(id.11182)	12	0.627	0.886	ivw-fe	0.006	0.340	0.158	0.732
				MR Egger	0.966	1.112	0.010	122.089
				Simple mode	0.112	0.215	0.038	1.231
				Weighted mode	0.792	0.812	0.179	3.686
				Weighted median	0.034	0.326	0.116	0.917
Family *Ruminococcaceae*(id.2050)	10	0.332	0.413	ivw-fe	0.001	0.058	0.011	0.304
				MR Egger	0.048	0.009	0.000	0.468
				Simple mode	0.545	0.296	0.007	13.148
				Weighted mode	0.317	0.171	0.007	4.495
				Weighted median	0.040	0.094	0.010	0.897

*No.of SNP* Number of SNPs being used as IVs., *ivw-fe* Fixed-effects inverse variance weighting, *OR* Odds Ratio; or_lci95-or_uci95, 95% confidence interval; Significant *P*. value was marked in red

**Table 2:** Detailed information on SNPs used in MR analyses

Bacterial taxa	SNP	Effect allele	Other allele	F	palindromic	exposure			outcome		
						Beta	SE	pval	Beta	SE	pval
Genus Eubacteriumbrachygroup(id.11296)	rs12151423	A	G	19.869	FALSE	0.101	0.023	8.29E-06	0.163	0.149	0.272
	rs13139592	T	C	19.910	FALSE	-0.146	0.033	8.12E-06	0.099	0.221	0.653
	rs1384962	A	G	20.613	FALSE	0.121	0.027	5.62E-06	0.236	0.161	0.142
	rs2913110	C	T	21.004	FALSE	0.105	0.023	4.58E-06	0.070	0.156	0.654
	rs4862235	G	A	21.553	FALSE	0.105	0.023	3.44E-06	0.194	0.150	0.195
	rs55932844	A	G	22.357	FALSE	-0.171	0.036	2.26E-06	-0.247	0.266	0.352
	rs62348779	T	C	21.666	FALSE	-0.201	0.043	3.25E-06	-0.581	0.281	0.039
	rs6591893	G	A	20.281	FALSE	0.108	0.024	6.69E-06	-0.007	0.157	0.967
	rs720439	A	G	19.845	FALSE	-0.112	0.025	8.40E-06	-0.212	0.173	0.221
	rs73199919	T	C	19.848	FALSE	-0.237	0.053	8.38E-06	0.357	0.349	0.307
	rs9613196	T	A	20.398	TRUE	-0.239	0.053	6.29E-06	-0.474	0.379	0.212
Genus Faecalibacterium(id.2057)	rs10927394	G	T	20.549	FALSE	-0.232	0.051	5.81E-06	-0.451	0.538	0.402
	rs114946999	C	T	20.649	FALSE	-0.086	0.019	5.52E-06	-0.152	0.224	0.499
	rs11776390	T	C	20.793	FALSE	-0.078	0.017	5.12E-06	0.191	0.302	0.527
	rs12320842	C	G	33.453	TRUE	0.095	0.016	7.30E-09	-0.018	0.217	0.933
	rs1271565	C	T	23.195	FALSE	-0.058	0.012	1.46E-06	0.217	0.170	0.200
	rs12753492	A	C	18.295	FALSE	0.064	0.015	1.89E-05	-0.271	0.235	0.248
	rs2835874	T	C	19.440	FALSE	-0.087	0.020	1.04E-05	0.018	0.390	0.963
	rs28376661	C	G	21.305	TRUE	0.050	0.011	3.92E-06	-0.100	0.162	0.538
	rs61875484	C	G	19.828	TRUE	0.082	0.018	8.47E-06	-0.434	0.255	0.089
	rs6910935	A	G	23.699	FALSE	0.135	0.028	1.13E-06	-0.492	0.310	0.112
	rs75499067	C	T	23.900	FALSE	0.228	0.047	1.01E-06	-0.340	0.284	0.231
	rs79656633	T	C	20.326	FALSE	0.146	0.032	6.53E-06	-0.582	0.249	0.019
	rs9536330	T	C	20.026	FALSE	-0.048	0.011	7.64E-06	0.092	0.149	0.537
Genus Prevotella7(id.11182)	rs11035469	A	G	21.365	FALSE	-0.144	0.031	3.80E-06	0.231	0.176	0.188
	rs118038478	A	G	19.239	FALSE	0.206	0.047	1.15E-05	-0.403	0.291	0.166
	rs12124567	A	G	19.448	FALSE	-0.121	0.028	1.03E-05	-0.022	0.187	0.905
	rs12195431	T	C	19.741	FALSE	0.197	0.044	8.87E-06	-0.022	0.255	0.931
	rs16937247	G	C	17.273	TRUE	0.146	0.035	3.24E-05	-0.008	0.188	0.966
	rs2240542	C	T	21.312	FALSE	0.121	0.026	3.90E-06	-0.185	0.169	0.271
	rs2918132	C	T	20.228	FALSE	-0.115	0.025	6.88E-06	0.340	0.155	0.028
	rs385483	A	G	22.038	FALSE	0.137	0.029	2.67E-06	-0.272	0.184	0.139
	rs57404562	C	A	24.202	FALSE	0.155	0.032	8.67E-07	-0.049	0.220	0.826
	rs9426434	T	C	19.713	FALSE	-0.124	0.028	9.00E-06	0.019	0.157	0.903
	rs9608249	A	G	22.130	FALSE	-0.158	0.034	2.55E-06	0.162	0.233	0.486
	rs9959718	G	A	23.333	FALSE	0.133	0.028	1.36E-06	-0.179	0.184	0.330
Family Ruminococcaceae(id.2050)	rs10093275	C	T	20.960	FALSE	0.053	0.012	4.69E-06	-0.038	0.158	0.809
	rs10166469	T	C	19.686	FALSE	-0.053	0.012	9.12E-06	0.086	0.170	0.612
	rs117677831	G	A	27.172	FALSE	0.098	0.019	1.86E-07	-0.049	0.268	0.856
	rs1612733	T	C	20.862	FALSE	0.109	0.024	4.94E-06	-0.722	0.329	0.028
	rs17376049	T	C	24.306	FALSE	0.085	0.017	8.22E-07	-0.525	0.236	0.026
	rs2113833	T	C	22.701	FALSE	0.169	0.036	1.89E-06	-0.345	0.415	0.405
	rs2426816	T	A	20.514	TRUE	-0.048	0.011	5.92E-06	0.104	0.149	0.483
	rs3009418	C	A	19.489	FALSE	0.093	0.021	1.01E-05	-0.080	0.354	0.822
	rs499272	G	C	19.382	TRUE	0.071	0.016	1.07E-05	0.198	0.224	0.377
	rs56199908	T	C	23.584	FALSE	-0.199	0.041	1.20E-06	0.730	0.331	0.027
	rs76724913	T	G	19.682	FALSE	0.090	0.020	9.15E-06	-0.517	0.254	0.042
Genus Anaerostipes(id.1991)	rs10502061	A	G	18.944	FALSE	0.084	0.019	1.35E-05	-0.059	0.233	0.800
	rs13376554	A	T	18.522	TRUE	0.197	0.046	1.68E-05	-0.360	0.472	0.445
	rs2014785	T	C	21.125	FALSE	0.052	0.011	4.30E-06	-0.221	0.152	0.145
	rs2396460	T	C	21.897	FALSE	-0.051	0.011	2.88E-06	0.131	0.150	0.382
	rs2804244	A	G	22.882	FALSE	-0.053	0.011	1.72E-06	0.171	0.153	0.262
	rs3900776	G	A	21.675	FALSE	-0.110	0.024	3.23E-06	-0.280	0.443	0.528
	rs60983350	G	A	21.449	FALSE	-0.054	0.012	3.63E-06	0.048	0.161	0.766
	rs62157625	T	C	22.787	FALSE	0.089	0.019	1.81E-06	-0.079	0.227	0.727
	rs62215703	G	A	22.260	FALSE	0.064	0.014	2.38E-06	-0.069	0.181	0.704
	rs6474958	A	G	19.935	FALSE	-0.050	0.011	8.01E-06	0.167	0.160	0.295
	rs6726833	C	A	21.463	FALSE	-0.088	0.019	3.61E-06	0.711	0.276	0.010
	rs6854026	T	C	21.732	FALSE	-0.051	0.011	3.14E-06	0.081	0.150	0.588
	rs7193624	C	T	24.803	FALSE	0.075	0.015	6.35E-07	0.126	0.269	0.641
	rs7823228	G	C	20.669	TRUE	-0.062	0.014	5.46E-06	0.242	0.199	0.224
	rs78735375	A	C	20.262	FALSE	-0.137	0.031	6.75E-06	-0.049	0.380	0.898

*SNP* Single nucleotide polymorphism, *SE* Standard error

**Fig. 5:**
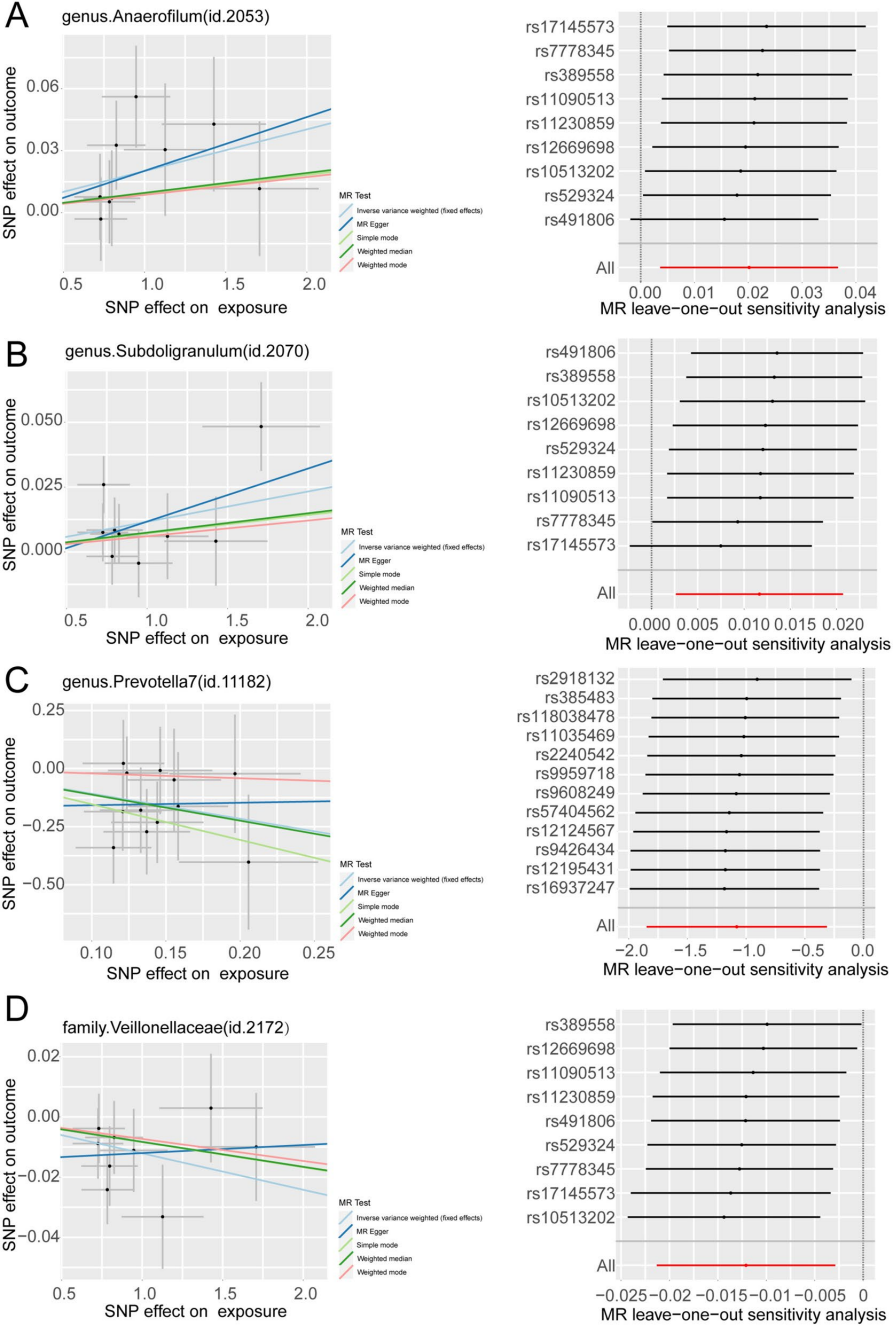
Scatter plots and leave-one-out plots of reverse mendelian randomization analysis

Utilizing the taxa mentioned above, we carried out a reverse MR analysis, with the GWAS data of bacteria serving as the exposure. Detailed information regarding the SNPs used as IVs and the results of the reverse MR analysis can be found in Tables 3, and 4 and Fig. 5. Detailed information regarding these SNPs can be found in Online Resource 2: Table S2. *Prevotella7, Anaerofilum*, *Subdoligranulum and Veillonellaceae* and GBM have a reverse causal relationship. Notably, Glioblastoma was associated with *Prevotella7*, which, in combination with a forward Mendelian randomization analysis, suggests a bidirectional causal relationship between *Prevotella7* and glioblastoma, raising the possibility that *Prevotella7* may be of screening and therapeutic significance for glioblastoma.

**Table 3:** The results of reverse MR analysis

Outcome Name (id)	No.of SNP	pleiotropy test (*p*. value)	Cochrane's Q heterogeneity test(Q_pval)	MR method	*p*.val	OR	or_lci95	or_uci95
Genus *Prevotella7*(id.11182)	9	0.621	0.861	ivw-fe	0.004	0.972	0.953	0.991
				MR Egger	0.232	0.955	0.890	1.023
				Simple mode	0.139	0.966	0.928	1.007
				Weighted mode	0.101	0.964	0.928	1.002
				Weighted median	0.015	0.969	0.945	0.994
Genus *Anaerofilum*(id.2053)	9	0.842	0.773	ivw-fe	0.017	1.020	1.004	1.037
				MR Egger	0.410	1.026	0.968	1.088
				Simple mode	0.623	1.009	0.974	1.045
				Weighted mode	0.619	1.009	0.976	1.042
				Weighted median	0.376	1.010	0.988	1.031
Genus *Subdoligranulum*(id.2070)	9	0.603	0.393	ivw-fe	0.009	1.012	1.003	1.021
				MR Egger	0.264	1.021	0.988	1.055
				Simple mode	0.523	1.007	0.986	1.028
				Weighted mode	0.562	1.006	0.987	1.026
				Weighted median	0.262	1.007	0.994	1.021
Family *Veillonellaceae*(id.2172)	9	0.380	0.752	ivw-fe	0.010	0.988	0.979	0.997
				MR Egger	0.876	1.003	0.971	1.035
				Simple mode	0.455	0.992	0.971	1.013
				Weighted mode	0.419	0.993	0.976	1.010
				Weighted median	0.198	0.992	0.979	1.004

No.of SNP, number of SNPs being used as IVs.; ivw-fe, fixed-effects inverse variance weighting; OR, Odds Ratio; or_lci95-or_uci95, 95% confidence interval; Significant *P*. value was marked in red

**Table 4:** The detail information of SNPs in reverse MR analysis

Bacterial taxa (outcome)	SNP	Effect allele	Other allele	BETA		SE		P.val		palindromic	F
				exposure	outcome	exposure	outcome	exposure	outcome		
genus.*Anaerofilum*(id.2053)	rs10513202	G	A	1.427	0.043	0.321	0.033	8.58E-06	0.188	FALSE	20
	rs11090513	T	G	0.798	0.007	0.175	0.023	5.00E-06	0.763	FALSE	21
	rs11230859	A	G	-0.725	-0.008	0.158	0.021	4.18E-06	0.713	FALSE	21
	rs12669698	C	T	1.127	0.030	0.255	0.032	9.73E-06	0.342	FALSE	20
	rs17145573	A	G	1.708	0.012	0.365	0.033	2.87E-06	0.721	FALSE	22
	rs389558	T	C	-0.783	-0.005	0.160	0.020	9.74E-07	0.793	FALSE	24
	rs491806	C	A	-0.948	-0.056	0.211	0.025	6.92E-06	0.023	FALSE	20
	rs529324	A	G	-0.826	-0.033	0.179	0.022	4.16E-06	0.130	FALSE	21
	rs7778345	G	A	0.730	-0.003	0.163	0.020	7.05E-06	0.880	FALSE	20
genus.*Prevotella7*(id.11182)	rs10513202	G	A	1.427	-0.019	0.321	0.038	8.58E-06	0.621	FALSE	20
	rs11090513	T	G	0.798	-0.007	0.175	0.028	5.00E-06	0.793	FALSE	21
	rs11230859	A	G	-0.725	0.022	0.158	0.025	4.18E-06	0.377	FALSE	21
	rs12669698	C	T	1.127	-0.039	0.255	0.038	9.73E-06	0.297	FALSE	20
	rs17145573	A	G	1.708	-0.072	0.365	0.039	2.87E-06	0.066	FALSE	22
	rs389558	T	C	-0.783	0.020	0.160	0.025	9.74E-07	0.430	FALSE	24
	rs491806	C	A	-0.948	0.040	0.211	0.029	6.92E-06	0.171	FALSE	20
	rs529324	A	G	-0.826	0.051	0.179	0.026	4.16E-06	0.048	FALSE	21
	rs7778345	G	A	0.730	0.010	0.163	0.024	7.05E-06	0.691	FALSE	20
genus.*Subdoligranulum*(id.2070)	rs10513202	G	A	1.427	0.004	0.321	0.017	8.58E-06	0.809	FALSE	20
	rs11090513	T	G	0.798	0.008	0.175	0.013	5.00E-06	0.502	FALSE	21
	rs11230859	A	G	-0.725	-0.008	0.158	0.011	4.18E-06	0.498	FALSE	21
	rs12669698	C	T	1.127	0.006	0.255	0.017	9.73E-06	0.716	FALSE	20
	rs17145573	A	G	1.708	0.048	0.365	0.017	2.87E-06	0.005	FALSE	22
	rs389558	T	C	-0.783	0.002	0.160	0.011	9.74E-07	0.876	FALSE	24
	rs491806	C	A	-0.948	0.004	0.211	0.013	6.92E-06	0.741	FALSE	20
	rs529324	A	G	-0.826	-0.007	0.179	0.012	4.16E-06	0.549	FALSE	21
	rs7778345	G	A	0.730	0.026	0.163	0.011	7.05E-06	0.018	FALSE	20
family.*Veillonellaceae*(id.2172)	rs10513202	G	A	1.427	0.003	0.321	0.018	8.58E-06	0.871	FALSE	20
	rs11090513	T	G	0.798	-0.016	0.175	0.013	5.00E-06	0.216	FALSE	21
	rs11230859	A	G	-0.725	0.009	0.158	0.012	4.18E-06	0.451	FALSE	21
	rs12669698	C	T	1.127	-0.033	0.255	0.017	9.73E-06	0.055	FALSE	20
	rs17145573	A	G	1.708	-0.010	0.365	0.018	2.87E-06	0.578	FALSE	22
	rs389558	T	C	-0.783	0.024	0.160	0.011	9.74E-07	0.034	FALSE	24
	rs491806	C	A	-0.948	0.011	0.211	0.014	6.92E-06	0.420	FALSE	20
	rs529324	A	G	-0.826	0.007	0.179	0.012	4.16E-06	0.574	FALSE	21
	rs7778345	G	A	0.730	-0.004	0.163	0.012	7.05E-06	0.740	FALSE	20

*SNP* Single nucleotide polymorphism, *SE* Standard error

## Discussion

In this study, our primary objective was to employ a Mendelian Randomization analysis to rigorously assess the causal relationship between gut microbiota and Glioblastoma. To accomplish this, we leveraged the aggregated gut microbiota statistics derived from the extensive GWAS meta-analysis conducted by the MiBioGen consortium. Simultaneously, we utilized aggregated GBM statistics, which were sourced from the IEU OpenGWAS project release data, thereby ensuring that our study was underpinned by a robust dataset. We identified four specific microbial taxa, namely *Eubacteriumbrachygroup, Anaerostipes, Faecalibacterium, Prevotella7,* and *Ruminococcaceae*, that exhibited significant associations with GBM. Remarkably, four of these taxa, *Anaerostipes, Faecalibacterium, Prevotella7*, and *Ruminococcaceae*, demonstrated a protective effect against GBM, suggesting their potential as therapeutic targets or indicators of reduced risk for this aggressive brain tumor. However, there are few studies on the effects of these microbiota and their metabolites on the development of GBM through specific pathways. Chronic inflammation has long been recognized as a factor associated with tumorigenesis, and GBM is no exception to this phenomenon. Therefore, our discussion is grounded in existing studies that investigate the responses of the flora within the organism, particularly focusing on inflammatory and immune responses.

The insights from previous research studies provide valuable context and support for our findings regarding *Eubacteriumbrachygroup* in the context of cancer. Wang et al. in 2021 highlighted the potential role of Eubacterium in cancer initiation by promoting inflammation. This observation underscores the complexity of microbial influences on cancer development and suggests that certain microbiota may create an inflammatory microenvironment that can contribute to carcinogenesis [32]. Moreover, the study conducted by Sama Rezasoltani et al. in 2022, which investigated saliva and fecal samples from colorectal cancer (CRC) patients compared to healthy controls, identified *Eubacteriumbrachygroup* as one of the top three genera showing differential abundance [33]. This finding strongly suggests that *Eubacteriumbrachygroup* may indeed have a role in cancer development and progression. Eubacterium has been identified as a producer of acetic acid and butyric acid [34]. Acetic acid and butyric acid, categorized as short-chain fatty acids (SCFAs), play pivotal roles in cellular processes. Acetic acid, in conjunction with glucose, participates in the tricarboxylic acid (TCA) cycle, influencing the production of acetyl-CoA [15]. Acetyl-CoA, an active substance, can drive GBM proliferation and survival through the acetylation of RICTOR by mTORC2 [16]. Moreover, SCFAs, including acetic acid and butyric acid, have been shown to stimulate the production of regulatory T cells [35]. These cells contribute to the immunosuppressive environment of GBM by producing interleukin-10 (IL-10) and transforming growth factor-beta (TGF-β) [36]. The findings from our MR analysis align with this, suggesting that Eubacterium is a potential risk factor for GBM.

However, the 2023 study by Reza N et al. introduces a perspective that contrasts with our findings [37]. According to their research, *Eubacterium* is associated with the release of a peptide recognized by TCC88. TCC88 is demonstrated to target glioblastoma neoantigens and exhibit a strong response to various peptides derived from glioblastoma. Additionally, it shows a robust response to a broad range of bacterial sources and targets derived from the intestinal microbiota. This capacity enables TCC88 to trigger substantial Tumor-Infiltrating Lymphocytes (TIL) responses and even elicit cross-reactive T cell responses against tumor targets in peripheral blood memory T cells based on the peptides secreted by the intestinal microbiota, ultimately playing an anti-tumor role.

*Anaerostipes*, identified for its potential role in inhibiting colorectal cancer (CRC) progression by regulating the immune response, aligns with our present findings indicating its protective effect against glioblastoma [38]. Its appearance as a differential genus in both GBM and CRC studies underscores its potential significance as a common microbial factor across different cancer types, emphasizing its relevance in cancer biology. In contrast, *Ruminococcaceae*, another microbial taxon of interest, has showcased diverse implications in health and disease. The discovery of the metabolite isoamylamine (IAA) produced by *Ruminococcaceae*, with its potential to induce S100A8 and result in microglial cell death, adds a layer of complexity [39, 40]. Microglia, innate immune cells in the brain, play a crucial role in glioblastoma, polarizing between pro-inflammatory (M1) and anti-inflammatory (M2) phenotypic profiles. The M2 cells' secretion of cytokines such as IL10, EGF, and VEGF can inhibit T cell proliferation and promote tumor growth and angiogenesis [41]. A higher M2/M1 ratio in GBM often indicates a poorer survival rate [42]. Considering the combined performance of *Anaerostipes* and *Ruminococcaceae* in previous studies and the results of this study, it is speculated that these genera may influence M2-type microglial polarization and potentially lead to M2-type microglial death in the tumor microenvironment of GBM patients by releasing specific metabolites through the damaged blood-brain barrier. Moreover, the higher relative abundances of *Ruminococcaceae* observed in melanoma patients who responded positively to anti-PD-1 immunotherapy raise intriguing questions about the potential role of the *Ruminococcaceae* family in modulating the immune response and influencing outcomes in cancer treatment. The MR results indicating *Ruminococcaceae* as a protective factor for GBM prompt further exploration into whether increasing the abundance of *Ruminococcaceae* microbiota could enhance the efficacy of immunotherapy for GBM. This observation underscores the need for additional research to unravel the specific mechanisms through which the *Ruminococcaceae* family may impact the immune response and contribute to improved outcomes in GBM treatment.

The multifaceted roles of the *Faecalibacterium* genus in human health and disease have garnered increasing attention in recent years. Notably, *Faecalibacterium* consists of two distinct phylogroups, and while their precise physiological functions remain partially understood, research has pointed toward their involvement in crucial processes, particularly in the context of inflammatory bowel disease (IBD) [43, 44]. *Faecalibacterium*'s association with IBD suggests its potential role in modulating the anti-inflammatory response, which is relevant in various disease contexts, including cancer. Indeed, studies have begun to unveil intriguing links between *Faecalibacterium* abundance and other forms of cancer, such as prostate cancer [45]. The connection between *Faecalibacterium* and prostate cancer highlights the intricate interplay between the gut microbiota and cancer development. These findings suggest that alterations in the relative abundance of *Faecalibacterium* may be linked to the pathogenesis of certain cancers, opening avenues for further investigation into the mechanistic underpinnings of these associations.

The identification of *Prevotella7* as being associated with glioblastoma through both forward and reverse Mendelian Randomization analyses is a noteworthy discovery. *Prevotella7* is a specific strain or subgenus of gut microorganisms belonging to the *Prevotella* genus, and it has previously been recognized for its roles in dietary and intestinal health [46]. In 2022, Arsenij U et al. found *Prevotella* in mouse glioblastoma tissue and found that *Prevotella* can produce Alpha-galactosylceramide (α-GalCer), a metabolite that stimulates invariant natural killer T (iNKT) cells to exert anticancer effects [47]. This finding is consistent with our findings suggesting that increasing the abundance of Prevonella may play a role in immunotherapy for glioblastoma. The consistent association of Prevotella7 with GBM in the study implies its potential as a valuable biomarker for early identification and treatment of GBM. This finding is particularly intriguing as it aligns with advanced research conducted in other disease contexts. For example, *Prevotella7* has shown promise in improving the prognosis of CRC, suggesting that it might have broader implications in cancer biology beyond GBM. Additionally, its utility as a diagnostic marker for oral squamous cell carcinoma (OSCC), with the ability to predict 80% of cases, further underscores its potential as a versatile biomarker in various cancer types [48, 49]. The identification of *Prevotella7* as a common factor in multiple cancer types suggests its significance in the broader context of cancer research and diagnosis. However, it's essential to conduct further research to understand the mechanistic underpinnings of *Prevotella7*'s involvement in these different cancer types and to evaluate its clinical utility as a diagnostic or prognostic marker.

The theoretical basis of this study is the gut-brain axis proposed in previous studies [17]. Human gut microbiota can modulate the development and function of the central nervous system (CNS) through gut-brain axis [50–52]. And this study has several advantages. MR analysis is a method used to establish causal inferences by leveraging existing genetic variations in nature. It employs randomization simulation, treating assignment to a control group, thereby enhancing our ability to formulate causal hypotheses with increased confidence. MR analysis employs genetic variation as an instrumental variable, effectively mitigating issues related to confounding and reverse causation [53]. This approach contributes to a clearer elucidation of relationships between variables. Observational studies frequently encounter numerous limitations, including confounding, selection bias, and memory bias. To some extent, MR analysis can circumvent these issues and offer more dependable causal inferences [54]. Genetic variation in the gut microbiota was derived from the most extensive meta-analysis of global genomic studies, ensuring robust instrumental variables for MR analysis. It identifies causal relationships between gut microbiota and GBM through MR analysis, reducing confounding factors and reversing causality in causal inference. A two-sample MR design was used and non-overlapping exposure and outcome pooled data were utilized to reduce bias [55].

However, Since the number of SNPs screened by the significance threshold (*P* < 5 × 10-8) of the conventional GWAS was too small, we raised the significance threshold accordingly for sensitivity analysis and to avoid horizontal pleiotropy. Moreover, MR analysis is affected by demographic and genetic sequencing errors, and the present study population is European, which makes it limited. Finally, although MR analysis can provide evidence of causality, explaining the biological mechanisms may still be complex and requires further experimental studies.

## Conclusion

In this bidirectional Mendelian randomization study, we identified five gut microbiota species with causal associations to glioblastoma. Especially significant was the bidirectional causal relationship observed with *Prevotella7*, suggesting potential implications for glioblastoma screening and treatment. To comprehensively comprehend *Prevotella7*'s protective role against glioblastoma and unveil its precise protective mechanisms, additional randomized controlled trials are necessary.

### Supplementary Information

**Additional file 1: Table S1.** Detailed information of SNPs from different taxa (exposure).

**Addtiional file 2: TableS2.** The detail information of SNP on Glioblastoma (exposure).

## Data Availability

The datasets generated during and/or analysed during the current study are available from the corresponding author on reasonable request.

## Abbreviations

MR: Mendelian randomization
GBM: Glioblastoma
IVW: inverse variance weighting
GWAS: genome-wide association study
IEU: Integrative Epidemiology Unit
SNPs: Single nucleotide polymorphisms
IVs: instrumental variables
OR: Odds Ratio
95%CI: 95% confidence interval
CRC: Colorectal cancer
IBD: Inflammatory bowel disease
OSCC: Oral squamous cell carcinoma
CNS: Central nervous system
