# Supplementary material for: Investigating the causal impact of gut microbiota on glioblastoma: a bidirectional Mendelian randomization study
Source: BMC Genomics. 2023 Dec 18;24:784. doi: 10.1186/s12864-023-09885-2 (PMC10726622; doi:10.1186/s12864-023-09885-2)
Supplement: Supplementary file 1 — Additional file 1: Table S1. Detailed information of SNPs from different taxa (exposure). [file 12864_2023_9885_MOESM1_ESM.docx]

**Table S1 Detailed information of SNPs from different taxa (exposure).**

| Bacterial taxa (exposure) | SNP | Effect allele | Other allele | palindromic | beta | | se | | pval | | F |
| --- | --- | --- | --- | --- | --- | --- | --- | --- | --- | --- | --- |
|  |  |  |  |  | exposure | outcome | exposure | outcome | exposure | outcome |  |
| Genus Clostridiuminnocuumgroup(id.14397) | rs10074000 | T | C | FALSE | -0.103 | -0.119 | 0.023 | 0.155 | 6.43E-06 | 0.443 | 20.357 |
|  | rs10506058 | A | G | FALSE | 0.100 | 0.112 | 0.022 | 0.150 | 7.03E-06 | 0.456 | 20.184 |
|  | rs1942371 | G | A | FALSE | -0.158 | -0.011 | 0.034 | 0.229 | 3.84E-06 | 0.963 | 21.343 |
|  | rs1948423 | T | A | TRUE | -0.109 | 0.080 | 0.023 | 0.157 | 3.37E-06 | 0.611 | 21.596 |
|  | rs40656 | C | T | FALSE | 0.143 | 0.087 | 0.031 | 0.188 | 4.50E-06 | 0.644 | 21.040 |
|  | rs4869133 | G | A | FALSE | -0.181 | -0.063 | 0.041 | 0.198 | 1.03E-05 | 0.751 | 19.448 |
|  | rs61267978 | T | C | FALSE | 0.147 | -0.100 | 0.032 | 0.228 | 4.57E-06 | 0.662 | 21.010 |
|  | rs6577484 | G | A | FALSE | 0.160 | 0.411 | 0.036 | 0.249 | 8.76E-06 | 0.099 | 19.764 |
|  | rs6890185 | T | C | FALSE | 0.113 | -0.143 | 0.023 | 0.159 | 1.14E-06 | 0.370 | 23.669 |
|  | rs71564433 | T | A | TRUE | -0.127 | -0.053 | 0.027 | 0.187 | 3.94E-06 | 0.776 | 21.296 |
|  | rs77845139 | A | G | FALSE | -0.115 | 0.055 | 0.026 | 0.174 | 7.78E-06 | 0.750 | 19.992 |
| Genus Eubacteriumbrachygroup(id.11296) | rs12151423 | A | G | FALSE | 0.101 | 0.163 | 0.023 | 0.149 | 8.29E-06 | 0.272 | 19.869 |
|  | rs13139592 | T | C | FALSE | -0.146 | 0.099 | 0.033 | 0.221 | 8.12E-06 | 0.653 | 19.910 |
|  | rs1384962 | A | G | FALSE | 0.121 | 0.236 | 0.027 | 0.161 | 5.62E-06 | 0.142 | 20.613 |
|  | rs2913110 | C | T | FALSE | 0.105 | 0.070 | 0.023 | 0.156 | 4.58E-06 | 0.654 | 21.004 |
|  | rs4862235 | G | A | FALSE | 0.105 | 0.194 | 0.023 | 0.150 | 3.44E-06 | 0.195 | 21.553 |
|  | rs55932844 | A | G | FALSE | -0.171 | -0.247 | 0.036 | 0.266 | 2.26E-06 | 0.352 | 22.357 |
|  | rs62348779 | T | C | FALSE | -0.201 | -0.581 | 0.043 | 0.281 | 3.25E-06 | 0.039 | 21.666 |
|  | rs6591893 | G | A | FALSE | 0.108 | -0.007 | 0.024 | 0.157 | 6.69E-06 | 0.967 | 20.281 |
|  | rs720439 | A | G | FALSE | -0.112 | -0.212 | 0.025 | 0.173 | 8.40E-06 | 0.221 | 19.845 |
|  | rs73199919 | T | C | FALSE | -0.237 | 0.357 | 0.053 | 0.349 | 8.38E-06 | 0.307 | 19.848 |
|  | rs9613196 | T | A | TRUE | -0.239 | -0.474 | 0.053 | 0.379 | 6.29E-06 | 0.212 | 20.398 |
| Genus Eubacteriumcoprostanoligenesgroup(id.11375) | rs1020520 | T | G | FALSE | -0.059 | 0.026 | 0.013 | 0.208 | 8.84E-06 | 0.902 | 19.747 |
|  | rs10444197 | A | G | FALSE | -0.051 | 0.084 | 0.011 | 0.156 | 8.26E-06 | 0.590 | 19.877 |
|  | rs11052069 | T | C | FALSE | 0.048 | -0.059 | 0.011 | 0.150 | 9.37E-06 | 0.695 | 19.636 |
|  | rs11720857 | C | T | FALSE | 0.063 | 0.280 | 0.014 | 0.194 | 1.27E-05 | 0.149 | 19.059 |
|  | rs11808093 | T | C | FALSE | 0.079 | 0.265 | 0.017 | 0.207 | 5.39E-06 | 0.200 | 20.694 |
|  | rs12906958 | C | T | FALSE | -0.053 | -0.040 | 0.012 | 0.164 | 4.25E-06 | 0.808 | 21.149 |
|  | rs17159861 | C | T | FALSE | 0.096 | -0.329 | 0.017 | 0.242 | 1.10E-08 | 0.175 | 32.654 |
|  | rs2644213 | G | A | FALSE | 0.054 | -0.029 | 0.012 | 0.162 | 8.82E-06 | 0.858 | 19.752 |
|  | rs4076415 | T | G | FALSE | 0.052 | -0.102 | 0.011 | 0.155 | 3.00E-06 | 0.510 | 21.815 |
|  | rs62024432 | C | T | FALSE | -0.077 | -0.064 | 0.017 | 0.252 | 7.74E-06 | 0.801 | 20.002 |
|  | rs6762473 | C | A | FALSE | 0.052 | 0.150 | 0.011 | 0.156 | 3.44E-06 | 0.338 | 21.552 |
|  | rs76898927 | G | A | FALSE | 0.123 | -0.160 | 0.027 | 0.328 | 3.84E-06 | 0.625 | 21.341 |
|  | rs79895140 | T | C | FALSE | -0.064 | 0.165 | 0.014 | 0.238 | 5.59E-06 | 0.488 | 20.623 |
|  | rs9648214 | T | C | FALSE | -0.083 | -0.192 | 0.016 | 0.264 | 4.57E-07 | 0.467 | 25.438 |
| Genus Eubacteriumeligensgroup(id.14372) | rs158115 | G | C | TRUE | 0.092 | -0.152 | 0.019 | 0.224 | 1.57E-06 | 0.498 | 23.057 |
|  | rs182318 | G | A | FALSE | -0.082 | 0.220 | 0.020 | 0.271 | 2.47E-05 | 0.416 | 17.785 |
|  | rs2200429 | A | G | FALSE | -0.089 | -0.288 | 0.020 | 0.252 | 7.53E-06 | 0.254 | 20.054 |
|  | rs265534 | T | G | FALSE | -0.056 | -0.003 | 0.012 | 0.149 | 2.69E-06 | 0.985 | 22.024 |
|  | rs4583233 | A | C | FALSE | 0.067 | 0.026 | 0.013 | 0.164 | 1.69E-07 | 0.876 | 27.363 |
|  | rs56080211 | C | T | FALSE | 0.123 | 0.237 | 0.028 | 0.280 | 1.34E-05 | 0.398 | 18.953 |
|  | rs6923695 | T | G | FALSE | 0.103 | -0.211 | 0.023 | 0.297 | 6.82E-06 | 0.478 | 20.243 |
|  | rs72839198 | C | G | TRUE | 0.156 | -0.352 | 0.038 | 0.262 | 3.41E-05 | 0.180 | 17.174 |
|  | rs74606150 | C | G | TRUE | -0.196 | -0.237 | 0.043 | 0.370 | 3.92E-06 | 0.522 | 21.302 |
| Genus Eubacteriumfissicatenagroup(id.14373) | rs10147907 | T | G | FALSE | 0.172 | 0.039 | 0.040 | 0.282 | 1.36E-05 | 0.891 | 18.922 |
|  | rs11818408 | G | A | FALSE | 0.106 | 0.043 | 0.024 | 0.153 | 8.04E-06 | 0.779 | 19.928 |
|  | rs11876297 | T | C | FALSE | 0.131 | 0.181 | 0.028 | 0.169 | 3.06E-06 | 0.284 | 21.779 |
|  | rs151257695 | A | G | FALSE | 0.210 | 0.157 | 0.045 | 0.288 | 4.10E-06 | 0.585 | 21.217 |
|  | rs1768152 | T | C | FALSE | 0.139 | -0.606 | 0.032 | 0.244 | 1.03E-05 | 0.013 | 19.462 |
|  | rs2733072 | G | A | FALSE | 0.110 | 0.243 | 0.023 | 0.149 | 1.57E-06 | 0.103 | 23.064 |
|  | rs3771393 | C | T | FALSE | 0.131 | 0.348 | 0.027 | 0.187 | 9.27E-07 | 0.062 | 24.074 |
|  | rs6934739 | A | G | FALSE | 0.111 | -0.068 | 0.025 | 0.158 | 1.04E-05 | 0.666 | 19.442 |
|  | rs7104872 | G | A | FALSE | 0.139 | -0.158 | 0.029 | 0.234 | 2.05E-06 | 0.500 | 22.548 |
| Genus Eubacteriumhalliigroup(id.11338) | rs10501370 | C | T | FALSE | -0.116 | 0.341 | 0.025 | 0.308 | 4.69E-06 | 0.268 | 20.958 |
|  | rs10798999 | C | T | FALSE | 0.060 | 0.099 | 0.013 | 0.169 | 2.07E-06 | 0.558 | 22.532 |
|  | rs10808115 | A | C | FALSE | -0.050 | -0.025 | 0.011 | 0.148 | 4.39E-06 | 0.868 | 21.085 |
|  | rs117748144 | T | C | FALSE | -0.127 | -0.200 | 0.029 | 0.333 | 1.04E-05 | 0.547 | 19.437 |
|  | rs13116360 | T | C | FALSE | 0.154 | -0.157 | 0.030 | 0.301 | 2.15E-07 | 0.602 | 26.896 |
|  | rs17074066 | T | C | FALSE | -0.081 | 0.404 | 0.019 | 0.514 | 1.70E-05 | 0.432 | 18.495 |
|  | rs17474256 | G | A | FALSE | 0.081 | 0.263 | 0.018 | 0.251 | 1.12E-05 | 0.295 | 19.297 |
|  | rs281379 | A | G | FALSE | -0.050 | -0.096 | 0.011 | 0.151 | 8.43E-06 | 0.526 | 19.838 |
|  | rs28584818 | A | G | FALSE | 0.126 | -0.143 | 0.027 | 0.278 | 2.67E-06 | 0.607 | 22.041 |
|  | rs60254196 | A | G | FALSE | -0.052 | -0.170 | 0.011 | 0.150 | 2.96E-06 | 0.258 | 21.844 |
|  | rs630939 | C | T | FALSE | -0.051 | 0.095 | 0.011 | 0.151 | 8.57E-06 | 0.529 | 19.806 |
|  | rs6550770 | T | C | FALSE | -0.198 | 0.807 | 0.044 | 0.371 | 7.97E-06 | 0.030 | 19.945 |
|  | rs74018587 | C | T | FALSE | 0.209 | 0.091 | 0.044 | 0.378 | 1.86E-06 | 0.810 | 22.734 |
|  | rs76496924 | A | T | TRUE | 0.154 | 0.489 | 0.035 | 0.281 | 1.16E-05 | 0.082 | 19.234 |
|  | rs78056098 | G | T | FALSE | -0.051 | -0.252 | 0.011 | 0.155 | 8.18E-06 | 0.104 | 19.895 |
|  | rs949971 | T | G | FALSE | -0.054 | 0.091 | 0.012 | 0.158 | 3.28E-06 | 0.565 | 21.646 |
| Genus Eubacteriumnodatumgroup(id.11297) | rs10263623 | C | T | FALSE | 0.193 | -0.174 | 0.044 | 0.374 | 1.05E-05 | 0.642 | 19.425 |
|  | rs10458299 | T | C | FALSE | -0.188 | -0.151 | 0.042 | 0.280 | 7.69E-06 | 0.591 | 20.013 |
|  | rs11006576 | A | G | FALSE | -0.110 | 0.130 | 0.025 | 0.150 | 7.48E-06 | 0.384 | 20.067 |
|  | rs113893692 | C | T | FALSE | -0.185 | 0.070 | 0.040 | 0.227 | 4.50E-06 | 0.759 | 21.038 |
|  | rs34297067 | A | G | FALSE | -0.187 | 0.188 | 0.034 | 0.210 | 4.41E-08 | 0.371 | 29.959 |
|  | rs61841040 | G | T | FALSE | 0.161 | -0.112 | 0.034 | 0.186 | 2.58E-06 | 0.548 | 22.108 |
|  | rs6818880 | A | G | FALSE | -0.110 | 0.122 | 0.025 | 0.150 | 7.62E-06 | 0.417 | 20.030 |
|  | rs77910827 | C | T | FALSE | 0.202 | -0.131 | 0.041 | 0.239 | 1.07E-06 | 0.583 | 23.807 |
|  | rs7827125 | C | T | FALSE | 0.122 | 0.150 | 0.027 | 0.166 | 6.56E-06 | 0.368 | 20.317 |
|  | rs7880204 | T | C | FALSE | -0.125 | -0.173 | 0.028 | 0.172 | 5.19E-06 | 0.315 | 20.766 |
|  | rs9425984 | T | C | FALSE | -0.130 | 0.115 | 0.029 | 0.179 | 8.40E-06 | 0.520 | 19.845 |
| Genus Eubacteriumoxidoreducensgroup(id.11339) | rs12129908 | C | A | FALSE | 0.089 | -0.129 | 0.020 | 0.151 | 6.67E-06 | 0.396 | 20.285 |
|  | rs12423772 | G | T | FALSE | 0.141 | -0.312 | 0.030 | 0.216 | 1.77E-06 | 0.147 | 22.834 |
|  | rs1425962 | G | C | TRUE | 0.091 | 0.004 | 0.020 | 0.153 | 6.28E-06 | 0.981 | 20.401 |
|  | rs2973294 | G | T | FALSE | 0.092 | -0.038 | 0.020 | 0.150 | 2.29E-06 | 0.803 | 22.331 |
|  | rs34561138 | G | A | FALSE | 0.216 | -0.490 | 0.046 | 0.383 | 2.61E-06 | 0.201 | 22.087 |
|  | rs440215 | C | T | FALSE | 0.093 | 0.007 | 0.020 | 0.150 | 1.79E-06 | 0.965 | 22.812 |
| Genus Eubacteriumrectalegroup(id.14374) | rs10248854 | C | A | FALSE | -0.053 | -0.148 | 0.011 | 0.152 | 3.29E-06 | 0.330 | 21.641 |
|  | rs10797540 | A | G | FALSE | 0.050 | -0.063 | 0.011 | 0.150 | 3.45E-06 | 0.675 | 21.551 |
|  | rs10892089 | C | G | TRUE | -0.064 | 0.085 | 0.014 | 0.191 | 5.70E-06 | 0.656 | 20.585 |
|  | rs117151453 | G | C | TRUE | -0.113 | -0.116 | 0.024 | 0.239 | 3.39E-06 | 0.627 | 21.582 |
|  | rs143694765 | T | C | FALSE | 0.087 | -0.162 | 0.020 | 0.246 | 1.09E-05 | 0.510 | 19.349 |
|  | rs16960159 | C | G | TRUE | -0.157 | 0.178 | 0.034 | 0.254 | 3.20E-06 | 0.483 | 21.691 |
|  | rs2884897 | A | G | FALSE | -0.129 | -0.016 | 0.029 | 0.404 | 7.61E-06 | 0.969 | 20.034 |
|  | rs314726 | T | C | FALSE | 0.053 | 0.128 | 0.011 | 0.150 | 1.37E-06 | 0.392 | 23.329 |
|  | rs35398954 | A | G | FALSE | -0.090 | -0.054 | 0.017 | 0.203 | 2.44E-07 | 0.791 | 26.653 |
|  | rs3980709 | A | T | TRUE | -0.062 | -0.274 | 0.014 | 0.195 | 9.43E-06 | 0.159 | 19.623 |
|  | rs59427698 | A | G | FALSE | -0.058 | 0.079 | 0.013 | 0.190 | 1.07E-05 | 0.677 | 19.379 |
|  | rs62547233 | A | G | FALSE | 0.054 | 0.200 | 0.012 | 0.165 | 8.12E-06 | 0.227 | 19.908 |
| Genus Eubacteriumruminantiumgroup(id.11340) | rs10131724 | A | C | FALSE | -0.200 | -0.337 | 0.041 | 0.251 | 1.43E-06 | 0.180 | 23.234 |
|  | rs10923018 | G | A | FALSE | 0.073 | 0.065 | 0.016 | 0.150 | 6.36E-06 | 0.667 | 20.378 |
|  | rs112375806 | T | A | TRUE | 0.143 | -0.123 | 0.029 | 0.218 | 1.10E-06 | 0.572 | 23.744 |
|  | rs11637981 | G | T | FALSE | -0.073 | -0.010 | 0.016 | 0.150 | 5.28E-06 | 0.948 | 20.733 |
|  | rs13025464 | T | C | FALSE | -0.074 | -0.360 | 0.016 | 0.152 | 6.79E-06 | 0.018 | 20.252 |
|  | rs139749 | C | T | FALSE | -0.085 | -0.269 | 0.017 | 0.158 | 8.60E-07 | 0.088 | 24.219 |
|  | rs16891896 | G | A | FALSE | -0.175 | -0.319 | 0.039 | 0.266 | 7.64E-06 | 0.230 | 20.027 |
|  | rs17519472 | C | T | FALSE | 0.108 | -0.127 | 0.023 | 0.217 | 4.08E-06 | 0.559 | 21.228 |
|  | rs209813 | G | A | FALSE | -0.103 | -0.108 | 0.024 | 0.211 | 1.20E-05 | 0.609 | 19.166 |
|  | rs2116427 | A | G | FALSE | 0.091 | 0.101 | 0.018 | 0.170 | 5.78E-07 | 0.552 | 24.983 |
|  | rs2229917 | A | G | FALSE | 0.154 | -0.481 | 0.032 | 0.371 | 2.14E-06 | 0.194 | 22.467 |
|  | rs2418654 | C | T | FALSE | -0.075 | -0.137 | 0.017 | 0.152 | 6.32E-06 | 0.368 | 20.388 |
|  | rs2817174 | C | T | FALSE | -0.073 | -0.242 | 0.016 | 0.152 | 7.26E-06 | 0.112 | 20.125 |
|  | rs57340348 | T | C | FALSE | -0.098 | -0.043 | 0.021 | 0.187 | 3.91E-06 | 0.818 | 21.311 |
|  | rs606117 | A | G | FALSE | 0.083 | 0.182 | 0.018 | 0.167 | 3.94E-06 | 0.276 | 21.296 |
|  | rs6676699 | G | T | FALSE | -0.089 | -0.161 | 0.020 | 0.163 | 6.16E-06 | 0.321 | 20.439 |
|  | rs7000472 | A | G | FALSE | -0.076 | -0.009 | 0.017 | 0.151 | 3.96E-06 | 0.951 | 21.284 |
|  | rs72836424 | C | T | FALSE | -0.140 | 0.723 | 0.030 | 0.243 | 3.32E-06 | 0.003 | 21.624 |
|  | rs73139629 | A | C | FALSE | -0.115 | -0.237 | 0.025 | 0.255 | 3.44E-06 | 0.352 | 21.555 |
| Genus Eubacteriumventriosumgroup(id.11341) | rs11617697 | A | G | FALSE | -0.143 | 0.107 | 0.029 | 0.328 | 5.50E-07 | 0.745 | 25.080 |
|  | rs12964517 | G | A | FALSE | 0.059 | -0.029 | 0.012 | 0.165 | 1.97E-06 | 0.859 | 22.624 |
|  | rs13082419 | C | T | FALSE | -0.072 | -0.222 | 0.016 | 0.238 | 9.25E-06 | 0.351 | 19.661 |
|  | rs16884680 | G | T | FALSE | -0.091 | 0.118 | 0.019 | 0.245 | 2.32E-06 | 0.632 | 22.309 |
|  | rs35179274 | C | T | FALSE | -0.063 | -0.005 | 0.014 | 0.192 | 5.36E-06 | 0.980 | 20.703 |
|  | rs3809430 | T | C | FALSE | -0.055 | -0.118 | 0.012 | 0.160 | 3.68E-06 | 0.462 | 21.426 |
|  | rs57199565 | T | C | FALSE | 0.078 | -0.195 | 0.016 | 0.197 | 1.02E-06 | 0.323 | 23.896 |
|  | rs6048195 | A | T | TRUE | -0.060 | 0.310 | 0.012 | 0.156 | 2.25E-07 | 0.047 | 26.807 |
|  | rs66746423 | C | T | FALSE | 0.075 | -0.044 | 0.016 | 0.206 | 5.23E-06 | 0.832 | 20.749 |
|  | rs66830358 | T | A | TRUE | 0.053 | 0.029 | 0.012 | 0.151 | 7.39E-06 | 0.850 | 20.088 |
|  | rs6704822 | A | G | FALSE | 0.074 | 0.133 | 0.017 | 0.222 | 9.25E-06 | 0.549 | 19.661 |
|  | rs72783037 | C | A | FALSE | 0.066 | -0.264 | 0.014 | 0.183 | 4.56E-06 | 0.149 | 21.012 |
|  | rs73615400 | T | C | FALSE | -0.096 | -0.162 | 0.019 | 0.252 | 7.65E-07 | 0.521 | 24.443 |
|  | rs78250280 | G | A | FALSE | 0.075 | 0.027 | 0.016 | 0.213 | 5.10E-06 | 0.897 | 20.798 |
|  | rs78869071 | C | T | FALSE | 0.098 | -0.227 | 0.023 | 0.270 | 1.34E-05 | 0.401 | 18.958 |
|  | rs876734 | C | T | FALSE | -0.062 | -0.149 | 0.013 | 0.167 | 3.04E-06 | 0.371 | 21.793 |
|  | rs9316536 | T | G | FALSE | -0.082 | -0.119 | 0.018 | 0.215 | 8.11E-06 | 0.581 | 19.911 |
| Genus Eubacteriumxylanophilumgroup(id.14375) | rs10140184 | A | C | FALSE | 0.058 | -0.118 | 0.013 | 0.152 | 4.69E-06 | 0.438 | 20.959 |
|  | rs10917203 | A | C | FALSE | 0.061 | -0.054 | 0.013 | 0.152 | 2.87E-06 | 0.725 | 21.898 |
|  | rs112176119 | C | T | FALSE | -0.113 | -0.247 | 0.025 | 0.258 | 3.90E-06 | 0.338 | 21.312 |
|  | rs12980122 | C | G | TRUE | -0.108 | 0.073 | 0.024 | 0.291 | 5.51E-06 | 0.802 | 20.652 |
|  | rs13239072 | G | A | FALSE | 0.069 | -0.052 | 0.014 | 0.167 | 1.46E-06 | 0.757 | 23.197 |
|  | rs17830032 | G | A | FALSE | -0.161 | 0.027 | 0.031 | 0.279 | 2.32E-07 | 0.924 | 26.749 |
|  | rs1999224 | G | T | FALSE | -0.095 | -0.058 | 0.020 | 0.247 | 3.11E-06 | 0.815 | 21.749 |
|  | rs2012708 | A | G | FALSE | 0.057 | -0.004 | 0.013 | 0.156 | 6.28E-06 | 0.981 | 20.401 |
|  | rs2213117 | T | G | FALSE | 0.088 | -0.017 | 0.019 | 0.204 | 3.42E-06 | 0.935 | 21.568 |
|  | rs4654122 | C | G | TRUE | 0.055 | -0.217 | 0.012 | 0.149 | 7.51E-06 | 0.145 | 20.058 |
|  | rs75586835 | A | G | FALSE | -0.114 | -0.231 | 0.026 | 0.293 | 1.39E-05 | 0.430 | 18.888 |
|  | rs79582700 | C | G | TRUE | -0.095 | -0.002 | 0.020 | 0.228 | 1.94E-06 | 0.994 | 22.649 |
| Genus Ruminococcusgauvreauiigroup(id.11342) | rs10931481 | G | A | FALSE | 0.061 | -0.094 | 0.013 | 0.164 | 2.93E-06 | 0.568 | 21.861 |
|  | rs11750752 | C | T | FALSE | 0.071 | 0.203 | 0.016 | 0.167 | 5.64E-06 | 0.226 | 20.606 |
|  | rs12079579 | A | G | FALSE | 0.096 | 0.107 | 0.021 | 0.266 | 7.61E-06 | 0.687 | 20.034 |
|  | rs12539819 | C | T | FALSE | 0.111 | -0.010 | 0.024 | 0.302 | 4.28E-06 | 0.974 | 21.137 |
|  | rs1391597 | C | T | FALSE | 0.059 | 0.195 | 0.012 | 0.152 | 2.28E-06 | 0.201 | 22.345 |
|  | rs2047242 | A | G | FALSE | -0.068 | 0.087 | 0.013 | 0.177 | 4.31E-07 | 0.622 | 25.552 |
|  | rs2105937 | A | G | FALSE | 0.058 | -0.308 | 0.013 | 0.156 | 5.63E-06 | 0.049 | 20.610 |
|  | rs2166943 | A | C | FALSE | 0.057 | 0.044 | 0.012 | 0.150 | 4.41E-06 | 0.773 | 21.077 |
|  | rs289410 | G | A | FALSE | -0.065 | -0.096 | 0.014 | 0.165 | 2.50E-06 | 0.561 | 22.168 |
|  | rs431418 | A | G | FALSE | -0.095 | 0.262 | 0.021 | 0.255 | 6.55E-06 | 0.304 | 20.321 |
|  | rs71386687 | T | G | FALSE | 0.121 | 0.166 | 0.024 | 0.238 | 3.92E-07 | 0.485 | 25.733 |
|  | rs73802842 | C | A | FALSE | 0.074 | 0.117 | 0.017 | 0.175 | 1.41E-05 | 0.503 | 18.859 |
|  | rs9870933 | A | G | FALSE | 0.062 | 0.180 | 0.013 | 0.152 | 8.19E-07 | 0.237 | 24.313 |
| Genus Ruminococcusgnavusgroup(id.14376) | rs11597105 | A | G | FALSE | 0.115 | 0.022 | 0.025 | 0.188 | 4.76E-06 | 0.907 | 20.930 |
|  | rs11864644 | T | C | FALSE | -0.140 | -0.456 | 0.032 | 0.236 | 1.12E-05 | 0.053 | 19.297 |
|  | rs12136548 | C | T | FALSE | 0.090 | -0.041 | 0.020 | 0.164 | 4.42E-06 | 0.804 | 21.074 |
|  | rs12989336 | G | A | FALSE | -0.085 | -0.365 | 0.019 | 0.166 | 6.60E-06 | 0.028 | 20.305 |
|  | rs13163520 | G | A | FALSE | -0.127 | -0.126 | 0.023 | 0.192 | 5.15E-08 | 0.512 | 29.661 |
|  | rs2909242 | C | A | FALSE | -0.091 | -0.019 | 0.018 | 0.158 | 7.10E-07 | 0.903 | 24.588 |
|  | rs3124783 | A | G | FALSE | -0.116 | -0.283 | 0.025 | 0.220 | 3.22E-06 | 0.200 | 21.682 |
|  | rs4388134 | C | T | FALSE | -0.090 | -0.010 | 0.020 | 0.166 | 8.74E-06 | 0.950 | 19.769 |
|  | rs62167033 | T | C | FALSE | 0.185 | -0.664 | 0.040 | 0.370 | 2.93E-06 | 0.073 | 21.861 |
|  | rs78399089 | T | C | FALSE | 0.144 | 0.055 | 0.033 | 0.233 | 9.76E-06 | 0.814 | 19.558 |
|  | rs934940 | A | C | FALSE | -0.105 | 0.050 | 0.023 | 0.217 | 4.75E-06 | 0.818 | 20.934 |
|  | rs9872758 | T | C | FALSE | 0.085 | 0.077 | 0.018 | 0.149 | 1.53E-06 | 0.604 | 23.113 |
| Genus Ruminococcustorquesgroup(id.14377) | rs10967781 | C | A | FALSE | 0.051 | 0.104 | 0.011 | 0.164 | 7.37E-06 | 0.524 | 20.095 |
|  | rs12434631 | A | G | FALSE | 0.075 | -0.463 | 0.015 | 0.243 | 1.12E-06 | 0.056 | 23.711 |
|  | rs1475330 | T | C | FALSE | 0.052 | 0.008 | 0.012 | 0.174 | 9.47E-06 | 0.964 | 19.615 |
|  | rs158487 | A | G | FALSE | 0.053 | -0.259 | 0.012 | 0.165 | 4.86E-06 | 0.118 | 20.893 |
|  | rs1972694 | T | A | TRUE | -0.061 | 0.091 | 0.014 | 0.194 | 7.75E-06 | 0.639 | 19.999 |
|  | rs35866622 | T | C | FALSE | -0.061 | -0.145 | 0.011 | 0.157 | 2.23E-08 | 0.354 | 31.285 |
|  | rs4073731 | T | C | FALSE | 0.065 | -0.187 | 0.014 | 0.202 | 4.56E-06 | 0.354 | 21.013 |
|  | rs60603763 | C | G | TRUE | 0.124 | -0.275 | 0.028 | 0.273 | 6.29E-06 | 0.314 | 20.397 |
|  | rs73130967 | A | T | TRUE | 0.077 | 0.628 | 0.017 | 0.264 | 4.69E-06 | 0.017 | 20.961 |
|  | rs77034621 | T | G | FALSE | -0.152 | 0.591 | 0.034 | 0.568 | 6.42E-06 | 0.298 | 20.359 |
|  | rs773123 | T | A | TRUE | 0.082 | -0.423 | 0.017 | 0.227 | 2.14E-06 | 0.062 | 22.462 |
|  | rs8080469 | G | A | FALSE | 0.049 | -0.123 | 0.011 | 0.148 | 4.55E-06 | 0.407 | 21.019 |
|  | rs8141465 | A | G | FALSE | 0.048 | -0.188 | 0.011 | 0.155 | 7.65E-06 | 0.225 | 20.023 |
| Genus Actinomyces(id.423) | rs10967781 | C | A | FALSE | 0.051 | 0.104 | 0.011 | 0.164 | 7.37E-06 | 0.524 | 20.095 |
|  | rs12434631 | A | G | FALSE | 0.075 | -0.463 | 0.015 | 0.243 | 1.12E-06 | 0.056 | 23.711 |
|  | rs1475330 | T | C | FALSE | 0.052 | 0.008 | 0.012 | 0.174 | 9.47E-06 | 0.964 | 19.615 |
|  | rs158487 | A | G | FALSE | 0.053 | -0.259 | 0.012 | 0.165 | 4.86E-06 | 0.118 | 20.893 |
|  | rs1972694 | T | A | TRUE | -0.061 | 0.091 | 0.014 | 0.194 | 7.75E-06 | 0.639 | 19.999 |
|  | rs35866622 | T | C | FALSE | -0.061 | -0.145 | 0.011 | 0.157 | 2.23E-08 | 0.354 | 31.285 |
|  | rs4073731 | T | C | FALSE | 0.065 | -0.187 | 0.014 | 0.202 | 4.56E-06 | 0.354 | 21.013 |
|  | rs60603763 | C | G | TRUE | 0.124 | -0.275 | 0.028 | 0.273 | 6.29E-06 | 0.314 | 20.397 |
|  | rs73130967 | A | T | TRUE | 0.077 | 0.628 | 0.017 | 0.264 | 4.69E-06 | 0.017 | 20.961 |
|  | rs77034621 | T | G | FALSE | -0.152 | 0.591 | 0.034 | 0.568 | 6.42E-06 | 0.298 | 20.359 |
|  | rs773123 | T | A | TRUE | 0.082 | -0.423 | 0.017 | 0.227 | 2.14E-06 | 0.062 | 22.462 |
|  | rs8080469 | G | A | FALSE | 0.049 | -0.123 | 0.011 | 0.148 | 4.55E-06 | 0.407 | 21.019 |
|  | rs8141465 | A | G | FALSE | 0.048 | -0.188 | 0.011 | 0.155 | 7.65E-06 | 0.225 | 20.023 |
| Genus Adlercreutzia(id.812) | rs10967781 | C | A | FALSE | 0.051 | 0.104 | 0.011 | 0.164 | 7.37E-06 | 0.524 | 20.095 |
|  | rs12434631 | A | G | FALSE | 0.075 | -0.463 | 0.015 | 0.243 | 1.12E-06 | 0.056 | 23.711 |
|  | rs1475330 | T | C | FALSE | 0.052 | 0.008 | 0.012 | 0.174 | 9.47E-06 | 0.964 | 19.615 |
|  | rs158487 | A | G | FALSE | 0.053 | -0.259 | 0.012 | 0.165 | 4.86E-06 | 0.118 | 20.893 |
|  | rs1972694 | T | A | TRUE | -0.061 | 0.091 | 0.014 | 0.194 | 7.75E-06 | 0.639 | 19.999 |
|  | rs35866622 | T | C | FALSE | -0.061 | -0.145 | 0.011 | 0.157 | 2.23E-08 | 0.354 | 31.285 |
|  | rs4073731 | T | C | FALSE | 0.065 | -0.187 | 0.014 | 0.202 | 4.56E-06 | 0.354 | 21.013 |
|  | rs60603763 | C | G | TRUE | 0.124 | -0.275 | 0.028 | 0.273 | 6.29E-06 | 0.314 | 20.397 |
|  | rs73130967 | A | T | TRUE | 0.077 | 0.628 | 0.017 | 0.264 | 4.69E-06 | 0.017 | 20.961 |
|  | rs77034621 | T | G | FALSE | -0.152 | 0.591 | 0.034 | 0.568 | 6.42E-06 | 0.298 | 20.359 |
|  | rs773123 | T | A | TRUE | 0.082 | -0.423 | 0.017 | 0.227 | 2.14E-06 | 0.062 | 22.462 |
|  | rs8080469 | G | A | FALSE | 0.049 | -0.123 | 0.011 | 0.148 | 4.55E-06 | 0.407 | 21.019 |
|  | rs8141465 | A | G | FALSE | 0.048 | -0.188 | 0.011 | 0.155 | 7.65E-06 | 0.225 | 20.023 |
| Genus Akkermansia(id.4037) | rs1046175 | C | G | TRUE | 0.113 | -0.328 | 0.026 | 0.291 | 1.06E-05 | 0.260 | 19.401 |
|  | rs11604400 | C | T | FALSE | -0.103 | 0.097 | 0.023 | 0.241 | 1.27E-05 | 0.688 | 19.060 |
|  | rs12522517 | A | T | TRUE | -0.105 | -0.174 | 0.023 | 0.213 | 7.86E-06 | 0.413 | 19.972 |
|  | rs13231526 | C | A | FALSE | 0.143 | -0.564 | 0.031 | 0.274 | 4.31E-06 | 0.040 | 21.123 |
|  | rs2147798 | C | G | TRUE | 0.092 | -0.033 | 0.019 | 0.173 | 1.55E-06 | 0.849 | 23.079 |
|  | rs34181676 | G | T | FALSE | -0.135 | 0.406 | 0.028 | 0.364 | 1.74E-06 | 0.265 | 22.867 |
|  | rs55719207 | G | A | FALSE | -0.070 | -0.042 | 0.016 | 0.153 | 9.66E-06 | 0.783 | 19.577 |
|  | rs6664405 | T | C | FALSE | -0.095 | 0.064 | 0.021 | 0.212 | 6.12E-06 | 0.761 | 20.451 |
|  | rs7680684 | C | T | FALSE | -0.083 | 0.123 | 0.017 | 0.157 | 7.95E-07 | 0.434 | 24.371 |
|  | rs80078995 | A | T | TRUE | -0.113 | -0.286 | 0.023 | 0.211 | 1.12E-06 | 0.175 | 23.704 |
|  | rs9490822 | C | T | FALSE | -0.073 | -0.162 | 0.016 | 0.149 | 2.42E-06 | 0.279 | 22.229 |
|  | rs9915817 | T | C | FALSE | 0.075 | 0.182 | 0.017 | 0.165 | 8.55E-06 | 0.269 | 19.810 |
| Genus Alistipes(id.968) | rs1107244 | G | A | FALSE | 0.076 | 0.288 | 0.017 | 0.276 | 9.37E-06 | 0.298 | 19.636 |
|  | rs11769002 | G | A | FALSE | -0.053 | 0.263 | 0.011 | 0.151 | 1.34E-06 | 0.082 | 23.368 |
|  | rs11958296 | A | G | FALSE | -0.098 | -0.155 | 0.022 | 0.357 | 6.94E-06 | 0.665 | 20.209 |
|  | rs12990744 | C | T | FALSE | -0.078 | 0.064 | 0.017 | 0.244 | 7.14E-06 | 0.795 | 20.156 |
|  | rs1689282 | A | C | FALSE | -0.052 | -0.100 | 0.011 | 0.160 | 5.01E-06 | 0.530 | 20.833 |
|  | rs2290844 | C | T | FALSE | 0.081 | -0.146 | 0.019 | 0.242 | 2.16E-05 | 0.547 | 18.042 |
|  | rs2875322 | T | C | FALSE | -0.058 | -0.179 | 0.013 | 0.199 | 9.87E-06 | 0.368 | 19.537 |
|  | rs34417064 | A | G | FALSE | -0.048 | -0.296 | 0.011 | 0.149 | 6.47E-06 | 0.047 | 20.344 |
|  | rs35909684 | A | C | FALSE | -0.085 | 0.206 | 0.019 | 0.285 | 1.08E-05 | 0.471 | 19.364 |
|  | rs4810359 | A | G | FALSE | -0.065 | 0.212 | 0.015 | 0.225 | 8.05E-06 | 0.345 | 19.927 |
|  | rs62576416 | T | C | FALSE | 0.049 | 0.008 | 0.011 | 0.153 | 7.14E-06 | 0.960 | 20.156 |
|  | rs7129639 | C | A | FALSE | -0.052 | -0.001 | 0.011 | 0.158 | 1.66E-06 | 0.996 | 22.948 |
|  | rs8130320 | A | G | FALSE | -0.049 | 0.065 | 0.011 | 0.150 | 4.82E-06 | 0.665 | 20.906 |
| Genus Allisonella(id.2174) | rs1901739 | T | G | FALSE | 0.116 | 0.007 | 0.025 | 0.149 | 3.22E-06 | 0.963 | 21.681 |
|  | rs35110698 | T | C | FALSE | -0.146 | 0.090 | 0.032 | 0.209 | 5.11E-06 | 0.669 | 20.797 |
|  | rs35778461 | C | T | FALSE | 0.147 | 0.161 | 0.030 | 0.177 | 7.99E-07 | 0.361 | 24.360 |
|  | rs594561 | C | T | FALSE | 0.112 | 0.069 | 0.025 | 0.148 | 8.22E-06 | 0.644 | 19.885 |
|  | rs602075 | A | G | FALSE | 0.169 | -0.324 | 0.030 | 0.168 | 1.27E-08 | 0.054 | 32.374 |
|  | rs6742198 | G | A | FALSE | 0.149 | -0.028 | 0.032 | 0.175 | 2.44E-06 | 0.872 | 22.210 |
|  | rs685403 | G | C | TRUE | -0.175 | 0.243 | 0.040 | 0.220 | 1.48E-05 | 0.269 | 18.763 |
|  | rs76904847 | G | A | FALSE | 0.149 | -0.204 | 0.033 | 0.196 | 9.19E-06 | 0.300 | 19.673 |
|  | rs7898615 | T | G | FALSE | 0.168 | -0.267 | 0.037 | 0.218 | 6.92E-06 | 0.221 | 20.214 |
| Genus Alloprevotella(id.961) | rs12675596 | G | T | FALSE | 0.146 | 0.150 | 0.029 | 0.181 | 5.41E-07 | 0.407 | 25.114 |
|  | rs17380632 | A | T | TRUE | 0.126 | 0.045 | 0.028 | 0.175 | 7.54E-06 | 0.799 | 20.051 |
|  | rs2154444 | T | G | FALSE | 0.138 | -0.038 | 0.031 | 0.169 | 7.67E-06 | 0.823 | 20.018 |
|  | rs34619204 | G | A | FALSE | -0.156 | 0.320 | 0.034 | 0.196 | 5.88E-06 | 0.103 | 20.528 |
|  | rs4364940 | A | G | FALSE | 0.126 | 0.277 | 0.028 | 0.160 | 7.48E-06 | 0.084 | 20.067 |
|  | rs4680035 | A | G | FALSE | -0.120 | 0.167 | 0.026 | 0.153 | 4.03E-06 | 0.274 | 21.250 |
|  | rs58212166 | A | G | FALSE | -0.162 | 0.080 | 0.036 | 0.193 | 6.88E-06 | 0.679 | 20.227 |
| Genus Anaerofilum(id.2053) | rs10794359 | T | C | FALSE | -0.095 | -0.304 | 0.020 | 0.151 | 2.00E-06 | 0.044 | 22.594 |
|  | rs1563175 | A | C | FALSE | 0.092 | -0.042 | 0.020 | 0.149 | 4.88E-06 | 0.777 | 20.884 |
|  | rs17012738 | T | G | FALSE | 0.090 | -0.007 | 0.020 | 0.148 | 6.43E-06 | 0.963 | 20.355 |
|  | rs17096874 | C | T | FALSE | -0.126 | -0.096 | 0.027 | 0.182 | 2.63E-06 | 0.598 | 22.071 |
|  | rs17105491 | G | C | TRUE | -0.193 | 0.140 | 0.041 | 0.306 | 2.65E-06 | 0.646 | 22.056 |
|  | rs356049 | G | A | FALSE | 0.133 | -0.192 | 0.029 | 0.299 | 4.75E-06 | 0.521 | 20.936 |
|  | rs4244069 | G | A | FALSE | -0.147 | 0.135 | 0.033 | 0.227 | 6.99E-06 | 0.553 | 20.197 |
|  | rs4506496 | G | A | FALSE | 0.103 | 0.029 | 0.021 | 0.164 | 1.28E-06 | 0.859 | 23.455 |
|  | rs712981 | A | C | FALSE | 0.101 | 0.003 | 0.020 | 0.153 | 6.84E-07 | 0.985 | 24.660 |
|  | rs79598899 | C | T | FALSE | 0.183 | 0.073 | 0.036 | 0.348 | 3.22E-07 | 0.834 | 26.116 |
|  | rs816292 | T | C | FALSE | -0.113 | 0.194 | 0.022 | 0.163 | 2.94E-07 | 0.233 | 26.288 |
|  | rs9299345 | T | C | FALSE | -0.136 | 0.225 | 0.030 | 0.252 | 6.45E-06 | 0.372 | 20.349 |
| Genus Anaerostipes(id.1991) | rs10502061 | A | G | FALSE | 0.084 | -0.059 | 0.019 | 0.233 | 1.35E-05 | 0.800 | 18.944 |
|  | rs13376554 | A | T | TRUE | 0.197 | -0.360 | 0.046 | 0.472 | 1.68E-05 | 0.445 | 18.522 |
|  | rs2014785 | T | C | FALSE | 0.052 | -0.221 | 0.011 | 0.152 | 4.30E-06 | 0.145 | 21.125 |
|  | rs2396460 | T | C | FALSE | -0.051 | 0.131 | 0.011 | 0.150 | 2.88E-06 | 0.382 | 21.897 |
|  | rs2804244 | A | G | FALSE | -0.053 | 0.171 | 0.011 | 0.153 | 1.72E-06 | 0.262 | 22.882 |
|  | rs3900776 | G | A | FALSE | -0.110 | -0.280 | 0.024 | 0.443 | 3.23E-06 | 0.528 | 21.675 |
|  | rs60983350 | G | A | FALSE | -0.054 | 0.048 | 0.012 | 0.161 | 3.63E-06 | 0.766 | 21.449 |
|  | rs62157625 | T | C | FALSE | 0.089 | -0.079 | 0.019 | 0.227 | 1.81E-06 | 0.727 | 22.787 |
|  | rs62215703 | G | A | FALSE | 0.064 | -0.069 | 0.014 | 0.181 | 2.38E-06 | 0.704 | 22.260 |
|  | rs6474958 | A | G | FALSE | -0.050 | 0.167 | 0.011 | 0.160 | 8.01E-06 | 0.295 | 19.935 |
|  | rs6726833 | C | A | FALSE | -0.088 | 0.711 | 0.019 | 0.276 | 3.61E-06 | 0.010 | 21.463 |
|  | rs6854026 | T | C | FALSE | -0.051 | 0.081 | 0.011 | 0.150 | 3.14E-06 | 0.588 | 21.732 |
|  | rs7193624 | C | T | FALSE | 0.075 | 0.126 | 0.015 | 0.269 | 6.35E-07 | 0.641 | 24.803 |
|  | rs7823228 | G | C | TRUE | -0.062 | 0.242 | 0.014 | 0.199 | 5.46E-06 | 0.224 | 20.669 |
|  | rs78735375 | A | C | FALSE | -0.137 | -0.049 | 0.031 | 0.380 | 6.75E-06 | 0.898 | 20.262 |
| Genus Anaerotruncus(id.2054) | rs10150232 | A | G | FALSE | 0.057 | -0.135 | 0.012 | 0.185 | 5.59E-06 | 0.467 | 20.622 |
|  | rs11018566 | A | G | FALSE | -0.156 | 1.040 | 0.037 | 0.346 | 1.91E-05 | 0.003 | 18.272 |
|  | rs115414803 | A | C | FALSE | -0.144 | -0.042 | 0.032 | 0.310 | 5.46E-06 | 0.893 | 20.669 |
|  | rs12056802 | C | G | TRUE | 0.077 | -0.215 | 0.018 | 0.222 | 1.24E-05 | 0.335 | 19.093 |
|  | rs1272208 | G | T | FALSE | -0.061 | -0.051 | 0.013 | 0.175 | 2.45E-06 | 0.770 | 22.202 |
|  | rs1431492 | C | T | FALSE | -0.065 | -0.119 | 0.015 | 0.202 | 7.45E-06 | 0.558 | 20.075 |
|  | rs17734739 | T | C | FALSE | 0.066 | 0.060 | 0.015 | 0.213 | 9.53E-06 | 0.780 | 19.603 |
|  | rs2704155 | T | A | TRUE | -0.106 | 1.131 | 0.023 | 3.557 | 6.06E-06 | 0.751 | 20.468 |
|  | rs2704155 | T | A | TRUE | -0.106 | 0.318 | 0.023 | 0.383 | 6.06E-06 | 0.406 | 20.468 |
|  | rs34449434 | A | C | FALSE | -0.050 | -0.017 | 0.011 | 0.157 | 1.17E-05 | 0.912 | 19.208 |
|  | rs4669806 | G | T | FALSE | 0.058 | -0.228 | 0.012 | 0.181 | 2.78E-06 | 0.209 | 21.962 |
|  | rs6494922 | A | G | FALSE | 0.090 | -0.304 | 0.020 | 0.330 | 8.00E-06 | 0.357 | 19.938 |
|  | rs6563550 | T | C | FALSE | 0.088 | 0.287 | 0.018 | 0.276 | 6.95E-07 | 0.298 | 24.629 |
|  | rs7155595 | C | A | FALSE | 0.054 | -0.105 | 0.012 | 0.162 | 5.73E-06 | 0.519 | 20.575 |
|  | rs7675045 | A | T | TRUE | -0.050 | -0.052 | 0.011 | 0.149 | 6.73E-06 | 0.726 | 20.269 |
|  | rs8005030 | C | T | FALSE | 0.055 | 0.284 | 0.012 | 0.158 | 2.54E-06 | 0.072 | 22.132 |
|  | rs9347879 | T | C | FALSE | 0.051 | 0.046 | 0.011 | 0.149 | 4.62E-06 | 0.756 | 20.988 |
| Genus Bacteroides(id.918) | rs11585893 | A | G | FALSE | -0.074 | -0.315 | 0.015 | 0.175 | 5.24E-07 | 0.072 | 25.175 |
|  | rs13207588 | A | G | FALSE | -0.059 | 0.248 | 0.013 | 0.191 | 6.40E-06 | 0.194 | 20.365 |
|  | rs1340391 | T | C | FALSE | -0.059 | -0.192 | 0.013 | 0.216 | 7.58E-06 | 0.375 | 20.040 |
|  | rs17619981 | T | G | FALSE | 0.088 | 0.255 | 0.019 | 0.217 | 2.46E-06 | 0.240 | 22.194 |
|  | rs2023437 | T | C | FALSE | -0.078 | 0.164 | 0.017 | 0.228 | 3.06E-06 | 0.472 | 21.780 |
|  | rs2366421 | T | A | TRUE | -0.053 | -0.177 | 0.012 | 0.191 | 6.50E-06 | 0.356 | 20.334 |
|  | rs28757219 | T | A | TRUE | 0.082 | -0.215 | 0.017 | 0.230 | 1.55E-06 | 0.351 | 23.089 |
|  | rs495004 | C | G | TRUE | -0.061 | -0.202 | 0.013 | 0.177 | 2.90E-06 | 0.254 | 21.881 |
|  | rs66474973 | G | T | FALSE | 0.081 | 0.169 | 0.016 | 0.244 | 7.81E-07 | 0.489 | 24.403 |
|  | rs66710942 | C | T | FALSE | 0.049 | -0.161 | 0.011 | 0.149 | 5.53E-06 | 0.279 | 20.644 |
|  | rs6795673 | C | T | FALSE | 0.054 | 0.236 | 0.011 | 0.149 | 3.11E-07 | 0.112 | 26.183 |
|  | rs9507307 | C | T | FALSE | 0.060 | 0.230 | 0.013 | 0.174 | 2.85E-06 | 0.187 | 21.912 |
| Genus Barnesiella(id.944) | rs11585893 | A | G | FALSE | -0.074 | -0.315 | 0.015 | 0.175 | 5.24E-07 | 0.072 | 25.175 |
|  | rs13207588 | A | G | FALSE | -0.059 | 0.248 | 0.013 | 0.191 | 6.40E-06 | 0.194 | 20.365 |
|  | rs1340391 | T | C | FALSE | -0.059 | -0.192 | 0.013 | 0.216 | 7.58E-06 | 0.375 | 20.040 |
|  | rs17619981 | T | G | FALSE | 0.088 | 0.255 | 0.019 | 0.217 | 2.46E-06 | 0.240 | 22.194 |
|  | rs2023437 | T | C | FALSE | -0.078 | 0.164 | 0.017 | 0.228 | 3.06E-06 | 0.472 | 21.780 |
|  | rs2366421 | T | A | TRUE | -0.053 | -0.177 | 0.012 | 0.191 | 6.50E-06 | 0.356 | 20.334 |
|  | rs28757219 | T | A | TRUE | 0.082 | -0.215 | 0.017 | 0.230 | 1.55E-06 | 0.351 | 23.089 |
|  | rs495004 | C | G | TRUE | -0.061 | -0.202 | 0.013 | 0.177 | 2.90E-06 | 0.254 | 21.881 |
|  | rs66474973 | G | T | FALSE | 0.081 | 0.169 | 0.016 | 0.244 | 7.81E-07 | 0.489 | 24.403 |
|  | rs66710942 | C | T | FALSE | 0.049 | -0.161 | 0.011 | 0.149 | 5.53E-06 | 0.279 | 20.644 |
|  | rs6795673 | C | T | FALSE | 0.054 | 0.236 | 0.011 | 0.149 | 3.11E-07 | 0.112 | 26.183 |
|  | rs9507307 | C | T | FALSE | 0.060 | 0.230 | 0.013 | 0.174 | 2.85E-06 | 0.187 | 21.912 |
| Genus Bilophila(id.3170) | rs11069458 | T | C | FALSE | -0.068 | -0.070 | 0.016 | 0.192 | 1.12E-05 | 0.715 | 19.293 |
|  | rs116261629 | G | C | TRUE | 0.128 | -0.535 | 0.026 | 0.256 | 9.50E-07 | 0.036 | 24.028 |
|  | rs1241171 | G | A | FALSE | -0.069 | 0.250 | 0.015 | 0.211 | 3.97E-06 | 0.235 | 21.281 |
|  | rs1571225 | C | T | FALSE | 0.083 | 0.213 | 0.017 | 0.201 | 1.26E-06 | 0.289 | 23.483 |
|  | rs1917709 | A | T | TRUE | 0.119 | -0.178 | 0.027 | 0.419 | 9.35E-06 | 0.671 | 19.639 |
|  | rs1969927 | G | A | FALSE | 0.056 | 0.106 | 0.013 | 0.158 | 8.67E-06 | 0.502 | 19.783 |
|  | rs2713349 | A | T | TRUE | 0.062 | 0.251 | 0.014 | 0.163 | 1.07E-05 | 0.123 | 19.389 |
|  | rs2728491 | G | T | FALSE | -0.063 | 0.272 | 0.014 | 0.174 | 6.83E-06 | 0.117 | 20.241 |
|  | rs3827020 | C | T | FALSE | 0.077 | 0.062 | 0.016 | 0.189 | 1.83E-06 | 0.745 | 22.766 |
|  | rs4798126 | G | A | FALSE | 0.073 | 0.100 | 0.017 | 0.189 | 1.28E-05 | 0.598 | 19.033 |
|  | rs542415 | T | C | FALSE | -0.061 | -0.097 | 0.013 | 0.152 | 4.25E-06 | 0.523 | 21.147 |
|  | rs60178956 | G | A | FALSE | -0.062 | 0.086 | 0.014 | 0.178 | 1.00E-05 | 0.628 | 19.502 |
|  | rs6793291 | C | A | FALSE | 0.113 | -0.451 | 0.024 | 0.335 | 3.08E-06 | 0.178 | 21.765 |
|  | rs72676854 | T | C | FALSE | 0.123 | 0.275 | 0.027 | 0.334 | 4.58E-06 | 0.410 | 21.007 |
|  | rs7802841 | C | A | FALSE | 0.067 | -0.112 | 0.014 | 0.162 | 1.14E-06 | 0.489 | 23.681 |
|  | rs8013541 | A | T | TRUE | -0.057 | -0.142 | 0.013 | 0.150 | 5.33E-06 | 0.345 | 20.716 |
|  | rs9899990 | A | G | FALSE | -0.103 | -0.127 | 0.023 | 0.270 | 1.13E-05 | 0.639 | 19.283 |
| Genus Blautia(id.1992) | rs11149971 | C | T | FALSE | 0.118 | 0.225 | 0.023 | 0.327 | 4.96E-07 | 0.491 | 25.281 |
|  | rs113271346 | C | T | FALSE | 0.078 | -0.348 | 0.017 | 0.266 | 5.43E-06 | 0.192 | 20.679 |
|  | rs115043014 | G | A | FALSE | -0.207 | -0.587 | 0.044 | 0.559 | 2.65E-06 | 0.294 | 22.056 |
|  | rs117001700 | T | C | FALSE | 0.196 | 0.059 | 0.044 | 0.601 | 8.48E-06 | 0.922 | 19.826 |
|  | rs12453000 | C | T | FALSE | 0.063 | -0.259 | 0.013 | 0.217 | 1.50E-06 | 0.233 | 23.143 |
|  | rs16892041 | T | C | FALSE | -0.062 | 0.117 | 0.014 | 0.182 | 1.13E-05 | 0.521 | 19.277 |
|  | rs2788271 | T | G | FALSE | -0.058 | 0.099 | 0.013 | 0.196 | 1.62E-05 | 0.614 | 18.592 |
|  | rs3005511 | A | G | FALSE | 0.050 | -0.050 | 0.011 | 0.162 | 6.08E-06 | 0.759 | 20.464 |
|  | rs4926264 | T | C | FALSE | 0.083 | 0.218 | 0.018 | 0.242 | 3.56E-06 | 0.368 | 21.487 |
|  | rs67794373 | C | T | FALSE | 0.060 | -0.275 | 0.012 | 0.178 | 1.09E-06 | 0.123 | 23.760 |
|  | rs682885 | A | G | FALSE | -0.049 | 0.052 | 0.011 | 0.158 | 4.32E-06 | 0.742 | 21.119 |
|  | rs72973581 | A | G | FALSE | 0.125 | -0.041 | 0.027 | 0.318 | 2.40E-06 | 0.898 | 22.242 |
|  | rs7860714 | A | G | FALSE | -0.050 | -0.055 | 0.011 | 0.157 | 4.85E-06 | 0.726 | 20.897 |
| Genus Butyricicoccus(id.2055) | rs10084203 | A | G | FALSE | 0.055 | 0.235 | 0.012 | 0.220 | 8.64E-06 | 0.285 | 19.791 |
|  | rs12034718 | A | G | FALSE | 0.070 | 0.040 | 0.016 | 0.179 | 9.34E-06 | 0.821 | 19.642 |
|  | rs12585793 | T | C | FALSE | -0.262 | -0.257 | 0.056 | 0.444 | 3.43E-06 | 0.564 | 21.558 |
|  | rs2017189 | G | T | FALSE | -0.051 | 0.011 | 0.011 | 0.149 | 4.25E-06 | 0.942 | 21.148 |
|  | rs4962426 | G | T | FALSE | 0.061 | -0.153 | 0.014 | 0.189 | 6.27E-06 | 0.416 | 20.403 |
|  | rs56221232 | T | C | FALSE | 0.083 | -0.143 | 0.017 | 0.247 | 7.56E-07 | 0.562 | 24.467 |
|  | rs62478070 | T | G | FALSE | 0.224 | 0.073 | 0.049 | 0.475 | 6.00E-06 | 0.878 | 20.488 |
|  | rs7322368 | T | C | FALSE | 0.082 | 0.022 | 0.018 | 0.258 | 8.45E-06 | 0.933 | 19.834 |
|  | rs75238760 | T | A | TRUE | 0.062 | -0.327 | 0.014 | 0.218 | 9.59E-06 | 0.133 | 19.592 |
| Genus Butyricimonas(id.945) | rs11228830 | A | G | FALSE | 0.135 | -0.297 | 0.030 | 0.272 | 5.82E-06 | 0.275 | 20.547 |
|  | rs113054641 | G | A | FALSE | -0.145 | 0.242 | 0.027 | 0.360 | 1.32E-07 | 0.501 | 27.842 |
|  | rs12304031 | G | A | FALSE | -0.086 | 0.005 | 0.020 | 0.225 | 1.17E-05 | 0.983 | 19.211 |
|  | rs12458763 | A | C | FALSE | 0.122 | 0.278 | 0.027 | 0.353 | 5.99E-06 | 0.430 | 20.492 |
|  | rs1862649 | G | A | FALSE | 0.113 | 0.080 | 0.025 | 0.294 | 5.16E-06 | 0.785 | 20.777 |
|  | rs2114713 | G | T | FALSE | 0.063 | 0.027 | 0.014 | 0.149 | 6.41E-06 | 0.859 | 20.362 |
|  | rs2642760 | G | C | TRUE | 0.071 | -0.169 | 0.015 | 0.161 | 9.28E-07 | 0.294 | 24.072 |
|  | rs270727 | G | C | TRUE | -0.069 | 0.060 | 0.015 | 0.161 | 4.16E-06 | 0.709 | 21.191 |
|  | rs326049 | C | G | TRUE | 0.076 | 0.212 | 0.017 | 0.170 | 5.83E-06 | 0.212 | 20.542 |
|  | rs62130338 | G | A | FALSE | -0.073 | 0.181 | 0.016 | 0.157 | 3.71E-06 | 0.249 | 21.412 |
|  | rs62390301 | T | C | FALSE | -0.087 | 0.083 | 0.017 | 0.186 | 5.99E-07 | 0.657 | 24.915 |
|  | rs7083431 | A | C | FALSE | 0.070 | -0.242 | 0.014 | 0.167 | 1.11E-06 | 0.146 | 23.725 |
|  | rs71428626 | G | T | FALSE | -0.133 | -0.348 | 0.029 | 0.412 | 4.34E-06 | 0.398 | 21.107 |
|  | rs72814525 | A | G | FALSE | 0.066 | 0.099 | 0.015 | 0.172 | 9.22E-06 | 0.566 | 19.666 |
|  | rs782080 | T | A | TRUE | 0.065 | -0.245 | 0.014 | 0.151 | 2.18E-06 | 0.104 | 22.433 |
|  | rs78453362 | A | G | FALSE | -0.149 | 0.282 | 0.033 | 0.474 | 4.87E-06 | 0.552 | 20.887 |
|  | rs9657374 | C | T | FALSE | 0.068 | 0.340 | 0.015 | 0.163 | 4.35E-06 | 0.038 | 21.106 |
| Genus CandidatusSoleaferrea(id.11350) | rs10090365 | A | G | FALSE | -0.083 | -0.015 | 0.018 | 0.148 | 4.02E-06 | 0.917 | 21.255 |
|  | rs10108780 | A | G | FALSE | -0.093 | -0.010 | 0.020 | 0.173 | 3.38E-06 | 0.955 | 21.587 |
|  | rs10809135 | T | C | FALSE | 0.083 | -0.050 | 0.018 | 0.151 | 4.73E-06 | 0.739 | 20.944 |
|  | rs11153159 | G | C | TRUE | -0.128 | -0.068 | 0.029 | 0.215 | 7.26E-06 | 0.752 | 20.124 |
|  | rs12500231 | A | T | TRUE | 0.081 | -0.096 | 0.018 | 0.149 | 8.40E-06 | 0.520 | 19.844 |
|  | rs2193878 | T | A | TRUE | 0.228 | -0.568 | 0.051 | 0.485 | 7.39E-06 | 0.242 | 20.091 |
|  | rs36160691 | G | A | FALSE | 0.104 | -0.065 | 0.024 | 0.164 | 1.17E-05 | 0.692 | 19.218 |
|  | rs386526 | C | G | TRUE | 0.082 | 0.210 | 0.018 | 0.147 | 5.50E-06 | 0.152 | 20.655 |
|  | rs4678258 | T | C | FALSE | 0.099 | -0.098 | 0.022 | 0.174 | 4.81E-06 | 0.575 | 20.912 |
|  | rs61825792 | T | C | FALSE | 0.112 | 0.080 | 0.023 | 0.203 | 1.30E-06 | 0.693 | 23.418 |
|  | rs6489992 | A | G | FALSE | -0.084 | -0.004 | 0.019 | 0.153 | 7.00E-06 | 0.979 | 20.192 |
|  | rs6494306 | A | G | FALSE | -0.097 | -0.038 | 0.021 | 0.159 | 6.05E-06 | 0.811 | 20.474 |
|  | rs6881988 | G | C | TRUE | -0.082 | 0.141 | 0.018 | 0.150 | 6.66E-06 | 0.349 | 20.290 |
|  | rs7400877 | T | C | FALSE | -0.095 | 0.098 | 0.021 | 0.182 | 7.84E-06 | 0.591 | 19.976 |
|  | rs830149 | C | G | TRUE | 0.185 | 0.131 | 0.040 | 0.316 | 3.35E-06 | 0.678 | 21.606 |
|  | rs9973954 | A | G | FALSE | 0.089 | 0.139 | 0.020 | 0.157 | 4.99E-06 | 0.375 | 20.842 |
| Genus Catenibacterium(id.2153) | rs12404911 | C | T | FALSE | 0.141 | -0.086 | 0.030 | 0.190 | 3.72E-06 | 0.649 | 21.406 |
|  | rs212393 | G | A | FALSE | -0.135 | 0.105 | 0.029 | 0.183 | 2.29E-06 | 0.566 | 22.332 |
|  | rs73128290 | A | G | FALSE | 0.130 | -0.084 | 0.028 | 0.163 | 5.14E-06 | 0.606 | 20.783 |
|  | rs7742829 | C | T | FALSE | 0.114 | 0.021 | 0.025 | 0.150 | 5.51E-06 | 0.888 | 20.652 |
| Genus ChristensenellaceaeR.7group(id.11283) | rs10461257 | A | G | FALSE | -0.055 | 0.073 | 0.012 | 0.159 | 6.13E-06 | 0.645 | 20.446 |
|  | rs17081797 | A | G | FALSE | -0.090 | 0.025 | 0.020 | 0.303 | 9.53E-06 | 0.934 | 19.602 |
|  | rs60954665 | T | G | FALSE | 0.050 | -0.285 | 0.011 | 0.148 | 6.91E-06 | 0.054 | 20.218 |
|  | rs62132810 | A | G | FALSE | -0.083 | 0.260 | 0.018 | 0.215 | 3.94E-06 | 0.225 | 21.293 |
|  | rs62190261 | A | C | FALSE | 0.096 | -0.489 | 0.021 | 0.266 | 8.07E-06 | 0.066 | 19.922 |
|  | rs62467127 | C | T | FALSE | 0.114 | -0.142 | 0.025 | 0.469 | 5.94E-06 | 0.762 | 20.507 |
|  | rs73952017 | C | T | FALSE | -0.086 | 0.180 | 0.019 | 0.244 | 9.16E-06 | 0.461 | 19.679 |
|  | rs78521377 | C | T | FALSE | 0.125 | -0.160 | 0.027 | 0.419 | 5.40E-06 | 0.703 | 20.689 |
|  | rs79150079 | C | A | FALSE | 0.122 | 0.250 | 0.027 | 0.295 | 7.27E-06 | 0.397 | 20.120 |
|  | rs892686 | A | G | FALSE | 0.051 | -0.009 | 0.011 | 0.149 | 3.90E-06 | 0.954 | 21.313 |
|  | rs999354 | T | A | TRUE | 0.058 | -0.156 | 0.012 | 0.154 | 7.36E-07 | 0.311 | 24.518 |
| Genus Clostridiumsensustricto1(id.1873) | rs11264403 | G | A | FALSE | -0.139 | -0.111 | 0.033 | 0.285 | 3.21E-05 | 0.697 | 17.288 |
|  | rs115807074 | A | G | FALSE | -0.227 | -0.584 | 0.049 | 0.704 | 3.92E-06 | 0.407 | 21.305 |
|  | rs11586026 | A | T | TRUE | 0.111 | -0.357 | 0.025 | 0.415 | 9.43E-06 | 0.389 | 19.624 |
|  | rs116847295 | C | T | FALSE | 0.110 | 0.082 | 0.025 | 0.226 | 7.75E-06 | 0.716 | 19.997 |
|  | rs12341505 | G | A | FALSE | 0.081 | -0.119 | 0.018 | 0.254 | 6.76E-06 | 0.640 | 20.259 |
|  | rs12490337 | C | G | TRUE | -0.062 | 0.047 | 0.014 | 0.185 | 7.33E-06 | 0.798 | 20.104 |
|  | rs2795528 | G | A | FALSE | -0.184 | -0.151 | 0.039 | 0.321 | 2.61E-06 | 0.638 | 22.085 |
|  | rs2817172 | C | T | FALSE | 0.058 | -0.238 | 0.012 | 0.152 | 3.01E-06 | 0.118 | 21.810 |
|  | rs550843 | T | C | FALSE | -0.078 | 0.122 | 0.017 | 0.168 | 3.68E-06 | 0.467 | 21.426 |
| Genus Collinsella(id.815) | rs10890671 | T | C | FALSE | -0.054 | 0.184 | 0.012 | 0.150 | 6.13E-06 | 0.220 | 20.446 |
|  | rs11597285 | G | T | FALSE | -0.054 | -0.046 | 0.012 | 0.152 | 8.10E-06 | 0.763 | 19.914 |
|  | rs12921100 | A | T | TRUE | 0.056 | 0.090 | 0.013 | 0.167 | 9.06E-06 | 0.591 | 19.699 |
|  | rs149807560 | C | A | FALSE | -0.104 | 0.454 | 0.024 | 0.294 | 9.83E-06 | 0.122 | 19.545 |
|  | rs2103510 | G | A | FALSE | 0.079 | 0.317 | 0.017 | 0.232 | 2.93E-06 | 0.173 | 21.863 |
|  | rs2671662 | C | G | TRUE | -0.057 | -0.136 | 0.012 | 0.150 | 2.00E-06 | 0.364 | 22.591 |
|  | rs59414781 | C | G | TRUE | 0.067 | -0.095 | 0.015 | 0.216 | 7.97E-06 | 0.659 | 19.944 |
|  | rs62102630 | A | G | FALSE | -0.070 | -0.259 | 0.015 | 0.217 | 5.59E-06 | 0.234 | 20.624 |
|  | rs62448869 | T | A | TRUE | -0.054 | -0.081 | 0.012 | 0.150 | 7.01E-06 | 0.588 | 20.190 |
|  | rs73052258 | G | A | FALSE | 0.093 | 0.268 | 0.020 | 0.281 | 4.43E-06 | 0.341 | 21.067 |
|  | rs75672793 | A | G | FALSE | -0.109 | -0.329 | 0.024 | 0.349 | 5.95E-06 | 0.347 | 20.503 |
|  | rs9541268 | C | A | FALSE | 0.096 | -0.037 | 0.020 | 0.258 | 1.15E-06 | 0.887 | 23.657 |
| Genus Coprobacter(id.949) | rs11532348 | C | T | FALSE | -0.104 | 0.314 | 0.023 | 0.207 | 4.61E-06 | 0.130 | 20.994 |
|  | rs12684609 | T | C | FALSE | 0.101 | -0.010 | 0.022 | 0.187 | 4.75E-06 | 0.956 | 20.934 |
|  | rs12908296 | A | G | FALSE | 0.101 | 0.126 | 0.022 | 0.196 | 6.58E-06 | 0.522 | 20.311 |
|  | rs12996055 | A | C | FALSE | 0.092 | -0.310 | 0.021 | 0.170 | 1.07E-05 | 0.068 | 19.378 |
|  | rs143662916 | C | T | FALSE | 0.253 | 0.678 | 0.054 | 0.436 | 2.75E-06 | 0.120 | 21.985 |
|  | rs189356 | G | A | FALSE | 0.078 | -0.018 | 0.017 | 0.150 | 5.53E-06 | 0.907 | 20.645 |
|  | rs213863 | C | T | FALSE | -0.089 | -0.083 | 0.019 | 0.155 | 2.47E-06 | 0.593 | 22.192 |
|  | rs305411 | A | G | FALSE | 0.129 | 0.118 | 0.026 | 0.245 | 1.03E-06 | 0.630 | 23.864 |
|  | rs3828477 | G | T | FALSE | -0.091 | -0.013 | 0.020 | 0.157 | 3.14E-06 | 0.936 | 21.728 |
|  | rs55672356 | T | A | TRUE | -0.193 | -0.358 | 0.041 | 0.418 | 2.98E-06 | 0.392 | 21.832 |
|  | rs72821405 | T | C | FALSE | -0.147 | 0.763 | 0.032 | 0.270 | 4.12E-06 | 0.005 | 21.210 |
|  | rs74919520 | G | A | FALSE | 0.126 | -0.197 | 0.028 | 0.241 | 5.38E-06 | 0.413 | 20.699 |
|  | rs76001613 | C | G | TRUE | 0.216 | 0.004 | 0.049 | 0.302 | 1.19E-05 | 0.991 | 19.179 |
| Genus Coprococcus1(id.11301) | rs1010560 | C | A | FALSE | 0.058 | 0.078 | 0.012 | 0.168 | 2.27E-06 | 0.644 | 22.354 |
|  | rs12794898 | G | T | FALSE | 0.090 | -0.139 | 0.020 | 0.226 | 4.64E-06 | 0.537 | 20.980 |
|  | rs12886051 | G | C | TRUE | -0.052 | -0.122 | 0.012 | 0.163 | 9.87E-06 | 0.454 | 19.536 |
|  | rs1519491 | T | C | FALSE | 0.050 | -0.175 | 0.011 | 0.152 | 1.10E-05 | 0.251 | 19.325 |
|  | rs1576241 | A | G | FALSE | -0.051 | 0.172 | 0.011 | 0.153 | 3.17E-06 | 0.260 | 21.708 |
|  | rs1762123 | C | T | FALSE | -0.089 | 0.256 | 0.020 | 0.248 | 7.12E-06 | 0.302 | 20.161 |
|  | rs2907920 | A | G | FALSE | 0.056 | 0.190 | 0.013 | 0.166 | 9.65E-06 | 0.251 | 19.579 |
|  | rs4277593 | G | A | FALSE | -0.059 | -0.225 | 0.011 | 0.151 | 9.92E-08 | 0.136 | 28.390 |
|  | rs56405618 | A | G | FALSE | -0.090 | -0.078 | 0.019 | 0.237 | 1.54E-06 | 0.742 | 23.095 |
|  | rs73031725 | T | C | FALSE | 0.168 | 0.024 | 0.036 | 0.423 | 2.38E-06 | 0.956 | 22.259 |
|  | rs73167075 | T | C | FALSE | 0.057 | 0.195 | 0.013 | 0.181 | 7.04E-06 | 0.281 | 20.184 |
|  | rs74101919 | T | C | FALSE | -0.072 | 0.155 | 0.014 | 0.237 | 6.68E-07 | 0.512 | 24.706 |
|  | rs7784490 | C | G | TRUE | -0.052 | -0.069 | 0.011 | 0.158 | 4.58E-06 | 0.662 | 21.007 |
|  | rs946513 | C | T | FALSE | 0.206 | -0.310 | 0.046 | 0.359 | 7.61E-06 | 0.387 | 20.034 |
| Genus Coprococcus2(id.11302) | rs12634070 | T | C | FALSE | 0.074 | 0.060 | 0.016 | 0.170 | 7.98E-06 | 0.725 | 19.942 |
|  | rs1958519 | T | A | TRUE | 0.067 | 0.211 | 0.014 | 0.150 | 1.57E-06 | 0.159 | 23.055 |
|  | rs2482516 | C | T | FALSE | 0.075 | 0.095 | 0.016 | 0.179 | 4.59E-06 | 0.596 | 21.002 |
|  | rs35890118 | A | G | FALSE | -0.067 | -0.091 | 0.015 | 0.171 | 6.61E-06 | 0.593 | 20.304 |
|  | rs59936925 | A | T | TRUE | 0.117 | 0.079 | 0.023 | 0.240 | 5.62E-07 | 0.740 | 25.038 |
|  | rs61823518 | A | C | FALSE | -0.096 | 0.326 | 0.022 | 0.243 | 9.48E-06 | 0.180 | 19.613 |
|  | rs6677933 | C | T | FALSE | -0.080 | -0.358 | 0.016 | 0.211 | 9.66E-07 | 0.089 | 23.995 |
|  | rs6894272 | T | C | FALSE | -0.113 | -0.182 | 0.025 | 0.230 | 7.31E-06 | 0.428 | 20.110 |
|  | rs72680320 | T | C | FALSE | -0.065 | -0.154 | 0.014 | 0.157 | 3.08E-06 | 0.326 | 21.766 |
|  | rs9426473 | A | G | FALSE | 0.073 | 0.113 | 0.016 | 0.170 | 6.82E-06 | 0.506 | 20.244 |
| Genus Coprococcus3(id.11303) | rs10810043 | A | G | FALSE | 0.052 | -0.425 | 0.012 | 0.159 | 8.70E-06 | 0.007 | 19.776 |
|  | rs11077359 | T | C | FALSE | -0.065 | -0.283 | 0.015 | 0.200 | 1.45E-05 | 0.156 | 18.807 |
|  | rs11080344 | C | T | FALSE | 0.052 | -0.131 | 0.011 | 0.149 | 4.82E-06 | 0.381 | 20.906 |
|  | rs13247359 | G | A | FALSE | 0.051 | 0.069 | 0.011 | 0.150 | 5.88E-06 | 0.645 | 20.527 |
|  | rs13394391 | C | T | FALSE | -0.071 | -0.075 | 0.015 | 0.204 | 2.60E-06 | 0.715 | 22.089 |
|  | rs178271 | T | C | FALSE | 0.145 | -0.095 | 0.029 | 0.564 | 7.95E-07 | 0.866 | 24.371 |
|  | rs4575475 | G | A | FALSE | 0.062 | 0.302 | 0.014 | 0.178 | 6.92E-06 | 0.090 | 20.215 |
|  | rs62481985 | G | C | TRUE | -0.058 | -0.106 | 0.012 | 0.151 | 4.15E-07 | 0.482 | 25.623 |
|  | rs7521171 | G | A | FALSE | -0.060 | 0.047 | 0.013 | 0.162 | 3.96E-06 | 0.772 | 21.285 |
|  | rs8100692 | T | C | FALSE | 0.058 | -0.197 | 0.011 | 0.149 | 3.61E-07 | 0.187 | 25.892 |
| Genus DefluviitaleaceaeUCG011(id.11287) | rs112893842 | T | C | FALSE | 0.114 | 0.331 | 0.023 | 0.257 | 1.02E-06 | 0.197 | 23.899 |
|  | rs12500663 | T | G | FALSE | -0.115 | -0.199 | 0.023 | 0.286 | 4.72E-07 | 0.485 | 25.374 |
|  | rs1582238 | T | C | FALSE | 0.081 | -0.113 | 0.017 | 0.155 | 1.49E-06 | 0.468 | 23.167 |
|  | rs28696126 | A | T | TRUE | -0.107 | -0.005 | 0.024 | 0.223 | 7.97E-06 | 0.982 | 19.945 |
|  | rs2892880 | G | A | FALSE | 0.082 | 0.049 | 0.018 | 0.172 | 6.77E-06 | 0.778 | 20.258 |
|  | rs4344384 | G | T | FALSE | 0.072 | -0.229 | 0.016 | 0.149 | 4.64E-06 | 0.125 | 20.981 |
|  | rs4677103 | A | G | FALSE | 0.098 | -0.097 | 0.020 | 0.197 | 7.14E-07 | 0.621 | 24.576 |
|  | rs55658617 | T | C | FALSE | 0.174 | -0.424 | 0.036 | 0.400 | 1.48E-06 | 0.290 | 23.173 |
|  | rs9608282 | T | G | FALSE | 0.143 | 0.184 | 0.030 | 0.414 | 1.86E-06 | 0.656 | 22.733 |
|  | rs9725395 | A | G | FALSE | -0.138 | 0.028 | 0.030 | 0.235 | 2.86E-06 | 0.905 | 21.911 |
| Genus Desulfovibrio(id.3173) | rs11160353 | T | A | TRUE | -0.069 | 0.123 | 0.015 | 0.151 | 2.64E-06 | 0.416 | 22.059 |
|  | rs12031543 | T | C | FALSE | -0.127 | 0.207 | 0.028 | 0.218 | 6.42E-06 | 0.344 | 20.357 |
|  | rs13066142 | G | A | FALSE | 0.119 | 0.140 | 0.025 | 0.256 | 2.05E-06 | 0.585 | 22.550 |
|  | rs16863365 | A | G | FALSE | 0.109 | -0.093 | 0.023 | 0.348 | 1.43E-06 | 0.790 | 23.235 |
|  | rs2032031 | A | G | FALSE | -0.065 | 0.184 | 0.015 | 0.149 | 1.06E-05 | 0.217 | 19.404 |
|  | rs2590913 | G | A | FALSE | 0.154 | -0.089 | 0.034 | 0.338 | 5.31E-06 | 0.793 | 20.721 |
|  | rs2853179 | C | T | FALSE | 0.081 | -0.226 | 0.017 | 0.178 | 3.16E-06 | 0.205 | 21.718 |
|  | rs4797774 | G | A | FALSE | 0.213 | -0.217 | 0.047 | 0.376 | 6.11E-06 | 0.565 | 20.454 |
|  | rs6580353 | T | C | FALSE | 0.077 | 0.112 | 0.017 | 0.187 | 5.57E-06 | 0.550 | 20.632 |
|  | rs72647089 | T | G | FALSE | -0.107 | 0.035 | 0.024 | 0.274 | 8.44E-06 | 0.899 | 19.836 |
|  | rs7729080 | C | A | FALSE | -0.070 | -0.036 | 0.016 | 0.163 | 8.30E-06 | 0.828 | 19.867 |
| Genus Dialister(id.2183) | rs10138457 | T | C | FALSE | -0.113 | -0.434 | 0.026 | 0.258 | 1.59E-05 | 0.093 | 18.629 |
|  | rs10938938 | G | A | FALSE | -0.077 | 0.322 | 0.017 | 0.202 | 6.02E-06 | 0.112 | 20.482 |
|  | rs11071887 | T | C | FALSE | 0.066 | -0.089 | 0.015 | 0.160 | 5.98E-06 | 0.578 | 20.496 |
|  | rs11166701 | G | A | FALSE | -0.066 | -0.036 | 0.013 | 0.149 | 6.71E-07 | 0.810 | 24.698 |
|  | rs2314294 | T | C | FALSE | 0.087 | 0.038 | 0.019 | 0.218 | 7.82E-06 | 0.863 | 19.981 |
|  | rs2435610 | A | C | FALSE | 0.065 | 0.067 | 0.014 | 0.171 | 6.32E-06 | 0.694 | 20.389 |
|  | rs4747450 | C | A | FALSE | 0.067 | 0.033 | 0.015 | 0.178 | 5.99E-06 | 0.855 | 20.490 |
|  | rs4753063 | G | A | FALSE | -0.060 | -0.187 | 0.013 | 0.149 | 4.54E-06 | 0.210 | 21.023 |
|  | rs517089 | T | A | TRUE | 0.076 | 0.346 | 0.017 | 0.197 | 7.71E-06 | 0.079 | 20.009 |
|  | rs75416973 | A | G | FALSE | 0.073 | -0.133 | 0.016 | 0.180 | 9.85E-06 | 0.461 | 19.541 |
|  | rs764177 | C | A | FALSE | -0.060 | -0.083 | 0.014 | 0.155 | 8.86E-06 | 0.592 | 19.742 |
|  | rs76680460 | G | A | FALSE | -0.161 | -0.193 | 0.036 | 0.370 | 9.55E-06 | 0.603 | 19.600 |
| Genus Dorea(id.1997) | rs11150408 | T | G | FALSE | 0.049 | 0.088 | 0.011 | 0.150 | 7.58E-06 | 0.557 | 20.042 |
|  | rs12216169 | T | A | TRUE | 0.088 | 0.234 | 0.019 | 0.232 | 5.30E-06 | 0.314 | 20.724 |
|  | rs12537781 | T | C | FALSE | -0.056 | 0.064 | 0.013 | 0.173 | 9.18E-06 | 0.713 | 19.675 |
|  | rs13279148 | G | A | FALSE | 0.072 | 0.428 | 0.015 | 0.235 | 2.13E-06 | 0.068 | 22.477 |
|  | rs1899291 | C | T | FALSE | 0.070 | 0.083 | 0.015 | 0.206 | 3.50E-06 | 0.686 | 21.523 |
|  | rs3005511 | A | G | FALSE | 0.052 | -0.050 | 0.011 | 0.162 | 4.80E-06 | 0.759 | 20.917 |
|  | rs345219 | T | G | FALSE | -0.050 | -0.094 | 0.011 | 0.151 | 1.00E-05 | 0.533 | 19.504 |
|  | rs3752849 | G | A | FALSE | 0.164 | -0.116 | 0.037 | 0.343 | 7.62E-06 | 0.736 | 20.032 |
|  | rs4793307 | C | T | FALSE | 0.057 | 0.160 | 0.012 | 0.175 | 2.76E-06 | 0.360 | 21.979 |
|  | rs62503162 | A | G | FALSE | -0.097 | -0.004 | 0.019 | 0.364 | 5.41E-07 | 0.992 | 25.113 |
|  | rs62583469 | T | A | TRUE | -0.063 | 0.045 | 0.014 | 0.216 | 8.25E-06 | 0.835 | 19.879 |
|  | rs73729431 | C | T | FALSE | -0.137 | -0.210 | 0.030 | 0.526 | 4.61E-06 | 0.691 | 20.994 |
| Genus Eggerthella(id.819) | rs112205261 | T | C | FALSE | -0.189 | -0.192 | 0.040 | 0.275 | 2.96E-06 | 0.486 | 21.839 |
|  | rs13070736 | A | C | FALSE | -0.121 | 0.056 | 0.027 | 0.202 | 8.18E-06 | 0.783 | 19.894 |
|  | rs1784405 | T | G | FALSE | 0.091 | -0.081 | 0.020 | 0.148 | 4.58E-06 | 0.583 | 21.004 |
|  | rs2223081 | G | A | FALSE | 0.103 | -0.150 | 0.022 | 0.166 | 3.50E-06 | 0.367 | 21.520 |
|  | rs2240838 | A | G | FALSE | 0.098 | -0.016 | 0.020 | 0.151 | 7.01E-07 | 0.915 | 24.613 |
|  | rs3851328 | T | G | FALSE | -0.108 | 0.240 | 0.024 | 0.180 | 5.20E-06 | 0.181 | 20.762 |
|  | rs4985746 | G | A | FALSE | 0.111 | 0.230 | 0.025 | 0.241 | 8.09E-06 | 0.339 | 19.917 |
|  | rs6430926 | C | T | FALSE | 0.088 | -0.206 | 0.020 | 0.150 | 8.09E-06 | 0.169 | 19.916 |
|  | rs67490567 | T | C | FALSE | 0.108 | 0.013 | 0.025 | 0.172 | 9.87E-06 | 0.939 | 19.537 |
|  | rs76663501 | C | T | FALSE | 0.175 | -0.146 | 0.038 | 0.339 | 3.62E-06 | 0.668 | 21.454 |
| Genus Eisenbergiella(id.11304) | rs11027642 | C | T | FALSE | 0.129 | 0.292 | 0.028 | 0.211 | 5.93E-06 | 0.166 | 20.512 |
|  | rs11651545 | T | C | FALSE | 0.107 | -0.036 | 0.024 | 0.184 | 6.85E-06 | 0.847 | 20.235 |
|  | rs11938607 | T | C | FALSE | 0.098 | 0.226 | 0.022 | 0.172 | 6.33E-06 | 0.188 | 20.386 |
|  | rs12257723 | A | C | FALSE | -0.095 | 0.177 | 0.021 | 0.158 | 6.69E-06 | 0.265 | 20.279 |
|  | rs12278566 | T | A | TRUE | -0.121 | -0.064 | 0.025 | 0.170 | 1.50E-06 | 0.704 | 23.143 |
|  | rs12710729 | C | A | FALSE | 0.089 | 0.089 | 0.020 | 0.159 | 7.20E-06 | 0.575 | 20.140 |
|  | rs13258851 | A | G | FALSE | 0.137 | 0.248 | 0.030 | 0.214 | 5.77E-06 | 0.248 | 20.563 |
|  | rs1508033 | A | C | FALSE | 0.092 | -0.235 | 0.020 | 0.162 | 2.93E-06 | 0.148 | 21.863 |
|  | rs1553971 | T | G | FALSE | 0.121 | -0.242 | 0.026 | 0.180 | 4.26E-06 | 0.178 | 21.146 |
|  | rs2683098 | C | T | FALSE | 0.107 | -0.012 | 0.023 | 0.181 | 1.87E-06 | 0.947 | 22.720 |
|  | rs3812426 | G | A | FALSE | 0.106 | 0.290 | 0.022 | 0.207 | 2.05E-06 | 0.160 | 22.551 |
|  | rs4462860 | G | A | FALSE | 0.094 | 0.047 | 0.020 | 0.153 | 3.02E-06 | 0.759 | 21.805 |
| Genus Enterorhabdus(id.820) | rs10098492 | T | C | FALSE | 0.132 | -0.177 | 0.029 | 0.314 | 6.62E-06 | 0.574 | 20.300 |
|  | rs11098863 | T | A | TRUE | -0.097 | 0.150 | 0.016 | 0.149 | 2.88E-09 | 0.314 | 35.262 |
|  | rs114731706 | T | G | FALSE | 0.182 | -0.881 | 0.038 | 0.412 | 1.85E-06 | 0.033 | 22.741 |
|  | rs2051957 | C | T | FALSE | 0.084 | -0.376 | 0.019 | 0.188 | 8.93E-06 | 0.046 | 19.727 |
|  | rs3017103 | A | G | FALSE | 0.098 | 0.155 | 0.021 | 0.189 | 2.69E-06 | 0.413 | 22.028 |
|  | rs73331712 | T | C | FALSE | 0.262 | -0.226 | 0.055 | 0.370 | 2.01E-06 | 0.542 | 22.589 |
|  | rs77655283 | G | A | FALSE | 0.133 | -0.119 | 0.030 | 0.295 | 8.27E-06 | 0.686 | 19.875 |
|  | rs7923280 | A | T | TRUE | 0.086 | -0.039 | 0.017 | 0.165 | 3.69E-07 | 0.812 | 25.851 |
|  | rs9470637 | A | T | TRUE | -0.076 | -0.056 | 0.017 | 0.149 | 5.52E-06 | 0.707 | 20.649 |
| Genus Erysipelatoclostridium(id.11381) | rs1434153 | G | A | FALSE | -0.068 | -0.080 | 0.015 | 0.150 | 7.11E-06 | 0.596 | 20.163 |
|  | rs16936671 | C | T | FALSE | -0.097 | -0.308 | 0.022 | 0.218 | 8.88E-06 | 0.158 | 19.739 |
|  | rs17804233 | T | C | FALSE | -0.066 | -0.173 | 0.014 | 0.149 | 4.28E-06 | 0.247 | 21.133 |
|  | rs2901723 | C | A | FALSE | 0.064 | -0.277 | 0.014 | 0.150 | 8.80E-06 | 0.065 | 19.755 |
|  | rs340991 | A | G | FALSE | -0.074 | 0.218 | 0.016 | 0.168 | 3.16E-06 | 0.194 | 21.718 |
|  | rs34528142 | C | G | TRUE | -0.088 | -0.308 | 0.020 | 0.186 | 7.56E-06 | 0.097 | 20.046 |
|  | rs3804326 | A | G | FALSE | 0.141 | 0.379 | 0.034 | 0.348 | 2.60E-05 | 0.277 | 17.691 |
|  | rs45480394 | T | G | FALSE | -0.069 | 0.065 | 0.015 | 0.155 | 6.02E-06 | 0.675 | 20.480 |
|  | rs4697572 | A | G | FALSE | -0.081 | 0.339 | 0.016 | 0.187 | 6.91E-07 | 0.070 | 24.640 |
|  | rs58236560 | G | T | FALSE | -0.111 | 0.342 | 0.023 | 0.227 | 2.12E-06 | 0.133 | 22.483 |
|  | rs61806970 | C | T | FALSE | 0.143 | -0.180 | 0.032 | 0.297 | 8.79E-06 | 0.545 | 19.759 |
|  | rs622418 | A | G | FALSE | -0.067 | -0.159 | 0.014 | 0.149 | 3.10E-06 | 0.285 | 21.756 |
|  | rs6474512 | A | C | FALSE | 0.067 | -0.187 | 0.014 | 0.153 | 2.87E-06 | 0.221 | 21.900 |
|  | rs710230 | T | C | FALSE | 0.143 | 0.053 | 0.028 | 0.292 | 3.52E-07 | 0.856 | 25.941 |
|  | rs7221249 | A | G | FALSE | 0.084 | -0.063 | 0.014 | 0.149 | 4.01E-09 | 0.672 | 34.619 |
|  | rs9590927 | G | A | FALSE | -0.065 | 0.290 | 0.014 | 0.149 | 6.72E-06 | 0.052 | 20.272 |
| Genus ErysipelotrichaceaeUCG003(id.11384) | rs11666127 | A | G | FALSE | -0.072 | 0.128 | 0.016 | 0.198 | 8.32E-06 | 0.517 | 19.863 |
|  | rs11877693 | C | G | TRUE | -0.095 | 0.410 | 0.019 | 0.219 | 5.39E-07 | 0.061 | 25.120 |
|  | rs11994308 | C | T | FALSE | 0.115 | -0.061 | 0.024 | 0.263 | 1.94E-06 | 0.817 | 22.652 |
|  | rs12251396 | A | G | FALSE | -0.071 | 0.058 | 0.016 | 0.207 | 8.87E-06 | 0.780 | 19.740 |
|  | rs17798136 | G | A | FALSE | 0.159 | 0.477 | 0.035 | 0.305 | 4.92E-06 | 0.117 | 20.867 |
|  | rs2044550 | T | C | FALSE | -0.061 | 0.079 | 0.014 | 0.184 | 7.50E-06 | 0.667 | 20.062 |
|  | rs28568391 | A | G | FALSE | -0.058 | -0.057 | 0.012 | 0.150 | 8.76E-07 | 0.703 | 24.182 |
|  | rs4758231 | G | T | FALSE | -0.055 | 0.247 | 0.012 | 0.165 | 6.16E-06 | 0.136 | 20.438 |
|  | rs59068084 | T | G | FALSE | 0.056 | 0.066 | 0.012 | 0.152 | 2.63E-06 | 0.667 | 22.068 |
|  | rs62403464 | T | C | FALSE | -0.073 | -0.198 | 0.016 | 0.194 | 2.94E-06 | 0.308 | 21.858 |
|  | rs6875357 | C | T | FALSE | 0.166 | -0.160 | 0.035 | 0.385 | 2.84E-06 | 0.677 | 21.924 |
|  | rs73074432 | C | T | FALSE | 0.072 | -0.403 | 0.016 | 0.235 | 1.14E-05 | 0.087 | 19.268 |
|  | rs74988980 | G | A | FALSE | -0.133 | 0.142 | 0.035 | 0.368 | 0.000133669 | 0.700 | 14.589 |
|  | rs75949021 | T | C | FALSE | -0.170 | -0.737 | 0.037 | 0.372 | 5.80E-06 | 0.048 | 20.554 |
|  | rs76502207 | T | C | FALSE | 0.145 | -0.343 | 0.029 | 0.378 | 5.86E-07 | 0.364 | 24.958 |
|  | rs79396538 | C | G | TRUE | 0.085 | 0.307 | 0.019 | 0.314 | 9.12E-06 | 0.327 | 19.688 |
|  | rs8053479 | A | G | FALSE | -0.084 | -0.205 | 0.019 | 0.237 | 6.98E-06 | 0.387 | 20.198 |
| Genus Escherichia.Shigella(id.3504) | rs112767262 | T | C | FALSE | 0.073 | 0.332 | 0.016 | 0.181 | 7.45E-06 | 0.066 | 20.075 |
|  | rs113127095 | A | G | FALSE | 0.151 | 0.409 | 0.032 | 0.376 | 3.03E-06 | 0.277 | 21.799 |
|  | rs113513883 | A | G | FALSE | 0.172 | -0.415 | 0.038 | 0.415 | 5.93E-06 | 0.317 | 20.511 |
|  | rs1154904 | A | G | FALSE | -0.061 | -0.032 | 0.013 | 0.148 | 2.67E-06 | 0.827 | 22.043 |
|  | rs11706043 | T | A | TRUE | 0.076 | -0.502 | 0.016 | 0.182 | 4.05E-06 | 0.006 | 21.242 |
|  | rs117092367 | A | T | TRUE | 0.117 | -0.243 | 0.026 | 0.299 | 9.14E-06 | 0.416 | 19.683 |
|  | rs118526 | C | A | FALSE | -0.059 | -0.083 | 0.014 | 0.162 | 1.24E-05 | 0.609 | 19.108 |
|  | rs2267739 | G | C | TRUE | 0.116 | 0.097 | 0.024 | 0.237 | 1.17E-06 | 0.683 | 23.627 |
|  | rs2798105 | A | G | FALSE | -0.101 | -0.200 | 0.022 | 0.248 | 5.57E-06 | 0.420 | 20.629 |
|  | rs35555519 | C | G | TRUE | 0.102 | 0.245 | 0.022 | 0.249 | 4.76E-06 | 0.326 | 20.933 |
|  | rs4731451 | G | A | FALSE | -0.061 | -0.076 | 0.014 | 0.160 | 6.41E-06 | 0.635 | 20.362 |
|  | rs57024273 | T | C | FALSE | 0.063 | -0.055 | 0.014 | 0.170 | 7.63E-06 | 0.746 | 20.028 |
|  | rs592299 | T | C | FALSE | -0.059 | 0.082 | 0.013 | 0.149 | 4.83E-06 | 0.583 | 20.903 |
|  | rs73208162 | A | G | FALSE | -0.119 | -0.532 | 0.025 | 0.415 | 1.56E-06 | 0.200 | 23.066 |
|  | rs7502686 | G | C | TRUE | -0.136 | -0.150 | 0.030 | 0.309 | 6.56E-06 | 0.627 | 20.317 |
| Genus Faecalibacterium(id.2057) | rs10927394 | G | T | FALSE | -0.232 | -0.451 | 0.051 | 0.538 | 5.81E-06 | 0.402 | 20.549 |
|  | rs114946999 | C | T | FALSE | -0.086 | -0.152 | 0.019 | 0.224 | 5.52E-06 | 0.499 | 20.649 |
|  | rs11776390 | T | C | FALSE | -0.078 | 0.191 | 0.017 | 0.302 | 5.12E-06 | 0.527 | 20.793 |
|  | rs12320842 | C | G | TRUE | 0.095 | -0.018 | 0.016 | 0.217 | 7.30E-09 | 0.933 | 33.453 |
|  | rs1271565 | C | T | FALSE | -0.058 | 0.217 | 0.012 | 0.170 | 1.46E-06 | 0.200 | 23.195 |
|  | rs12753492 | A | C | FALSE | 0.064 | -0.271 | 0.015 | 0.235 | 1.89E-05 | 0.248 | 18.295 |
|  | rs2835874 | T | C | FALSE | -0.087 | 0.018 | 0.020 | 0.390 | 1.04E-05 | 0.963 | 19.440 |
|  | rs28376661 | C | G | TRUE | 0.050 | -0.100 | 0.011 | 0.162 | 3.92E-06 | 0.538 | 21.305 |
|  | rs61875484 | C | G | TRUE | 0.082 | -0.434 | 0.018 | 0.255 | 8.47E-06 | 0.089 | 19.828 |
|  | rs6910935 | A | G | FALSE | 0.135 | -0.492 | 0.028 | 0.310 | 1.13E-06 | 0.112 | 23.699 |
|  | rs75499067 | C | T | FALSE | 0.228 | -0.340 | 0.047 | 0.284 | 1.01E-06 | 0.231 | 23.900 |
|  | rs79656633 | T | C | FALSE | 0.146 | -0.582 | 0.032 | 0.249 | 6.53E-06 | 0.019 | 20.326 |
|  | rs9536330 | T | C | FALSE | -0.048 | 0.092 | 0.011 | 0.149 | 7.64E-06 | 0.537 | 20.026 |
| Genus FamilyXIIIAD3011group(id.11293) | rs11126423 | C | T | FALSE | 0.090 | -0.213 | 0.020 | 0.259 | 4.08E-06 | 0.412 | 21.226 |
|  | rs11736617 | G | A | FALSE | -0.076 | -0.021 | 0.017 | 0.321 | 1.02E-05 | 0.949 | 19.475 |
|  | rs12314465 | A | G | FALSE | -0.092 | 0.467 | 0.020 | 0.267 | 3.10E-06 | 0.080 | 21.752 |
|  | rs12911842 | A | T | TRUE | -0.081 | -0.064 | 0.018 | 0.263 | 9.47E-06 | 0.808 | 19.615 |
|  | rs149302 | T | C | FALSE | -0.065 | 0.222 | 0.014 | 0.176 | 6.55E-06 | 0.206 | 20.321 |
|  | rs16840310 | A | G | FALSE | -0.061 | -0.097 | 0.012 | 0.151 | 6.43E-07 | 0.521 | 24.780 |
|  | rs16940167 | C | T | FALSE | 0.073 | 0.203 | 0.016 | 0.190 | 4.62E-06 | 0.285 | 20.989 |
|  | rs17156849 | G | A | FALSE | -0.113 | 0.447 | 0.025 | 0.314 | 4.18E-06 | 0.155 | 21.181 |
|  | rs62029761 | A | G | FALSE | 0.129 | 0.060 | 0.028 | 0.334 | 3.09E-06 | 0.857 | 21.762 |
|  | rs62200412 | C | T | FALSE | -0.080 | -0.251 | 0.016 | 0.173 | 1.02E-06 | 0.148 | 23.894 |
|  | rs72730932 | C | A | FALSE | -0.090 | 0.170 | 0.018 | 0.254 | 3.79E-07 | 0.503 | 25.798 |
|  | rs739451 | C | T | FALSE | 0.065 | 0.189 | 0.015 | 0.184 | 1.07E-05 | 0.305 | 19.386 |
|  | rs9276029 | A | G | FALSE | -0.081 | -0.003 | 0.019 | 0.203 | 1.24E-05 | 0.989 | 19.097 |
|  | rs9837139 | A | G | FALSE | 0.108 | -0.309 | 0.024 | 0.269 | 7.78E-06 | 0.251 | 19.990 |
|  | rs9852893 | C | G | TRUE | 0.066 | -0.228 | 0.013 | 0.168 | 3.45E-07 | 0.175 | 25.982 |
| Genus FamilyXIIIUCG001(id.11294) | rs112362903 | A | G | FALSE | -0.149 | 0.222 | 0.033 | 0.397 | 7.74E-06 | 0.577 | 20.001 |
|  | rs116979587 | T | A | TRUE | -0.122 | 0.227 | 0.026 | 0.347 | 3.08E-06 | 0.512 | 21.764 |
|  | rs12049454 | T | C | FALSE | -0.065 | -0.023 | 0.013 | 0.153 | 1.38E-06 | 0.879 | 23.312 |
|  | rs1426266 | T | C | FALSE | -0.067 | -0.276 | 0.014 | 0.167 | 1.22E-06 | 0.098 | 23.547 |
|  | rs2276529 | C | G | TRUE | -0.076 | -0.252 | 0.017 | 0.186 | 4.23E-06 | 0.175 | 21.157 |
|  | rs3842897 | G | A | FALSE | -0.113 | -0.339 | 0.024 | 0.264 | 3.48E-06 | 0.200 | 21.531 |
|  | rs62414802 | C | T | FALSE | -0.061 | -0.009 | 0.013 | 0.172 | 5.44E-06 | 0.959 | 20.676 |
|  | rs7119679 | G | A | FALSE | -0.081 | 0.072 | 0.017 | 0.178 | 3.67E-06 | 0.686 | 21.429 |
|  | rs76463770 | A | G | FALSE | 0.193 | 0.262 | 0.042 | 0.432 | 4.23E-06 | 0.544 | 21.158 |
|  | rs8076666 | A | G | FALSE | 0.089 | -0.155 | 0.020 | 0.228 | 7.63E-06 | 0.496 | 20.029 |
| Genus Flavonifractor(id.2059) | rs114873521 | C | T | FALSE | -0.130 | 0.253 | 0.029 | 0.290 | 9.75E-06 | 0.383 | 19.561 |
|  | rs11642826 | G | C | TRUE | 0.147 | 0.107 | 0.033 | 0.260 | 6.30E-06 | 0.681 | 20.395 |
|  | rs11811696 | T | C | FALSE | -0.116 | 0.233 | 0.024 | 0.272 | 1.47E-06 | 0.391 | 23.190 |
|  | rs12030302 | A | G | FALSE | -0.069 | 0.112 | 0.014 | 0.149 | 4.76E-07 | 0.451 | 25.361 |
|  | rs12038887 | C | G | TRUE | 0.094 | -0.135 | 0.021 | 0.222 | 8.42E-06 | 0.542 | 19.840 |
|  | rs34066017 | A | G | FALSE | 0.076 | 0.196 | 0.016 | 0.184 | 1.73E-06 | 0.288 | 22.878 |
|  | rs6761463 | G | C | TRUE | -0.083 | 0.251 | 0.018 | 0.217 | 6.26E-06 | 0.248 | 20.408 |
|  | rs806808 | T | C | FALSE | 0.067 | -0.029 | 0.014 | 0.150 | 1.03E-06 | 0.845 | 23.877 |
| Genus Fusicatenibacter(id.11305) | rs10439674 | A | G | FALSE | -0.057 | -0.129 | 0.013 | 0.185 | 1.08E-05 | 0.483 | 19.367 |
|  | rs167879 | C | T | FALSE | -0.066 | -0.003 | 0.015 | 0.209 | 9.27E-06 | 0.988 | 19.656 |
|  | rs16866708 | G | A | FALSE | -0.070 | -0.074 | 0.016 | 0.188 | 6.34E-06 | 0.695 | 20.382 |
|  | rs1864685 | A | C | FALSE | -0.049 | -0.015 | 0.011 | 0.151 | 4.72E-06 | 0.921 | 20.949 |
|  | rs2025938 | G | A | FALSE | -0.097 | -0.211 | 0.021 | 0.297 | 2.49E-06 | 0.479 | 22.172 |
|  | rs2039204 | T | A | TRUE | -0.050 | 0.164 | 0.011 | 0.155 | 4.06E-06 | 0.290 | 21.235 |
|  | rs206581 | A | G | FALSE | -0.057 | -0.284 | 0.013 | 0.181 | 8.84E-06 | 0.116 | 19.748 |
|  | rs2132128 | G | A | FALSE | -0.077 | 0.280 | 0.016 | 0.248 | 1.48E-06 | 0.260 | 23.176 |
|  | rs3303 | T | C | FALSE | -0.095 | -0.083 | 0.020 | 0.305 | 2.97E-06 | 0.785 | 21.839 |
|  | rs4378146 | A | C | FALSE | -0.062 | -0.075 | 0.013 | 0.170 | 8.51E-07 | 0.661 | 24.239 |
|  | rs60254196 | A | G | FALSE | -0.049 | -0.170 | 0.011 | 0.150 | 6.71E-06 | 0.258 | 20.273 |
|  | rs62353480 | A | G | FALSE | -0.070 | -0.066 | 0.015 | 0.199 | 1.45E-06 | 0.741 | 23.210 |
|  | rs6515626 | G | A | FALSE | 0.142 | 0.003 | 0.031 | 0.300 | 6.33E-06 | 0.992 | 20.386 |
|  | rs704418 | T | C | FALSE | 0.074 | -0.003 | 0.015 | 0.226 | 9.96E-07 | 0.990 | 23.937 |
|  | rs73103914 | A | G | FALSE | -0.060 | 0.029 | 0.013 | 0.208 | 8.89E-06 | 0.889 | 19.736 |
|  | rs792108 | T | C | FALSE | -0.051 | -0.142 | 0.011 | 0.151 | 8.00E-06 | 0.346 | 19.939 |
|  | rs8028026 | A | G | FALSE | -0.079 | -0.253 | 0.018 | 0.257 | 1.14E-05 | 0.326 | 19.259 |
|  | rs8063430 | T | C | FALSE | -0.104 | -0.062 | 0.022 | 0.337 | 2.84E-06 | 0.855 | 21.924 |
|  | rs9905659 | G | A | FALSE | -0.062 | 0.005 | 0.014 | 0.192 | 6.44E-06 | 0.979 | 20.354 |
| Genus Gordonibacter(id.821) | rs117347059 | G | C | TRUE | -0.128 | -0.226 | 0.029 | 0.191 | 6.85E-06 | 0.236 | 20.236 |
|  | rs13412653 | A | C | FALSE | 0.108 | 0.166 | 0.024 | 0.154 | 6.87E-06 | 0.281 | 20.229 |
|  | rs16955299 | G | A | FALSE | -0.196 | 0.351 | 0.043 | 0.252 | 5.88E-06 | 0.163 | 20.527 |
|  | rs322296 | G | A | FALSE | 0.179 | -0.368 | 0.038 | 0.281 | 2.17E-06 | 0.192 | 22.439 |
|  | rs35042269 | C | A | FALSE | -0.180 | -0.067 | 0.040 | 0.233 | 7.80E-06 | 0.774 | 19.985 |
|  | rs3765837 | T | G | FALSE | -0.191 | 0.004 | 0.043 | 0.289 | 1.09E-05 | 0.989 | 19.348 |
|  | rs4596722 | A | G | FALSE | 0.103 | 0.001 | 0.023 | 0.149 | 8.83E-06 | 0.996 | 19.748 |
|  | rs61934597 | C | T | FALSE | -0.172 | 0.320 | 0.039 | 0.304 | 9.03E-06 | 0.294 | 19.707 |
|  | rs71545975 | A | G | FALSE | -0.154 | -0.161 | 0.034 | 0.198 | 5.54E-06 | 0.415 | 20.639 |
|  | rs7220558 | A | T | TRUE | 0.117 | 0.085 | 0.023 | 0.151 | 6.45E-07 | 0.575 | 24.774 |
|  | rs72714787 | C | A | FALSE | 0.181 | -0.319 | 0.038 | 0.222 | 1.51E-06 | 0.150 | 23.141 |
|  | rs72939513 | A | G | FALSE | -0.214 | 0.075 | 0.049 | 0.338 | 1.29E-05 | 0.824 | 19.026 |
|  | rs7294633 | C | T | FALSE | 0.129 | 0.246 | 0.025 | 0.167 | 2.63E-07 | 0.140 | 26.501 |
|  | rs76287110 | A | T | TRUE | -0.243 | -0.104 | 0.047 | 0.273 | 1.87E-07 | 0.704 | 27.163 |
|  | rs768830 | G | A | FALSE | 0.150 | 0.015 | 0.033 | 0.205 | 6.93E-06 | 0.941 | 20.212 |
| Genus Haemophilus(id.3698) | rs10781340 | G | A | FALSE | 0.095 | 0.671 | 0.020 | 0.229 | 3.02E-06 | 0.003 | 21.803 |
|  | rs10840326 | C | G | TRUE | -0.068 | 0.092 | 0.015 | 0.155 | 7.74E-06 | 0.552 | 20.001 |
|  | rs111582866 | G | A | FALSE | -0.124 | -0.056 | 0.026 | 0.265 | 1.78E-06 | 0.832 | 22.815 |
|  | rs12191680 | C | G | TRUE | 0.107 | 0.096 | 0.020 | 0.245 | 1.05E-07 | 0.695 | 28.288 |
|  | rs12876183 | T | A | TRUE | 0.075 | 0.095 | 0.017 | 0.157 | 7.35E-06 | 0.543 | 20.100 |
|  | rs35509 | G | A | FALSE | 0.128 | 0.142 | 0.027 | 0.366 | 1.83E-06 | 0.698 | 22.768 |
|  | rs4822728 | T | C | FALSE | 0.071 | -0.224 | 0.015 | 0.149 | 3.12E-06 | 0.132 | 21.740 |
|  | rs56310940 | G | C | TRUE | -0.108 | -0.113 | 0.025 | 0.225 | 1.15E-05 | 0.616 | 19.250 |
|  | rs76022354 | C | T | FALSE | 0.245 | -0.247 | 0.051 | 0.341 | 1.30E-06 | 0.469 | 23.421 |
|  | rs78909003 | T | C | FALSE | -0.246 | -0.208 | 0.050 | 0.328 | 1.03E-06 | 0.525 | 23.880 |
|  | rs9328464 | T | C | FALSE | 0.072 | -0.213 | 0.015 | 0.149 | 1.18E-06 | 0.154 | 23.615 |
|  | rs9382510 | C | T | FALSE | -0.094 | -0.122 | 0.017 | 0.169 | 6.07E-08 | 0.468 | 29.342 |
|  | rs9574096 | A | T | TRUE | -0.074 | -0.152 | 0.016 | 0.154 | 2.10E-06 | 0.323 | 22.504 |
|  | rs9895850 | T | C | FALSE | -0.193 | 0.143 | 0.042 | 0.365 | 3.66E-06 | 0.695 | 21.437 |
| Genus Holdemanella(id.11393) | rs12415649 | G | C | TRUE | 0.084 | 0.160 | 0.019 | 0.179 | 1.06E-05 | 0.372 | 19.401 |
|  | rs12513188 | G | A | FALSE | 0.090 | -0.018 | 0.020 | 0.169 | 3.68E-06 | 0.916 | 21.426 |
|  | rs17586763 | T | C | FALSE | -0.227 | -0.511 | 0.051 | 0.331 | 8.37E-06 | 0.122 | 19.850 |
|  | rs1830029 | C | G | TRUE | -0.095 | -0.052 | 0.021 | 0.178 | 6.09E-06 | 0.768 | 20.460 |
|  | rs1926302 | G | A | FALSE | -0.108 | -0.343 | 0.023 | 0.180 | 3.08E-06 | 0.057 | 21.768 |
|  | rs34187114 | C | A | FALSE | -0.105 | -0.148 | 0.023 | 0.234 | 3.77E-06 | 0.527 | 21.381 |
|  | rs35228298 | G | A | FALSE | 0.093 | -0.168 | 0.020 | 0.203 | 4.07E-06 | 0.407 | 21.231 |
|  | rs4541991 | T | C | FALSE | -0.093 | -0.254 | 0.019 | 0.160 | 1.85E-06 | 0.112 | 22.747 |
|  | rs4802175 | G | A | FALSE | 0.079 | -0.124 | 0.017 | 0.160 | 5.19E-06 | 0.441 | 20.766 |
|  | rs607782 | T | C | FALSE | -0.085 | 0.025 | 0.017 | 0.155 | 7.36E-07 | 0.873 | 24.518 |
|  | rs62113381 | T | C | FALSE | -0.105 | 0.114 | 0.023 | 0.220 | 5.50E-06 | 0.603 | 20.653 |
|  | rs73011279 | T | C | FALSE | -0.096 | -0.188 | 0.020 | 0.177 | 1.40E-06 | 0.286 | 23.274 |
|  | rs75764681 | T | C | FALSE | -0.283 | 0.418 | 0.060 | 0.367 | 2.29E-06 | 0.256 | 22.338 |
|  | rs761624 | C | G | TRUE | 0.096 | 0.285 | 0.018 | 0.162 | 8.99E-08 | 0.078 | 28.579 |
| Genus Holdemania(id.2157) | rs10885477 | T | C | FALSE | -0.135 | -0.141 | 0.030 | 0.339 | 7.60E-06 | 0.678 | 20.037 |
|  | rs111745969 | A | G | FALSE | 0.121 | -0.099 | 0.027 | 0.219 | 5.61E-06 | 0.653 | 20.616 |
|  | rs113593397 | A | G | FALSE | -0.129 | 0.205 | 0.028 | 0.252 | 5.03E-06 | 0.416 | 20.827 |
|  | rs116500994 | G | T | FALSE | -0.138 | -0.058 | 0.029 | 0.353 | 2.75E-06 | 0.870 | 21.981 |
|  | rs12701617 | A | G | FALSE | -0.066 | 0.014 | 0.015 | 0.148 | 9.82E-06 | 0.924 | 19.545 |
|  | rs150096134 | T | A | TRUE | 0.162 | -0.225 | 0.033 | 0.249 | 1.05E-06 | 0.367 | 23.838 |
|  | rs1867876 | T | C | FALSE | 0.084 | 0.135 | 0.016 | 0.163 | 2.02E-07 | 0.407 | 27.009 |
|  | rs41438744 | C | G | TRUE | -0.125 | -0.269 | 0.027 | 0.276 | 3.79E-06 | 0.330 | 21.369 |
|  | rs4146507 | C | T | FALSE | 0.079 | -0.335 | 0.018 | 0.174 | 7.10E-06 | 0.054 | 20.167 |
|  | rs4636956 | A | G | FALSE | -0.067 | -0.011 | 0.015 | 0.151 | 8.84E-06 | 0.944 | 19.748 |
|  | rs55888180 | C | G | TRUE | 0.129 | -0.273 | 0.028 | 0.329 | 5.37E-06 | 0.407 | 20.702 |
|  | rs73139538 | G | A | FALSE | -0.149 | 0.046 | 0.033 | 0.437 | 5.69E-06 | 0.917 | 20.590 |
|  | rs77293403 | A | G | FALSE | 0.165 | 0.087 | 0.034 | 0.396 | 1.47E-06 | 0.827 | 23.182 |
|  | rs80149660 | C | T | FALSE | -0.233 | -0.746 | 0.052 | 0.363 | 7.20E-06 | 0.040 | 20.138 |
|  | rs9500080 | C | T | FALSE | 0.093 | 0.028 | 0.018 | 0.196 | 2.21E-07 | 0.885 | 26.840 |
|  | rs9529719 | T | C | FALSE | 0.074 | -0.276 | 0.016 | 0.159 | 3.97E-06 | 0.083 | 21.281 |
|  | rs967319 | T | C | FALSE | 0.079 | 0.320 | 0.018 | 0.174 | 8.12E-06 | 0.066 | 19.910 |
| Genus Howardella(id.2000) | rs10048062 | C | T | FALSE | -0.147 | -0.101 | 0.034 | 0.261 | 1.19E-05 | 0.699 | 19.172 |
|  | rs12452946 | A | G | FALSE | -0.106 | -0.087 | 0.023 | 0.149 | 3.79E-06 | 0.560 | 21.370 |
|  | rs1484873 | A | G | FALSE | -0.228 | 0.293 | 0.046 | 0.210 | 8.79E-07 | 0.164 | 24.177 |
|  | rs17167098 | G | A | FALSE | -0.169 | 0.004 | 0.035 | 0.216 | 1.50E-06 | 0.986 | 23.142 |
|  | rs2154047 | C | A | FALSE | -0.193 | 0.183 | 0.042 | 0.260 | 4.57E-06 | 0.482 | 21.010 |
|  | rs36081916 | T | C | FALSE | -0.181 | -0.167 | 0.040 | 0.268 | 6.89E-06 | 0.533 | 20.224 |
|  | rs3791893 | A | G | FALSE | 0.147 | 0.083 | 0.034 | 0.216 | 1.55E-05 | 0.702 | 18.677 |
|  | rs609430 | T | G | FALSE | -0.112 | -0.044 | 0.024 | 0.156 | 2.85E-06 | 0.778 | 21.918 |
|  | rs61771805 | A | T | TRUE | -0.137 | -0.273 | 0.030 | 0.228 | 4.06E-06 | 0.232 | 21.236 |
|  | rs672217 | G | A | FALSE | 0.164 | -0.049 | 0.035 | 0.187 | 2.73E-06 | 0.794 | 21.996 |
| Genus Hungatella(id.11306) | rs10044993 | C | A | FALSE | 0.140 | 0.352 | 0.032 | 0.266 | 1.05E-05 | 0.185 | 19.409 |
|  | rs13249325 | T | G | FALSE | -0.100 | 0.192 | 0.023 | 0.149 | 9.51E-06 | 0.199 | 19.608 |
|  | rs17092615 | G | A | FALSE | 0.152 | -0.133 | 0.034 | 0.217 | 6.61E-06 | 0.540 | 20.302 |
|  | rs34471047 | A | G | FALSE | -0.163 | -0.046 | 0.033 | 0.194 | 1.15E-06 | 0.811 | 23.651 |
|  | rs72759041 | G | T | FALSE | -0.126 | -0.118 | 0.028 | 0.185 | 8.00E-06 | 0.524 | 19.937 |
| Genus Intestinibacter(id.11345) | rs10805326 | G | A | FALSE | 0.078 | -0.132 | 0.014 | 0.163 | 2.86E-08 | 0.418 | 30.803 |
|  | rs10805326 | G | A | FALSE | 0.078 | -1.081 | 0.014 | 1.413 | 2.86E-08 | 0.444 | 30.803 |
|  | rs11109097 | C | T | FALSE | 0.062 | -0.287 | 0.014 | 0.150 | 6.60E-06 | 0.056 | 20.305 |
|  | rs118030283 | G | A | FALSE | -0.152 | -0.155 | 0.032 | 0.356 | 2.88E-06 | 0.663 | 21.896 |
|  | rs12210583 | T | G | FALSE | 0.155 | -0.112 | 0.035 | 0.393 | 1.12E-05 | 0.776 | 19.298 |
|  | rs16938435 | T | C | FALSE | -0.112 | -0.166 | 0.024 | 0.251 | 1.89E-06 | 0.508 | 22.706 |
|  | rs2098844 | C | T | FALSE | -0.058 | 0.197 | 0.013 | 0.154 | 7.47E-06 | 0.200 | 20.070 |
|  | rs2702387 | A | G | FALSE | 0.061 | 0.125 | 0.013 | 0.151 | 4.12E-06 | 0.409 | 21.208 |
|  | rs4327025 | G | A | FALSE | -0.081 | -0.065 | 0.015 | 0.191 | 1.53E-07 | 0.735 | 27.546 |
|  | rs447950 | A | G | FALSE | 0.063 | 0.099 | 0.014 | 0.153 | 4.26E-06 | 0.518 | 21.143 |
|  | rs478972 | T | C | FALSE | -0.143 | -0.218 | 0.030 | 0.260 | 1.57E-06 | 0.403 | 23.061 |
|  | rs6062862 | A | G | FALSE | 0.092 | 0.074 | 0.020 | 0.271 | 6.26E-06 | 0.785 | 20.406 |
|  | rs68093214 | C | T | FALSE | 0.066 | -0.068 | 0.015 | 0.173 | 9.93E-06 | 0.693 | 19.525 |
|  | rs6875660 | C | T | FALSE | 0.089 | -0.130 | 0.019 | 0.310 | 4.39E-06 | 0.674 | 21.088 |
|  | rs893394 | G | A | FALSE | 0.058 | 0.118 | 0.013 | 0.151 | 8.12E-06 | 0.434 | 19.910 |
|  | rs9348442 | C | T | FALSE | 0.099 | -0.116 | 0.022 | 0.227 | 7.80E-06 | 0.610 | 19.987 |
| Genus Intestinimonas(id.2062) | rs1000888 | C | G | TRUE | -0.059 | -0.003 | 0.013 | 0.157 | 9.61E-06 | 0.983 | 19.587 |
|  | rs10262702 | T | C | FALSE | 0.092 | 0.175 | 0.019 | 0.229 | 2.47E-06 | 0.444 | 22.189 |
|  | rs11258178 | A | G | FALSE | 0.066 | 0.004 | 0.013 | 0.149 | 8.40E-07 | 0.980 | 24.264 |
|  | rs12226153 | A | G | FALSE | -0.151 | -0.237 | 0.031 | 0.605 | 8.46E-07 | 0.695 | 24.250 |
|  | rs12566247 | T | A | TRUE | 0.064 | 0.049 | 0.014 | 0.151 | 2.52E-06 | 0.744 | 22.153 |
|  | rs17067892 | C | T | FALSE | 0.107 | 0.006 | 0.025 | 0.263 | 1.81E-05 | 0.983 | 18.383 |
|  | rs1859797 | G | A | FALSE | 0.060 | -0.059 | 0.013 | 0.150 | 4.64E-06 | 0.693 | 20.981 |
|  | rs2276760 | A | G | FALSE | -0.069 | -0.083 | 0.015 | 0.175 | 7.06E-06 | 0.637 | 20.178 |
|  | rs2731794 | C | T | FALSE | 0.121 | -0.220 | 0.026 | 0.401 | 2.81E-06 | 0.583 | 21.942 |
|  | rs2930225 | G | T | FALSE | 0.073 | -0.084 | 0.015 | 0.177 | 1.84E-06 | 0.637 | 22.750 |
|  | rs4113676 | A | C | FALSE | -0.219 | 0.310 | 0.049 | 0.607 | 8.27E-06 | 0.609 | 19.873 |
|  | rs4784055 | T | C | FALSE | -0.175 | -0.426 | 0.039 | 0.339 | 5.57E-06 | 0.210 | 20.631 |
|  | rs62240188 | G | A | FALSE | 0.130 | -0.430 | 0.027 | 0.256 | 1.13E-06 | 0.092 | 23.701 |
|  | rs6934519 | C | T | FALSE | 0.069 | 0.094 | 0.015 | 0.170 | 4.64E-06 | 0.583 | 20.982 |
|  | rs716604 | A | G | FALSE | 0.082 | -0.290 | 0.017 | 0.177 | 8.29E-07 | 0.102 | 24.289 |
|  | rs7170984 | T | C | FALSE | -0.066 | -0.060 | 0.014 | 0.166 | 2.94E-06 | 0.717 | 21.858 |
|  | rs72982915 | C | T | FALSE | 0.183 | 0.523 | 0.040 | 0.324 | 5.42E-06 | 0.107 | 20.682 |
|  | rs9823439 | T | C | FALSE | -0.058 | -0.117 | 0.013 | 0.149 | 9.56E-06 | 0.433 | 19.598 |
|  | rs994794 | G | C | TRUE | -0.142 | 0.582 | 0.032 | 0.423 | 6.67E-06 | 0.169 | 20.285 |
| Genus Lachnoclostridium(id.11308) | rs1031599 | G | T | FALSE | -0.079 | 0.213 | 0.018 | 0.303 | 7.59E-06 | 0.481 | 20.039 |
|  | rs12566975 | T | C | FALSE | -0.047 | -0.241 | 0.011 | 0.149 | 9.65E-06 | 0.106 | 19.580 |
|  | rs1528479 | G | A | FALSE | -0.050 | -0.137 | 0.011 | 0.154 | 8.67E-06 | 0.374 | 19.783 |
|  | rs1997204 | T | C | FALSE | -0.108 | 0.870 | 0.024 | 0.359 | 7.99E-06 | 0.015 | 19.941 |
|  | rs2385421 | A | G | FALSE | 0.075 | 0.003 | 0.018 | 0.233 | 3.65E-05 | 0.990 | 17.046 |
|  | rs3821998 | C | A | FALSE | -0.086 | -0.113 | 0.019 | 0.244 | 7.18E-06 | 0.642 | 20.144 |
|  | rs4738679 | G | A | FALSE | -0.052 | 0.100 | 0.011 | 0.153 | 5.06E-06 | 0.512 | 20.813 |
|  | rs6112314 | A | C | FALSE | -0.056 | 0.292 | 0.011 | 0.157 | 2.07E-07 | 0.063 | 26.964 |
|  | rs615997 | T | C | FALSE | 0.051 | 0.261 | 0.011 | 0.148 | 1.54E-06 | 0.078 | 23.094 |
|  | rs61915992 | A | T | TRUE | 0.080 | -0.039 | 0.017 | 0.214 | 3.04E-06 | 0.857 | 21.791 |
|  | rs62028349 | G | C | TRUE | 0.047 | -0.181 | 0.011 | 0.149 | 9.20E-06 | 0.224 | 19.670 |
|  | rs62285313 | A | G | FALSE | 0.086 | 0.040 | 0.018 | 0.248 | 1.94E-06 | 0.872 | 22.655 |
|  | rs72829893 | G | T | FALSE | 0.117 | -0.144 | 0.027 | 0.246 | 1.18E-05 | 0.557 | 19.199 |
|  | rs78068103 | A | G | FALSE | 0.089 | -0.544 | 0.019 | 0.235 | 5.06E-06 | 0.020 | 20.814 |
|  | rs789029 | C | T | FALSE | -0.064 | 0.134 | 0.014 | 0.212 | 3.35E-06 | 0.529 | 21.603 |
| Genus Lachnospira(id.2004) | rs13157098 | A | G | FALSE | -0.077 | 0.139 | 0.016 | 0.202 | 7.60E-07 | 0.492 | 24.456 |
|  | rs2326833 | C | G | TRUE | -0.078 | 0.024 | 0.017 | 0.205 | 4.48E-06 | 0.906 | 21.049 |
|  | rs2520509 | A | G | FALSE | 0.052 | -0.390 | 0.012 | 0.163 | 7.35E-06 | 0.017 | 20.099 |
|  | rs4686798 | T | C | FALSE | 0.053 | 0.098 | 0.011 | 0.155 | 2.93E-06 | 0.525 | 21.860 |
|  | rs4923324 | G | A | FALSE | -0.062 | -0.018 | 0.013 | 0.202 | 3.69E-06 | 0.930 | 21.419 |
|  | rs56791201 | T | C | FALSE | 0.052 | -0.005 | 0.011 | 0.155 | 2.85E-06 | 0.974 | 21.917 |
| Genus LachnospiraceaeFCS020group(id.11314) | rs10030408 | A | G | FALSE | 0.055 | -0.049 | 0.012 | 0.148 | 7.48E-06 | 0.740 | 20.066 |
|  | rs10093861 | G | A | FALSE | -0.057 | -0.160 | 0.012 | 0.151 | 2.66E-06 | 0.289 | 22.048 |
|  | rs113859143 | G | C | TRUE | -0.109 | 0.195 | 0.024 | 0.324 | 6.82E-06 | 0.548 | 20.243 |
|  | rs12078956 | C | G | TRUE | 0.106 | 0.043 | 0.022 | 0.241 | 1.94E-06 | 0.859 | 22.657 |
|  | rs1254846 | G | A | FALSE | 0.106 | 0.155 | 0.023 | 0.226 | 5.18E-06 | 0.492 | 20.771 |
|  | rs1363769 | T | C | FALSE | -0.201 | -0.030 | 0.045 | 0.411 | 8.02E-06 | 0.941 | 19.933 |
|  | rs2322265 | C | T | FALSE | -0.067 | -0.119 | 0.014 | 0.170 | 2.52E-06 | 0.484 | 22.149 |
|  | rs2862811 | T | C | FALSE | 0.056 | -0.149 | 0.012 | 0.161 | 3.47E-06 | 0.357 | 21.535 |
|  | rs35035870 | T | C | FALSE | -0.191 | 0.284 | 0.041 | 0.377 | 4.23E-06 | 0.450 | 21.158 |
|  | rs369444 | C | G | TRUE | 0.125 | 0.329 | 0.026 | 0.288 | 1.27E-06 | 0.253 | 23.471 |
|  | rs3999074 | G | T | FALSE | -0.055 | 0.209 | 0.012 | 0.149 | 6.22E-06 | 0.161 | 20.418 |
|  | rs4452603 | T | G | FALSE | 0.060 | -0.109 | 0.014 | 0.169 | 8.84E-06 | 0.518 | 19.748 |
|  | rs7249113 | G | A | FALSE | 0.068 | -0.165 | 0.013 | 0.163 | 3.58E-07 | 0.310 | 25.907 |
|  | rs72793667 | A | G | FALSE | -0.117 | -0.590 | 0.025 | 0.375 | 2.14E-06 | 0.116 | 22.467 |
|  | rs9788306 | C | T | FALSE | -0.063 | 0.093 | 0.013 | 0.165 | 1.56E-06 | 0.572 | 23.074 |
|  | rs9919338 | G | C | TRUE | -0.055 | 0.081 | 0.012 | 0.149 | 4.49E-06 | 0.588 | 21.044 |
| Genus LachnospiraceaeNC2004group(id.11316) | rs117467633 | T | C | FALSE | -0.170 | -0.482 | 0.038 | 0.376 | 9.49E-06 | 0.200 | 19.612 |
|  | rs12127733 | G | A | FALSE | 0.115 | 0.328 | 0.025 | 0.193 | 2.84E-06 | 0.089 | 21.922 |
|  | rs12208226 | C | A | FALSE | -0.155 | -0.117 | 0.034 | 0.243 | 5.46E-06 | 0.630 | 20.668 |
|  | rs12863463 | G | A | FALSE | -0.156 | -0.176 | 0.035 | 0.277 | 5.97E-06 | 0.525 | 20.498 |
|  | rs1331592 | C | G | TRUE | 0.095 | -0.034 | 0.021 | 0.175 | 5.28E-06 | 0.846 | 20.734 |
|  | rs17067076 | G | A | FALSE | -0.155 | -0.248 | 0.035 | 0.235 | 1.13E-05 | 0.291 | 19.277 |
|  | rs1928659 | T | C | FALSE | 0.103 | 0.342 | 0.023 | 0.185 | 5.97E-06 | 0.065 | 20.498 |
|  | rs1929743 | T | C | FALSE | 0.084 | -0.216 | 0.019 | 0.157 | 1.09E-05 | 0.171 | 19.351 |
|  | rs3756315 | A | G | FALSE | -0.088 | 0.169 | 0.019 | 0.163 | 2.74E-06 | 0.301 | 21.991 |
|  | rs6116753 | G | A | FALSE | 0.099 | 0.157 | 0.021 | 0.193 | 1.97E-06 | 0.415 | 22.623 |
| Genus LachnospiraceaeND3007group(id.11317) | rs2861203 | G | A | FALSE | 0.057 | 0.162 | 0.013 | 0.163 | 6.91E-06 | 0.321 | 20.218 |
|  | rs72776675 | T | C | FALSE | -0.065 | -0.098 | 0.015 | 0.197 | 1.23E-05 | 0.620 | 19.123 |
|  | rs9932954 | A | G | FALSE | -0.056 | 0.134 | 0.012 | 0.157 | 1.27E-06 | 0.395 | 23.467 |
| Genus LachnospiraceaeNK4A136group(id.11319) | rs10952110 | G | T | FALSE | 0.049 | 0.113 | 0.011 | 0.150 | 8.61E-06 | 0.452 | 19.796 |
|  | rs11263806 | A | G | FALSE | -0.052 | -0.054 | 0.012 | 0.157 | 7.02E-06 | 0.731 | 20.189 |
|  | rs12362320 | G | C | TRUE | 0.057 | 0.079 | 0.012 | 0.151 | 6.91E-07 | 0.600 | 24.641 |
|  | rs12611395 | A | G | FALSE | -0.090 | 0.316 | 0.020 | 0.242 | 6.17E-06 | 0.192 | 20.435 |
|  | rs160061 | A | G | FALSE | 0.051 | 0.166 | 0.011 | 0.149 | 2.00E-06 | 0.264 | 22.596 |
|  | rs28540839 | A | C | FALSE | 0.051 | -0.097 | 0.011 | 0.149 | 4.31E-06 | 0.517 | 21.124 |
|  | rs2880566 | T | C | FALSE | 0.060 | -0.127 | 0.013 | 0.209 | 8.53E-06 | 0.544 | 19.815 |
|  | rs4955932 | T | C | FALSE | -0.049 | 0.016 | 0.011 | 0.154 | 6.78E-06 | 0.917 | 20.253 |
|  | rs59805249 | T | C | FALSE | 0.094 | -0.358 | 0.021 | 0.261 | 6.76E-06 | 0.170 | 20.260 |
|  | rs68104925 | T | C | FALSE | -0.055 | -0.087 | 0.012 | 0.162 | 1.95E-06 | 0.591 | 22.647 |
|  | rs7073658 | T | G | FALSE | -0.050 | 0.209 | 0.011 | 0.149 | 5.25E-06 | 0.162 | 20.746 |
|  | rs73044693 | A | G | FALSE | -0.108 | 0.098 | 0.023 | 0.291 | 2.87E-06 | 0.737 | 21.900 |
|  | rs7616165 | G | T | FALSE | -0.231 | 0.427 | 0.048 | 0.458 | 1.86E-06 | 0.351 | 22.739 |
|  | rs76193507 | A | G | FALSE | -0.230 | 0.404 | 0.050 | 0.267 | 4.29E-06 | 0.130 | 21.129 |
|  | rs7832116 | A | G | FALSE | -0.071 | -0.247 | 0.015 | 0.224 | 2.46E-06 | 0.270 | 22.199 |
|  | rs954878 | A | G | FALSE | -0.052 | -0.113 | 0.011 | 0.154 | 1.81E-06 | 0.463 | 22.782 |
| Genus LachnospiraceaeUCG001(id.11321) | rs10815577 | C | G | TRUE | -0.068 | 0.070 | 0.014 | 0.150 | 1.95E-06 | 0.643 | 22.640 |
|  | rs12131224 | C | T | FALSE | 0.117 | 0.009 | 0.026 | 0.240 | 6.20E-06 | 0.972 | 20.424 |
|  | rs2050911 | G | A | FALSE | 0.075 | 0.058 | 0.015 | 0.157 | 1.05E-06 | 0.712 | 23.831 |
|  | rs2371284 | T | C | FALSE | -0.076 | -0.220 | 0.017 | 0.178 | 7.52E-06 | 0.216 | 20.056 |
|  | rs437876 | T | C | FALSE | 0.078 | -0.152 | 0.014 | 0.157 | 5.96E-08 | 0.334 | 29.376 |
|  | rs4981345 | T | C | FALSE | -0.068 | 0.182 | 0.015 | 0.159 | 5.32E-06 | 0.253 | 20.717 |
|  | rs573933 | T | C | FALSE | -0.108 | -0.951 | 0.023 | 0.256 | 3.42E-06 | 0.000 | 21.565 |
|  | rs62496417 | T | G | FALSE | -0.075 | -0.174 | 0.017 | 0.180 | 6.25E-06 | 0.334 | 20.410 |
|  | rs7213933 | T | A | TRUE | -0.082 | 0.497 | 0.018 | 0.204 | 8.95E-06 | 0.015 | 19.723 |
|  | rs7341608 | T | C | FALSE | -0.078 | -0.180 | 0.018 | 0.217 | 9.99E-06 | 0.406 | 19.514 |
|  | rs78848836 | A | G | FALSE | -0.119 | 0.084 | 0.026 | 0.245 | 4.74E-06 | 0.731 | 20.942 |
|  | rs79476906 | T | A | TRUE | -0.087 | 0.002 | 0.020 | 0.206 | 9.85E-06 | 0.992 | 19.541 |
|  | rs8052586 | T | C | FALSE | 0.176 | 0.371 | 0.040 | 0.310 | 8.42E-06 | 0.232 | 19.841 |
|  | rs8104225 | A | G | FALSE | 0.089 | 0.052 | 0.020 | 0.179 | 6.39E-06 | 0.770 | 20.369 |
|  | rs9403580 | C | T | FALSE | 0.108 | 0.149 | 0.023 | 0.221 | 2.71E-06 | 0.500 | 22.010 |
|  | rs985416 | C | T | FALSE | 0.097 | -0.297 | 0.018 | 0.193 | 9.46E-08 | 0.124 | 28.481 |
| Genus LachnospiraceaeUCG004(id.11324) | rs11128180 | A | G | FALSE | 0.065 | -0.223 | 0.014 | 0.176 | 3.72E-06 | 0.204 | 21.404 |
|  | rs12072562 | T | C | FALSE | 0.133 | 0.114 | 0.030 | 0.382 | 1.16E-05 | 0.766 | 19.225 |
|  | rs12673420 | G | A | FALSE | 0.055 | -0.007 | 0.012 | 0.149 | 2.83E-06 | 0.961 | 21.930 |
|  | rs12747809 | G | A | FALSE | -0.062 | -0.090 | 0.013 | 0.165 | 7.47E-07 | 0.587 | 24.489 |
|  | rs12894272 | A | G | FALSE | 0.058 | -0.059 | 0.013 | 0.157 | 3.66E-06 | 0.704 | 21.436 |
|  | rs233486 | A | G | FALSE | -0.080 | -0.085 | 0.018 | 0.213 | 6.86E-06 | 0.690 | 20.231 |
|  | rs2444793 | C | T | FALSE | -0.054 | 0.017 | 0.012 | 0.152 | 4.44E-06 | 0.909 | 21.064 |
|  | rs2706242 | G | C | TRUE | -0.090 | 0.254 | 0.020 | 0.267 | 6.38E-06 | 0.340 | 20.372 |
|  | rs2726805 | A | G | FALSE | 0.055 | -0.121 | 0.012 | 0.151 | 5.68E-06 | 0.421 | 20.594 |
|  | rs2882478 | G | A | FALSE | -0.058 | 0.154 | 0.012 | 0.149 | 1.08E-06 | 0.303 | 23.781 |
|  | rs35182105 | A | G | FALSE | -0.110 | 0.769 | 0.024 | 0.333 | 5.89E-06 | 0.021 | 20.522 |
|  | rs6656451 | C | T | FALSE | -0.054 | 0.225 | 0.012 | 0.149 | 5.36E-06 | 0.130 | 20.703 |
|  | rs7629954 | A | G | FALSE | 0.108 | -0.332 | 0.024 | 0.353 | 5.32E-06 | 0.348 | 20.717 |
| Genus LachnospiraceaeUCG008(id.11328) | rs10741777 | T | C | FALSE | -0.097 | 0.162 | 0.019 | 0.161 | 5.75E-07 | 0.315 | 24.994 |
|  | rs10793103 | C | T | FALSE | 0.097 | 0.097 | 0.018 | 0.150 | 7.67E-08 | 0.517 | 28.889 |
|  | rs10801803 | G | A | FALSE | -0.117 | 0.264 | 0.024 | 0.213 | 1.48E-06 | 0.217 | 23.178 |
|  | rs13024781 | T | C | FALSE | -0.080 | 0.127 | 0.017 | 0.149 | 2.24E-06 | 0.394 | 22.380 |
|  | rs57091572 | A | G | FALSE | -0.110 | 0.041 | 0.024 | 0.218 | 2.82E-06 | 0.851 | 21.937 |
|  | rs57254474 | G | A | FALSE | 0.089 | 0.068 | 0.020 | 0.181 | 8.44E-06 | 0.706 | 19.837 |
|  | rs61944774 | A | G | FALSE | 0.180 | 0.159 | 0.039 | 0.339 | 4.95E-06 | 0.639 | 20.855 |
|  | rs62277846 | C | T | FALSE | 0.102 | 0.115 | 0.021 | 0.189 | 1.45E-06 | 0.541 | 23.212 |
|  | rs67078837 | T | C | FALSE | -0.085 | -0.182 | 0.017 | 0.151 | 7.22E-07 | 0.227 | 24.556 |
|  | rs75356640 | G | A | FALSE | 0.137 | 0.043 | 0.030 | 0.230 | 6.67E-06 | 0.851 | 20.284 |
|  | rs955844 | A | C | FALSE | 0.112 | 0.033 | 0.023 | 0.213 | 9.24E-07 | 0.878 | 24.080 |
|  | rs9873555 | G | C | TRUE | -0.121 | 0.038 | 0.023 | 0.232 | 2.11E-07 | 0.871 | 26.933 |
| Genus LachnospiraceaeUCG010(id.11330) | rs10414815 | T | C | FALSE | 0.105 | 0.119 | 0.023 | 0.348 | 5.63E-06 | 0.734 | 20.610 |
|  | rs11192447 | A | G | FALSE | 0.127 | 0.710 | 0.024 | 0.344 | 1.99E-07 | 0.039 | 27.039 |
|  | rs12346653 | C | T | FALSE | 0.066 | -0.330 | 0.014 | 0.182 | 2.45E-06 | 0.069 | 22.205 |
|  | rs17730011 | G | A | FALSE | -0.070 | -0.018 | 0.016 | 0.183 | 7.75E-06 | 0.920 | 19.998 |
|  | rs2153460 | A | T | TRUE | -0.068 | -0.237 | 0.016 | 0.200 | 1.25E-05 | 0.236 | 19.089 |
|  | rs2833528 | C | T | FALSE | -0.056 | 0.035 | 0.013 | 0.154 | 1.08E-05 | 0.821 | 19.359 |
|  | rs336138 | G | T | FALSE | 0.078 | 0.265 | 0.017 | 0.231 | 5.74E-06 | 0.252 | 20.573 |
|  | rs4576377 | A | C | FALSE | -0.057 | -0.350 | 0.013 | 0.155 | 6.72E-06 | 0.024 | 20.272 |
|  | rs72761829 | A | T | TRUE | 0.112 | -0.148 | 0.024 | 0.266 | 2.82E-06 | 0.578 | 21.934 |
|  | rs72894957 | G | A | FALSE | 0.222 | -0.054 | 0.049 | 0.493 | 4.89E-06 | 0.914 | 20.879 |
|  | rs74315802 | G | T | FALSE | 0.087 | 0.043 | 0.018 | 0.196 | 2.28E-06 | 0.829 | 22.343 |
|  | rs9981767 | A | C | FALSE | 0.066 | -0.086 | 0.013 | 0.170 | 6.95E-07 | 0.615 | 24.629 |
| Genus Lactobacillus(id.1837) | rs12693845 | C | T | FALSE | -0.081 | 0.019 | 0.018 | 0.154 | 5.64E-06 | 0.903 | 20.608 |
|  | rs1530559 | G | A | FALSE | 0.080 | -0.025 | 0.018 | 0.151 | 6.43E-06 | 0.869 | 20.355 |
|  | rs16861661 | G | A | FALSE | -0.183 | -0.649 | 0.038 | 0.304 | 1.58E-06 | 0.033 | 23.049 |
|  | rs328312 | T | A | TRUE | 0.082 | 0.172 | 0.017 | 0.149 | 1.51E-06 | 0.250 | 23.141 |
|  | rs6092149 | A | T | TRUE | -0.080 | -0.160 | 0.017 | 0.151 | 2.97E-06 | 0.289 | 21.835 |
|  | rs62314653 | C | A | FALSE | 0.188 | 0.337 | 0.039 | 0.318 | 1.97E-06 | 0.289 | 22.626 |
|  | rs7399658 | G | A | FALSE | -0.107 | 0.339 | 0.022 | 0.195 | 1.38E-06 | 0.082 | 23.313 |
|  | rs75127669 | C | A | FALSE | 0.140 | -0.151 | 0.031 | 0.287 | 6.70E-06 | 0.598 | 20.278 |
|  | rs768253 | T | G | FALSE | -0.079 | 0.163 | 0.017 | 0.150 | 4.03E-06 | 0.276 | 21.252 |
|  | rs77478751 | A | G | FALSE | -0.220 | -0.050 | 0.048 | 0.234 | 3.80E-06 | 0.830 | 21.361 |
|  | rs921925 | A | C | FALSE | 0.099 | 0.090 | 0.020 | 0.181 | 1.25E-06 | 0.619 | 23.495 |
| Genus Lactococcus(id.1851) | rs10417872 | T | G | FALSE | 0.118 | -0.102 | 0.025 | 0.164 | 1.40E-06 | 0.534 | 23.276 |
|  | rs123059 | T | C | FALSE | -0.137 | 0.071 | 0.027 | 0.181 | 6.46E-07 | 0.693 | 24.769 |
|  | rs12621813 | G | A | FALSE | 0.108 | -0.125 | 0.024 | 0.168 | 6.24E-06 | 0.459 | 20.413 |
|  | rs17168302 | G | A | FALSE | 0.192 | -0.229 | 0.042 | 0.245 | 6.28E-06 | 0.351 | 20.402 |
|  | rs2293361 | C | T | FALSE | -0.199 | 0.348 | 0.043 | 0.329 | 3.79E-06 | 0.290 | 21.369 |
|  | rs34757988 | G | C | TRUE | 0.122 | 0.105 | 0.023 | 0.153 | 9.25E-08 | 0.490 | 28.525 |
|  | rs4766997 | C | T | FALSE | 0.115 | 0.190 | 0.024 | 0.150 | 1.53E-06 | 0.206 | 23.109 |
|  | rs55910161 | C | T | FALSE | 0.146 | -0.312 | 0.031 | 0.237 | 1.90E-06 | 0.188 | 22.695 |
|  | rs6674304 | C | T | FALSE | 0.201 | 0.449 | 0.044 | 0.387 | 5.60E-06 | 0.246 | 20.619 |
|  | rs757872 | G | C | TRUE | 0.141 | -0.121 | 0.028 | 0.197 | 3.31E-07 | 0.540 | 26.058 |
|  | rs7992246 | T | C | FALSE | 0.104 | 0.105 | 0.023 | 0.152 | 6.29E-06 | 0.491 | 20.398 |
| Genus Marvinbryantia(id.2005) | rs11620597 | T | C | FALSE | 0.119 | 0.034 | 0.027 | 0.466 | 1.09E-05 | 0.943 | 19.340 |
|  | rs11645029 | G | C | TRUE | -0.061 | 0.356 | 0.013 | 0.149 | 4.07E-06 | 0.017 | 21.233 |
|  | rs1187983 | C | T | FALSE | -0.094 | 0.249 | 0.019 | 0.245 | 1.28E-06 | 0.310 | 23.450 |
|  | rs146541147 | G | A | FALSE | 0.119 | 0.377 | 0.027 | 0.412 | 9.53E-06 | 0.359 | 19.603 |
|  | rs2724813 | A | G | FALSE | -0.084 | -0.156 | 0.017 | 0.175 | 5.22E-07 | 0.371 | 25.180 |
|  | rs2842896 | C | T | FALSE | -0.065 | 0.089 | 0.013 | 0.151 | 7.36E-07 | 0.558 | 24.519 |
|  | rs2863363 | A | G | FALSE | 0.063 | -0.210 | 0.014 | 0.172 | 3.21E-06 | 0.223 | 21.688 |
|  | rs3125832 | A | C | FALSE | 0.068 | -0.013 | 0.015 | 0.179 | 6.03E-06 | 0.942 | 20.477 |
|  | rs61884471 | G | A | FALSE | 0.124 | 0.161 | 0.025 | 0.239 | 5.49E-07 | 0.500 | 25.085 |
|  | rs72948274 | A | C | FALSE | -0.126 | 0.034 | 0.027 | 0.304 | 3.45E-06 | 0.912 | 21.546 |
|  | rs8006832 | G | T | FALSE | -0.095 | -0.227 | 0.022 | 0.258 | 1.11E-05 | 0.378 | 19.317 |
| Genus Methanobrevibacter(id.123) | rs10202904 | T | G | FALSE | -0.113 | 0.015 | 0.024 | 0.151 | 2.38E-06 | 0.923 | 22.260 |
|  | rs11018665 | A | T | TRUE | 0.113 | -0.159 | 0.025 | 0.173 | 8.86E-06 | 0.357 | 19.743 |
|  | rs1334944 | T | C | FALSE | 0.115 | 0.093 | 0.026 | 0.166 | 6.52E-06 | 0.573 | 20.329 |
|  | rs4779844 | G | C | TRUE | 0.110 | -0.002 | 0.025 | 0.156 | 9.81E-06 | 0.989 | 19.548 |
|  | rs4802933 | A | G | FALSE | -0.136 | 0.042 | 0.031 | 0.177 | 1.07E-05 | 0.813 | 19.374 |
|  | rs6776814 | T | C | FALSE | -0.189 | -0.759 | 0.042 | 0.518 | 6.80E-06 | 0.143 | 20.250 |
|  | rs73457410 | A | G | FALSE | 0.218 | 0.466 | 0.044 | 0.301 | 9.32E-07 | 0.122 | 24.064 |
|  | rs894996 | C | A | FALSE | 0.214 | -0.519 | 0.046 | 0.286 | 2.64E-06 | 0.069 | 22.064 |
| Genus Odoribacter(id.952) | rs10093869 | A | G | FALSE | -0.058 | -0.096 | 0.013 | 0.151 | 4.07E-06 | 0.524 | 21.234 |
|  | rs10423795 | C | T | FALSE | 0.055 | 0.096 | 0.012 | 0.154 | 5.49E-06 | 0.533 | 20.657 |
|  | rs16918425 | A | T | TRUE | 0.100 | -0.455 | 0.022 | 0.345 | 7.91E-06 | 0.188 | 19.959 |
|  | rs28417404 | A | G | FALSE | -0.073 | 0.015 | 0.016 | 0.250 | 6.65E-06 | 0.951 | 20.290 |
|  | rs4793970 | A | G | FALSE | -0.058 | -0.154 | 0.013 | 0.156 | 8.11E-06 | 0.321 | 19.912 |
|  | rs503751 | C | G | TRUE | 0.062 | 0.022 | 0.012 | 0.149 | 1.99E-07 | 0.883 | 27.047 |
|  | rs6856150 | G | A | FALSE | 0.088 | 0.220 | 0.019 | 0.226 | 5.56E-06 | 0.330 | 20.635 |
|  | rs74553962 | T | G | FALSE | 0.121 | 0.463 | 0.026 | 0.283 | 4.26E-06 | 0.102 | 21.146 |
|  | rs77779484 | G | A | FALSE | -0.133 | 0.034 | 0.027 | 0.317 | 6.65E-07 | 0.915 | 24.713 |
| Genus Olsenella(id.822) | rs1035588 | A | G | FALSE | -0.108 | -0.057 | 0.024 | 0.154 | 4.97E-06 | 0.711 | 20.850 |
|  | rs17148768 | G | A | FALSE | 0.140 | 0.172 | 0.030 | 0.200 | 2.03E-06 | 0.389 | 22.570 |
|  | rs2759329 | G | A | FALSE | -0.111 | -0.169 | 0.024 | 0.154 | 2.80E-06 | 0.274 | 21.947 |
|  | rs35225860 | A | G | FALSE | -0.224 | -0.208 | 0.048 | 0.375 | 3.56E-06 | 0.579 | 21.486 |
|  | rs61090148 | A | G | FALSE | -0.105 | 0.170 | 0.023 | 0.150 | 5.92E-06 | 0.258 | 20.515 |
|  | rs62112538 | C | T | FALSE | -0.199 | -0.286 | 0.041 | 0.241 | 9.60E-07 | 0.234 | 24.006 |
|  | rs72691585 | C | A | FALSE | -0.249 | 0.047 | 0.052 | 0.220 | 1.73E-06 | 0.832 | 22.872 |
|  | rs7540303 | C | T | FALSE | 0.108 | 0.060 | 0.024 | 0.154 | 4.86E-06 | 0.696 | 20.892 |
|  | rs8066522 | G | A | FALSE | -0.107 | -0.060 | 0.024 | 0.158 | 9.35E-06 | 0.704 | 19.640 |
|  | rs9460691 | C | A | FALSE | 0.120 | 0.298 | 0.027 | 0.189 | 7.98E-06 | 0.116 | 19.941 |
| Genus Oscillibacter(id.2063) | rs11627628 | T | C | FALSE | 0.144 | 0.405 | 0.029 | 0.285 | 7.04E-07 | 0.155 | 24.605 |
|  | rs11990279 | T | C | FALSE | -0.082 | 0.069 | 0.018 | 0.204 | 4.85E-06 | 0.735 | 20.897 |
|  | rs12417956 | C | G | TRUE | 0.079 | 0.002 | 0.017 | 0.166 | 6.42E-06 | 0.990 | 20.358 |
|  | rs12649930 | T | G | FALSE | 0.122 | 0.234 | 0.026 | 0.249 | 2.82E-06 | 0.348 | 21.935 |
|  | rs133832 | A | C | FALSE | -0.080 | 0.110 | 0.016 | 0.165 | 9.67E-07 | 0.507 | 23.993 |
|  | rs137917150 | T | A | TRUE | -0.175 | 0.227 | 0.039 | 0.260 | 6.62E-06 | 0.382 | 20.300 |
|  | rs16866406 | A | G | FALSE | 0.099 | -0.083 | 0.021 | 0.206 | 2.18E-06 | 0.685 | 22.426 |
|  | rs16934185 | A | G | FALSE | -0.130 | -0.139 | 0.028 | 0.247 | 4.19E-06 | 0.574 | 21.175 |
|  | rs234108 | A | G | FALSE | 0.075 | -0.116 | 0.015 | 0.152 | 9.07E-07 | 0.446 | 24.116 |
|  | rs36095275 | C | T | FALSE | -0.075 | -0.003 | 0.016 | 0.151 | 1.62E-06 | 0.983 | 23.005 |
|  | rs4506202 | A | G | FALSE | -0.071 | -0.157 | 0.015 | 0.150 | 2.99E-06 | 0.295 | 21.825 |
|  | rs61883564 | A | G | FALSE | -0.101 | 0.413 | 0.022 | 0.216 | 4.52E-06 | 0.055 | 21.029 |
|  | rs6901560 | C | G | TRUE | 0.086 | -0.065 | 0.019 | 0.192 | 4.78E-06 | 0.736 | 20.922 |
|  | rs75453768 | G | T | FALSE | 0.122 | 0.100 | 0.027 | 0.252 | 5.47E-06 | 0.693 | 20.666 |
|  | rs761240 | T | G | FALSE | -0.177 | 0.096 | 0.039 | 0.345 | 5.54E-06 | 0.781 | 20.639 |
|  | rs9393920 | A | G | FALSE | -0.074 | 0.284 | 0.015 | 0.157 | 8.27E-07 | 0.070 | 24.294 |
| Genus Oscillospira(id.2064) | rs11627628 | T | C | FALSE | 0.144 | 0.405 | 0.029 | 0.285 | 7.04E-07 | 0.155 | 24.605 |
|  | rs11990279 | T | C | FALSE | -0.082 | 0.069 | 0.018 | 0.204 | 4.85E-06 | 0.735 | 20.897 |
|  | rs12417956 | C | G | TRUE | 0.079 | 0.002 | 0.017 | 0.166 | 6.42E-06 | 0.990 | 20.358 |
|  | rs12649930 | T | G | FALSE | 0.122 | 0.234 | 0.026 | 0.249 | 2.82E-06 | 0.348 | 21.935 |
|  | rs133832 | A | C | FALSE | -0.080 | 0.110 | 0.016 | 0.165 | 9.67E-07 | 0.507 | 23.993 |
|  | rs137917150 | T | A | TRUE | -0.175 | 0.227 | 0.039 | 0.260 | 6.62E-06 | 0.382 | 20.300 |
|  | rs16866406 | A | G | FALSE | 0.099 | -0.083 | 0.021 | 0.206 | 2.18E-06 | 0.685 | 22.426 |
|  | rs16934185 | A | G | FALSE | -0.130 | -0.139 | 0.028 | 0.247 | 4.19E-06 | 0.574 | 21.175 |
|  | rs234108 | A | G | FALSE | 0.075 | -0.116 | 0.015 | 0.152 | 9.07E-07 | 0.446 | 24.116 |
|  | rs36095275 | C | T | FALSE | -0.075 | -0.003 | 0.016 | 0.151 | 1.62E-06 | 0.983 | 23.005 |
|  | rs4506202 | A | G | FALSE | -0.071 | -0.157 | 0.015 | 0.150 | 2.99E-06 | 0.295 | 21.825 |
|  | rs61883564 | A | G | FALSE | -0.101 | 0.413 | 0.022 | 0.216 | 4.52E-06 | 0.055 | 21.029 |
|  | rs6901560 | C | G | TRUE | 0.086 | -0.065 | 0.019 | 0.192 | 4.78E-06 | 0.736 | 20.922 |
|  | rs75453768 | G | T | FALSE | 0.122 | 0.100 | 0.027 | 0.252 | 5.47E-06 | 0.693 | 20.666 |
|  | rs761240 | T | G | FALSE | -0.177 | 0.096 | 0.039 | 0.345 | 5.54E-06 | 0.781 | 20.639 |
|  | rs9393920 | A | G | FALSE | -0.074 | 0.284 | 0.015 | 0.157 | 8.27E-07 | 0.070 | 24.294 |
| Genus Oxalobacter(id.2978) | rs10464997 | G | A | FALSE | 0.138 | -0.320 | 0.029 | 0.193 | 3.00E-06 | 0.098 | 21.815 |
|  | rs11108500 | A | G | FALSE | -0.199 | 0.275 | 0.043 | 0.264 | 3.17E-06 | 0.297 | 21.708 |
|  | rs111966731 | T | C | FALSE | 0.213 | 0.187 | 0.047 | 0.265 | 6.22E-06 | 0.481 | 20.419 |
|  | rs12002250 | A | C | FALSE | 0.217 | 0.479 | 0.047 | 0.373 | 3.22E-06 | 0.200 | 21.679 |
|  | rs1569853 | T | C | FALSE | -0.138 | -0.037 | 0.030 | 0.229 | 3.33E-06 | 0.871 | 21.617 |
|  | rs36057338 | G | T | FALSE | 0.208 | 0.464 | 0.042 | 0.410 | 8.15E-07 | 0.257 | 24.323 |
|  | rs3862635 | C | T | FALSE | -0.172 | 0.031 | 0.039 | 0.258 | 1.25E-05 | 0.905 | 19.086 |
|  | rs4428215 | G | A | FALSE | 0.130 | -0.104 | 0.024 | 0.171 | 7.50E-08 | 0.542 | 28.931 |
|  | rs6000536 | C | T | FALSE | -0.131 | -0.096 | 0.025 | 0.199 | 2.45E-07 | 0.628 | 26.637 |
|  | rs6071435 | T | A | TRUE | -0.106 | -0.153 | 0.021 | 0.153 | 9.10E-07 | 0.317 | 24.109 |
|  | rs6993398 | G | A | FALSE | 0.127 | -0.016 | 0.028 | 0.192 | 5.06E-06 | 0.936 | 20.813 |
|  | rs736744 | C | T | FALSE | 0.118 | -0.144 | 0.021 | 0.150 | 2.41E-08 | 0.337 | 31.135 |
| Genus Parabacteroides(id.954) | rs115602804 | G | A | FALSE | 0.103 | -0.110 | 0.022 | 0.245 | 3.69E-06 | 0.654 | 21.418 |
|  | rs11965579 | G | C | TRUE | 0.163 | -0.259 | 0.038 | 0.505 | 2.01E-05 | 0.608 | 18.181 |
|  | rs3860755 | G | C | TRUE | 0.056 | 0.048 | 0.012 | 0.159 | 1.66E-06 | 0.764 | 22.957 |
|  | rs4236095 | G | A | FALSE | 0.076 | 0.123 | 0.016 | 0.246 | 1.22E-06 | 0.618 | 23.542 |
|  | rs60884758 | C | T | FALSE | -0.070 | -0.016 | 0.014 | 0.195 | 7.82E-07 | 0.936 | 24.401 |
|  | rs6657302 | T | C | FALSE | -0.105 | -0.489 | 0.023 | 0.310 | 3.58E-06 | 0.115 | 21.479 |
|  | rs72893646 | A | T | TRUE | -0.072 | 0.201 | 0.016 | 0.220 | 4.93E-06 | 0.361 | 20.863 |
|  | rs7298818 | C | T | FALSE | 0.089 | -0.568 | 0.020 | 0.247 | 9.68E-06 | 0.021 | 19.574 |
| Genus Paraprevotella(id.962) | rs10842464 | T | C | FALSE | -0.076 | 0.016 | 0.017 | 0.162 | 1.11E-05 | 0.920 | 19.305 |
|  | rs145020347 | A | G | FALSE | -0.125 | -0.120 | 0.026 | 0.209 | 2.02E-06 | 0.567 | 22.579 |
|  | rs17109926 | A | G | FALSE | -0.099 | -0.032 | 0.022 | 0.168 | 4.83E-06 | 0.847 | 20.903 |
|  | rs17785622 | A | G | FALSE | 0.248 | 0.173 | 0.052 | 0.391 | 2.23E-06 | 0.658 | 22.385 |
|  | rs2081023 | A | G | FALSE | -0.123 | 0.281 | 0.024 | 0.213 | 2.19E-07 | 0.186 | 26.854 |
|  | rs3008582 | T | C | FALSE | 0.106 | -0.315 | 0.023 | 0.192 | 3.28E-06 | 0.101 | 21.643 |
|  | rs3801748 | G | A | FALSE | 0.078 | 0.028 | 0.017 | 0.154 | 5.59E-06 | 0.854 | 20.624 |
|  | rs4756632 | G | T | FALSE | -0.139 | -0.404 | 0.029 | 0.222 | 1.65E-06 | 0.069 | 22.959 |
|  | rs4767113 | C | T | FALSE | 0.088 | 0.051 | 0.018 | 0.159 | 1.58E-06 | 0.748 | 23.047 |
|  | rs58117850 | C | A | FALSE | -0.150 | -0.252 | 0.032 | 0.322 | 3.68E-06 | 0.434 | 21.427 |
|  | rs7240324 | T | G | FALSE | -0.102 | 0.035 | 0.023 | 0.172 | 6.57E-06 | 0.838 | 20.315 |
|  | rs9602779 | A | C | FALSE | -0.107 | 0.024 | 0.022 | 0.173 | 1.27E-06 | 0.889 | 23.463 |
|  | rs9900242 | A | G | FALSE | -0.085 | 0.196 | 0.018 | 0.156 | 1.13E-06 | 0.209 | 23.699 |
| Genus Parasutterella(id.2892) | rs10899911 | A | G | FALSE | -0.072 | 0.261 | 0.015 | 0.177 | 1.30E-06 | 0.139 | 23.429 |
|  | rs11715853 | G | A | FALSE | -0.066 | -0.352 | 0.015 | 0.163 | 5.70E-06 | 0.031 | 20.586 |
|  | rs1403396 | A | T | TRUE | -0.076 | 0.075 | 0.016 | 0.183 | 1.95E-06 | 0.683 | 22.641 |
|  | rs2090816 | A | C | FALSE | 0.084 | -0.133 | 0.018 | 0.192 | 2.11E-06 | 0.488 | 22.494 |
|  | rs35055552 | T | C | FALSE | 0.110 | 0.157 | 0.024 | 0.217 | 3.27E-06 | 0.471 | 21.653 |
|  | rs35414597 | T | A | TRUE | -0.068 | -0.197 | 0.014 | 0.162 | 1.44E-06 | 0.225 | 23.220 |
|  | rs55877868 | A | C | FALSE | -0.104 | 0.047 | 0.023 | 0.247 | 4.66E-06 | 0.848 | 20.974 |
|  | rs62273907 | A | G | FALSE | 0.229 | 0.256 | 0.050 | 0.298 | 4.91E-06 | 0.390 | 20.873 |
|  | rs6809952 | G | A | FALSE | -0.068 | -0.076 | 0.015 | 0.171 | 5.64E-06 | 0.657 | 20.606 |
|  | rs6828768 | C | T | FALSE | 0.064 | 0.232 | 0.013 | 0.149 | 1.58E-06 | 0.119 | 23.051 |
|  | rs7303158 | C | T | FALSE | 0.065 | -0.043 | 0.013 | 0.149 | 1.45E-06 | 0.772 | 23.214 |
|  | rs7311004 | T | C | FALSE | -0.062 | 0.016 | 0.014 | 0.150 | 6.01E-06 | 0.917 | 20.485 |
|  | rs7572229 | G | A | FALSE | 0.066 | -0.056 | 0.013 | 0.149 | 5.95E-07 | 0.705 | 24.929 |
|  | rs78383039 | T | C | FALSE | -0.146 | -0.063 | 0.030 | 0.377 | 8.46E-07 | 0.867 | 24.250 |
|  | rs8039785 | T | G | FALSE | 0.062 | 0.205 | 0.013 | 0.149 | 3.33E-06 | 0.167 | 21.615 |
|  | rs823424 | G | A | FALSE | -0.071 | -0.046 | 0.016 | 0.171 | 5.48E-06 | 0.790 | 20.661 |
| Genus Peptococcus(id.2037) | rs10031059 | T | C | FALSE | -0.121 | 0.036 | 0.023 | 0.175 | 8.09E-08 | 0.839 | 28.784 |
|  | rs57905414 | T | A | TRUE | 0.185 | 0.342 | 0.032 | 0.262 | 1.04E-08 | 0.192 | 32.770 |
|  | rs77681628 | C | T | FALSE | 0.200 | 0.358 | 0.039 | 0.273 | 2.32E-07 | 0.190 | 26.745 |
| Genus Phascolarctobacterium(id.2168) | rs11929846 | T | C | FALSE | -0.070 | -0.029 | 0.016 | 0.181 | 1.02E-05 | 0.872 | 19.479 |
|  | rs12618201 | A | G | FALSE | 0.064 | -0.140 | 0.014 | 0.150 | 3.44E-06 | 0.350 | 21.556 |
|  | rs1264476 | T | G | FALSE | 0.077 | -0.371 | 0.017 | 0.190 | 3.81E-06 | 0.051 | 21.357 |
|  | rs130483 | A | G | FALSE | 0.066 | 0.018 | 0.014 | 0.152 | 5.07E-06 | 0.906 | 20.810 |
|  | rs28525131 | G | A | FALSE | -0.119 | 0.424 | 0.027 | 0.345 | 1.03E-05 | 0.219 | 19.450 |
|  | rs56069061 | G | A | FALSE | -0.111 | 0.542 | 0.023 | 0.296 | 1.40E-06 | 0.067 | 23.279 |
|  | rs56157888 | A | C | FALSE | 0.095 | -0.019 | 0.019 | 0.180 | 8.54E-07 | 0.915 | 24.232 |
|  | rs6427992 | G | C | TRUE | -0.065 | -0.059 | 0.014 | 0.154 | 2.09E-06 | 0.701 | 22.514 |
|  | rs74540770 | G | A | FALSE | -0.121 | 0.319 | 0.026 | 0.274 | 2.88E-06 | 0.245 | 21.895 |
|  | rs75882962 | T | C | FALSE | 0.097 | -0.043 | 0.019 | 0.225 | 3.72E-07 | 0.850 | 25.835 |
|  | rs76124218 | C | G | TRUE | -0.159 | -0.563 | 0.034 | 0.402 | 3.74E-06 | 0.162 | 21.395 |
|  | rs7982713 | G | A | FALSE | 0.073 | -0.013 | 0.016 | 0.166 | 8.42E-06 | 0.938 | 19.839 |
| Genus Prevotella7(id.11182) | rs11035469 | A | G | FALSE | -0.144 | 0.231 | 0.031 | 0.176 | 3.80E-06 | 0.188 | 21.365 |
|  | rs118038478 | A | G | FALSE | 0.206 | -0.403 | 0.047 | 0.291 | 1.15E-05 | 0.166 | 19.239 |
|  | rs12124567 | A | G | FALSE | -0.121 | -0.022 | 0.028 | 0.187 | 1.03E-05 | 0.905 | 19.448 |
|  | rs12195431 | T | C | FALSE | 0.197 | -0.022 | 0.044 | 0.255 | 8.87E-06 | 0.931 | 19.741 |
|  | rs16937247 | G | C | TRUE | 0.146 | -0.008 | 0.035 | 0.188 | 3.24E-05 | 0.966 | 17.273 |
|  | rs2240542 | C | T | FALSE | 0.121 | -0.185 | 0.026 | 0.169 | 3.90E-06 | 0.271 | 21.312 |
|  | rs2918132 | C | T | FALSE | -0.115 | 0.340 | 0.025 | 0.155 | 6.88E-06 | 0.028 | 20.228 |
|  | rs385483 | A | G | FALSE | 0.137 | -0.272 | 0.029 | 0.184 | 2.67E-06 | 0.139 | 22.038 |
|  | rs57404562 | C | A | FALSE | 0.155 | -0.049 | 0.032 | 0.220 | 8.67E-07 | 0.826 | 24.202 |
|  | rs9426434 | T | C | FALSE | -0.124 | 0.019 | 0.028 | 0.157 | 9.00E-06 | 0.903 | 19.713 |
|  | rs9608249 | A | G | FALSE | -0.158 | 0.162 | 0.034 | 0.233 | 2.55E-06 | 0.486 | 22.130 |
|  | rs9959718 | G | A | FALSE | 0.133 | -0.179 | 0.028 | 0.184 | 1.36E-06 | 0.330 | 23.333 |
| Genus Prevotella9(id.11183) | rs10512344 | C | G | TRUE | 0.247 | -0.425 | 0.054 | 0.547 | 5.45E-06 | 0.437 | 20.671 |
|  | rs111509883 | T | C | FALSE | 0.171 | 0.211 | 0.035 | 0.244 | 8.53E-07 | 0.387 | 24.235 |
|  | rs11199734 | A | T | TRUE | 0.077 | -0.123 | 0.017 | 0.180 | 5.49E-06 | 0.495 | 20.657 |
|  | rs11685699 | C | T | FALSE | -0.141 | 0.030 | 0.030 | 0.284 | 1.74E-06 | 0.916 | 22.858 |
|  | rs117271932 | A | G | FALSE | 0.208 | 0.697 | 0.044 | 0.321 | 2.30E-06 | 0.030 | 22.326 |
|  | rs12648235 | T | C | FALSE | 0.079 | 0.027 | 0.018 | 0.177 | 9.72E-06 | 0.879 | 19.565 |
|  | rs1304512 | G | A | FALSE | 0.076 | -0.086 | 0.017 | 0.168 | 4.41E-06 | 0.609 | 21.078 |
|  | rs16966465 | G | C | TRUE | 0.074 | -0.393 | 0.017 | 0.192 | 6.88E-06 | 0.040 | 20.225 |
|  | rs2104588 | T | C | FALSE | 0.106 | 0.056 | 0.024 | 0.319 | 8.98E-06 | 0.860 | 19.716 |
|  | rs2495052 | A | G | FALSE | 0.084 | 0.041 | 0.019 | 0.211 | 8.58E-06 | 0.847 | 19.805 |
|  | rs2683313 | A | G | FALSE | -0.072 | 0.012 | 0.015 | 0.160 | 1.76E-06 | 0.939 | 22.843 |
|  | rs4968431 | G | T | FALSE | 0.064 | 0.249 | 0.014 | 0.156 | 8.99E-06 | 0.111 | 19.716 |
|  | rs7232121 | G | C | TRUE | 0.067 | 0.035 | 0.014 | 0.151 | 3.25E-06 | 0.817 | 21.663 |
|  | rs7237249 | C | T | FALSE | -0.082 | 0.081 | 0.018 | 0.189 | 6.10E-06 | 0.669 | 20.458 |
|  | rs72815774 | T | C | FALSE | -0.176 | 0.063 | 0.039 | 0.332 | 7.38E-06 | 0.850 | 20.091 |
|  | rs746764 | T | C | FALSE | -0.092 | 0.121 | 0.019 | 0.182 | 2.15E-06 | 0.506 | 22.457 |
|  | rs7976209 | T | C | FALSE | -0.087 | 0.116 | 0.020 | 0.205 | 1.09E-05 | 0.571 | 19.351 |
|  | rs9428102 | A | G | FALSE | -0.078 | 0.102 | 0.018 | 0.179 | 9.71E-06 | 0.571 | 19.568 |
|  | rs9613013 | G | A | FALSE | 0.092 | -0.265 | 0.020 | 0.228 | 5.99E-06 | 0.245 | 20.493 |
| Genus RikenellaceaeRC9gutgroup(id.11191) | rs12501673 | A | G | FALSE | 0.116 | -0.391 | 0.026 | 0.169 | 9.07E-06 | 0.020 | 19.698 |
|  | rs17032291 | T | C | FALSE | -0.170 | 0.349 | 0.037 | 0.225 | 4.13E-06 | 0.121 | 21.206 |
|  | rs17582787 | A | G | FALSE | -0.158 | 0.163 | 0.034 | 0.200 | 3.48E-06 | 0.415 | 21.534 |
|  | rs2074881 | T | C | FALSE | -0.142 | 0.033 | 0.032 | 0.222 | 1.13E-05 | 0.883 | 19.283 |
|  | rs2900503 | G | T | FALSE | -0.172 | 0.081 | 0.033 | 0.201 | 1.33E-07 | 0.688 | 27.829 |
|  | rs2998141 | T | C | FALSE | -0.136 | -0.154 | 0.029 | 0.176 | 3.28E-06 | 0.380 | 21.644 |
|  | rs4270579 | G | A | FALSE | -0.118 | 0.105 | 0.027 | 0.160 | 1.33E-05 | 0.511 | 18.960 |
|  | rs4717843 | G | T | FALSE | -0.119 | -0.302 | 0.026 | 0.150 | 4.61E-06 | 0.044 | 20.992 |
|  | rs7113155 | G | C | TRUE | 0.114 | 0.058 | 0.025 | 0.154 | 3.88E-06 | 0.707 | 21.322 |
|  | rs7193937 | G | C | TRUE | 0.124 | 0.007 | 0.028 | 0.157 | 7.63E-06 | 0.965 | 20.030 |
|  | rs7712231 | A | G | FALSE | 0.156 | -0.522 | 0.035 | 0.221 | 8.22E-06 | 0.018 | 19.886 |
|  | rs80309088 | G | A | FALSE | 0.174 | -0.419 | 0.038 | 0.228 | 5.64E-06 | 0.066 | 20.606 |
|  | rs9887954 | G | A | FALSE | -0.115 | 0.187 | 0.025 | 0.152 | 3.98E-06 | 0.219 | 21.272 |
| Genus Romboutsia(id.11347) | rs10279978 | A | G | FALSE | -0.062 | -0.035 | 0.013 | 0.161 | 1.10E-06 | 0.829 | 23.749 |
|  | rs11221428 | T | C | FALSE | -0.073 | 0.008 | 0.016 | 0.176 | 4.39E-06 | 0.966 | 21.088 |
|  | rs114398731 | G | C | TRUE | -0.131 | 0.102 | 0.029 | 0.331 | 8.20E-06 | 0.758 | 19.891 |
|  | rs16843578 | C | T | FALSE | -0.088 | 0.030 | 0.020 | 0.331 | 8.67E-06 | 0.929 | 19.785 |
|  | rs28603357 | T | C | FALSE | -0.215 | 1.197 | 0.047 | 0.525 | 5.98E-06 | 0.023 | 20.493 |
|  | rs34302036 | A | G | FALSE | 0.055 | -0.121 | 0.012 | 0.151 | 5.27E-06 | 0.420 | 20.737 |
|  | rs61841503 | G | A | FALSE | 0.093 | 0.337 | 0.017 | 0.221 | 6.04E-08 | 0.127 | 29.351 |
|  | rs62504452 | A | G | FALSE | -0.071 | -0.108 | 0.016 | 0.215 | 5.83E-06 | 0.616 | 20.544 |
|  | rs7109293 | A | G | FALSE | 0.092 | -0.155 | 0.021 | 0.234 | 7.92E-06 | 0.509 | 19.957 |
|  | rs75200530 | T | G | FALSE | -0.191 | -0.445 | 0.042 | 0.441 | 5.96E-06 | 0.312 | 20.500 |
|  | rs75987356 | G | A | FALSE | -0.130 | 0.257 | 0.028 | 0.280 | 3.83E-06 | 0.358 | 21.347 |
|  | rs77702691 | A | G | FALSE | -0.094 | -0.176 | 0.021 | 0.260 | 5.99E-06 | 0.498 | 20.493 |
|  | rs9389266 | T | G | FALSE | 0.072 | 0.051 | 0.016 | 0.196 | 8.50E-06 | 0.793 | 19.821 |
|  | rs9567264 | C | T | FALSE | 0.058 | 0.004 | 0.013 | 0.157 | 5.38E-06 | 0.981 | 20.697 |
| Genus Roseburia(id.2012) | rs116270582 | T | A | TRUE | -0.154 | -0.627 | 0.033 | 0.380 | 2.99E-06 | 0.099 | 21.823 |
|  | rs12740451 | T | C | FALSE | 0.070 | -0.123 | 0.015 | 0.218 | 5.60E-06 | 0.571 | 20.620 |
|  | rs147990086 | A | G | FALSE | -0.058 | 0.203 | 0.013 | 0.198 | 1.23E-05 | 0.306 | 19.120 |
|  | rs16910295 | T | C | FALSE | -0.098 | 0.004 | 0.021 | 0.330 | 2.89E-06 | 0.990 | 21.887 |
|  | rs2034589 | G | C | TRUE | 0.063 | 0.011 | 0.012 | 0.194 | 3.40E-07 | 0.955 | 26.008 |
|  | rs2160994 | T | C | FALSE | 0.055 | -0.089 | 0.011 | 0.158 | 9.79E-07 | 0.573 | 23.969 |
|  | rs2943022 | T | C | FALSE | 0.049 | 0.189 | 0.011 | 0.151 | 3.75E-06 | 0.210 | 21.391 |
|  | rs302266 | T | C | FALSE | -0.078 | 0.007 | 0.017 | 0.224 | 7.01E-06 | 0.976 | 20.192 |
|  | rs329182 | T | C | FALSE | 0.069 | 0.007 | 0.015 | 0.203 | 6.32E-06 | 0.973 | 20.389 |
|  | rs4748237 | G | C | TRUE | 0.049 | -0.011 | 0.011 | 0.150 | 4.45E-06 | 0.941 | 21.062 |
|  | rs55858165 | A | C | FALSE | 0.179 | -0.454 | 0.040 | 0.392 | 9.54E-06 | 0.246 | 19.601 |
|  | rs57466170 | C | T | FALSE | 0.074 | 0.167 | 0.017 | 0.281 | 1.56E-05 | 0.552 | 18.667 |
|  | rs6445851 | G | A | FALSE | -0.050 | -0.141 | 0.011 | 0.154 | 4.26E-06 | 0.359 | 21.144 |
|  | rs6930661 | C | T | FALSE | -0.096 | 0.166 | 0.020 | 0.316 | 2.72E-06 | 0.600 | 22.008 |
|  | rs75326254 | C | T | FALSE | -0.105 | 0.434 | 0.023 | 0.313 | 5.86E-06 | 0.166 | 20.533 |
|  | rs78753150 | A | C | FALSE | 0.097 | -0.168 | 0.021 | 0.251 | 6.03E-06 | 0.503 | 20.479 |
|  | rs9300744 | C | T | FALSE | -0.059 | 0.064 | 0.013 | 0.194 | 3.13E-06 | 0.741 | 21.733 |
| Genus Ruminiclostridium5(id.11355) | rs10827477 | A | G | FALSE | -0.055 | -0.015 | 0.012 | 0.156 | 2.00E-06 | 0.923 | 22.592 |
|  | rs113753996 | T | C | FALSE | 0.082 | -0.406 | 0.017 | 0.193 | 2.55E-06 | 0.035 | 22.129 |
|  | rs1223978 | T | C | FALSE | 0.048 | -0.047 | 0.011 | 0.150 | 7.73E-06 | 0.752 | 20.003 |
|  | rs1492620 | T | C | FALSE | -0.083 | -0.199 | 0.018 | 0.224 | 3.99E-06 | 0.374 | 21.271 |
|  | rs2286384 | G | C | TRUE | -0.052 | 0.069 | 0.011 | 0.148 | 1.39E-06 | 0.640 | 23.300 |
|  | rs243585 | C | G | TRUE | -0.059 | 0.122 | 0.012 | 0.168 | 1.29E-06 | 0.466 | 23.443 |
|  | rs2482038 | C | A | FALSE | 0.052 | 0.079 | 0.011 | 0.152 | 1.83E-06 | 0.601 | 22.764 |
|  | rs2791343 | T | C | FALSE | 0.052 | -0.152 | 0.011 | 0.152 | 5.07E-06 | 0.316 | 20.810 |
|  | rs2801960 | C | G | TRUE | 0.052 | -0.124 | 0.012 | 0.162 | 6.02E-06 | 0.444 | 20.483 |
|  | rs2833828 | G | A | FALSE | 0.049 | 0.187 | 0.011 | 0.151 | 6.66E-06 | 0.215 | 20.288 |
|  | rs4955951 | A | G | FALSE | -0.071 | 0.129 | 0.017 | 0.230 | 1.68E-05 | 0.577 | 18.526 |
|  | rs6121460 | G | A | FALSE | 0.093 | -0.162 | 0.020 | 0.280 | 2.82E-06 | 0.563 | 21.934 |
|  | rs73002572 | G | C | TRUE | 0.182 | -0.012 | 0.041 | 0.237 | 1.16E-05 | 0.961 | 19.223 |
|  | rs79968837 | A | G | FALSE | -0.095 | 0.068 | 0.019 | 0.334 | 9.06E-07 | 0.838 | 24.118 |
|  | rs8053158 | A | G | FALSE | -0.074 | -0.074 | 0.016 | 0.230 | 3.27E-06 | 0.749 | 21.651 |
| Genus Ruminiclostridium6(id.11356) | rs10829821 | T | C | FALSE | -0.098 | 0.116 | 0.022 | 0.261 | 6.26E-06 | 0.657 | 20.406 |
|  | rs116969552 | A | G | FALSE | -0.167 | -0.451 | 0.038 | 0.446 | 9.48E-06 | 0.312 | 19.614 |
|  | rs11992182 | A | C | FALSE | 0.063 | 0.066 | 0.014 | 0.178 | 5.75E-06 | 0.709 | 20.568 |
|  | rs12362316 | A | G | FALSE | 0.074 | -0.060 | 0.015 | 0.176 | 1.48E-06 | 0.735 | 23.174 |
|  | rs1871858 | C | G | TRUE | -0.105 | -0.168 | 0.024 | 0.238 | 8.87E-06 | 0.480 | 19.740 |
|  | rs2548459 | C | T | FALSE | 0.055 | -0.100 | 0.012 | 0.151 | 6.18E-06 | 0.508 | 20.432 |
|  | rs35362464 | C | A | FALSE | 0.072 | -0.013 | 0.017 | 0.215 | 1.34E-05 | 0.951 | 18.956 |
|  | rs56212330 | A | G | FALSE | 0.059 | -0.113 | 0.013 | 0.166 | 8.68E-06 | 0.496 | 19.783 |
|  | rs589368 | G | A | FALSE | -0.147 | -0.547 | 0.033 | 0.409 | 8.81E-06 | 0.181 | 19.753 |
|  | rs61060922 | T | G | FALSE | 0.159 | 0.971 | 0.032 | 0.413 | 7.91E-07 | 0.019 | 24.380 |
|  | rs67479537 | T | C | FALSE | 0.119 | -0.435 | 0.026 | 0.351 | 7.04E-06 | 0.216 | 20.183 |
|  | rs71414120 | T | G | FALSE | 0.201 | 0.221 | 0.041 | 0.343 | 7.70E-07 | 0.518 | 24.432 |
|  | rs72991535 | T | G | FALSE | 0.136 | 0.301 | 0.030 | 0.406 | 4.35E-06 | 0.459 | 21.105 |
|  | rs792058 | G | A | FALSE | 0.055 | -0.026 | 0.013 | 0.151 | 9.92E-06 | 0.865 | 19.527 |
|  | rs79968172 | G | A | FALSE | 0.116 | 0.118 | 0.024 | 0.314 | 1.77E-06 | 0.707 | 22.827 |
|  | rs9555756 | A | C | FALSE | -0.080 | 0.383 | 0.018 | 0.266 | 5.34E-06 | 0.150 | 20.712 |
| Genus Ruminiclostridium9(id.11357) | rs113048721 | C | G | TRUE | 0.060 | 0.081 | 0.013 | 0.209 | 6.02E-06 | 0.700 | 20.482 |
|  | rs12040548 | G | T | FALSE | 0.057 | 0.011 | 0.012 | 0.166 | 3.13E-06 | 0.946 | 21.733 |
|  | rs12419854 | T | A | TRUE | -0.073 | 0.001 | 0.016 | 0.169 | 2.89E-06 | 0.997 | 21.890 |
|  | rs13033315 | T | A | TRUE | 0.051 | -0.226 | 0.011 | 0.159 | 4.47E-06 | 0.154 | 21.051 |
|  | rs57665991 | C | G | TRUE | -0.064 | -0.106 | 0.012 | 0.172 | 1.98E-07 | 0.538 | 27.052 |
|  | rs6082461 | A | C | FALSE | 0.059 | -0.122 | 0.013 | 0.184 | 7.59E-06 | 0.506 | 20.038 |
|  | rs7137760 | C | T | FALSE | 0.051 | 0.019 | 0.011 | 0.149 | 5.95E-06 | 0.900 | 20.503 |
|  | rs73592673 | A | T | TRUE | -0.082 | -0.090 | 0.017 | 0.223 | 1.47E-06 | 0.687 | 23.189 |
|  | rs74303178 | T | C | FALSE | 0.053 | -0.192 | 0.012 | 0.160 | 7.95E-06 | 0.231 | 19.950 |
|  | rs78191726 | T | C | FALSE | 0.094 | -0.075 | 0.021 | 0.289 | 6.94E-06 | 0.794 | 20.210 |
|  | rs79082720 | C | G | TRUE | 0.093 | -0.059 | 0.020 | 0.346 | 5.83E-06 | 0.864 | 20.543 |
|  | rs918449 | A | G | FALSE | -0.095 | -0.654 | 0.020 | 0.290 | 1.42E-06 | 0.024 | 23.258 |
|  | rs9522712 | T | C | FALSE | 0.070 | -0.165 | 0.015 | 0.212 | 6.30E-06 | 0.436 | 20.396 |
|  | rs9809789 | C | T | FALSE | -0.072 | 0.014 | 0.016 | 0.194 | 7.01E-06 | 0.943 | 20.189 |
| Genus RuminococcaceaeNK4A214group(id.11358) | rs11241747 | C | T | FALSE | 0.053 | -0.111 | 0.012 | 0.163 | 8.69E-06 | 0.495 | 19.781 |
|  | rs114244418 | C | G | TRUE | -0.175 | 0.122 | 0.037 | 0.382 | 2.58E-06 | 0.751 | 22.105 |
|  | rs11586410 | G | A | FALSE | -0.086 | -0.051 | 0.017 | 0.207 | 3.76E-07 | 0.805 | 25.815 |
|  | rs12642039 | T | C | FALSE | -0.055 | 0.148 | 0.012 | 0.155 | 3.63E-06 | 0.338 | 21.452 |
|  | rs12731 | A | G | FALSE | -0.053 | -0.058 | 0.012 | 0.153 | 4.51E-06 | 0.703 | 21.035 |
|  | rs13087692 | T | G | FALSE | 0.057 | 0.183 | 0.013 | 0.162 | 5.05E-06 | 0.259 | 20.818 |
|  | rs136761 | G | A | FALSE | -0.059 | -0.046 | 0.012 | 0.155 | 8.19E-07 | 0.768 | 24.312 |
|  | rs147475196 | A | G | FALSE | -0.134 | 0.400 | 0.030 | 0.244 | 5.86E-06 | 0.101 | 20.535 |
|  | rs34576931 | G | C | TRUE | -0.087 | 0.166 | 0.019 | 0.291 | 7.42E-06 | 0.568 | 20.081 |
|  | rs35559912 | T | C | FALSE | -0.093 | -0.054 | 0.020 | 0.230 | 5.57E-06 | 0.816 | 20.629 |
|  | rs4814689 | C | T | FALSE | -0.108 | 0.284 | 0.023 | 0.365 | 2.68E-06 | 0.436 | 22.036 |
|  | rs5994253 | A | G | FALSE | -0.081 | -0.174 | 0.016 | 0.214 | 2.64E-07 | 0.416 | 26.493 |
|  | rs62027366 | T | C | FALSE | 0.062 | 0.081 | 0.014 | 0.186 | 7.75E-06 | 0.664 | 19.998 |
|  | rs6681678 | C | T | FALSE | -0.100 | -0.147 | 0.024 | 0.411 | 2.99E-05 | 0.721 | 17.422 |
|  | rs73158814 | C | G | TRUE | -0.109 | -0.217 | 0.023 | 0.399 | 1.56E-06 | 0.586 | 23.073 |
|  | rs7573569 | T | C | FALSE | 0.108 | -0.313 | 0.023 | 0.307 | 4.00E-06 | 0.308 | 21.264 |
| Genus RuminococcaceaeUCG002(id.11360) | rs10916131 | C | T | FALSE | -0.069 | -0.009 | 0.015 | 0.200 | 2.31E-06 | 0.964 | 22.321 |
|  | rs10927423 | C | A | FALSE | -0.071 | 0.251 | 0.015 | 0.194 | 1.36E-06 | 0.195 | 23.340 |
|  | rs10964441 | G | A | FALSE | -0.149 | 0.539 | 0.034 | 0.247 | 1.54E-05 | 0.029 | 18.683 |
|  | rs113147300 | A | G | FALSE | -0.076 | -0.135 | 0.016 | 0.215 | 4.05E-06 | 0.529 | 21.240 |
|  | rs11607472 | A | G | FALSE | -0.078 | -0.191 | 0.018 | 0.297 | 9.65E-06 | 0.521 | 19.580 |
|  | rs116974815 | C | A | FALSE | -0.190 | -0.057 | 0.040 | 0.298 | 1.72E-06 | 0.849 | 22.890 |
|  | rs12463378 | A | G | FALSE | -0.052 | 0.134 | 0.011 | 0.162 | 3.20E-06 | 0.409 | 21.694 |
|  | rs15256 | C | T | FALSE | 0.073 | 0.237 | 0.017 | 0.230 | 1.36E-05 | 0.303 | 18.928 |
|  | rs2265670 | C | G | TRUE | -0.051 | 0.270 | 0.011 | 0.149 | 2.75E-06 | 0.069 | 21.983 |
|  | rs362417 | G | C | TRUE | -0.055 | -0.095 | 0.012 | 0.181 | 5.77E-06 | 0.601 | 20.564 |
|  | rs55793120 | T | C | FALSE | 0.137 | -0.283 | 0.027 | 0.315 | 5.39E-07 | 0.370 | 25.119 |
|  | rs56030423 | G | A | FALSE | -0.098 | 0.307 | 0.022 | 0.289 | 5.54E-06 | 0.288 | 20.640 |
|  | rs57079348 | T | G | FALSE | -0.077 | 0.350 | 0.017 | 0.319 | 9.39E-06 | 0.272 | 19.632 |
|  | rs62374283 | T | C | FALSE | -0.058 | 0.125 | 0.012 | 0.155 | 1.45E-06 | 0.420 | 23.215 |
|  | rs6542556 | A | G | FALSE | 0.051 | -0.127 | 0.011 | 0.153 | 7.86E-06 | 0.409 | 19.972 |
|  | rs67746927 | C | G | TRUE | -0.054 | 0.063 | 0.011 | 0.151 | 8.89E-07 | 0.678 | 24.154 |
|  | rs6793778 | C | T | FALSE | -0.056 | -0.067 | 0.013 | 0.170 | 8.18E-06 | 0.695 | 19.896 |
|  | rs7120052 | A | C | FALSE | 0.062 | 0.271 | 0.014 | 0.189 | 4.02E-06 | 0.152 | 21.254 |
|  | rs7155595 | C | A | FALSE | 0.057 | -0.105 | 0.012 | 0.162 | 1.11E-06 | 0.519 | 23.734 |
|  | rs7249614 | A | G | FALSE | -0.049 | -0.114 | 0.011 | 0.153 | 8.70E-06 | 0.456 | 19.777 |
|  | rs72874194 | G | C | TRUE | -0.077 | -0.163 | 0.017 | 0.218 | 4.11E-06 | 0.456 | 21.212 |
|  | rs7342369 | C | A | FALSE | -0.053 | 0.081 | 0.012 | 0.171 | 5.39E-06 | 0.636 | 20.694 |
|  | rs76847269 | A | G | FALSE | 0.164 | 0.757 | 0.036 | 0.473 | 4.41E-06 | 0.110 | 21.077 |
|  | rs77564310 | A | C | FALSE | -0.071 | -0.143 | 0.014 | 0.184 | 4.14E-07 | 0.438 | 25.630 |
|  | rs79016051 | C | T | FALSE | -0.089 | 0.051 | 0.019 | 0.221 | 2.78E-06 | 0.816 | 21.964 |
|  | rs882348 | A | G | FALSE | -0.080 | -0.232 | 0.018 | 0.230 | 7.52E-06 | 0.312 | 20.057 |
| Genus RuminococcaceaeUCG003(id.11361) | rs10490280 | C | T | FALSE | -0.067 | 0.172 | 0.014 | 0.190 | 2.73E-06 | 0.365 | 21.994 |
|  | rs11243416 | T | C | FALSE | -0.093 | 0.101 | 0.019 | 0.293 | 1.29E-06 | 0.732 | 23.437 |
|  | rs11831525 | A | C | FALSE | 0.071 | -0.142 | 0.015 | 0.180 | 2.71E-06 | 0.429 | 22.012 |
|  | rs139730 | G | C | TRUE | -0.058 | -0.232 | 0.013 | 0.173 | 9.80E-06 | 0.181 | 19.550 |
|  | rs16959793 | A | C | FALSE | -0.063 | -0.086 | 0.013 | 0.151 | 1.90E-06 | 0.572 | 22.692 |
|  | rs2523124 | T | C | FALSE | -0.055 | -0.040 | 0.012 | 0.151 | 6.06E-06 | 0.793 | 20.468 |
|  | rs3013089 | G | A | FALSE | -0.055 | 0.027 | 0.012 | 0.154 | 4.60E-06 | 0.863 | 20.998 |
|  | rs4452755 | A | C | FALSE | -0.063 | -0.033 | 0.013 | 0.157 | 2.49E-06 | 0.836 | 22.172 |
|  | rs4532474 | G | A | FALSE | 0.077 | -0.162 | 0.017 | 0.200 | 6.39E-06 | 0.418 | 20.367 |
|  | rs4629039 | T | A | TRUE | 0.055 | -0.280 | 0.012 | 0.153 | 6.35E-06 | 0.067 | 20.380 |
|  | rs646327 | G | A | FALSE | 0.059 | -0.097 | 0.012 | 0.151 | 7.18E-07 | 0.520 | 24.567 |
|  | rs6759615 | A | G | FALSE | 0.103 | -0.130 | 0.020 | 0.248 | 3.04E-07 | 0.600 | 26.224 |
|  | rs73341548 | T | G | FALSE | -0.170 | -0.065 | 0.032 | 0.317 | 9.50E-08 | 0.838 | 28.473 |
|  | rs78720113 | A | G | FALSE | -0.115 | 0.041 | 0.025 | 0.278 | 3.81E-06 | 0.883 | 21.357 |
| Genus RuminococcaceaeUCG004(id.11362) | rs10976229 | T | G | FALSE | 0.096 | -0.060 | 0.021 | 0.223 | 7.70E-06 | 0.788 | 20.010 |
|  | rs11961899 | G | A | FALSE | -0.071 | -0.110 | 0.016 | 0.165 | 1.15E-05 | 0.503 | 19.237 |
|  | rs12125734 | G | T | FALSE | 0.134 | -0.345 | 0.026 | 0.258 | 1.95E-07 | 0.182 | 27.086 |
|  | rs2248146 | T | C | FALSE | 0.069 | -0.250 | 0.015 | 0.156 | 7.22E-06 | 0.110 | 20.135 |
|  | rs3800178 | C | T | FALSE | -0.080 | 0.118 | 0.018 | 0.171 | 6.15E-06 | 0.490 | 20.442 |
|  | rs516741 | T | G | FALSE | -0.076 | 0.073 | 0.016 | 0.190 | 3.09E-06 | 0.703 | 21.757 |
|  | rs550351 | A | C | FALSE | 0.079 | -0.021 | 0.018 | 0.150 | 1.27E-05 | 0.888 | 19.055 |
|  | rs6769553 | A | G | FALSE | 0.085 | 0.114 | 0.016 | 0.169 | 6.78E-08 | 0.501 | 29.126 |
|  | rs7123615 | C | G | TRUE | -0.079 | -0.009 | 0.018 | 0.186 | 1.20E-05 | 0.961 | 19.162 |
|  | rs7569771 | A | G | FALSE | -0.076 | -0.010 | 0.017 | 0.173 | 8.29E-06 | 0.956 | 19.869 |
|  | rs872501 | G | A | FALSE | 0.116 | -0.282 | 0.026 | 0.284 | 7.62E-06 | 0.322 | 20.031 |
|  | rs9818949 | G | T | FALSE | 0.086 | -0.224 | 0.019 | 0.189 | 5.17E-06 | 0.236 | 20.774 |
| Genus RuminococcaceaeUCG005(id.11363) | rs10873449 | T | C | FALSE | 0.065 | 0.121 | 0.014 | 0.189 | 5.41E-06 | 0.525 | 20.688 |
|  | rs10937802 | G | A | FALSE | 0.076 | -0.004 | 0.017 | 0.229 | 7.03E-06 | 0.988 | 20.185 |
|  | rs10950694 | T | C | FALSE | 0.058 | -0.048 | 0.011 | 0.155 | 4.16E-07 | 0.755 | 25.620 |
|  | rs114279581 | A | G | FALSE | -0.147 | 0.178 | 0.032 | 0.279 | 3.50E-06 | 0.523 | 21.519 |
|  | rs12288512 | A | G | FALSE | 0.067 | -0.031 | 0.014 | 0.175 | 3.91E-06 | 0.858 | 21.308 |
|  | rs12604884 | G | T | FALSE | 0.068 | -0.282 | 0.014 | 0.192 | 2.63E-06 | 0.142 | 22.071 |
|  | rs2893871 | G | A | FALSE | -0.074 | -0.183 | 0.016 | 0.237 | 2.17E-06 | 0.441 | 22.435 |
|  | rs34781347 | G | A | FALSE | 0.189 | 0.131 | 0.039 | 0.294 | 1.05E-06 | 0.657 | 23.835 |
|  | rs35166120 | C | G | TRUE | -0.069 | 0.024 | 0.015 | 0.181 | 2.71E-06 | 0.896 | 22.011 |
|  | rs394449 | A | T | TRUE | 0.069 | -0.133 | 0.015 | 0.219 | 3.17E-06 | 0.543 | 21.710 |
|  | rs55793120 | T | C | FALSE | 0.122 | -0.283 | 0.028 | 0.315 | 1.37E-05 | 0.370 | 18.905 |
|  | rs60081663 | C | G | TRUE | 0.158 | 0.229 | 0.032 | 0.413 | 7.56E-07 | 0.579 | 24.467 |
|  | rs72776570 | C | A | FALSE | 0.087 | 0.093 | 0.020 | 0.253 | 1.01E-05 | 0.713 | 19.498 |
|  | rs7449320 | C | A | FALSE | 0.060 | 0.137 | 0.013 | 0.176 | 4.66E-06 | 0.437 | 20.972 |
|  | rs7555878 | A | G | FALSE | 0.059 | 0.007 | 0.013 | 0.173 | 2.83E-06 | 0.969 | 21.931 |
|  | rs7586445 | G | A | FALSE | 0.078 | -0.117 | 0.018 | 0.222 | 9.27E-06 | 0.598 | 19.657 |
|  | rs898577 | T | C | FALSE | -0.123 | 0.371 | 0.029 | 0.325 | 1.78E-05 | 0.254 | 18.413 |
| Genus RuminococcaceaeUCG009(id.11366) | rs113006825 | T | C | FALSE | -0.093 | -0.024 | 0.021 | 0.182 | 8.00E-06 | 0.897 | 19.937 |
|  | rs12508214 | C | T | FALSE | -0.077 | -0.064 | 0.017 | 0.158 | 4.53E-06 | 0.685 | 21.026 |
|  | rs138460696 | A | G | FALSE | 0.139 | -0.542 | 0.032 | 0.285 | 1.03E-05 | 0.057 | 19.451 |
|  | rs1550196 | G | A | FALSE | 0.131 | 0.205 | 0.026 | 0.251 | 6.20E-07 | 0.413 | 24.849 |
|  | rs2058609 | A | G | FALSE | 0.082 | -0.281 | 0.017 | 0.167 | 2.97E-06 | 0.091 | 21.837 |
|  | rs2192926 | A | G | FALSE | -0.089 | 0.066 | 0.019 | 0.158 | 3.95E-06 | 0.676 | 21.290 |
|  | rs4079028 | C | T | FALSE | 0.092 | 0.154 | 0.020 | 0.173 | 4.37E-06 | 0.374 | 21.094 |
|  | rs4708333 | T | G | FALSE | -0.084 | 0.007 | 0.017 | 0.157 | 1.52E-06 | 0.964 | 23.122 |
|  | rs61779334 | G | C | TRUE | -0.138 | -0.310 | 0.029 | 0.250 | 2.25E-06 | 0.215 | 22.369 |
|  | rs6952765 | G | A | FALSE | 0.073 | 0.049 | 0.017 | 0.160 | 1.14E-05 | 0.758 | 19.269 |
|  | rs758191 | T | G | FALSE | 0.177 | 0.195 | 0.038 | 0.252 | 2.37E-06 | 0.441 | 22.270 |
|  | rs78410648 | A | G | FALSE | 0.121 | -0.020 | 0.028 | 0.241 | 1.28E-05 | 0.934 | 19.034 |
|  | rs8009993 | G | C | TRUE | -0.136 | 0.088 | 0.024 | 0.209 | 2.83E-08 | 0.675 | 30.821 |
|  | rs9558661 | T | C | FALSE | -0.090 | -0.021 | 0.020 | 0.187 | 7.73E-06 | 0.912 | 20.004 |
| Genus RuminococcaceaeUCG010(id.11367) | rs12597105 | G | A | FALSE | 0.067 | -0.096 | 0.014 | 0.190 | 3.40E-06 | 0.611 | 21.579 |
|  | rs2820282 | A | C | FALSE | -0.059 | -0.175 | 0.013 | 0.153 | 2.55E-06 | 0.252 | 22.127 |
|  | rs35506912 | G | C | TRUE | -0.069 | 0.265 | 0.015 | 0.185 | 2.73E-06 | 0.152 | 21.994 |
|  | rs682403 | A | G | FALSE | -0.059 | 0.162 | 0.012 | 0.149 | 2.39E-06 | 0.275 | 22.257 |
|  | rs6958419 | C | T | FALSE | -0.059 | -0.008 | 0.012 | 0.149 | 2.79E-06 | 0.960 | 21.958 |
|  | rs73218807 | G | A | FALSE | -0.166 | 0.369 | 0.037 | 0.271 | 6.26E-06 | 0.174 | 20.406 |
|  | rs7441445 | C | T | FALSE | -0.057 | 0.018 | 0.013 | 0.149 | 6.74E-06 | 0.904 | 20.267 |
|  | rs7935775 | A | T | TRUE | -0.063 | -0.071 | 0.014 | 0.169 | 4.73E-06 | 0.676 | 20.945 |
| Genus RuminococcaceaeUCG011(id.11368) | rs10274562 | C | T | FALSE | 0.111 | -0.054 | 0.024 | 0.153 | 5.75E-06 | 0.725 | 20.570 |
|  | rs12636310 | G | A | FALSE | 0.133 | -0.024 | 0.028 | 0.172 | 2.53E-06 | 0.888 | 22.146 |
|  | rs12724320 | C | T | FALSE | -0.121 | -0.045 | 0.025 | 0.153 | 1.23E-06 | 0.770 | 23.524 |
|  | rs1416041 | A | C | FALSE | -0.182 | 0.119 | 0.034 | 0.184 | 8.12E-08 | 0.519 | 28.778 |
|  | rs2729556 | C | T | FALSE | -0.109 | -0.183 | 0.023 | 0.149 | 3.04E-06 | 0.220 | 21.793 |
|  | rs4490371 | T | C | FALSE | -0.112 | 0.290 | 0.025 | 0.152 | 7.08E-06 | 0.056 | 20.172 |
|  | rs79113084 | C | T | FALSE | -0.152 | -0.083 | 0.032 | 0.240 | 1.65E-06 | 0.730 | 22.966 |
|  | rs9729514 | A | G | FALSE | 0.185 | 0.075 | 0.039 | 0.256 | 2.78E-06 | 0.770 | 21.962 |
| Genus RuminococcaceaeUCG013(id.11370) | rs11581881 | C | T | FALSE | 0.066 | -0.063 | 0.014 | 0.180 | 4.92E-06 | 0.728 | 20.870 |
|  | rs12189346 | G | A | FALSE | 0.068 | -0.030 | 0.015 | 0.191 | 2.54E-06 | 0.875 | 22.137 |
|  | rs12336782 | T | C | FALSE | -0.086 | 0.061 | 0.019 | 0.279 | 6.13E-06 | 0.827 | 20.448 |
|  | rs12485353 | G | A | FALSE | -0.061 | -0.054 | 0.013 | 0.172 | 3.39E-06 | 0.754 | 21.584 |
|  | rs12781711 | C | T | FALSE | -0.066 | -0.082 | 0.012 | 0.171 | 2.33E-08 | 0.632 | 31.194 |
|  | rs16918863 | A | C | FALSE | 0.111 | -0.127 | 0.024 | 0.307 | 3.44E-06 | 0.680 | 21.552 |
|  | rs1729063 | G | C | TRUE | -0.053 | 0.049 | 0.012 | 0.156 | 1.00E-05 | 0.754 | 19.512 |
|  | rs2428106 | C | G | TRUE | -0.049 | 0.116 | 0.011 | 0.150 | 7.96E-06 | 0.440 | 19.948 |
|  | rs2730183 | G | A | FALSE | -0.049 | 0.017 | 0.011 | 0.152 | 8.73E-06 | 0.909 | 19.770 |
|  | rs4385846 | G | T | FALSE | 0.060 | 0.060 | 0.013 | 0.190 | 5.63E-06 | 0.752 | 20.612 |
|  | rs75088940 | T | C | FALSE | -0.094 | -0.052 | 0.020 | 0.291 | 2.63E-06 | 0.857 | 22.072 |
|  | rs76973485 | G | T | FALSE | 0.195 | -0.345 | 0.042 | 0.377 | 3.13E-06 | 0.360 | 21.735 |
|  | rs7784330 | G | A | FALSE | -0.050 | -0.025 | 0.011 | 0.155 | 8.72E-06 | 0.874 | 19.774 |
|  | rs9313055 | T | C | FALSE | 0.105 | 0.014 | 0.023 | 0.272 | 7.39E-06 | 0.959 | 20.089 |
|  | rs9565219 | T | A | TRUE | -0.052 | -0.096 | 0.012 | 0.151 | 8.31E-06 | 0.528 | 19.865 |
| Genus RuminococcaceaeUCG014(id.11371) | rs10495392 | C | T | FALSE | -0.082 | -0.037 | 0.019 | 0.284 | 1.05E-05 | 0.897 | 19.417 |
|  | rs10791168 | A | G | FALSE | -0.066 | -0.224 | 0.015 | 0.194 | 9.48E-06 | 0.248 | 19.614 |
|  | rs10941294 | C | T | FALSE | -0.122 | 0.136 | 0.026 | 0.327 | 2.68E-06 | 0.677 | 22.036 |
|  | rs115777838 | T | C | FALSE | -0.188 | -0.061 | 0.039 | 0.239 | 1.11E-06 | 0.799 | 23.731 |
|  | rs12638134 | T | G | FALSE | 0.058 | 0.069 | 0.012 | 0.150 | 1.12E-06 | 0.646 | 23.702 |
|  | rs17296933 | C | G | TRUE | -0.083 | -0.301 | 0.019 | 0.235 | 7.37E-06 | 0.199 | 20.094 |
|  | rs34402072 | C | T | FALSE | -0.069 | -0.104 | 0.016 | 0.210 | 1.04E-05 | 0.620 | 19.431 |
|  | rs439810 | G | C | TRUE | -0.058 | -0.171 | 0.013 | 0.158 | 5.21E-06 | 0.278 | 20.757 |
|  | rs56105232 | G | A | FALSE | 0.139 | -0.396 | 0.030 | 0.315 | 3.22E-06 | 0.209 | 21.678 |
|  | rs61898819 | A | T | TRUE | 0.061 | -0.011 | 0.014 | 0.188 | 1.18E-05 | 0.952 | 19.190 |
|  | rs62478832 | T | A | TRUE | -0.058 | -0.053 | 0.013 | 0.157 | 6.64E-06 | 0.734 | 20.296 |
|  | rs72809222 | T | C | FALSE | 0.067 | -0.027 | 0.014 | 0.182 | 1.56E-06 | 0.882 | 23.078 |
|  | rs73186226 | G | A | FALSE | -0.099 | 0.193 | 0.022 | 0.289 | 4.57E-06 | 0.503 | 21.011 |
|  | rs74060145 | C | G | TRUE | -0.116 | -0.177 | 0.025 | 0.277 | 5.26E-06 | 0.521 | 20.741 |
|  | rs77627087 | C | G | TRUE | 0.068 | -0.114 | 0.015 | 0.195 | 6.76E-06 | 0.558 | 20.259 |
|  | rs79640386 | T | A | TRUE | -0.111 | 0.065 | 0.025 | 0.221 | 7.88E-06 | 0.770 | 19.968 |
|  | rs853612 | A | G | FALSE | -0.053 | -0.156 | 0.012 | 0.152 | 9.62E-06 | 0.305 | 19.585 |
|  | rs995642 | C | T | FALSE | 0.060 | -0.282 | 0.013 | 0.175 | 2.03E-06 | 0.106 | 22.562 |
| Genus Ruminococcus1(id.11373) | rs10769159 | G | C | TRUE | -0.064 | -0.140 | 0.011 | 0.152 | 5.97E-09 | 0.358 | 33.844 |
|  | rs10995816 | C | G | TRUE | -0.076 | 0.036 | 0.017 | 0.235 | 1.29E-05 | 0.879 | 19.028 |
|  | rs11783695 | G | T | FALSE | -0.073 | 0.234 | 0.016 | 0.205 | 5.40E-06 | 0.255 | 20.689 |
|  | rs17781867 | C | T | FALSE | 0.100 | 0.272 | 0.021 | 0.292 | 2.36E-06 | 0.352 | 22.275 |
|  | rs3000856 | T | A | TRUE | -0.071 | -0.244 | 0.016 | 0.203 | 1.29E-05 | 0.228 | 19.028 |
|  | rs3819978 | C | T | FALSE | -0.115 | -0.451 | 0.026 | 0.284 | 9.77E-06 | 0.112 | 19.557 |
|  | rs4849717 | T | A | TRUE | 0.133 | 0.196 | 0.030 | 0.285 | 9.50E-06 | 0.493 | 19.609 |
|  | rs6105066 | T | C | FALSE | -0.061 | 0.094 | 0.013 | 0.168 | 6.13E-06 | 0.579 | 20.447 |
|  | rs6493760 | C | T | FALSE | 0.054 | 0.020 | 0.012 | 0.156 | 3.86E-06 | 0.898 | 21.334 |
|  | rs6750529 | C | T | FALSE | 0.057 | 0.036 | 0.013 | 0.171 | 7.79E-06 | 0.835 | 19.989 |
|  | rs7117576 | A | G | FALSE | 0.083 | -0.159 | 0.017 | 0.260 | 1.21E-06 | 0.541 | 23.561 |
|  | rs7583465 | C | T | FALSE | 0.053 | 0.151 | 0.011 | 0.149 | 2.80E-06 | 0.311 | 21.952 |
|  | rs78572139 | G | A | FALSE | 0.125 | 0.109 | 0.028 | 0.249 | 7.64E-06 | 0.663 | 20.026 |
|  | rs78613526 | G | A | FALSE | 0.167 | 0.109 | 0.037 | 0.348 | 5.21E-06 | 0.753 | 20.757 |
| Genus Ruminococcus2(id.11374) | rs10199274 | G | C | TRUE | 0.066 | -0.108 | 0.014 | 0.187 | 1.94E-06 | 0.565 | 22.649 |
|  | rs12406309 | A | C | FALSE | -0.063 | -0.238 | 0.014 | 0.180 | 8.93E-06 | 0.185 | 19.728 |
|  | rs1819812 | G | T | FALSE | 0.084 | -0.216 | 0.018 | 0.332 | 5.22E-06 | 0.515 | 20.756 |
|  | rs2368224 | T | G | FALSE | 0.200 | -0.109 | 0.044 | 0.344 | 5.28E-06 | 0.752 | 20.734 |
|  | rs2846589 | G | T | FALSE | 0.052 | 0.027 | 0.012 | 0.152 | 7.26E-06 | 0.859 | 20.125 |
|  | rs2997412 | A | G | FALSE | -0.057 | 0.060 | 0.012 | 0.166 | 3.37E-06 | 0.718 | 21.594 |
|  | rs4400279 | A | G | FALSE | 0.055 | 0.009 | 0.012 | 0.159 | 5.42E-06 | 0.955 | 20.684 |
|  | rs4799823 | C | T | FALSE | 0.084 | 0.235 | 0.018 | 0.195 | 4.38E-06 | 0.229 | 21.089 |
|  | rs55707116 | C | A | FALSE | 0.087 | -0.065 | 0.019 | 0.281 | 4.74E-06 | 0.817 | 20.940 |
|  | rs58681734 | A | G | FALSE | 0.072 | 0.065 | 0.016 | 0.185 | 6.73E-06 | 0.727 | 20.269 |
|  | rs61791565 | T | C | FALSE | -0.052 | -0.045 | 0.012 | 0.150 | 7.83E-06 | 0.761 | 19.980 |
|  | rs75140805 | T | G | FALSE | 0.084 | -0.060 | 0.018 | 0.197 | 2.11E-06 | 0.762 | 22.493 |
|  | rs7635831 | G | A | FALSE | 0.062 | -0.231 | 0.013 | 0.155 | 1.65E-06 | 0.137 | 22.965 |
|  | rs7693984 | G | A | FALSE | -0.103 | -0.426 | 0.024 | 0.354 | 1.22E-05 | 0.229 | 19.130 |
|  | rs78120384 | A | G | FALSE | -0.193 | -0.467 | 0.039 | 0.258 | 8.73E-07 | 0.070 | 24.189 |
| Genus Sellimonas(id.14369) | rs113379006 | T | C | FALSE | -0.163 | 0.306 | 0.036 | 0.199 | 5.15E-06 | 0.123 | 20.782 |
|  | rs13417181 | T | C | FALSE | 0.167 | -0.381 | 0.034 | 0.180 | 8.09E-07 | 0.034 | 24.337 |
|  | rs2016057 | A | C | FALSE | -0.126 | 0.072 | 0.026 | 0.152 | 8.92E-07 | 0.638 | 24.149 |
|  | rs2187447 | A | C | FALSE | 0.243 | -0.021 | 0.053 | 0.310 | 3.96E-06 | 0.946 | 21.285 |
|  | rs2371572 | A | C | FALSE | 0.127 | -0.018 | 0.025 | 0.150 | 3.85E-07 | 0.906 | 25.770 |
|  | rs41816 | A | G | FALSE | 0.132 | 0.270 | 0.029 | 0.162 | 5.58E-06 | 0.097 | 20.626 |
|  | rs4600608 | A | G | FALSE | -0.137 | 0.003 | 0.030 | 0.182 | 5.47E-06 | 0.986 | 20.666 |
|  | rs553697 | T | C | FALSE | -0.154 | 0.305 | 0.034 | 0.194 | 5.77E-06 | 0.115 | 20.562 |
|  | rs56203279 | T | C | FALSE | -0.124 | -0.176 | 0.027 | 0.157 | 4.04E-06 | 0.263 | 21.246 |
|  | rs72553859 | G | C | TRUE | -0.150 | -0.346 | 0.033 | 0.201 | 6.09E-06 | 0.085 | 20.461 |
| Genus Senegalimassilia(id.11160) | rs10036909 | C | T | FALSE | 0.186 | -0.463 | 0.040 | 0.379 | 3.70E-06 | 0.222 | 21.416 |
|  | rs10221578 | G | A | FALSE | 0.078 | -0.001 | 0.017 | 0.149 | 5.72E-06 | 0.997 | 20.578 |
|  | rs11787826 | C | A | FALSE | 0.081 | -0.017 | 0.017 | 0.151 | 2.02E-06 | 0.909 | 22.579 |
|  | rs1990708 | A | C | FALSE | -0.110 | 0.181 | 0.025 | 0.273 | 9.69E-06 | 0.507 | 19.572 |
|  | rs2017373 | C | T | FALSE | 0.078 | -0.184 | 0.018 | 0.154 | 9.71E-06 | 0.233 | 19.567 |
|  | rs57512504 | T | A | TRUE | 0.082 | -0.006 | 0.017 | 0.150 | 1.96E-06 | 0.968 | 22.637 |
|  | rs7225245 | G | A | FALSE | 0.079 | -0.180 | 0.017 | 0.151 | 3.39E-06 | 0.234 | 21.583 |
|  | rs72887800 | T | A | TRUE | -0.082 | 0.136 | 0.018 | 0.152 | 2.86E-06 | 0.370 | 21.907 |
| Genus Slackia(id.825) | rs10409783 | A | G | FALSE | 0.095 | 0.087 | 0.021 | 0.166 | 6.76E-06 | 0.602 | 20.261 |
|  | rs112764253 | T | A | TRUE | 0.195 | 0.248 | 0.041 | 0.430 | 2.24E-06 | 0.564 | 22.379 |
|  | rs12440440 | A | G | FALSE | 0.090 | -0.069 | 0.019 | 0.157 | 2.22E-06 | 0.660 | 22.397 |
|  | rs13339230 | C | G | TRUE | 0.147 | -0.044 | 0.033 | 0.265 | 8.68E-06 | 0.868 | 19.782 |
|  | rs16894137 | C | T | FALSE | -0.123 | 0.089 | 0.026 | 0.221 | 3.04E-06 | 0.688 | 21.791 |
|  | rs35156985 | T | C | FALSE | -0.156 | 0.049 | 0.035 | 0.369 | 7.70E-06 | 0.894 | 20.010 |
|  | rs4492265 | A | G | FALSE | -0.091 | 0.018 | 0.019 | 0.162 | 2.29E-06 | 0.913 | 22.334 |
|  | rs58767323 | G | C | TRUE | -0.103 | -0.586 | 0.023 | 0.197 | 5.94E-06 | 0.003 | 20.508 |
|  | rs8901 | C | T | FALSE | 0.093 | -0.001 | 0.019 | 0.163 | 5.65E-07 | 0.997 | 25.028 |
| Genus Subdoligranulum(id.2070) | rs10065321 | T | C | FALSE | -0.051 | -0.031 | 0.011 | 0.151 | 2.10E-06 | 0.835 | 22.503 |
|  | rs10497836 | C | T | FALSE | -0.052 | 0.117 | 0.012 | 0.182 | 1.01E-05 | 0.521 | 19.493 |
|  | rs12638227 | G | C | TRUE | -0.056 | 0.087 | 0.011 | 0.152 | 2.84E-07 | 0.567 | 26.356 |
|  | rs1667315 | G | A | FALSE | 0.049 | 0.111 | 0.011 | 0.153 | 6.37E-06 | 0.467 | 20.374 |
|  | rs16962433 | A | T | TRUE | 0.086 | -0.192 | 0.019 | 0.305 | 6.07E-06 | 0.530 | 20.466 |
|  | rs2114677 | C | T | FALSE | -0.104 | -0.032 | 0.023 | 0.235 | 6.39E-06 | 0.892 | 20.368 |
|  | rs2171249 | C | T | FALSE | 0.107 | -0.118 | 0.023 | 0.287 | 4.71E-06 | 0.682 | 20.950 |
|  | rs35940633 | G | A | FALSE | -0.051 | -0.228 | 0.011 | 0.159 | 3.40E-06 | 0.151 | 21.577 |
|  | rs3761728 | T | G | FALSE | -0.054 | 0.073 | 0.012 | 0.172 | 4.83E-06 | 0.673 | 20.903 |
|  | rs4347804 | A | G | FALSE | 0.166 | -0.326 | 0.036 | 0.420 | 3.40E-06 | 0.438 | 21.579 |
|  | rs6555306 | T | C | FALSE | -0.074 | 0.014 | 0.016 | 0.213 | 1.89E-06 | 0.946 | 22.705 |
|  | rs75158211 | T | C | FALSE | -0.072 | -0.119 | 0.016 | 0.210 | 5.61E-06 | 0.571 | 20.616 |
|  | rs76528319 | G | T | FALSE | -0.143 | 0.196 | 0.031 | 0.268 | 3.99E-06 | 0.464 | 21.271 |
|  | rs76664262 | T | A | TRUE | 0.083 | -0.136 | 0.019 | 0.237 | 6.80E-06 | 0.566 | 20.249 |
| Genus Sutterella(id.2896) | rs1145877 | A | G | FALSE | -0.074 | -0.241 | 0.016 | 0.215 | 5.94E-06 | 0.263 | 20.507 |
|  | rs11591622 | T | G | FALSE | -0.069 | -0.191 | 0.015 | 0.202 | 5.43E-06 | 0.342 | 20.680 |
|  | rs13173038 | A | G | FALSE | -0.072 | -0.126 | 0.015 | 0.173 | 2.18E-06 | 0.467 | 22.428 |
|  | rs143438747 | T | C | FALSE | -0.146 | -0.167 | 0.031 | 0.283 | 2.02E-06 | 0.555 | 22.572 |
|  | rs2050185 | G | A | FALSE | 0.058 | -0.120 | 0.013 | 0.154 | 7.95E-06 | 0.437 | 19.950 |
|  | rs2321387 | G | A | FALSE | -0.059 | 0.274 | 0.012 | 0.150 | 1.92E-06 | 0.069 | 22.674 |
|  | rs2613606 | C | T | FALSE | -0.056 | -0.032 | 0.012 | 0.150 | 7.26E-06 | 0.834 | 20.124 |
|  | rs607327 | C | T | FALSE | 0.058 | 0.226 | 0.013 | 0.153 | 7.42E-06 | 0.141 | 20.083 |
|  | rs62501473 | G | A | FALSE | 0.069 | -0.005 | 0.015 | 0.169 | 3.38E-06 | 0.977 | 21.588 |
|  | rs7499539 | A | G | FALSE | 0.062 | 0.085 | 0.013 | 0.169 | 2.43E-06 | 0.616 | 22.218 |
|  | rs7638039 | T | C | FALSE | 0.065 | -0.227 | 0.014 | 0.173 | 7.22E-06 | 0.189 | 20.133 |
|  | rs9350083 | T | G | FALSE | -0.059 | 0.103 | 0.013 | 0.156 | 9.50E-06 | 0.508 | 19.609 |
| Genus Terrisporobacter(id.11348) | rs1883097 | C | T | FALSE | 0.226 | -0.456 | 0.045 | 0.385 | 6.37E-07 | 0.236 | 24.798 |
|  | rs2569953 | A | C | FALSE | -0.078 | -0.130 | 0.017 | 0.150 | 8.95E-06 | 0.388 | 19.723 |
|  | rs2872237 | C | A | FALSE | -0.081 | 0.110 | 0.018 | 0.152 | 3.67E-06 | 0.471 | 21.431 |
|  | rs58405430 | G | T | FALSE | 0.135 | -0.118 | 0.030 | 0.308 | 7.83E-06 | 0.701 | 19.978 |
|  | rs7034891 | G | C | TRUE | -0.080 | -0.154 | 0.017 | 0.152 | 4.25E-06 | 0.311 | 21.147 |
|  | rs7184125 | T | C | FALSE | 0.091 | 0.328 | 0.021 | 0.167 | 8.96E-06 | 0.050 | 19.721 |
| Genus Turicibacter(id.2162) | rs11054680 | T | C | FALSE | -0.105 | 0.040 | 0.023 | 0.198 | 3.94E-06 | 0.838 | 21.295 |
|  | rs114145484 | G | C | TRUE | -0.122 | 0.101 | 0.027 | 0.313 | 6.32E-06 | 0.747 | 20.388 |
|  | rs11649454 | G | C | TRUE | 0.095 | 0.088 | 0.020 | 0.205 | 2.95E-06 | 0.669 | 21.848 |
|  | rs11666533 | C | T | FALSE | -0.112 | -0.191 | 0.025 | 0.268 | 6.93E-06 | 0.476 | 20.211 |
|  | rs12603364 | T | C | FALSE | 0.111 | 0.063 | 0.023 | 0.208 | 8.92E-07 | 0.763 | 24.148 |
|  | rs149744580 | A | G | FALSE | 0.170 | 0.172 | 0.032 | 0.335 | 7.25E-08 | 0.608 | 28.997 |
|  | rs2221441 | G | C | TRUE | 0.071 | -0.108 | 0.015 | 0.150 | 3.66E-06 | 0.470 | 21.436 |
|  | rs2834977 | T | C | FALSE | -0.096 | 0.026 | 0.021 | 0.207 | 4.03E-06 | 0.899 | 21.248 |
|  | rs2952020 | G | A | FALSE | -0.076 | -0.061 | 0.017 | 0.173 | 4.67E-06 | 0.723 | 20.967 |
|  | rs4247078 | C | G | TRUE | 0.071 | -0.076 | 0.016 | 0.152 | 4.73E-06 | 0.619 | 20.945 |
|  | rs4869133 | G | A | FALSE | 0.131 | -0.063 | 0.027 | 0.198 | 1.41E-06 | 0.751 | 23.267 |
|  | rs55756211 | T | C | FALSE | -0.115 | 0.508 | 0.024 | 0.283 | 1.73E-06 | 0.072 | 22.871 |
|  | rs61265175 | G | C | TRUE | -0.086 | 0.087 | 0.019 | 0.192 | 3.81E-06 | 0.650 | 21.359 |
|  | rs7199484 | G | A | FALSE | -0.073 | 0.081 | 0.016 | 0.161 | 4.96E-06 | 0.614 | 20.853 |
| Genus Tyzzerella3(id.11335) | rs10898797 | C | T | FALSE | 0.122 | -0.054 | 0.027 | 0.238 | 8.38E-06 | 0.820 | 19.850 |
|  | rs112102233 | A | G | FALSE | -0.216 | -0.422 | 0.048 | 0.356 | 5.89E-06 | 0.235 | 20.522 |
|  | rs1232220 | G | T | FALSE | -0.144 | 0.109 | 0.032 | 0.249 | 6.18E-06 | 0.663 | 20.432 |
|  | rs17706273 | T | C | FALSE | -0.140 | -0.123 | 0.027 | 0.274 | 3.23E-07 | 0.654 | 26.109 |
|  | rs17809157 | A | T | TRUE | -0.164 | -0.159 | 0.034 | 0.238 | 1.12E-06 | 0.504 | 23.717 |
|  | rs191093 | G | A | FALSE | 0.159 | 0.000 | 0.035 | 0.242 | 6.78E-06 | 0.999 | 20.255 |
|  | rs4904512 | T | C | FALSE | -0.117 | 0.430 | 0.025 | 0.223 | 2.86E-06 | 0.053 | 21.905 |
|  | rs55799124 | A | G | FALSE | -0.114 | -0.058 | 0.024 | 0.169 | 1.65E-06 | 0.730 | 22.968 |
|  | rs67476743 | T | G | FALSE | 0.132 | 0.112 | 0.022 | 0.168 | 2.66E-09 | 0.506 | 35.417 |
|  | rs6920448 | C | T | FALSE | -0.141 | -0.403 | 0.031 | 0.256 | 3.86E-06 | 0.115 | 21.334 |
|  | rs7019909 | T | C | FALSE | 0.144 | -0.072 | 0.030 | 0.230 | 1.76E-06 | 0.754 | 22.841 |
|  | rs7333521 | T | C | FALSE | -0.207 | -0.008 | 0.045 | 0.414 | 4.82E-06 | 0.985 | 20.908 |
|  | rs75091807 | G | T | FALSE | -0.185 | -0.164 | 0.038 | 0.310 | 1.37E-06 | 0.596 | 23.320 |
|  | rs7561370 | T | C | FALSE | 0.131 | 0.224 | 0.029 | 0.210 | 4.48E-06 | 0.286 | 21.047 |
| Genus unknownGenus（id.826) | rs10060821 | T | C | FALSE | 0.062 | -0.014 | 0.014 | 0.173 | 9.30E-06 | 0.936 | 19.649 |
|  | rs10782329 | C | T | FALSE | 0.060 | 0.126 | 0.014 | 0.162 | 1.17E-05 | 0.435 | 19.204 |
|  | rs11257188 | A | C | FALSE | -0.110 | 0.287 | 0.023 | 0.225 | 1.24E-06 | 0.203 | 23.514 |
|  | rs13147561 | A | G | FALSE | -0.099 | -0.510 | 0.023 | 0.256 | 1.32E-05 | 0.046 | 18.974 |
|  | rs142372196 | A | G | FALSE | -0.074 | -0.078 | 0.016 | 0.251 | 4.76E-06 | 0.757 | 20.933 |
|  | rs55922055 | T | C | FALSE | -0.240 | 0.168 | 0.048 | 0.420 | 4.77E-07 | 0.690 | 25.354 |
|  | rs599752 | C | T | FALSE | -0.063 | 0.028 | 0.014 | 0.167 | 4.93E-06 | 0.869 | 20.866 |
|  | rs6431586 | A | G | FALSE | -0.095 | 0.102 | 0.021 | 0.273 | 3.51E-06 | 0.710 | 21.513 |
|  | rs66494181 | T | G | FALSE | 0.068 | 0.264 | 0.015 | 0.164 | 2.45E-06 | 0.107 | 22.208 |
|  | rs72846345 | T | C | FALSE | 0.088 | 0.218 | 0.019 | 0.269 | 4.28E-06 | 0.419 | 21.136 |
|  | rs740358 | G | T | FALSE | -0.130 | -0.055 | 0.026 | 0.305 | 8.83E-07 | 0.858 | 24.167 |
|  | rs74811325 | G | A | FALSE | 0.107 | -0.127 | 0.024 | 0.247 | 5.46E-06 | 0.609 | 20.668 |
|  | rs7935794 | C | G | TRUE | -0.127 | -0.173 | 0.027 | 0.320 | 3.33E-06 | 0.588 | 21.617 |
|  | rs9571849 | T | C | FALSE | 0.137 | 0.006 | 0.032 | 0.278 | 1.55E-05 | 0.982 | 18.672 |
|  | rs999857 | G | A | FALSE | 0.076 | 0.042 | 0.017 | 0.170 | 4.19E-06 | 0.804 | 21.177 |
| Genus unknownGenus（id.959) | rs10082444 | C | T | FALSE | -0.114 | 0.049 | 0.026 | 0.201 | 8.34E-06 | 0.806 | 19.858 |
|  | rs10892421 | C | T | FALSE | -0.104 | -0.072 | 0.021 | 0.150 | 1.21E-06 | 0.633 | 23.564 |
|  | rs115909738 | A | C | FALSE | -0.135 | 0.152 | 0.030 | 0.193 | 6.15E-06 | 0.432 | 20.442 |
|  | rs11691923 | G | A | FALSE | -0.092 | -0.004 | 0.021 | 0.149 | 8.06E-06 | 0.978 | 19.924 |
|  | rs1969793 | G | A | FALSE | 0.103 | -0.195 | 0.022 | 0.160 | 2.74E-06 | 0.222 | 21.991 |
|  | rs2199860 | C | G | TRUE | 0.121 | 0.097 | 0.022 | 0.169 | 5.09E-08 | 0.567 | 29.683 |
|  | rs2317372 | C | T | FALSE | -0.095 | -0.082 | 0.021 | 0.153 | 7.13E-06 | 0.593 | 20.159 |
|  | rs2831909 | T | C | FALSE | 0.132 | -0.109 | 0.031 | 0.228 | 1.57E-05 | 0.632 | 18.650 |
|  | rs474008 | T | A | TRUE | -0.102 | -0.021 | 0.023 | 0.173 | 9.31E-06 | 0.903 | 19.649 |
|  | rs4766410 | C | T | FALSE | 0.097 | 0.121 | 0.021 | 0.170 | 4.95E-06 | 0.476 | 20.858 |
|  | rs56210598 | C | G | TRUE | -0.112 | -0.035 | 0.023 | 0.163 | 7.76E-07 | 0.830 | 24.417 |
|  | rs66473610 | T | G | FALSE | 0.181 | 0.420 | 0.040 | 0.232 | 6.09E-06 | 0.070 | 20.459 |
|  | rs75285799 | T | C | FALSE | 0.132 | -0.058 | 0.028 | 0.205 | 1.64E-06 | 0.776 | 22.979 |
|  | rs9534068 | A | G | FALSE | 0.099 | -0.069 | 0.021 | 0.154 | 2.98E-06 | 0.653 | 21.832 |
| Genus unknownGenus（id.1868) | rs1044939 | C | T | FALSE | 0.109 | 0.185 | 0.024 | 0.249 | 6.65E-06 | 0.457 | 20.293 |
|  | rs115372687 | A | G | FALSE | 0.109 | -0.304 | 0.024 | 0.265 | 7.45E-06 | 0.251 | 20.075 |
|  | rs11709800 | T | C | FALSE | 0.088 | -0.155 | 0.017 | 0.200 | 1.23E-07 | 0.439 | 27.976 |
|  | rs12634544 | T | C | FALSE | -0.081 | -0.091 | 0.016 | 0.162 | 3.07E-07 | 0.576 | 26.208 |
|  | rs12930556 | C | T | FALSE | 0.064 | -0.204 | 0.014 | 0.150 | 4.38E-06 | 0.172 | 21.091 |
|  | rs138437711 | A | G | FALSE | -0.087 | -0.191 | 0.019 | 0.190 | 5.33E-06 | 0.316 | 20.715 |
|  | rs17291611 | A | G | FALSE | -0.091 | 0.258 | 0.019 | 0.212 | 2.09E-06 | 0.223 | 22.508 |
|  | rs3010848 | G | A | FALSE | -0.070 | 0.017 | 0.015 | 0.156 | 2.02E-06 | 0.915 | 22.578 |
|  | rs35947731 | C | T | FALSE | -0.131 | 0.077 | 0.029 | 0.256 | 5.37E-06 | 0.765 | 20.700 |
|  | rs4848741 | A | G | FALSE | -0.068 | 0.076 | 0.015 | 0.173 | 4.93E-06 | 0.661 | 20.863 |
|  | rs6595770 | G | T | FALSE | -0.085 | -0.140 | 0.018 | 0.188 | 1.50E-06 | 0.458 | 23.154 |
|  | rs72819830 | A | G | FALSE | 0.227 | 0.528 | 0.047 | 0.444 | 1.21E-06 | 0.235 | 23.557 |
|  | rs75546071 | G | C | TRUE | -0.160 | 0.812 | 0.036 | 0.335 | 7.02E-06 | 0.015 | 20.187 |
| Genus unknownGenus（id.2001) | rs10123122 | G | C | TRUE | 0.128 | 0.087 | 0.029 | 0.388 | 9.02E-06 | 0.822 | 19.708 |
|  | rs111373036 | A | G | FALSE | -0.086 | -0.329 | 0.018 | 0.211 | 1.80E-06 | 0.120 | 22.794 |
|  | rs11140959 | A | G | FALSE | 0.190 | 0.219 | 0.043 | 0.218 | 1.08E-05 | 0.316 | 19.365 |
|  | rs12696594 | A | G | FALSE | -0.064 | 0.220 | 0.014 | 0.150 | 5.94E-06 | 0.143 | 20.506 |
|  | rs2599390 | G | A | FALSE | 0.094 | 0.054 | 0.020 | 0.223 | 2.12E-06 | 0.809 | 22.481 |
|  | rs4876383 | T | A | TRUE | 0.076 | 0.145 | 0.015 | 0.164 | 6.56E-07 | 0.378 | 24.741 |
|  | rs55736413 | C | T | FALSE | 0.158 | -0.070 | 0.036 | 0.352 | 9.79E-06 | 0.841 | 19.553 |
|  | rs58850151 | T | C | FALSE | -0.081 | 0.250 | 0.018 | 0.168 | 8.35E-06 | 0.137 | 19.856 |
|  | rs6516833 | A | G | FALSE | 0.134 | 0.122 | 0.028 | 0.362 | 1.37E-06 | 0.736 | 23.321 |
|  | rs7334707 | T | G | FALSE | 0.119 | 0.337 | 0.027 | 0.343 | 8.00E-06 | 0.326 | 19.938 |
|  | rs79929882 | A | G | FALSE | -0.104 | 0.184 | 0.023 | 0.227 | 6.96E-06 | 0.418 | 20.203 |
| Genus unknownGenus（id.2041) | rs1032598 | G | A | FALSE | -0.089 | 0.069 | 0.019 | 0.200 | 2.74E-06 | 0.731 | 21.988 |
|  | rs11941716 | A | G | FALSE | 0.101 | 0.088 | 0.022 | 0.210 | 6.44E-06 | 0.675 | 20.353 |
|  | rs16843660 | A | G | FALSE | 0.235 | -0.517 | 0.049 | 0.419 | 1.73E-06 | 0.217 | 22.876 |
|  | rs1962916 | A | G | FALSE | 0.074 | -0.074 | 0.016 | 0.154 | 5.41E-06 | 0.630 | 20.687 |
|  | rs2336448 | T | C | FALSE | 0.077 | -0.187 | 0.016 | 0.149 | 1.51E-06 | 0.208 | 23.130 |
|  | rs249459 | A | G | FALSE | 0.074 | 0.038 | 0.017 | 0.157 | 7.89E-06 | 0.810 | 19.965 |
|  | rs2651663 | A | G | FALSE | -0.076 | 0.114 | 0.017 | 0.154 | 6.13E-06 | 0.459 | 20.448 |
|  | rs35703006 | G | T | FALSE | 0.093 | 0.026 | 0.019 | 0.172 | 1.19E-06 | 0.882 | 23.593 |
|  | rs553072 | G | A | FALSE | 0.109 | -0.040 | 0.023 | 0.194 | 2.10E-06 | 0.838 | 22.500 |
|  | rs6514318 | T | C | FALSE | 0.128 | 0.126 | 0.028 | 0.344 | 5.37E-06 | 0.715 | 20.701 |
|  | rs7187855 | A | C | FALSE | 0.200 | 0.104 | 0.042 | 0.351 | 1.76E-06 | 0.767 | 22.846 |
|  | rs921383 | G | A | FALSE | 0.072 | 0.101 | 0.016 | 0.153 | 5.95E-06 | 0.507 | 20.503 |
| Genus unknownGenus（id.2071) | rs10200320 | C | T | FALSE | 0.064 | 0.336 | 0.014 | 0.182 | 7.10E-06 | 0.065 | 20.167 |
|  | rs11195523 | A | C | FALSE | 0.069 | -0.084 | 0.015 | 0.173 | 2.11E-06 | 0.628 | 22.488 |
|  | rs11684166 | A | G | FALSE | -0.077 | 0.027 | 0.017 | 0.195 | 4.84E-06 | 0.890 | 20.900 |
|  | rs11809762 | G | A | FALSE | -0.093 | 0.100 | 0.019 | 0.188 | 8.92E-07 | 0.595 | 24.148 |
|  | rs11904514 | G | A | FALSE | -0.109 | -0.170 | 0.025 | 0.307 | 1.17E-05 | 0.579 | 19.209 |
|  | rs12147596 | C | T | FALSE | -0.072 | -0.065 | 0.014 | 0.161 | 3.71E-07 | 0.689 | 25.838 |
|  | rs16823675 | C | T | FALSE | -0.077 | 0.009 | 0.015 | 0.183 | 3.09E-07 | 0.960 | 26.191 |
|  | rs17086536 | C | A | FALSE | -0.101 | -0.207 | 0.022 | 0.287 | 6.93E-06 | 0.470 | 20.213 |
|  | rs1809136 | G | C | TRUE | 0.099 | 0.138 | 0.023 | 0.278 | 1.36E-05 | 0.620 | 18.923 |
|  | rs2898979 | G | C | TRUE | 0.090 | 0.248 | 0.020 | 0.211 | 8.25E-06 | 0.240 | 19.879 |
|  | rs2939766 | A | G | FALSE | -0.059 | 0.157 | 0.013 | 0.150 | 5.73E-06 | 0.294 | 20.577 |
|  | rs34985298 | G | A | FALSE | -0.062 | 0.025 | 0.014 | 0.158 | 5.97E-06 | 0.876 | 20.498 |
|  | rs35740166 | C | T | FALSE | -0.112 | 0.060 | 0.023 | 0.254 | 7.48E-07 | 0.814 | 24.488 |
|  | rs4644504 | T | C | FALSE | -0.097 | -0.037 | 0.022 | 0.297 | 7.31E-06 | 0.901 | 20.111 |
|  | rs6007642 | C | T | FALSE | -0.079 | 0.136 | 0.018 | 0.180 | 8.70E-06 | 0.452 | 19.777 |
|  | rs72700702 | T | C | FALSE | -0.092 | -0.241 | 0.019 | 0.236 | 1.22E-06 | 0.306 | 23.553 |
|  | rs76532867 | T | C | FALSE | 0.112 | 0.225 | 0.024 | 0.377 | 3.56E-06 | 0.551 | 21.490 |
| Genus unknownGenus（id.2755) | rs1035406 | G | A | FALSE | -0.111 | 0.137 | 0.025 | 0.231 | 9.78E-06 | 0.553 | 19.554 |
|  | rs11591293 | G | T | FALSE | 0.071 | -0.032 | 0.016 | 0.150 | 7.88E-06 | 0.831 | 19.967 |
|  | rs11630875 | T | C | FALSE | 0.093 | -0.200 | 0.020 | 0.223 | 5.68E-06 | 0.369 | 20.595 |
|  | rs12977163 | G | C | TRUE | 0.074 | -0.191 | 0.016 | 0.153 | 5.51E-06 | 0.213 | 20.651 |
|  | rs13336560 | C | T | FALSE | -0.072 | -0.152 | 0.016 | 0.153 | 5.13E-06 | 0.319 | 20.788 |
|  | rs1549633 | A | C | FALSE | 0.102 | 0.372 | 0.022 | 0.232 | 3.40E-06 | 0.110 | 21.576 |
|  | rs2074723 | T | C | FALSE | 0.166 | -0.615 | 0.038 | 0.332 | 9.61E-06 | 0.064 | 19.588 |
|  | rs3730086 | A | G | FALSE | 0.081 | 0.218 | 0.018 | 0.171 | 7.43E-06 | 0.201 | 20.078 |
|  | rs3754624 | C | T | FALSE | 0.096 | 0.031 | 0.020 | 0.195 | 1.52E-06 | 0.875 | 23.119 |
|  | rs55876211 | C | T | FALSE | -0.091 | 0.002 | 0.020 | 0.171 | 3.39E-06 | 0.989 | 21.579 |
|  | rs56250014 | A | T | TRUE | 0.119 | -0.247 | 0.026 | 0.256 | 3.73E-06 | 0.334 | 21.399 |
|  | rs61933850 | G | A | FALSE | 0.165 | -0.232 | 0.036 | 0.222 | 4.88E-06 | 0.297 | 20.883 |
|  | rs74354280 | C | T | FALSE | -0.090 | 0.044 | 0.021 | 0.166 | 1.10E-05 | 0.792 | 19.327 |
|  | rs76784716 | A | G | FALSE | 0.140 | 0.104 | 0.029 | 0.237 | 1.07E-06 | 0.661 | 23.805 |
|  | rs9813022 | A | G | FALSE | -0.082 | -0.169 | 0.016 | 0.154 | 5.26E-07 | 0.273 | 25.168 |
| Genus unknownGenus（id.1000000073) | rs10517600 | T | G | FALSE | 0.063 | 0.253 | 0.014 | 0.153 | 6.83E-06 | 0.099 | 20.241 |
|  | rs10904722 | C | T | FALSE | -0.067 | 0.091 | 0.015 | 0.173 | 4.88E-06 | 0.599 | 20.882 |
|  | rs118104867 | C | T | FALSE | 0.214 | -0.091 | 0.046 | 0.299 | 2.45E-06 | 0.761 | 22.207 |
|  | rs13409132 | A | G | FALSE | -0.165 | -0.134 | 0.035 | 0.391 | 2.64E-06 | 0.732 | 22.065 |
|  | rs17121075 | G | A | FALSE | 0.077 | -0.108 | 0.017 | 0.182 | 7.96E-06 | 0.552 | 19.948 |
|  | rs2191834 | G | T | FALSE | 0.075 | -0.124 | 0.016 | 0.174 | 2.73E-06 | 0.475 | 21.998 |
|  | rs28691777 | C | T | FALSE | 0.137 | -0.008 | 0.027 | 0.361 | 2.80E-07 | 0.982 | 26.380 |
|  | rs34088226 | A | G | FALSE | -0.118 | -0.274 | 0.027 | 0.320 | 1.21E-05 | 0.392 | 19.145 |
|  | rs55682560 | C | T | FALSE | -0.132 | -0.098 | 0.026 | 0.276 | 4.83E-07 | 0.722 | 25.330 |
|  | rs6588624 | G | A | FALSE | -0.066 | 0.034 | 0.014 | 0.149 | 1.63E-06 | 0.819 | 22.985 |
|  | rs66714985 | A | C | FALSE | 0.117 | -0.388 | 0.025 | 0.246 | 3.64E-06 | 0.115 | 21.446 |
|  | rs6755871 | C | G | TRUE | -0.061 | 0.167 | 0.014 | 0.149 | 1.00E-05 | 0.261 | 19.508 |
|  | rs7226487 | A | G | FALSE | -0.064 | 0.187 | 0.014 | 0.149 | 3.47E-06 | 0.207 | 21.537 |
|  | rs7538034 | T | G | FALSE | -0.079 | -0.126 | 0.017 | 0.201 | 2.19E-06 | 0.529 | 22.423 |
|  | rs7725895 | A | G | FALSE | -0.116 | 0.354 | 0.024 | 0.231 | 1.33E-06 | 0.125 | 23.380 |
|  | rs989682 | A | G | FALSE | 0.070 | -0.026 | 0.016 | 0.176 | 6.24E-06 | 0.884 | 20.413 |
|  | rs9979874 | G | C | TRUE | -0.074 | -0.089 | 0.015 | 0.159 | 9.76E-07 | 0.578 | 23.975 |
| Genus unknownGenus（id.1000001215) | rs11150282 | T | C | FALSE | 0.098 | 0.093 | 0.020 | 0.155 | 6.25E-07 | 0.549 | 24.834 |
|  | rs113884518 | T | C | FALSE | -0.206 | 0.068 | 0.046 | 0.471 | 6.13E-06 | 0.885 | 20.446 |
|  | rs1221147 | T | A | TRUE | 0.126 | 0.398 | 0.028 | 0.210 | 6.06E-06 | 0.058 | 20.471 |
|  | rs16851659 | G | C | TRUE | -0.090 | 0.055 | 0.019 | 0.156 | 1.60E-06 | 0.726 | 23.025 |
|  | rs28678345 | T | C | FALSE | 0.213 | -0.097 | 0.047 | 0.349 | 6.17E-06 | 0.780 | 20.434 |
|  | rs367480 | G | A | FALSE | -0.084 | 0.193 | 0.019 | 0.155 | 6.00E-06 | 0.214 | 20.487 |
|  | rs4129395 | G | A | FALSE | 0.090 | 0.261 | 0.019 | 0.149 | 1.05E-06 | 0.079 | 23.826 |
|  | rs73074665 | A | T | TRUE | 0.165 | -0.004 | 0.036 | 0.300 | 3.76E-06 | 0.990 | 21.381 |
|  | rs789069 | A | C | FALSE | -0.104 | 0.098 | 0.023 | 0.213 | 8.94E-06 | 0.645 | 19.725 |
|  | rs79790072 | T | C | FALSE | 0.226 | -0.139 | 0.049 | 0.429 | 3.60E-06 | 0.745 | 21.466 |
|  | rs8028558 | A | G | FALSE | 0.083 | 0.037 | 0.019 | 0.154 | 9.53E-06 | 0.811 | 19.603 |
|  | rs9864379 | T | C | FALSE | -0.161 | 0.513 | 0.029 | 0.210 | 4.18E-08 | 0.015 | 30.065 |
| Genus unknownGenus（id.1000005472) | rs10071529 | G | C | TRUE | 0.125 | 0.034 | 0.028 | 0.241 | 7.15E-06 | 0.887 | 20.154 |
|  | rs114184530 | T | C | FALSE | -0.155 | 0.181 | 0.034 | 0.267 | 5.48E-06 | 0.496 | 20.662 |
|  | rs11779863 | G | A | FALSE | -0.077 | 0.043 | 0.017 | 0.206 | 7.18E-06 | 0.833 | 20.146 |
|  | rs12566890 | T | G | FALSE | -0.103 | 0.341 | 0.024 | 0.223 | 1.99E-05 | 0.126 | 18.196 |
|  | rs13100746 | C | T | FALSE | 0.064 | 0.144 | 0.014 | 0.149 | 7.53E-06 | 0.335 | 20.055 |
|  | rs17235252 | T | C | FALSE | -0.122 | -0.120 | 0.026 | 0.231 | 1.79E-06 | 0.603 | 22.808 |
|  | rs3932485 | C | T | FALSE | 0.063 | 0.161 | 0.014 | 0.152 | 9.79E-06 | 0.288 | 19.551 |
|  | rs515984 | T | C | FALSE | -0.088 | 0.101 | 0.019 | 0.232 | 4.41E-06 | 0.663 | 21.078 |
|  | rs62188991 | G | C | TRUE | -0.111 | 0.297 | 0.024 | 0.320 | 4.36E-06 | 0.353 | 21.099 |
|  | rs638542 | G | A | FALSE | -0.071 | 0.024 | 0.016 | 0.164 | 6.96E-06 | 0.886 | 20.204 |
|  | rs739151 | C | G | TRUE | 0.065 | -0.016 | 0.014 | 0.149 | 3.02E-06 | 0.913 | 21.804 |
|  | rs74603314 | T | C | FALSE | 0.231 | 0.190 | 0.049 | 0.380 | 2.45E-06 | 0.616 | 22.207 |
|  | rs76373661 | G | A | FALSE | 0.091 | -0.076 | 0.020 | 0.204 | 7.67E-06 | 0.710 | 20.019 |
|  | rs7706512 | G | A | FALSE | 0.066 | 0.072 | 0.014 | 0.149 | 2.25E-06 | 0.626 | 22.371 |
|  | rs7801843 | A | G | FALSE | -0.087 | 0.072 | 0.019 | 0.208 | 7.81E-06 | 0.731 | 19.983 |
|  | rs949341 | G | A | FALSE | 0.066 | 0.087 | 0.015 | 0.166 | 8.19E-06 | 0.600 | 19.893 |
| Genus unknownGenus（id.1000005479) | rs10872669 | A | G | FALSE | -0.123 | 0.225 | 0.028 | 0.262 | 8.03E-06 | 0.392 | 19.932 |
|  | rs11135366 | C | G | TRUE | 0.084 | 0.109 | 0.018 | 0.175 | 4.58E-06 | 0.534 | 21.007 |
|  | rs12748533 | G | T | FALSE | -0.082 | 0.016 | 0.017 | 0.165 | 1.99E-06 | 0.922 | 22.605 |
|  | rs17043770 | G | A | FALSE | -0.177 | 0.126 | 0.034 | 0.243 | 2.74E-07 | 0.606 | 26.426 |
|  | rs61508842 | T | C | FALSE | 0.123 | 0.649 | 0.027 | 0.266 | 6.80E-06 | 0.014 | 20.250 |
|  | rs6831034 | T | A | TRUE | -0.096 | -0.062 | 0.021 | 0.257 | 3.15E-06 | 0.809 | 21.724 |
|  | rs689695 | C | A | FALSE | 0.081 | -0.119 | 0.017 | 0.163 | 1.08E-06 | 0.467 | 23.776 |
|  | rs738193 | T | C | FALSE | 0.085 | -0.176 | 0.017 | 0.156 | 3.24E-07 | 0.259 | 26.100 |
|  | rs78609301 | A | G | FALSE | -0.087 | -0.066 | 0.020 | 0.163 | 9.41E-06 | 0.686 | 19.628 |
|  | rs941000 | C | T | FALSE | 0.085 | 0.093 | 0.016 | 0.155 | 1.95E-07 | 0.549 | 27.083 |
| Genus unknownGenus（id.1000006162) | rs11251024 | G | A | FALSE | 0.104 | -0.317 | 0.021 | 0.168 | 4.65E-07 | 0.059 | 25.404 |
|  | rs11606187 | A | G | FALSE | -0.155 | -0.249 | 0.033 | 0.215 | 2.18E-06 | 0.248 | 22.432 |
|  | rs13219468 | G | C | TRUE | 0.115 | 0.221 | 0.024 | 0.165 | 1.13E-06 | 0.180 | 23.694 |
|  | rs13385922 | T | C | FALSE | 0.093 | 0.090 | 0.020 | 0.156 | 3.91E-06 | 0.564 | 21.311 |
|  | rs166849 | G | A | FALSE | 0.091 | -0.060 | 0.020 | 0.151 | 6.64E-06 | 0.693 | 20.293 |
|  | rs2172426 | C | T | FALSE | -0.102 | 0.140 | 0.020 | 0.152 | 2.85E-07 | 0.357 | 26.348 |
|  | rs267959 | A | G | FALSE | 0.099 | -0.035 | 0.021 | 0.166 | 2.42E-06 | 0.834 | 22.231 |
|  | rs2930903 | C | G | TRUE | 0.091 | 0.063 | 0.021 | 0.155 | 1.44E-05 | 0.684 | 18.810 |
|  | rs4383094 | T | C | FALSE | 0.149 | -0.026 | 0.032 | 0.215 | 3.28E-06 | 0.905 | 21.646 |
|  | rs55921101 | A | T | TRUE | -0.109 | 0.113 | 0.024 | 0.176 | 3.74E-06 | 0.519 | 21.392 |
|  | rs6051857 | A | G | FALSE | -0.134 | 0.017 | 0.029 | 0.193 | 4.55E-06 | 0.929 | 21.016 |
|  | rs60583455 | T | C | FALSE | 0.109 | 0.449 | 0.021 | 0.156 | 2.69E-07 | 0.004 | 26.460 |
|  | rs60775321 | T | C | FALSE | -0.096 | 0.041 | 0.021 | 0.165 | 6.77E-06 | 0.804 | 20.256 |
|  | rs72671304 | T | C | FALSE | 0.172 | 0.268 | 0.037 | 0.283 | 3.21E-06 | 0.344 | 21.688 |
|  | rs7911787 | G | T | FALSE | -0.223 | 0.035 | 0.047 | 0.422 | 2.10E-06 | 0.935 | 22.504 |
|  | rs9542068 | T | C | FALSE | 0.099 | 0.073 | 0.022 | 0.158 | 5.59E-06 | 0.642 | 20.625 |
| Genus Veillonella (id.2198) | rs11614532 | G | C | TRUE | 0.074 | 0.160 | 0.017 | 0.167 | 6.59E-06 | 0.336 | 20.309 |
|  | rs12679709 | C | G | TRUE | -0.079 | -0.056 | 0.016 | 0.155 | 1.48E-06 | 0.717 | 23.178 |
|  | rs1882878 | A | G | FALSE | -0.077 | -0.249 | 0.016 | 0.163 | 2.71E-06 | 0.127 | 22.010 |
|  | rs2013594 | T | C | FALSE | -0.072 | 0.058 | 0.016 | 0.153 | 3.40E-06 | 0.703 | 21.577 |
|  | rs55807413 | A | G | FALSE | 0.107 | 0.120 | 0.024 | 0.248 | 6.28E-06 | 0.628 | 20.400 |
|  | rs62376424 | C | T | FALSE | -0.076 | -0.032 | 0.016 | 0.161 | 3.13E-06 | 0.840 | 21.733 |
|  | rs6656807 | A | G | FALSE | 0.070 | 0.114 | 0.015 | 0.155 | 4.95E-06 | 0.462 | 20.855 |
|  | rs742016 | A | G | FALSE | -0.069 | 0.062 | 0.015 | 0.158 | 4.27E-06 | 0.695 | 21.137 |
|  | rs7645873 | A | T | TRUE | 0.076 | 0.290 | 0.016 | 0.176 | 3.55E-06 | 0.099 | 21.494 |
| Genus Victivallis (id.2256) | rs11899949 | G | A | FALSE | 0.131 | -0.017 | 0.028 | 0.161 | 2.28E-06 | 0.915 | 22.341 |
|  | rs12512543 | A | C | FALSE | -0.178 | -0.091 | 0.037 | 0.271 | 1.98E-06 | 0.738 | 22.619 |
|  | rs173120 | T | C | FALSE | 0.134 | 0.201 | 0.029 | 0.190 | 3.99E-06 | 0.290 | 21.272 |
|  | rs1882775 | A | G | FALSE | -0.138 | -0.065 | 0.031 | 0.194 | 9.81E-06 | 0.738 | 19.549 |
|  | rs2546432 | T | C | FALSE | -0.111 | 0.279 | 0.025 | 0.150 | 9.07E-06 | 0.063 | 19.698 |
|  | rs342302 | A | G | FALSE | -0.153 | -0.013 | 0.035 | 0.215 | 1.39E-05 | 0.953 | 18.878 |
|  | rs4764863 | G | A | FALSE | 0.122 | 0.203 | 0.025 | 0.150 | 7.80E-07 | 0.175 | 24.407 |
|  | rs4895919 | T | C | FALSE | -0.117 | 0.016 | 0.025 | 0.149 | 2.32E-06 | 0.915 | 22.313 |
|  | rs56349194 | A | G | FALSE | -0.159 | 0.317 | 0.032 | 0.220 | 4.95E-07 | 0.149 | 25.282 |
|  | rs592514 | T | A | TRUE | -0.181 | -0.245 | 0.039 | 0.225 | 3.61E-06 | 0.276 | 21.461 |
|  | rs6445926 | G | C | TRUE | 0.117 | -0.157 | 0.025 | 0.151 | 2.79E-06 | 0.298 | 21.955 |
|  | rs911666 | T | C | FALSE | -0.119 | -0.114 | 0.026 | 0.161 | 6.60E-06 | 0.480 | 20.305 |
| Family Acidaminococcaceae(id.2166) | rs262812 | T | C | FALSE | -0.066 | 0.007 | 0.014 | 0.163 | 3.90E-06 | 0.964 | 21.314 |
|  | rs2933324 | A | G | FALSE | -0.066 | -0.065 | 0.014 | 0.178 | 2.31E-06 | 0.715 | 22.315 |
|  | rs45497800 | T | C | FALSE | -0.118 | -0.303 | 0.026 | 0.215 | 4.65E-06 | 0.159 | 20.975 |
|  | rs6427992 | G | C | TRUE | -0.060 | -0.059 | 0.013 | 0.154 | 4.27E-06 | 0.701 | 21.138 |
|  | rs6589457 | A | G | FALSE | 0.166 | -0.111 | 0.035 | 0.342 | 2.07E-06 | 0.745 | 22.533 |
|  | rs6923842 | T | C | FALSE | -0.080 | 0.071 | 0.017 | 0.231 | 2.53E-06 | 0.759 | 22.140 |
|  | rs74540770 | G | A | FALSE | -0.109 | 0.319 | 0.024 | 0.274 | 7.66E-06 | 0.245 | 20.021 |
|  | rs78702810 | T | C | FALSE | -0.144 | -0.184 | 0.032 | 0.246 | 8.70E-06 | 0.454 | 19.778 |
| Family Actinomycetaceae(id.421) | rs2889192 | G | T | FALSE | 0.089 | -0.120 | 0.020 | 0.209 | 5.33E-06 | 0.565 | 20.714 |
|  | rs34583783 | G | T | FALSE | 0.124 | 0.014 | 0.026 | 0.314 | 2.83E-06 | 0.964 | 21.930 |
|  | rs35011108 | A | G | FALSE | 0.242 | -0.092 | 0.050 | 0.302 | 1.59E-06 | 0.761 | 23.041 |
|  | rs4073240 | G | A | FALSE | 0.075 | -0.202 | 0.016 | 0.153 | 5.64E-06 | 0.187 | 20.605 |
|  | rs58484246 | T | C | FALSE | 0.076 | -0.012 | 0.017 | 0.156 | 6.63E-06 | 0.941 | 20.297 |
| Family Alcaligenaceae(id.2875) | rs112135816 | T | G | FALSE | -0.078 | -0.440 | 0.017 | 0.307 | 6.25E-06 | 0.152 | 20.411 |
|  | rs112159068 | A | T | TRUE | 0.131 | -0.203 | 0.028 | 0.452 | 2.90E-06 | 0.653 | 21.881 |
|  | rs1153990 | A | G | FALSE | -0.059 | -0.028 | 0.013 | 0.161 | 4.71E-06 | 0.860 | 20.950 |
|  | rs147968 | C | T | FALSE | 0.049 | 0.230 | 0.011 | 0.149 | 9.13E-06 | 0.122 | 19.685 |
|  | rs17000015 | A | T | TRUE | -0.061 | 0.031 | 0.013 | 0.167 | 1.17E-06 | 0.853 | 23.619 |
|  | rs2367850 | G | C | TRUE | 0.063 | -0.053 | 0.013 | 0.183 | 1.78E-06 | 0.772 | 22.814 |
|  | rs28480294 | T | C | FALSE | -0.052 | 0.001 | 0.012 | 0.156 | 7.61E-06 | 0.996 | 20.033 |
|  | rs4033856 | C | T | FALSE | 0.082 | -0.363 | 0.017 | 0.264 | 1.27E-06 | 0.169 | 23.466 |
|  | rs62191117 | A | G | FALSE | 0.068 | 0.013 | 0.013 | 0.185 | 3.04E-07 | 0.944 | 26.221 |
|  | rs62395635 | T | C | FALSE | 0.111 | -0.088 | 0.024 | 0.315 | 3.55E-06 | 0.780 | 21.495 |
|  | rs67737557 | C | G | TRUE | 0.074 | -0.014 | 0.015 | 0.186 | 1.13E-06 | 0.941 | 23.700 |
|  | rs6969323 | A | C | FALSE | -0.059 | 0.011 | 0.013 | 0.178 | 4.30E-06 | 0.953 | 21.127 |
|  | rs7302582 | G | C | TRUE | -0.107 | 0.115 | 0.023 | 0.329 | 4.54E-06 | 0.727 | 21.020 |
|  | rs74776516 | T | G | FALSE | -0.094 | -0.093 | 0.021 | 0.295 | 9.59E-06 | 0.753 | 19.591 |
|  | rs7638039 | T | C | FALSE | 0.060 | -0.227 | 0.013 | 0.173 | 2.34E-06 | 0.189 | 22.295 |
|  | rs9537886 | A | C | FALSE | -0.057 | 0.258 | 0.011 | 0.150 | 2.50E-07 | 0.085 | 26.604 |
| Family Bacteroidaceae(id.917) | rs11585893 | A | G | FALSE | -0.074 | -0.315 | 0.015 | 0.175 | 5.24E-07 | 0.072 | 25.175 |
|  | rs13207588 | A | G | FALSE | -0.059 | 0.248 | 0.013 | 0.191 | 6.40E-06 | 0.194 | 20.365 |
|  | rs1340391 | T | C | FALSE | -0.059 | -0.192 | 0.013 | 0.216 | 7.58E-06 | 0.375 | 20.040 |
|  | rs17619981 | T | G | FALSE | 0.088 | 0.255 | 0.019 | 0.217 | 2.46E-06 | 0.240 | 22.194 |
|  | rs2023437 | T | C | FALSE | -0.078 | 0.164 | 0.017 | 0.228 | 3.06E-06 | 0.472 | 21.780 |
|  | rs2366421 | T | A | TRUE | -0.053 | -0.177 | 0.012 | 0.191 | 6.50E-06 | 0.356 | 20.334 |
|  | rs28757219 | T | A | TRUE | 0.082 | -0.215 | 0.017 | 0.230 | 1.55E-06 | 0.351 | 23.089 |
|  | rs495004 | C | G | TRUE | -0.061 | -0.202 | 0.013 | 0.177 | 2.90E-06 | 0.254 | 21.881 |
|  | rs66474973 | G | T | FALSE | 0.081 | 0.169 | 0.016 | 0.244 | 7.81E-07 | 0.489 | 24.403 |
|  | rs66710942 | C | T | FALSE | 0.049 | -0.161 | 0.011 | 0.149 | 5.53E-06 | 0.279 | 20.644 |
|  | rs6795673 | C | T | FALSE | 0.054 | 0.236 | 0.011 | 0.149 | 3.11E-07 | 0.112 | 26.183 |
|  | rs9507307 | C | T | FALSE | 0.060 | 0.230 | 0.013 | 0.174 | 2.85E-06 | 0.187 | 21.912 |
| Family BacteroidalesS24.7group(id.11173) | rs10872669 | A | G | FALSE | -0.123 | 0.225 | 0.028 | 0.262 | 8.03E-06 | 0.392 | 19.932 |
|  | rs11135366 | C | G | TRUE | 0.084 | 0.109 | 0.018 | 0.175 | 4.58E-06 | 0.534 | 21.007 |
|  | rs12748533 | G | T | FALSE | -0.082 | 0.016 | 0.017 | 0.165 | 1.99E-06 | 0.922 | 22.605 |
|  | rs17043770 | G | A | FALSE | -0.177 | 0.126 | 0.034 | 0.243 | 2.74E-07 | 0.606 | 26.426 |
|  | rs61508842 | T | C | FALSE | 0.123 | 0.649 | 0.027 | 0.266 | 6.80E-06 | 0.014 | 20.250 |
|  | rs6831034 | T | A | TRUE | -0.096 | -0.062 | 0.021 | 0.257 | 3.15E-06 | 0.809 | 21.724 |
|  | rs689695 | C | A | FALSE | 0.081 | -0.119 | 0.017 | 0.163 | 1.08E-06 | 0.467 | 23.776 |
|  | rs738193 | T | C | FALSE | 0.085 | -0.176 | 0.017 | 0.156 | 3.24E-07 | 0.259 | 26.100 |
|  | rs78609301 | A | G | FALSE | -0.087 | -0.066 | 0.020 | 0.163 | 9.41E-06 | 0.686 | 19.628 |
|  | rs941000 | C | T | FALSE | 0.085 | 0.093 | 0.016 | 0.155 | 1.95E-07 | 0.549 | 27.083 |
| Family Bifidobacteriaceae(id.433) | rs182549 | A | G | FALSE | 0.117 | 0.072 | 0.013 | 0.152 | 2.47E-20 | 0.638 | 85.372 |
| Family Christensenellaceae(id.1866) | rs117186816 | G | A | FALSE | -0.206 | 0.141 | 0.044 | 0.264 | 2.23E-06 | 0.594 | 22.389 |
|  | rs12380890 | A | G | FALSE | -0.050 | -0.205 | 0.011 | 0.149 | 5.67E-06 | 0.169 | 20.597 |
|  | rs12657403 | A | G | FALSE | 0.078 | -0.502 | 0.017 | 0.331 | 5.52E-06 | 0.129 | 20.649 |
|  | rs4076564 | G | A | FALSE | -0.189 | 0.270 | 0.039 | 0.335 | 1.50E-06 | 0.421 | 23.143 |
|  | rs4805124 | C | G | TRUE | 0.049 | 0.285 | 0.011 | 0.148 | 8.17E-06 | 0.054 | 19.897 |
|  | rs5763231 | C | T | FALSE | -0.068 | -0.079 | 0.015 | 0.170 | 5.06E-06 | 0.645 | 20.815 |
|  | rs62573205 | G | A | FALSE | -0.065 | -0.097 | 0.013 | 0.185 | 9.35E-07 | 0.599 | 24.057 |
|  | rs7211194 | C | T | FALSE | 0.049 | 0.153 | 0.011 | 0.149 | 9.14E-06 | 0.307 | 19.684 |
|  | rs72706624 | G | T | FALSE | 0.088 | -0.125 | 0.020 | 0.242 | 7.92E-06 | 0.607 | 19.957 |
|  | rs77867022 | G | T | FALSE | -0.160 | 0.055 | 0.038 | 0.285 | 2.37E-05 | 0.847 | 17.866 |
|  | rs870002 | C | T | FALSE | 0.049 | 0.324 | 0.011 | 0.151 | 8.49E-06 | 0.032 | 19.824 |
|  | rs892686 | A | G | FALSE | 0.051 | -0.009 | 0.011 | 0.149 | 3.92E-06 | 0.954 | 21.304 |
| Family Clostridiaceae1(id.1869) | rs10875374 | C | T | FALSE | -0.054 | 0.112 | 0.012 | 0.149 | 6.99E-06 | 0.450 | 20.197 |
|  | rs12186080 | G | A | FALSE | 0.075 | 0.022 | 0.016 | 0.201 | 4.11E-06 | 0.914 | 21.212 |
|  | rs12341505 | G | A | FALSE | 0.081 | -0.119 | 0.018 | 0.254 | 5.58E-06 | 0.640 | 20.627 |
|  | rs12490337 | C | G | TRUE | -0.062 | 0.047 | 0.014 | 0.185 | 6.62E-06 | 0.798 | 20.300 |
|  | rs2795528 | G | A | FALSE | -0.181 | -0.151 | 0.039 | 0.321 | 3.67E-06 | 0.638 | 21.432 |
|  | rs2817172 | C | T | FALSE | 0.056 | -0.238 | 0.012 | 0.152 | 5.46E-06 | 0.118 | 20.668 |
|  | rs4723021 | T | C | FALSE | -0.106 | -0.427 | 0.024 | 0.296 | 1.10E-05 | 0.150 | 19.331 |
|  | rs550843 | T | C | FALSE | -0.073 | 0.122 | 0.017 | 0.168 | 1.28E-05 | 0.467 | 19.043 |
|  | rs56188186 | A | G | FALSE | 0.097 | -0.050 | 0.022 | 0.345 | 8.58E-06 | 0.886 | 19.804 |
|  | rs62397761 | A | G | FALSE | 0.062 | -0.314 | 0.014 | 0.159 | 6.14E-06 | 0.048 | 20.443 |
|  | rs881532 | A | G | FALSE | -0.053 | -0.170 | 0.012 | 0.148 | 7.50E-06 | 0.253 | 20.060 |
| Family ClostridialesvadinBB60group(id.11286) | rs10517600 | T | G | FALSE | 0.063 | 0.253 | 0.014 | 0.153 | 6.83E-06 | 0.099 | 20.241 |
|  | rs10904722 | C | T | FALSE | -0.067 | 0.091 | 0.015 | 0.173 | 4.88E-06 | 0.599 | 20.882 |
|  | rs118104867 | C | T | FALSE | 0.214 | -0.091 | 0.046 | 0.299 | 2.45E-06 | 0.761 | 22.207 |
|  | rs13409132 | A | G | FALSE | -0.165 | -0.134 | 0.035 | 0.391 | 2.64E-06 | 0.732 | 22.065 |
|  | rs17121075 | G | A | FALSE | 0.077 | -0.108 | 0.017 | 0.182 | 7.96E-06 | 0.552 | 19.948 |
|  | rs2191834 | G | T | FALSE | 0.075 | -0.124 | 0.016 | 0.174 | 2.73E-06 | 0.475 | 21.998 |
|  | rs28691777 | C | T | FALSE | 0.137 | -0.008 | 0.027 | 0.361 | 2.80E-07 | 0.982 | 26.380 |
|  | rs34088226 | A | G | FALSE | -0.118 | -0.274 | 0.027 | 0.320 | 1.21E-05 | 0.392 | 19.145 |
|  | rs55682560 | C | T | FALSE | -0.132 | -0.098 | 0.026 | 0.276 | 4.83E-07 | 0.722 | 25.330 |
|  | rs6588624 | G | A | FALSE | -0.066 | 0.034 | 0.014 | 0.149 | 1.63E-06 | 0.819 | 22.985 |
|  | rs66714985 | A | C | FALSE | 0.117 | -0.388 | 0.025 | 0.246 | 3.64E-06 | 0.115 | 21.446 |
|  | rs6755871 | C | G | TRUE | -0.061 | 0.167 | 0.014 | 0.149 | 1.00E-05 | 0.261 | 19.508 |
|  | rs7226487 | A | G | FALSE | -0.064 | 0.187 | 0.014 | 0.149 | 3.47E-06 | 0.207 | 21.537 |
|  | rs7538034 | T | G | FALSE | -0.079 | -0.126 | 0.017 | 0.201 | 2.19E-06 | 0.529 | 22.423 |
|  | rs7725895 | A | G | FALSE | -0.116 | 0.354 | 0.024 | 0.231 | 1.33E-06 | 0.125 | 23.380 |
|  | rs989682 | A | G | FALSE | 0.070 | -0.026 | 0.016 | 0.176 | 6.24E-06 | 0.884 | 20.413 |
|  | rs9979874 | G | C | TRUE | -0.074 | -0.089 | 0.015 | 0.159 | 9.76E-07 | 0.578 | 23.975 |
| Family Coriobacteriaceae(id.811) | rs11250875 | T | C | FALSE | 0.061 | 0.342 | 0.013 | 0.180 | 3.49E-06 | 0.057 | 21.526 |
|  | rs11656361 | A | C | FALSE | 0.077 | -0.071 | 0.018 | 0.194 | 1.06E-05 | 0.713 | 19.394 |
|  | rs12974142 | G | A | FALSE | 0.079 | 0.502 | 0.018 | 0.290 | 8.31E-06 | 0.083 | 19.865 |
|  | rs13307134 | C | T | FALSE | 0.057 | 0.016 | 0.013 | 0.199 | 7.46E-06 | 0.937 | 20.072 |
|  | rs1397793 | G | A | FALSE | -0.050 | -0.063 | 0.011 | 0.164 | 9.15E-06 | 0.701 | 19.681 |
|  | rs1816223 | A | G | FALSE | -0.059 | -0.186 | 0.013 | 0.186 | 5.51E-06 | 0.318 | 20.652 |
|  | rs240104 | T | C | FALSE | -0.060 | 0.054 | 0.013 | 0.166 | 1.96E-06 | 0.745 | 22.630 |
|  | rs2442778 | G | A | FALSE | -0.116 | -0.140 | 0.026 | 0.338 | 6.72E-06 | 0.678 | 20.272 |
|  | rs3025411 | A | G | FALSE | 0.093 | 0.020 | 0.021 | 0.245 | 9.72E-06 | 0.934 | 19.566 |
|  | rs34739816 | G | T | FALSE | 0.097 | -0.171 | 0.021 | 0.317 | 3.37E-06 | 0.590 | 21.594 |
|  | rs62448869 | T | A | TRUE | -0.049 | -0.081 | 0.011 | 0.150 | 8.20E-06 | 0.588 | 19.891 |
|  | rs67561917 | A | G | FALSE | -0.071 | 0.086 | 0.015 | 0.194 | 3.56E-06 | 0.659 | 21.486 |
|  | rs719099 | A | G | FALSE | 0.078 | 0.104 | 0.016 | 0.251 | 5.86E-07 | 0.680 | 24.957 |
|  | rs76779974 | C | G | TRUE | 0.077 | -0.398 | 0.017 | 0.254 | 6.53E-06 | 0.118 | 20.326 |
|  | rs80046645 | C | G | TRUE | 0.256 | -0.621 | 0.056 | 0.380 | 5.31E-06 | 0.102 | 20.724 |
|  | rs8010111 | G | A | FALSE | -0.103 | -0.082 | 0.023 | 0.268 | 6.52E-06 | 0.759 | 20.328 |
| Family Defluviitaleaceae(id.1924) | rs112893842 | T | C | FALSE | 0.111 | 0.331 | 0.023 | 0.257 | 1.91E-06 | 0.197 | 22.686 |
|  | rs1582238 | T | C | FALSE | 0.080 | -0.113 | 0.017 | 0.155 | 1.59E-06 | 0.468 | 23.042 |
|  | rs17051335 | C | T | FALSE | -0.134 | -0.069 | 0.029 | 0.248 | 4.29E-06 | 0.781 | 21.129 |
|  | rs1908593 | T | C | FALSE | 0.070 | -0.401 | 0.016 | 0.152 | 7.32E-06 | 0.008 | 20.108 |
|  | rs2357086 | T | C | FALSE | -0.112 | -0.210 | 0.022 | 0.284 | 3.16E-07 | 0.459 | 26.152 |
|  | rs28696126 | A | T | TRUE | -0.107 | -0.005 | 0.024 | 0.223 | 6.89E-06 | 0.982 | 20.223 |
|  | rs4344384 | G | T | FALSE | 0.071 | -0.229 | 0.016 | 0.149 | 5.62E-06 | 0.125 | 20.612 |
|  | rs4677103 | A | G | FALSE | 0.098 | -0.097 | 0.020 | 0.197 | 7.06E-07 | 0.621 | 24.598 |
|  | rs540220 | T | C | FALSE | -0.124 | -0.292 | 0.029 | 0.260 | 1.89E-05 | 0.261 | 18.293 |
|  | rs55658617 | T | C | FALSE | 0.177 | -0.424 | 0.036 | 0.400 | 9.57E-07 | 0.290 | 24.013 |
|  | rs9608282 | T | G | FALSE | 0.139 | 0.184 | 0.030 | 0.414 | 3.44E-06 | 0.656 | 21.554 |
|  | rs9725395 | A | G | FALSE | -0.138 | 0.028 | 0.030 | 0.235 | 2.77E-06 | 0.905 | 21.969 |
| Family Desulfovibrionaceae(id.3169) | rs112381107 | C | T | FALSE | 0.211 | -0.084 | 0.046 | 0.314 | 3.71E-06 | 0.790 | 21.409 |
|  | rs11599763 | T | C | FALSE | -0.056 | -0.032 | 0.012 | 0.153 | 2.23E-06 | 0.836 | 22.388 |
|  | rs16851319 | G | C | TRUE | -0.073 | -0.244 | 0.015 | 0.197 | 1.21E-06 | 0.217 | 23.556 |
|  | rs17791387 | A | G | FALSE | -0.073 | 0.104 | 0.015 | 0.253 | 2.32E-06 | 0.680 | 22.314 |
|  | rs2692012 | A | G | FALSE | 0.114 | -0.302 | 0.025 | 0.329 | 6.64E-06 | 0.359 | 20.294 |
|  | rs2838334 | G | A | FALSE | 0.057 | 0.038 | 0.012 | 0.156 | 4.23E-06 | 0.809 | 21.157 |
|  | rs3935584 | C | T | FALSE | -0.053 | -0.259 | 0.012 | 0.149 | 5.56E-06 | 0.083 | 20.635 |
|  | rs4506934 | C | T | FALSE | -0.094 | -0.189 | 0.020 | 0.232 | 2.81E-06 | 0.415 | 21.945 |
|  | rs6058181 | C | T | FALSE | 0.083 | -0.012 | 0.017 | 0.202 | 5.03E-07 | 0.954 | 25.253 |
|  | rs7164160 | A | T | TRUE | -0.057 | -0.156 | 0.013 | 0.193 | 9.12E-06 | 0.418 | 19.687 |
|  | rs72647048 | T | C | FALSE | -0.077 | -0.040 | 0.017 | 0.234 | 6.60E-06 | 0.865 | 20.307 |
|  | rs9928243 | C | A | FALSE | -0.054 | -0.102 | 0.012 | 0.149 | 4.27E-06 | 0.495 | 21.142 |
| Family Enterobacteriaceae(id.3469) | rs11026530 | T | C | FALSE | 0.082 | 0.220 | 0.019 | 0.211 | 1.02E-05 | 0.297 | 19.471 |
|  | rs111229068 | A | T | TRUE | 0.111 | 0.216 | 0.024 | 0.278 | 4.79E-06 | 0.439 | 20.921 |
|  | rs2374343 | C | G | TRUE | 0.058 | -0.234 | 0.013 | 0.151 | 3.80E-06 | 0.122 | 21.365 |
|  | rs35673018 | G | A | FALSE | 0.090 | 0.054 | 0.020 | 0.261 | 9.29E-06 | 0.837 | 19.653 |
|  | rs4792380 | A | T | TRUE | 0.116 | -0.632 | 0.026 | 0.436 | 7.26E-06 | 0.147 | 20.125 |
|  | rs504442 | T | G | FALSE | 0.084 | 0.113 | 0.019 | 0.244 | 8.93E-06 | 0.644 | 19.728 |
|  | rs6092361 | A | G | FALSE | 0.060 | 0.304 | 0.013 | 0.156 | 3.05E-06 | 0.052 | 21.786 |
|  | rs61973590 | C | G | TRUE | -0.061 | 0.081 | 0.013 | 0.157 | 5.95E-06 | 0.603 | 20.504 |
|  | rs78143293 | A | G | FALSE | -0.085 | 0.039 | 0.017 | 0.228 | 6.39E-07 | 0.864 | 24.792 |
|  | rs79757635 | C | A | FALSE | 0.076 | 0.090 | 0.017 | 0.221 | 9.47E-06 | 0.683 | 19.615 |
|  | rs80319214 | C | G | TRUE | 0.099 | -0.305 | 0.022 | 0.288 | 4.48E-06 | 0.289 | 21.048 |
| Family Erysipelotrichaceae(id.2149) | rs10781552 | C | T | FALSE | -0.055 | 0.080 | 0.012 | 0.165 | 1.96E-06 | 0.629 | 22.633 |
|  | rs17530232 | A | G | FALSE | 0.103 | 0.276 | 0.022 | 0.333 | 4.49E-06 | 0.407 | 21.042 |
|  | rs1884466 | C | T | FALSE | -0.048 | 0.294 | 0.011 | 0.149 | 8.78E-06 | 0.049 | 19.760 |
|  | rs2300774 | G | A | FALSE | 0.052 | -0.113 | 0.011 | 0.150 | 9.18E-07 | 0.448 | 24.094 |
|  | rs290833 | T | G | FALSE | -0.050 | -0.020 | 0.011 | 0.150 | 7.98E-06 | 0.894 | 19.942 |
|  | rs35161940 | T | C | FALSE | -0.081 | 0.079 | 0.017 | 0.241 | 1.52E-06 | 0.742 | 23.118 |
|  | rs4078432 | C | T | FALSE | -0.061 | -0.184 | 0.013 | 0.198 | 5.31E-06 | 0.354 | 20.723 |
|  | rs56970041 | T | G | FALSE | 0.072 | -0.096 | 0.016 | 0.308 | 1.07E-05 | 0.756 | 19.385 |
|  | rs62504403 | C | T | FALSE | 0.068 | -0.039 | 0.013 | 0.188 | 1.00E-07 | 0.837 | 28.371 |
|  | rs7234058 | T | C | FALSE | -0.095 | 0.383 | 0.019 | 0.260 | 1.10E-06 | 0.141 | 23.744 |
|  | rs7826267 | T | G | FALSE | -0.084 | -0.081 | 0.020 | 0.305 | 2.51E-05 | 0.791 | 17.755 |
|  | rs8003149 | C | T | FALSE | 0.054 | 0.021 | 0.012 | 0.158 | 4.04E-06 | 0.896 | 21.248 |
|  | rs921306 | T | C | FALSE | 0.049 | 0.144 | 0.011 | 0.153 | 6.02E-06 | 0.345 | 20.481 |
| Family FamilyXI(id.1936) | rs10759623 | C | T | FALSE | -0.162 | 0.167 | 0.032 | 0.179 | 4.60E-07 | 0.351 | 25.425 |
|  | rs11547158 | A | G | FALSE | -0.178 | 0.128 | 0.037 | 0.219 | 1.90E-06 | 0.559 | 22.696 |
|  | rs17379710 | T | C | FALSE | -0.116 | -0.022 | 0.025 | 0.151 | 3.91E-06 | 0.885 | 21.308 |
|  | rs2155352 | A | G | FALSE | -0.151 | -0.061 | 0.030 | 0.175 | 6.38E-07 | 0.727 | 24.795 |
|  | rs2156611 | T | C | FALSE | -0.112 | -0.250 | 0.025 | 0.149 | 7.47E-06 | 0.092 | 20.068 |
|  | rs3733511 | A | G | FALSE | 0.128 | -0.008 | 0.027 | 0.163 | 3.03E-06 | 0.960 | 21.795 |
|  | rs488164 | T | G | FALSE | 0.118 | -0.353 | 0.026 | 0.155 | 3.76E-06 | 0.023 | 21.386 |
|  | rs6025153 | G | C | TRUE | 0.119 | 0.199 | 0.026 | 0.153 | 5.96E-06 | 0.194 | 20.502 |
|  | rs697771 | A | G | FALSE | -0.118 | -0.118 | 0.025 | 0.149 | 2.88E-06 | 0.432 | 21.895 |
|  | rs78222136 | G | C | TRUE | 0.225 | -0.050 | 0.051 | 0.263 | 1.11E-05 | 0.850 | 19.308 |
| Family FamilyXIII(id.1957) | rs10404377 | C | A | FALSE | -0.050 | -0.211 | 0.011 | 0.153 | 6.58E-06 | 0.168 | 20.311 |
|  | rs118170811 | A | G | FALSE | 0.152 | -0.680 | 0.032 | 0.425 | 1.74E-06 | 0.109 | 22.860 |
|  | rs12643275 | A | T | TRUE | -0.055 | -0.086 | 0.012 | 0.162 | 2.25E-06 | 0.598 | 22.372 |
|  | rs12904405 | A | G | FALSE | -0.085 | 0.328 | 0.019 | 0.255 | 9.82E-06 | 0.198 | 19.546 |
|  | rs1999289 | A | T | TRUE | -0.064 | -0.221 | 0.014 | 0.178 | 4.73E-06 | 0.216 | 20.944 |
|  | rs3098182 | G | T | FALSE | 0.051 | 0.040 | 0.011 | 0.149 | 3.89E-06 | 0.786 | 21.316 |
|  | rs482905 | G | T | FALSE | 0.060 | 0.357 | 0.013 | 0.172 | 2.72E-06 | 0.038 | 22.003 |
|  | rs6501525 | A | G | FALSE | 0.056 | 0.405 | 0.012 | 0.158 | 1.18E-06 | 0.010 | 23.603 |
|  | rs66753613 | G | A | FALSE | 0.065 | -0.091 | 0.014 | 0.195 | 6.36E-06 | 0.642 | 20.375 |
|  | rs6797051 | C | T | FALSE | -0.081 | 0.041 | 0.017 | 0.253 | 2.56E-06 | 0.871 | 22.121 |
|  | rs7076829 | T | A | TRUE | -0.052 | 0.064 | 0.011 | 0.155 | 4.27E-06 | 0.680 | 21.140 |
|  | rs7514702 | T | C | FALSE | -0.066 | 0.165 | 0.014 | 0.208 | 2.83E-06 | 0.429 | 21.931 |
| Family Lachnospiraceae(id.1987) | rs10402491 | C | T | FALSE | 0.066 | 0.213 | 0.015 | 0.199 | 8.37E-06 | 0.283 | 19.852 |
|  | rs11139361 | T | C | FALSE | 0.049 | -0.149 | 0.011 | 0.159 | 7.65E-06 | 0.347 | 20.022 |
|  | rs112040820 | A | G | FALSE | 0.055 | -0.055 | 0.012 | 0.170 | 2.68E-06 | 0.748 | 22.032 |
|  | rs11755180 | C | G | TRUE | -0.049 | -0.064 | 0.010 | 0.150 | 2.69E-06 | 0.668 | 22.024 |
|  | rs11841382 | G | T | FALSE | -0.072 | 0.351 | 0.017 | 0.269 | 3.91E-05 | 0.192 | 16.913 |
|  | rs11979110 | T | C | FALSE | -0.050 | 0.079 | 0.011 | 0.149 | 1.86E-06 | 0.597 | 22.730 |
|  | rs1205443 | A | G | FALSE | 0.050 | -0.149 | 0.011 | 0.157 | 7.80E-06 | 0.342 | 19.987 |
|  | rs12760724 | A | C | FALSE | -0.048 | 0.067 | 0.011 | 0.158 | 7.14E-06 | 0.673 | 20.155 |
|  | rs13005175 | A | G | FALSE | 0.099 | 0.088 | 0.022 | 0.361 | 5.39E-06 | 0.807 | 20.694 |
|  | rs2159863 | A | G | FALSE | -0.059 | 0.137 | 0.013 | 0.194 | 5.35E-06 | 0.480 | 20.706 |
|  | rs2910921 | T | C | FALSE | 0.160 | -0.064 | 0.036 | 0.441 | 7.69E-06 | 0.884 | 20.014 |
|  | rs3127230 | C | T | FALSE | -0.050 | 0.048 | 0.011 | 0.161 | 7.20E-06 | 0.767 | 20.141 |
|  | rs35524804 | T | C | FALSE | -0.061 | 0.222 | 0.013 | 0.181 | 1.23E-06 | 0.222 | 23.537 |
|  | rs7359994 | T | C | FALSE | -0.050 | 0.012 | 0.011 | 0.151 | 7.71E-06 | 0.939 | 20.009 |
|  | rs79086868 | T | C | FALSE | 0.078 | 0.127 | 0.016 | 0.247 | 2.33E-06 | 0.607 | 22.302 |
|  | rs959845 | C | T | FALSE | -0.049 | -0.186 | 0.011 | 0.153 | 4.66E-06 | 0.224 | 20.971 |
|  | rs9929145 | G | A | FALSE | -0.126 | -0.469 | 0.025 | 0.342 | 2.95E-07 | 0.170 | 26.282 |
| Family Lactobacillaceae(id.1836) | rs1530559 | G | A | FALSE | 0.077 | -0.025 | 0.018 | 0.151 | 1.26E-05 | 0.869 | 19.063 |
|  | rs16861661 | G | A | FALSE | -0.193 | -0.649 | 0.038 | 0.304 | 3.47E-07 | 0.033 | 25.969 |
|  | rs328312 | T | A | TRUE | 0.083 | 0.172 | 0.017 | 0.149 | 9.30E-07 | 0.250 | 24.068 |
|  | rs6092149 | A | T | TRUE | -0.083 | -0.160 | 0.017 | 0.151 | 1.24E-06 | 0.289 | 23.510 |
|  | rs62314653 | C | A | FALSE | 0.177 | 0.337 | 0.039 | 0.318 | 6.35E-06 | 0.289 | 20.379 |
|  | rs7399658 | G | A | FALSE | -0.105 | 0.339 | 0.022 | 0.195 | 2.07E-06 | 0.082 | 22.529 |
|  | rs74599091 | A | G | FALSE | 0.192 | 0.165 | 0.043 | 0.549 | 6.54E-06 | 0.764 | 20.323 |
|  | rs768253 | T | G | FALSE | -0.079 | 0.163 | 0.017 | 0.150 | 3.41E-06 | 0.276 | 21.570 |
|  | rs77478751 | A | G | FALSE | -0.219 | -0.050 | 0.047 | 0.234 | 3.69E-06 | 0.830 | 21.421 |
|  | rs921925 | A | C | FALSE | 0.100 | 0.090 | 0.020 | 0.181 | 7.22E-07 | 0.619 | 24.555 |
|  | rs9345899 | A | G | FALSE | -0.124 | -0.422 | 0.028 | 0.243 | 8.72E-06 | 0.082 | 19.774 |
| Family Methanobacteriaceae(id.121) | rs10202904 | T | G | FALSE | -0.122 | 0.015 | 0.024 | 0.151 | 2.30E-07 | 0.923 | 26.761 |
|  | rs10424197 | G | A | FALSE | -0.111 | -0.129 | 0.025 | 0.171 | 6.94E-06 | 0.451 | 20.211 |
|  | rs11018665 | A | T | TRUE | 0.111 | -0.159 | 0.025 | 0.173 | 8.61E-06 | 0.357 | 19.797 |
|  | rs12825290 | C | G | TRUE | -0.217 | -0.013 | 0.049 | 0.317 | 1.12E-05 | 0.967 | 19.296 |
|  | rs4257531 | G | A | FALSE | 0.164 | -0.412 | 0.036 | 0.244 | 6.57E-06 | 0.091 | 20.316 |
|  | rs56131665 | G | A | FALSE | 0.179 | -0.057 | 0.039 | 0.243 | 5.46E-06 | 0.813 | 20.669 |
|  | rs62241835 | G | T | FALSE | -0.203 | -0.757 | 0.042 | 0.519 | 1.14E-06 | 0.145 | 23.683 |
|  | rs6508769 | T | C | FALSE | 0.154 | 0.058 | 0.034 | 0.209 | 8.35E-06 | 0.783 | 19.856 |
|  | rs73068003 | G | T | FALSE | -0.158 | 0.133 | 0.035 | 0.250 | 6.95E-06 | 0.594 | 20.206 |
|  | rs73457410 | A | G | FALSE | 0.215 | 0.466 | 0.044 | 0.301 | 8.18E-07 | 0.122 | 24.316 |
|  | rs75208022 | C | T | FALSE | -0.227 | 0.103 | 0.049 | 0.253 | 3.16E-06 | 0.684 | 21.717 |
|  | rs894996 | C | A | FALSE | 0.217 | -0.519 | 0.045 | 0.286 | 1.35E-06 | 0.069 | 23.349 |
| Family Oxalobacteraceae(id.2966) | rs111966731 | T | C | FALSE | 0.204 | 0.187 | 0.045 | 0.265 | 4.71E-06 | 0.481 | 20.952 |
|  | rs11246212 | T | C | FALSE | 0.136 | -0.126 | 0.029 | 0.214 | 3.11E-06 | 0.555 | 21.745 |
|  | rs12002250 | A | C | FALSE | 0.196 | 0.479 | 0.045 | 0.373 | 1.06E-05 | 0.200 | 19.408 |
|  | rs12509763 | C | G | TRUE | -0.163 | -0.223 | 0.036 | 0.252 | 6.00E-06 | 0.376 | 20.487 |
|  | rs1569853 | T | C | FALSE | -0.140 | -0.037 | 0.028 | 0.229 | 6.65E-07 | 0.871 | 24.714 |
|  | rs17138946 | G | T | FALSE | -0.189 | -0.366 | 0.043 | 0.296 | 1.07E-05 | 0.216 | 19.388 |
|  | rs36057338 | G | T | FALSE | 0.182 | 0.464 | 0.040 | 0.410 | 5.25E-06 | 0.257 | 20.743 |
|  | rs4428215 | G | A | FALSE | 0.126 | -0.104 | 0.023 | 0.171 | 4.76E-08 | 0.542 | 29.812 |
|  | rs561239 | A | G | FALSE | 0.106 | 0.190 | 0.024 | 0.184 | 8.32E-06 | 0.299 | 19.864 |
|  | rs6000536 | C | T | FALSE | -0.118 | -0.096 | 0.024 | 0.199 | 9.35E-07 | 0.628 | 24.058 |
|  | rs62435498 | C | A | FALSE | 0.182 | -0.086 | 0.040 | 0.247 | 5.87E-06 | 0.729 | 20.531 |
|  | rs736744 | C | T | FALSE | 0.106 | -0.144 | 0.020 | 0.150 | 1.40E-07 | 0.337 | 27.721 |
|  | rs7993559 | C | A | FALSE | 0.092 | -0.128 | 0.020 | 0.151 | 4.65E-06 | 0.398 | 20.975 |
|  | rs80330081 | A | C | FALSE | -0.188 | 0.141 | 0.042 | 0.245 | 9.56E-06 | 0.566 | 19.597 |
|  | rs934049 | G | A | FALSE | 0.110 | -0.034 | 0.024 | 0.184 | 4.24E-06 | 0.853 | 21.152 |
| Family Pasteurellaceae(id.3689) | rs10840326 | C | G | TRUE | -0.072 | 0.092 | 0.015 | 0.155 | 1.52E-06 | 0.552 | 23.126 |
|  | rs10965428 | C | A | FALSE | -0.120 | -0.126 | 0.026 | 0.323 | 3.43E-06 | 0.697 | 21.561 |
|  | rs111582866 | G | A | FALSE | -0.114 | -0.056 | 0.026 | 0.265 | 8.81E-06 | 0.832 | 19.753 |
|  | rs12050685 | A | G | FALSE | -0.067 | 0.088 | 0.015 | 0.164 | 1.07E-05 | 0.594 | 19.385 |
|  | rs12191680 | C | G | TRUE | 0.102 | 0.096 | 0.020 | 0.245 | 2.18E-07 | 0.695 | 26.869 |
|  | rs16970009 | A | G | FALSE | 0.187 | -0.752 | 0.043 | 0.524 | 1.29E-05 | 0.151 | 19.027 |
|  | rs35510 | A | G | FALSE | 0.123 | 0.142 | 0.026 | 0.366 | 3.62E-06 | 0.698 | 21.456 |
|  | rs4822728 | T | C | FALSE | 0.069 | -0.224 | 0.015 | 0.149 | 4.23E-06 | 0.132 | 21.156 |
|  | rs6092684 | A | T | TRUE | 0.068 | -0.137 | 0.015 | 0.150 | 3.08E-06 | 0.361 | 21.769 |
|  | rs62568866 | T | A | TRUE | -0.118 | 0.071 | 0.026 | 0.243 | 5.29E-06 | 0.770 | 20.730 |
|  | rs6972479 | A | G | FALSE | -0.078 | 0.063 | 0.018 | 0.184 | 8.25E-06 | 0.732 | 19.878 |
|  | rs72756943 | G | A | FALSE | 0.140 | 0.629 | 0.030 | 0.298 | 3.91E-06 | 0.035 | 21.308 |
|  | rs73139353 | A | C | FALSE | -0.223 | 0.491 | 0.048 | 0.265 | 4.38E-06 | 0.064 | 21.092 |
|  | rs731534 | C | G | TRUE | -0.099 | -0.082 | 0.022 | 0.236 | 5.10E-06 | 0.729 | 20.798 |
|  | rs76022354 | C | T | FALSE | 0.243 | -0.247 | 0.050 | 0.341 | 1.21E-06 | 0.469 | 23.560 |
|  | rs78909003 | T | C | FALSE | -0.241 | -0.208 | 0.050 | 0.328 | 1.31E-06 | 0.525 | 23.415 |
|  | rs9382510 | C | T | FALSE | -0.088 | -0.122 | 0.017 | 0.169 | 2.12E-07 | 0.468 | 26.921 |
|  | rs9895850 | T | C | FALSE | -0.176 | 0.143 | 0.041 | 0.365 | 1.70E-05 | 0.695 | 18.497 |
|  | rs9938097 | T | C | FALSE | -0.071 | 0.044 | 0.016 | 0.153 | 6.94E-06 | 0.775 | 20.209 |
| Family Peptococcaceae(id.2024) | rs117452796 | A | G | FALSE | -0.258 | -0.357 | 0.055 | 0.423 | 2.68E-06 | 0.398 | 22.036 |
|  | rs12144792 | C | T | FALSE | 0.064 | -0.076 | 0.014 | 0.154 | 5.13E-06 | 0.623 | 20.788 |
|  | rs12634826 | T | G | FALSE | -0.074 | 0.106 | 0.015 | 0.156 | 9.48E-07 | 0.499 | 24.031 |
|  | rs12992764 | T | G | FALSE | 0.068 | 0.130 | 0.014 | 0.151 | 1.25E-06 | 0.392 | 23.502 |
|  | rs150600492 | A | C | FALSE | 0.136 | 0.437 | 0.029 | 0.320 | 2.79E-06 | 0.172 | 21.957 |
|  | rs35703006 | G | T | FALSE | 0.081 | 0.026 | 0.016 | 0.172 | 7.63E-07 | 0.882 | 24.448 |
|  | rs4990837 | A | G | FALSE | 0.091 | 0.213 | 0.019 | 0.189 | 9.68E-07 | 0.262 | 23.990 |
|  | rs75430375 | C | T | FALSE | -0.148 | -0.442 | 0.032 | 0.351 | 3.17E-06 | 0.208 | 21.712 |
|  | rs75898026 | A | G | FALSE | -0.082 | -0.143 | 0.017 | 0.183 | 2.19E-06 | 0.437 | 22.423 |
| Family Peptostreptococcaceae(id.2042) | rs10805326 | G | A | FALSE | 0.057 | -0.132 | 0.012 | 0.163 | 3.92E-06 | 0.418 | 21.306 |
|  | rs10805326 | G | A | FALSE | 0.057 | -1.081 | 0.012 | 1.413 | 3.92E-06 | 0.444 | 21.306 |
|  | rs117020988 | C | T | FALSE | 0.182 | 0.341 | 0.037 | 0.274 | 9.49E-07 | 0.214 | 24.028 |
|  | rs12377846 | C | A | FALSE | -0.252 | -0.459 | 0.051 | 0.438 | 8.42E-07 | 0.295 | 24.260 |
|  | rs12986312 | T | G | FALSE | 0.057 | 0.058 | 0.013 | 0.162 | 5.62E-06 | 0.721 | 20.613 |
|  | rs1467258 | G | A | FALSE | 0.073 | 0.024 | 0.016 | 0.192 | 7.76E-06 | 0.900 | 19.995 |
|  | rs1520207 | C | T | FALSE | 0.053 | 0.117 | 0.011 | 0.149 | 2.96E-06 | 0.431 | 21.842 |
|  | rs2644627 | C | G | TRUE | -0.056 | -0.052 | 0.011 | 0.151 | 6.49E-07 | 0.731 | 24.761 |
|  | rs4692811 | C | T | FALSE | 0.064 | -0.005 | 0.013 | 0.158 | 4.21E-07 | 0.977 | 25.595 |
|  | rs59865771 | C | T | FALSE | -0.057 | -0.269 | 0.013 | 0.157 | 6.45E-06 | 0.087 | 20.349 |
|  | rs61841503 | G | A | FALSE | 0.092 | 0.337 | 0.016 | 0.221 | 1.19E-08 | 0.127 | 32.495 |
|  | rs6721459 | A | G | FALSE | 0.051 | 0.257 | 0.011 | 0.151 | 5.29E-06 | 0.089 | 20.730 |
|  | rs75819860 | T | A | TRUE | 0.126 | 0.130 | 0.027 | 0.243 | 2.54E-06 | 0.594 | 22.133 |
|  | rs76982728 | T | C | FALSE | 0.124 | -0.521 | 0.027 | 0.509 | 3.27E-06 | 0.306 | 21.652 |
|  | rs77540684 | T | G | FALSE | 0.107 | -0.332 | 0.025 | 0.255 | 1.39E-05 | 0.193 | 18.877 |
|  | rs9573934 | C | T | FALSE | -0.069 | -0.236 | 0.014 | 0.201 | 1.20E-06 | 0.239 | 23.574 |
| Family Porphyromonadaceae(id.943) | rs10119172 | C | G | TRUE | -0.074 | 0.071 | 0.014 | 0.195 | 2.33E-07 | 0.718 | 26.739 |
|  | rs10762312 | G | A | FALSE | -0.052 | -0.128 | 0.012 | 0.161 | 1.05E-05 | 0.424 | 19.427 |
|  | rs10858364 | G | T | FALSE | 0.055 | -0.011 | 0.012 | 0.176 | 4.71E-06 | 0.953 | 20.951 |
|  | rs17065783 | A | G | FALSE | -0.059 | -0.080 | 0.012 | 0.201 | 1.31E-06 | 0.690 | 23.403 |
|  | rs1980561 | A | G | FALSE | -0.049 | -0.018 | 0.011 | 0.149 | 8.97E-06 | 0.903 | 19.719 |
|  | rs3111851 | C | G | TRUE | 0.058 | -0.132 | 0.012 | 0.170 | 1.56E-06 | 0.436 | 23.071 |
|  | rs35233670 | T | C | FALSE | -0.047 | 0.209 | 0.011 | 0.149 | 7.94E-06 | 0.160 | 19.953 |
|  | rs35961441 | A | C | FALSE | 0.092 | 0.123 | 0.021 | 0.383 | 1.01E-05 | 0.748 | 19.492 |
|  | rs6953849 | A | G | FALSE | 0.072 | -0.208 | 0.015 | 0.193 | 1.89E-06 | 0.282 | 22.702 |
|  | rs7330827 | T | C | FALSE | -0.104 | -0.055 | 0.024 | 0.318 | 1.21E-05 | 0.864 | 19.140 |
|  | rs864093 | A | C | FALSE | -0.053 | -0.070 | 0.012 | 0.182 | 7.02E-06 | 0.698 | 20.188 |
| Family Prevotellaceae(id.960) | rs10131942 | C | G | TRUE | 0.070 | 0.141 | 0.016 | 0.202 | 9.47E-06 | 0.484 | 19.615 |
|  | rs12057990 | C | T | FALSE | 0.059 | -0.219 | 0.013 | 0.167 | 8.21E-06 | 0.191 | 19.889 |
|  | rs12118202 | T | C | FALSE | -0.075 | -0.115 | 0.015 | 0.192 | 3.28E-07 | 0.550 | 26.075 |
|  | rs13069367 | A | C | FALSE | -0.054 | -0.203 | 0.012 | 0.152 | 6.49E-06 | 0.180 | 20.338 |
|  | rs148376875 | T | G | FALSE | 0.085 | 0.363 | 0.018 | 0.207 | 2.39E-06 | 0.079 | 22.252 |
|  | rs2206482 | T | G | FALSE | -0.057 | 0.274 | 0.012 | 0.152 | 1.27E-06 | 0.071 | 23.464 |
|  | rs2278540 | G | A | FALSE | 0.055 | 0.007 | 0.012 | 0.155 | 6.91E-06 | 0.966 | 20.218 |
|  | rs34071565 | T | A | TRUE | -0.157 | -0.277 | 0.035 | 0.312 | 6.66E-06 | 0.373 | 20.290 |
|  | rs34660375 | A | G | FALSE | -0.081 | 0.124 | 0.018 | 0.212 | 6.18E-06 | 0.560 | 20.431 |
|  | rs3758087 | T | C | FALSE | 0.056 | 0.206 | 0.012 | 0.166 | 6.01E-06 | 0.215 | 20.485 |
|  | rs3860225 | A | G | FALSE | 0.084 | 0.250 | 0.017 | 0.259 | 5.79E-07 | 0.336 | 24.981 |
|  | rs4493272 | T | C | FALSE | -0.060 | 0.096 | 0.012 | 0.149 | 2.96E-07 | 0.518 | 26.275 |
|  | rs4685827 | T | C | FALSE | -0.068 | -0.143 | 0.015 | 0.175 | 2.96E-06 | 0.412 | 21.840 |
|  | rs7252711 | A | G | FALSE | -0.074 | -0.156 | 0.016 | 0.238 | 4.75E-06 | 0.512 | 20.934 |
|  | rs7975087 | C | A | FALSE | -0.060 | -0.532 | 0.014 | 0.196 | 8.99E-06 | 0.007 | 19.716 |
|  | rs912860 | G | A | FALSE | -0.229 | 0.606 | 0.048 | 0.484 | 2.13E-06 | 0.211 | 22.476 |
|  | rs9586501 | G | A | FALSE | 0.059 | 0.166 | 0.013 | 0.169 | 3.24E-06 | 0.325 | 21.666 |
|  | rs9958960 | G | A | FALSE | -0.091 | 0.043 | 0.017 | 0.205 | 1.37E-07 | 0.833 | 27.765 |
| Family Rhodospirillaceae(id.2717) | rs1035406 | G | A | FALSE | -0.114 | 0.137 | 0.025 | 0.231 | 6.01E-06 | 0.553 | 20.485 |
|  | rs11591293 | G | T | FALSE | 0.074 | -0.032 | 0.016 | 0.150 | 2.84E-06 | 0.831 | 21.923 |
|  | rs12977163 | G | C | TRUE | 0.073 | -0.191 | 0.016 | 0.153 | 7.79E-06 | 0.213 | 19.989 |
|  | rs13336560 | C | T | FALSE | -0.070 | -0.152 | 0.016 | 0.153 | 9.02E-06 | 0.319 | 19.709 |
|  | rs1549633 | A | C | FALSE | 0.100 | 0.372 | 0.022 | 0.232 | 4.86E-06 | 0.110 | 20.891 |
|  | rs16855101 | C | T | FALSE | 0.119 | 0.075 | 0.025 | 0.222 | 1.87E-06 | 0.736 | 22.728 |
|  | rs1923415 | A | G | FALSE | -0.100 | -0.154 | 0.023 | 0.261 | 1.10E-05 | 0.556 | 19.321 |
|  | rs3754624 | C | T | FALSE | 0.097 | 0.031 | 0.020 | 0.195 | 1.20E-06 | 0.875 | 23.575 |
|  | rs4278423 | T | C | FALSE | 0.108 | 0.018 | 0.024 | 0.305 | 5.08E-06 | 0.953 | 20.809 |
|  | rs4822789 | G | C | TRUE | 0.073 | -0.041 | 0.016 | 0.153 | 4.77E-06 | 0.788 | 20.927 |
|  | rs55876211 | C | T | FALSE | -0.091 | 0.002 | 0.020 | 0.171 | 3.43E-06 | 0.989 | 21.557 |
|  | rs56250014 | A | T | TRUE | 0.115 | -0.247 | 0.026 | 0.256 | 7.06E-06 | 0.334 | 20.177 |
|  | rs61933850 | G | A | FALSE | 0.165 | -0.232 | 0.036 | 0.222 | 4.88E-06 | 0.297 | 20.883 |
|  | rs6679026 | T | C | FALSE | 0.112 | 0.012 | 0.025 | 0.245 | 8.20E-06 | 0.960 | 19.891 |
|  | rs72714493 | A | G | FALSE | 0.082 | 0.054 | 0.018 | 0.208 | 6.24E-06 | 0.795 | 20.414 |
|  | rs74354280 | C | T | FALSE | -0.091 | 0.044 | 0.020 | 0.166 | 8.58E-06 | 0.792 | 19.805 |
|  | rs9813022 | A | G | FALSE | -0.084 | -0.169 | 0.016 | 0.154 | 2.61E-07 | 0.273 | 26.522 |
| Family Rikenellaceae(id.967) | rs10217435 | C | T | FALSE | -0.088 | -0.102 | 0.020 | 0.215 | 7.66E-06 | 0.637 | 20.022 |
|  | rs10832801 | A | C | FALSE | -0.053 | 0.050 | 0.012 | 0.166 | 1.30E-05 | 0.764 | 19.005 |
|  | rs1939881 | G | A | FALSE | -0.106 | -0.059 | 0.021 | 0.320 | 3.05E-07 | 0.854 | 26.219 |
|  | rs2447496 | G | A | FALSE | -0.055 | -0.439 | 0.012 | 0.170 | 6.63E-06 | 0.010 | 20.297 |
|  | rs36021379 | A | G | FALSE | -0.066 | 0.387 | 0.014 | 0.203 | 5.98E-06 | 0.057 | 20.496 |
|  | rs4264350 | T | C | FALSE | -0.053 | -0.010 | 0.011 | 0.149 | 1.24E-06 | 0.947 | 23.514 |
|  | rs4783173 | C | G | TRUE | 0.048 | 0.005 | 0.011 | 0.153 | 1.20E-05 | 0.973 | 19.160 |
|  | rs59663348 | G | A | FALSE | 0.057 | 0.033 | 0.013 | 0.172 | 4.98E-06 | 0.850 | 20.845 |
|  | rs62532512 | C | A | FALSE | -0.050 | 0.045 | 0.011 | 0.150 | 2.66E-06 | 0.766 | 22.045 |
|  | rs6744030 | C | T | FALSE | 0.070 | -0.296 | 0.016 | 0.184 | 9.21E-06 | 0.107 | 19.669 |
|  | rs6837275 | A | G | FALSE | 0.057 | -0.095 | 0.012 | 0.163 | 1.60E-06 | 0.559 | 23.022 |
|  | rs74474130 | T | G | FALSE | 0.138 | -0.753 | 0.030 | 0.407 | 3.18E-06 | 0.064 | 21.702 |
|  | rs77885767 | C | T | FALSE | -0.156 | -0.187 | 0.034 | 0.353 | 3.44E-06 | 0.595 | 21.552 |
|  | rs7832304 | T | G | FALSE | -0.072 | -0.119 | 0.016 | 0.222 | 6.52E-06 | 0.591 | 20.330 |
|  | rs8130320 | A | G | FALSE | -0.049 | 0.065 | 0.011 | 0.150 | 4.67E-06 | 0.665 | 20.968 |
|  | rs9389714 | C | T | FALSE | -0.064 | 0.099 | 0.014 | 0.260 | 8.97E-06 | 0.703 | 19.718 |
|  | rs9578457 | G | A | FALSE | -0.141 | -0.213 | 0.032 | 0.347 | 7.49E-06 | 0.539 | 20.064 |
|  | rs9603208 | G | T | FALSE | 0.082 | 0.236 | 0.016 | 0.248 | 2.67E-07 | 0.340 | 26.474 |
| Family Ruminococcaceae(id.2050) | rs10093275 | C | T | FALSE | 0.053 | -0.038 | 0.012 | 0.158 | 4.69E-06 | 0.809 | 20.960 |
|  | rs10166469 | T | C | FALSE | -0.053 | 0.086 | 0.012 | 0.170 | 9.12E-06 | 0.612 | 19.686 |
|  | rs117677831 | G | A | FALSE | 0.098 | -0.049 | 0.019 | 0.268 | 1.86E-07 | 0.856 | 27.172 |
|  | rs1612733 | T | C | FALSE | 0.109 | -0.722 | 0.024 | 0.329 | 4.94E-06 | 0.028 | 20.862 |
|  | rs17376049 | T | C | FALSE | 0.085 | -0.525 | 0.017 | 0.236 | 8.22E-07 | 0.026 | 24.306 |
|  | rs2113833 | T | C | FALSE | 0.169 | -0.345 | 0.036 | 0.415 | 1.89E-06 | 0.405 | 22.701 |
|  | rs2426816 | T | A | TRUE | -0.048 | 0.104 | 0.011 | 0.149 | 5.92E-06 | 0.483 | 20.514 |
|  | rs3009418 | C | A | FALSE | 0.093 | -0.080 | 0.021 | 0.354 | 1.01E-05 | 0.822 | 19.489 |
|  | rs499272 | G | C | TRUE | 0.071 | 0.198 | 0.016 | 0.224 | 1.07E-05 | 0.377 | 19.382 |
|  | rs56199908 | T | C | FALSE | -0.199 | 0.730 | 0.041 | 0.331 | 1.20E-06 | 0.027 | 23.584 |
|  | rs76724913 | T | G | FALSE | 0.090 | -0.517 | 0.020 | 0.254 | 9.15E-06 | 0.042 | 19.682 |
| Family Streptococcaceae(id.1850) | rs11110281 | T | C | FALSE | -0.131 | 0.333 | 0.023 | 0.340 | 7.55E-09 | 0.328 | 33.387 |
|  | rs2952251 | A | G | FALSE | -0.064 | -0.105 | 0.013 | 0.189 | 4.35E-07 | 0.578 | 25.530 |
|  | rs4968759 | A | G | FALSE | -0.054 | -0.044 | 0.011 | 0.148 | 1.01E-06 | 0.766 | 23.905 |
| Family unknownfamily(id.1000001214) | rs11150282 | T | C | FALSE | 0.098 | 0.093 | 0.020 | 0.155 | 6.25E-07 | 0.549 | 24.834 |
|  | rs113884518 | T | C | FALSE | -0.206 | 0.068 | 0.046 | 0.471 | 6.13E-06 | 0.885 | 20.446 |
|  | rs1221147 | T | A | TRUE | 0.126 | 0.398 | 0.028 | 0.210 | 6.06E-06 | 0.058 | 20.471 |
|  | rs16851659 | G | C | TRUE | -0.090 | 0.055 | 0.019 | 0.156 | 1.60E-06 | 0.726 | 23.025 |
|  | rs28678345 | T | C | FALSE | 0.213 | -0.097 | 0.047 | 0.349 | 6.17E-06 | 0.780 | 20.434 |
|  | rs367480 | G | A | FALSE | -0.084 | 0.193 | 0.019 | 0.155 | 6.00E-06 | 0.214 | 20.487 |
|  | rs4129395 | G | A | FALSE | 0.090 | 0.261 | 0.019 | 0.149 | 1.05E-06 | 0.079 | 23.826 |
|  | rs73074665 | A | T | TRUE | 0.165 | -0.004 | 0.036 | 0.300 | 3.76E-06 | 0.990 | 21.381 |
|  | rs789069 | A | C | FALSE | -0.104 | 0.098 | 0.023 | 0.213 | 8.94E-06 | 0.645 | 19.725 |
|  | rs79790072 | T | C | FALSE | 0.226 | -0.139 | 0.049 | 0.429 | 3.60E-06 | 0.745 | 21.466 |
|  | rs8028558 | A | G | FALSE | 0.083 | 0.037 | 0.019 | 0.154 | 9.53E-06 | 0.811 | 19.603 |
|  | rs9864379 | T | C | FALSE | -0.161 | 0.513 | 0.029 | 0.210 | 4.18E-08 | 0.015 | 30.065 |
| Family unknownfamily(id.1000005471) | rs10071529 | G | C | TRUE | 0.125 | 0.034 | 0.028 | 0.241 | 7.15E-06 | 0.887 | 20.154 |
|  | rs114184530 | T | C | FALSE | -0.155 | 0.181 | 0.034 | 0.267 | 5.48E-06 | 0.496 | 20.662 |
|  | rs11779863 | G | A | FALSE | -0.077 | 0.043 | 0.017 | 0.206 | 7.18E-06 | 0.833 | 20.146 |
|  | rs12566890 | T | G | FALSE | -0.103 | 0.341 | 0.024 | 0.223 | 1.99E-05 | 0.126 | 18.196 |
|  | rs13100746 | C | T | FALSE | 0.064 | 0.144 | 0.014 | 0.149 | 7.53E-06 | 0.335 | 20.055 |
|  | rs17235252 | T | C | FALSE | -0.122 | -0.120 | 0.026 | 0.231 | 1.79E-06 | 0.603 | 22.808 |
|  | rs3932485 | C | T | FALSE | 0.063 | 0.161 | 0.014 | 0.152 | 9.79E-06 | 0.288 | 19.551 |
|  | rs515984 | T | C | FALSE | -0.088 | 0.101 | 0.019 | 0.232 | 4.41E-06 | 0.663 | 21.078 |
|  | rs62188991 | G | C | TRUE | -0.111 | 0.297 | 0.024 | 0.320 | 4.36E-06 | 0.353 | 21.099 |
|  | rs638542 | G | A | FALSE | -0.071 | 0.024 | 0.016 | 0.164 | 6.96E-06 | 0.886 | 20.204 |
|  | rs739151 | C | G | TRUE | 0.065 | -0.016 | 0.014 | 0.149 | 3.02E-06 | 0.913 | 21.804 |
|  | rs74603314 | T | C | FALSE | 0.231 | 0.190 | 0.049 | 0.380 | 2.45E-06 | 0.616 | 22.207 |
|  | rs7706512 | G | A | FALSE | 0.066 | 0.072 | 0.014 | 0.149 | 2.25E-06 | 0.626 | 22.371 |
|  | rs7801843 | A | G | FALSE | -0.087 | 0.072 | 0.019 | 0.208 | 7.81E-06 | 0.731 | 19.983 |
|  | rs949341 | G | A | FALSE | 0.066 | 0.087 | 0.015 | 0.166 | 8.19E-06 | 0.600 | 19.893 |
| Family unknownfamily(id.1000006161) | rs11251024 | G | A | FALSE | 0.104 | -0.317 | 0.021 | 0.168 | 4.65E-07 | 0.059 | 25.404 |
|  | rs11606187 | A | G | FALSE | -0.155 | -0.249 | 0.033 | 0.215 | 2.18E-06 | 0.248 | 22.432 |
|  | rs13219468 | G | C | TRUE | 0.115 | 0.221 | 0.024 | 0.165 | 1.13E-06 | 0.180 | 23.694 |
|  | rs13385922 | T | C | FALSE | 0.093 | 0.090 | 0.020 | 0.156 | 3.91E-06 | 0.564 | 21.311 |
|  | rs166849 | G | A | FALSE | 0.091 | -0.060 | 0.020 | 0.151 | 6.64E-06 | 0.693 | 20.293 |
|  | rs2172426 | C | T | FALSE | -0.102 | 0.140 | 0.020 | 0.152 | 2.85E-07 | 0.357 | 26.348 |
|  | rs267959 | A | G | FALSE | 0.099 | -0.035 | 0.021 | 0.166 | 2.42E-06 | 0.834 | 22.231 |
|  | rs2930903 | C | G | TRUE | 0.091 | 0.063 | 0.021 | 0.155 | 1.44E-05 | 0.684 | 18.810 |
|  | rs4383094 | T | C | FALSE | 0.149 | -0.026 | 0.032 | 0.215 | 3.28E-06 | 0.905 | 21.646 |
|  | rs55921101 | A | T | TRUE | -0.109 | 0.113 | 0.024 | 0.176 | 3.74E-06 | 0.519 | 21.392 |
|  | rs6051857 | A | G | FALSE | -0.134 | 0.017 | 0.029 | 0.193 | 4.55E-06 | 0.929 | 21.016 |
|  | rs60583455 | T | C | FALSE | 0.109 | 0.449 | 0.021 | 0.156 | 2.69E-07 | 0.004 | 26.460 |
|  | rs60775321 | T | C | FALSE | -0.096 | 0.041 | 0.021 | 0.165 | 6.77E-06 | 0.804 | 20.256 |
|  | rs72671304 | T | C | FALSE | 0.172 | 0.268 | 0.037 | 0.283 | 3.21E-06 | 0.344 | 21.688 |
|  | rs7911787 | G | T | FALSE | -0.223 | 0.035 | 0.047 | 0.422 | 2.10E-06 | 0.935 | 22.504 |
|  | rs9542068 | T | C | FALSE | 0.099 | 0.073 | 0.022 | 0.158 | 5.59E-06 | 0.642 | 20.625 |
| Family Veillonellaceae(id.2172) | rs111810795 | C | T | FALSE | -0.087 | -0.555 | 0.018 | 0.250 | 1.57E-06 | 0.026 | 23.059 |
|  | rs114889439 | A | G | FALSE | -0.254 | 0.306 | 0.054 | 0.408 | 2.51E-06 | 0.453 | 22.160 |
|  | rs11700976 | C | A | FALSE | 0.050 | 0.039 | 0.011 | 0.149 | 9.67E-06 | 0.795 | 19.576 |
|  | rs12186441 | G | A | FALSE | 0.208 | -0.647 | 0.045 | 0.371 | 4.57E-06 | 0.081 | 21.010 |
|  | rs12668619 | A | G | FALSE | 0.055 | 0.005 | 0.012 | 0.159 | 2.60E-06 | 0.976 | 22.093 |
|  | rs12741784 | C | T | FALSE | -0.062 | -0.064 | 0.012 | 0.170 | 1.88E-07 | 0.708 | 27.151 |
|  | rs1442060 | G | A | FALSE | -0.051 | -0.166 | 0.011 | 0.150 | 4.55E-06 | 0.269 | 21.017 |
|  | rs1447205 | G | C | TRUE | -0.052 | -0.027 | 0.012 | 0.155 | 6.73E-06 | 0.860 | 20.269 |
|  | rs1693340 | T | C | FALSE | 0.082 | 0.354 | 0.018 | 0.288 | 7.17E-06 | 0.220 | 20.147 |
|  | rs2175069 | A | G | FALSE | -0.053 | 0.183 | 0.011 | 0.153 | 4.54E-06 | 0.233 | 21.022 |
|  | rs2561116 | T | G | FALSE | -0.084 | -0.448 | 0.019 | 0.316 | 7.91E-06 | 0.157 | 19.960 |
|  | rs2585520 | G | T | FALSE | -0.090 | -0.371 | 0.020 | 0.381 | 6.33E-06 | 0.330 | 20.387 |
|  | rs4263802 | G | A | FALSE | 0.051 | 0.180 | 0.011 | 0.158 | 9.30E-06 | 0.253 | 19.649 |
|  | rs4461038 | A | G | FALSE | -0.055 | 0.059 | 0.012 | 0.163 | 3.40E-06 | 0.717 | 21.579 |
|  | rs4797169 | T | C | FALSE | 0.059 | -0.045 | 0.013 | 0.174 | 4.62E-06 | 0.797 | 20.991 |
|  | rs6692542 | A | G | FALSE | 0.053 | 0.007 | 0.012 | 0.157 | 6.21E-06 | 0.966 | 20.422 |
|  | rs6909981 | C | T | FALSE | -0.064 | 0.258 | 0.014 | 0.224 | 6.63E-06 | 0.250 | 20.298 |
|  | rs73557284 | A | G | FALSE | 0.205 | -0.153 | 0.047 | 0.253 | 1.12E-05 | 0.545 | 19.292 |
|  | rs75768969 | C | G | TRUE | -0.070 | -0.027 | 0.016 | 0.264 | 7.23E-06 | 0.918 | 20.131 |
|  | rs79535861 | A | C | FALSE | 0.101 | -0.367 | 0.021 | 0.259 | 1.16E-06 | 0.157 | 23.646 |
|  | rs9345168 | C | A | FALSE | 0.051 | 0.206 | 0.011 | 0.150 | 6.95E-06 | 0.169 | 20.207 |
| Family Verrucomicrobiaceae(id.4036) | rs11184341 | G | C | TRUE | 0.066 | 0.093 | 0.014 | 0.167 | 4.08E-06 | 0.577 | 21.227 |
|  | rs111862613 | T | C | FALSE | 0.091 | -0.015 | 0.020 | 0.203 | 4.02E-06 | 0.943 | 21.255 |
|  | rs117107102 | A | G | FALSE | 0.205 | 0.031 | 0.043 | 0.349 | 2.11E-06 | 0.928 | 22.493 |
|  | rs11729256 | T | C | FALSE | 0.075 | 0.259 | 0.015 | 0.197 | 5.95E-07 | 0.188 | 24.928 |
|  | rs12908520 | G | A | FALSE | 0.062 | -0.022 | 0.013 | 0.150 | 2.27E-06 | 0.885 | 22.353 |
|  | rs2602429 | C | T | FALSE | 0.075 | 0.054 | 0.016 | 0.170 | 1.82E-06 | 0.753 | 22.781 |
|  | rs4242783 | G | A | FALSE | 0.069 | 0.033 | 0.015 | 0.167 | 3.19E-06 | 0.843 | 21.699 |
|  | rs4936098 | A | G | FALSE | 0.065 | -0.126 | 0.014 | 0.156 | 1.82E-06 | 0.420 | 22.775 |
|  | rs61779207 | G | A | FALSE | -0.076 | -0.065 | 0.017 | 0.180 | 6.09E-06 | 0.718 | 20.459 |
|  | rs74542928 | T | C | FALSE | 0.112 | -0.234 | 0.024 | 0.343 | 2.11E-06 | 0.496 | 22.492 |
|  | rs9349825 | A | G | FALSE | -0.070 | 0.172 | 0.015 | 0.189 | 1.69E-06 | 0.362 | 22.919 |
|  | rs941682 | G | A | FALSE | -0.063 | -0.047 | 0.014 | 0.167 | 1.12E-05 | 0.777 | 19.296 |
| Family Victivallaceae(id.2255) | rs11671100 | A | C | FALSE | -0.160 | 0.108 | 0.035 | 0.187 | 4.66E-06 | 0.564 | 20.970 |
|  | rs2546105 | A | T | TRUE | 0.127 | 0.204 | 0.026 | 0.172 | 8.41E-07 | 0.236 | 24.262 |
|  | rs2944282 | T | C | FALSE | -0.124 | -0.462 | 0.026 | 0.166 | 1.35E-06 | 0.005 | 23.349 |
|  | rs34296005 | T | A | TRUE | 0.128 | -0.015 | 0.026 | 0.162 | 6.77E-07 | 0.926 | 24.679 |
|  | rs34962571 | A | C | FALSE | -0.187 | -0.185 | 0.042 | 0.249 | 7.90E-06 | 0.458 | 19.963 |
|  | rs4396289 | C | T | FALSE | -0.153 | 0.315 | 0.029 | 0.220 | 1.16E-07 | 0.151 | 28.090 |
|  | rs61702987 | T | C | FALSE | 0.146 | 0.032 | 0.030 | 0.237 | 1.24E-06 | 0.891 | 23.522 |
|  | rs62570196 | C | T | FALSE | -0.246 | -0.062 | 0.048 | 0.369 | 3.50E-07 | 0.867 | 25.953 |
|  | rs6545794 | A | G | FALSE | -0.198 | 0.014 | 0.041 | 0.228 | 1.45E-06 | 0.950 | 23.215 |
|  | rs67832247 | C | T | FALSE | -0.123 | -0.133 | 0.027 | 0.161 | 3.65E-06 | 0.410 | 21.440 |
|  | rs7077363 | G | A | FALSE | 0.149 | 0.343 | 0.032 | 0.202 | 2.89E-06 | 0.089 | 21.887 |
|  | rs7314815 | G | A | FALSE | 0.101 | 0.202 | 0.023 | 0.150 | 7.43E-06 | 0.177 | 20.079 |
|  | rs7627405 | C | T | FALSE | -0.134 | -0.139 | 0.030 | 0.189 | 8.56E-06 | 0.462 | 19.809 |
|  | rs7860510 | G | C | TRUE | 0.165 | 0.408 | 0.038 | 0.279 | 1.11E-05 | 0.144 | 19.313 |
| Class Alphaproteobacteria(id.2379) | rs10803434 | A | T | TRUE | -0.069 | 0.170 | 0.015 | 0.149 | 3.26E-06 | 0.253 | 21.655 |
|  | rs12977163 | G | C | TRUE | 0.069 | -0.191 | 0.015 | 0.153 | 7.22E-06 | 0.213 | 20.133 |
|  | rs140912403 | C | T | FALSE | -0.161 | 0.145 | 0.032 | 0.319 | 4.25E-07 | 0.649 | 25.577 |
|  | rs17061716 | G | C | TRUE | 0.093 | 0.001 | 0.021 | 0.203 | 7.91E-06 | 0.997 | 19.960 |
|  | rs34569731 | A | G | FALSE | 0.071 | 0.046 | 0.016 | 0.155 | 7.63E-06 | 0.769 | 20.028 |
|  | rs55876211 | C | T | FALSE | -0.081 | 0.002 | 0.018 | 0.171 | 1.14E-05 | 0.989 | 19.255 |
|  | rs62285697 | C | T | FALSE | 0.081 | -0.042 | 0.018 | 0.173 | 8.54E-06 | 0.811 | 19.812 |
|  | rs76784716 | A | G | FALSE | 0.133 | 0.104 | 0.027 | 0.237 | 6.43E-07 | 0.661 | 24.777 |
|  | rs7960664 | G | A | FALSE | 0.097 | -0.194 | 0.022 | 0.265 | 6.60E-06 | 0.463 | 20.305 |
|  | rs9813022 | A | G | FALSE | -0.075 | -0.169 | 0.015 | 0.154 | 1.05E-06 | 0.273 | 23.840 |
| Class Bacilli(id.1673) | rs10034695 | C | T | FALSE | -0.104 | 0.293 | 0.025 | 0.234 | 2.52E-05 | 0.211 | 17.750 |
|  | rs11110282 | A | G | FALSE | -0.101 | 0.332 | 0.022 | 0.340 | 3.24E-06 | 0.329 | 21.669 |
|  | rs111552159 | C | G | TRUE | 0.128 | 0.411 | 0.029 | 0.362 | 8.11E-06 | 0.257 | 19.912 |
|  | rs11730038 | G | A | FALSE | -0.063 | -0.069 | 0.013 | 0.165 | 9.57E-07 | 0.673 | 24.012 |
|  | rs12797734 | T | C | FALSE | 0.057 | 0.333 | 0.013 | 0.173 | 6.41E-06 | 0.054 | 20.363 |
|  | rs13068444 | A | G | FALSE | 0.060 | 0.165 | 0.014 | 0.194 | 1.05E-05 | 0.396 | 19.414 |
|  | rs1595463 | C | A | FALSE | 0.048 | -0.171 | 0.011 | 0.150 | 1.12E-05 | 0.255 | 19.300 |
|  | rs1962325 | C | G | TRUE | 0.055 | 0.110 | 0.011 | 0.157 | 1.38E-06 | 0.484 | 23.311 |
|  | rs28564647 | T | G | FALSE | -0.061 | 0.303 | 0.014 | 0.200 | 8.36E-06 | 0.129 | 19.855 |
|  | rs2952251 | A | G | FALSE | -0.060 | -0.105 | 0.012 | 0.189 | 1.34E-06 | 0.578 | 23.361 |
|  | rs34989881 | A | G | FALSE | 0.111 | 0.001 | 0.025 | 0.346 | 6.37E-06 | 0.998 | 20.374 |
|  | rs35344081 | G | A | FALSE | 0.062 | -0.238 | 0.013 | 0.168 | 1.08E-06 | 0.156 | 23.772 |
|  | rs4028634 | T | C | FALSE | 0.052 | -0.177 | 0.011 | 0.154 | 2.14E-06 | 0.252 | 22.465 |
|  | rs4459992 | T | C | FALSE | 0.054 | 0.224 | 0.012 | 0.158 | 4.12E-06 | 0.157 | 21.207 |
|  | rs57872228 | C | T | FALSE | -0.071 | -0.095 | 0.015 | 0.229 | 1.09E-06 | 0.678 | 23.757 |
|  | rs694949 | A | G | FALSE | -0.081 | -0.398 | 0.018 | 0.251 | 6.57E-06 | 0.114 | 20.314 |
|  | rs74352383 | T | A | TRUE | -0.104 | -0.584 | 0.022 | 0.270 | 1.48E-06 | 0.031 | 23.171 |
|  | rs74663707 | C | T | FALSE | 0.098 | -0.111 | 0.022 | 0.303 | 1.24E-05 | 0.715 | 19.095 |
|  | rs76717940 | T | A | TRUE | 0.156 | -0.095 | 0.033 | 0.328 | 1.62E-06 | 0.772 | 23.004 |
|  | rs77558518 | A | G | FALSE | -0.107 | 0.023 | 0.022 | 0.254 | 1.49E-06 | 0.929 | 23.158 |
|  | rs78938557 | T | C | FALSE | 0.108 | 0.077 | 0.023 | 0.439 | 3.51E-06 | 0.861 | 21.514 |
|  | rs9581006 | C | T | FALSE | 0.225 | 0.294 | 0.047 | 0.386 | 1.45E-06 | 0.447 | 23.207 |
| Class Bacteroidia(id.912) | rs11146701 | A | G | FALSE | 0.047 | -0.156 | 0.011 | 0.194 | 7.03E-06 | 0.421 | 20.186 |
|  | rs12938514 | A | G | FALSE | -0.200 | 0.540 | 0.042 | 0.876 | 1.53E-06 | 0.537 | 23.106 |
|  | rs13291434 | T | C | FALSE | 0.069 | 0.037 | 0.015 | 0.214 | 3.53E-06 | 0.862 | 21.507 |
|  | rs17343978 | A | C | FALSE | -0.055 | 0.028 | 0.012 | 0.184 | 4.44E-06 | 0.879 | 21.067 |
|  | rs2032750 | T | C | FALSE | -0.051 | -0.005 | 0.011 | 0.150 | 1.94E-06 | 0.974 | 22.657 |
|  | rs4916508 | G | A | FALSE | -0.047 | -0.067 | 0.011 | 0.150 | 9.34E-06 | 0.655 | 19.641 |
|  | rs55773148 | G | A | FALSE | -0.122 | -0.513 | 0.024 | 0.330 | 2.86E-07 | 0.120 | 26.341 |
|  | rs62531359 | T | G | FALSE | 0.066 | -0.009 | 0.015 | 0.196 | 1.22E-05 | 0.964 | 19.138 |
|  | rs62575403 | C | T | FALSE | 0.140 | -0.474 | 0.031 | 0.378 | 6.75E-06 | 0.210 | 20.264 |
|  | rs72706335 | T | C | FALSE | -0.222 | 0.354 | 0.049 | 0.476 | 6.57E-06 | 0.457 | 20.315 |
|  | rs73846128 | A | G | FALSE | -0.065 | 0.129 | 0.013 | 0.208 | 1.18E-06 | 0.535 | 23.602 |
|  | rs7546249 | T | A | TRUE | -0.057 | -0.247 | 0.012 | 0.163 | 1.66E-06 | 0.130 | 22.948 |
|  | rs79585701 | A | C | FALSE | 0.065 | 0.242 | 0.015 | 0.213 | 1.54E-05 | 0.257 | 18.687 |
|  | rs929878 | C | T | FALSE | -0.055 | 0.212 | 0.012 | 0.182 | 6.37E-06 | 0.243 | 20.372 |
| Class Betaproteobacteria(id.2867) | rs11128180 | A | G | FALSE | 0.059 | -0.223 | 0.013 | 0.176 | 4.13E-06 | 0.204 | 21.203 |
|  | rs12467854 | G | C | TRUE | 0.064 | 0.083 | 0.013 | 0.183 | 1.13E-06 | 0.648 | 23.692 |
|  | rs1511453 | A | G | FALSE | 0.092 | -0.344 | 0.020 | 0.321 | 3.54E-06 | 0.284 | 21.502 |
|  | rs1928341 | A | G | FALSE | 0.053 | 0.027 | 0.011 | 0.151 | 1.93E-06 | 0.859 | 22.661 |
|  | rs2321387 | G | A | FALSE | -0.049 | 0.274 | 0.011 | 0.150 | 6.18E-06 | 0.069 | 20.430 |
|  | rs2367850 | G | C | TRUE | 0.064 | -0.053 | 0.013 | 0.183 | 9.14E-07 | 0.772 | 24.102 |
|  | rs2613606 | C | T | FALSE | -0.051 | -0.032 | 0.011 | 0.150 | 2.56E-06 | 0.834 | 22.123 |
|  | rs320161 | A | G | FALSE | 0.057 | 0.376 | 0.013 | 0.175 | 5.77E-06 | 0.032 | 20.564 |
|  | rs4033856 | C | T | FALSE | 0.083 | -0.363 | 0.017 | 0.264 | 6.44E-07 | 0.169 | 24.776 |
|  | rs56386628 | C | T | FALSE | -0.062 | 0.179 | 0.014 | 0.171 | 4.52E-06 | 0.297 | 21.031 |
|  | rs6087811 | T | G | FALSE | -0.098 | -0.313 | 0.020 | 0.249 | 8.34E-07 | 0.208 | 24.278 |
|  | rs62395635 | T | C | FALSE | 0.110 | -0.088 | 0.024 | 0.315 | 3.32E-06 | 0.780 | 21.623 |
|  | rs72747231 | C | G | TRUE | -0.137 | -0.280 | 0.029 | 0.353 | 2.26E-06 | 0.426 | 22.357 |
|  | rs75242906 | C | T | FALSE | -0.121 | -0.383 | 0.028 | 0.270 | 1.61E-05 | 0.156 | 18.601 |
| Class Clostridia(id.1859) | rs10774377 | A | G | FALSE | 0.053 | -0.043 | 0.011 | 0.150 | 3.75E-06 | 0.774 | 21.390 |
|  | rs112334273 | G | A | FALSE | 0.064 | -0.106 | 0.013 | 0.166 | 4.87E-07 | 0.524 | 25.314 |
|  | rs13105690 | T | C | FALSE | -0.053 | 0.019 | 0.012 | 0.166 | 7.57E-06 | 0.908 | 20.044 |
|  | rs13179700 | T | C | FALSE | 0.051 | -0.151 | 0.011 | 0.157 | 2.98E-06 | 0.334 | 21.830 |
|  | rs13421739 | C | G | TRUE | 0.089 | 0.484 | 0.018 | 0.294 | 7.60E-07 | 0.100 | 24.457 |
|  | rs1842454 | G | A | FALSE | -0.055 | 0.041 | 0.013 | 0.191 | 1.73E-05 | 0.830 | 18.466 |
|  | rs2273429 | A | G | FALSE | -0.072 | -0.054 | 0.015 | 0.243 | 2.26E-06 | 0.824 | 22.364 |
|  | rs3792064 | G | A | FALSE | 0.080 | -0.280 | 0.018 | 0.329 | 1.31E-05 | 0.395 | 19.003 |
|  | rs6126494 | G | T | FALSE | -0.097 | -0.294 | 0.021 | 0.311 | 4.67E-06 | 0.344 | 20.967 |
|  | rs6797343 | G | T | FALSE | 0.059 | 0.285 | 0.013 | 0.188 | 1.07E-05 | 0.129 | 19.375 |
|  | rs6814436 | T | C | FALSE | 0.074 | -0.265 | 0.015 | 0.213 | 9.27E-07 | 0.214 | 24.075 |
|  | rs6815608 | T | C | FALSE | 0.104 | 0.248 | 0.021 | 0.208 | 8.55E-07 | 0.233 | 24.229 |
|  | rs6934062 | G | C | TRUE | 0.052 | -0.097 | 0.012 | 0.159 | 6.29E-06 | 0.543 | 20.396 |
|  | rs72738886 | T | C | FALSE | 0.087 | 0.129 | 0.019 | 0.279 | 5.38E-06 | 0.643 | 20.697 |
|  | rs72915163 | T | C | FALSE | -0.058 | -1.204 | 0.012 | 3.983 | 1.44E-06 | 0.763 | 23.227 |
|  | rs72915163 | T | C | FALSE | -0.058 | -0.135 | 0.012 | 0.182 | 1.44E-06 | 0.459 | 23.227 |
|  | rs76860606 | C | G | TRUE | 0.095 | 0.175 | 0.023 | 0.267 | 2.59E-05 | 0.512 | 17.698 |
| Class Coriobacteriia(id.809) | rs11250875 | T | C | FALSE | 0.061 | 0.342 | 0.013 | 0.180 | 3.49E-06 | 0.057 | 21.526 |
|  | rs11656361 | A | C | FALSE | 0.077 | -0.071 | 0.018 | 0.194 | 1.06E-05 | 0.713 | 19.394 |
|  | rs12974142 | G | A | FALSE | 0.079 | 0.502 | 0.018 | 0.290 | 8.31E-06 | 0.083 | 19.865 |
|  | rs13307134 | C | T | FALSE | 0.057 | 0.016 | 0.013 | 0.199 | 7.46E-06 | 0.937 | 20.072 |
|  | rs1397793 | G | A | FALSE | -0.050 | -0.063 | 0.011 | 0.164 | 9.15E-06 | 0.701 | 19.681 |
|  | rs1816223 | A | G | FALSE | -0.059 | -0.186 | 0.013 | 0.186 | 5.51E-06 | 0.318 | 20.652 |
|  | rs240104 | T | C | FALSE | -0.060 | 0.054 | 0.013 | 0.166 | 1.96E-06 | 0.745 | 22.630 |
|  | rs2442778 | G | A | FALSE | -0.116 | -0.140 | 0.026 | 0.338 | 6.72E-06 | 0.678 | 20.272 |
|  | rs3025411 | A | G | FALSE | 0.093 | 0.020 | 0.021 | 0.245 | 9.72E-06 | 0.934 | 19.566 |
|  | rs34739816 | G | T | FALSE | 0.097 | -0.171 | 0.021 | 0.317 | 3.37E-06 | 0.590 | 21.594 |
|  | rs62448869 | T | A | TRUE | -0.049 | -0.081 | 0.011 | 0.150 | 8.20E-06 | 0.588 | 19.891 |
|  | rs67561917 | A | G | FALSE | -0.071 | 0.086 | 0.015 | 0.194 | 3.56E-06 | 0.659 | 21.486 |
|  | rs719099 | A | G | FALSE | 0.078 | 0.104 | 0.016 | 0.251 | 5.86E-07 | 0.680 | 24.957 |
|  | rs76779974 | C | G | TRUE | 0.077 | -0.398 | 0.017 | 0.254 | 6.53E-06 | 0.118 | 20.326 |
|  | rs80046645 | C | G | TRUE | 0.256 | -0.621 | 0.056 | 0.380 | 5.31E-06 | 0.102 | 20.724 |
|  | rs8010111 | G | A | FALSE | -0.103 | -0.082 | 0.023 | 0.268 | 6.52E-06 | 0.759 | 20.328 |
| Class Deltaproteobacteria(id.3087) | rs112381107 | C | T | FALSE | 0.207 | -0.084 | 0.046 | 0.314 | 6.14E-06 | 0.790 | 20.445 |
|  | rs11599763 | T | C | FALSE | -0.054 | -0.032 | 0.012 | 0.153 | 3.56E-06 | 0.836 | 21.490 |
|  | rs16851319 | G | C | TRUE | -0.071 | -0.244 | 0.015 | 0.197 | 2.91E-06 | 0.217 | 21.874 |
|  | rs17084793 | G | A | FALSE | -0.071 | 0.273 | 0.016 | 0.210 | 8.38E-06 | 0.194 | 19.850 |
|  | rs17791387 | A | G | FALSE | -0.074 | 0.104 | 0.015 | 0.253 | 1.83E-06 | 0.680 | 22.761 |
|  | rs2692012 | A | G | FALSE | 0.110 | -0.302 | 0.025 | 0.329 | 1.33E-05 | 0.359 | 18.968 |
|  | rs2838334 | G | A | FALSE | 0.056 | 0.038 | 0.012 | 0.156 | 6.03E-06 | 0.809 | 20.480 |
|  | rs3935584 | C | T | FALSE | -0.052 | -0.259 | 0.012 | 0.149 | 6.01E-06 | 0.083 | 20.485 |
|  | rs4506934 | C | T | FALSE | -0.094 | -0.189 | 0.020 | 0.232 | 3.25E-06 | 0.415 | 21.666 |
|  | rs55744759 | A | G | FALSE | -0.078 | -0.039 | 0.017 | 0.234 | 4.96E-06 | 0.867 | 20.854 |
|  | rs6058181 | C | T | FALSE | 0.083 | -0.012 | 0.017 | 0.202 | 6.51E-07 | 0.954 | 24.755 |
|  | rs62020470 | A | G | FALSE | -0.059 | -0.161 | 0.013 | 0.193 | 6.03E-06 | 0.405 | 20.480 |
|  | rs7936267 | A | G | FALSE | -0.056 | 0.253 | 0.012 | 0.155 | 5.29E-06 | 0.104 | 20.731 |
|  | rs9928243 | C | A | FALSE | -0.054 | -0.102 | 0.012 | 0.149 | 4.78E-06 | 0.495 | 20.923 |
| Class Erysipelotrichia(id.2147) | rs10781552 | C | T | FALSE | -0.055 | 0.080 | 0.012 | 0.165 | 1.96E-06 | 0.629 | 22.633 |
|  | rs17530232 | A | G | FALSE | 0.103 | 0.276 | 0.022 | 0.333 | 4.49E-06 | 0.407 | 21.042 |
|  | rs1884466 | C | T | FALSE | -0.048 | 0.294 | 0.011 | 0.149 | 8.78E-06 | 0.049 | 19.760 |
|  | rs2300774 | G | A | FALSE | 0.052 | -0.113 | 0.011 | 0.150 | 9.18E-07 | 0.448 | 24.094 |
|  | rs290833 | T | G | FALSE | -0.050 | -0.020 | 0.011 | 0.150 | 7.98E-06 | 0.894 | 19.942 |
|  | rs35161940 | T | C | FALSE | -0.081 | 0.079 | 0.017 | 0.241 | 1.52E-06 | 0.742 | 23.118 |
|  | rs4078432 | C | T | FALSE | -0.061 | -0.184 | 0.013 | 0.198 | 5.31E-06 | 0.354 | 20.723 |
|  | rs56970041 | T | G | FALSE | 0.072 | -0.096 | 0.016 | 0.308 | 1.07E-05 | 0.756 | 19.385 |
|  | rs62504403 | C | T | FALSE | 0.068 | -0.039 | 0.013 | 0.188 | 1.00E-07 | 0.837 | 28.371 |
|  | rs7234058 | T | C | FALSE | -0.095 | 0.383 | 0.019 | 0.260 | 1.10E-06 | 0.141 | 23.744 |
|  | rs7826267 | T | G | FALSE | -0.084 | -0.081 | 0.020 | 0.305 | 2.51E-05 | 0.791 | 17.755 |
|  | rs8003149 | C | T | FALSE | 0.054 | 0.021 | 0.012 | 0.158 | 4.04E-06 | 0.896 | 21.248 |
|  | rs921306 | T | C | FALSE | 0.049 | 0.144 | 0.011 | 0.153 | 6.02E-06 | 0.345 | 20.481 |
| Class Gammaproteobacteria(id.3303) | rs11181912 | G | A | FALSE | -0.058 | 0.003 | 0.012 | 0.159 | 1.09E-06 | 0.983 | 23.767 |
|  | rs12404135 | A | G | FALSE | -0.079 | -0.402 | 0.017 | 0.277 | 4.76E-06 | 0.146 | 20.933 |
|  | rs6469506 | T | A | TRUE | 0.054 | -0.185 | 0.012 | 0.158 | 3.25E-06 | 0.241 | 21.661 |
|  | rs6706173 | A | C | FALSE | 0.074 | 0.215 | 0.015 | 0.214 | 3.94E-07 | 0.316 | 25.726 |
|  | rs75101789 | C | T | FALSE | 0.073 | 0.272 | 0.016 | 0.263 | 7.73E-06 | 0.300 | 20.002 |
|  | rs79795896 | A | G | FALSE | -0.159 | -0.090 | 0.035 | 0.349 | 5.87E-06 | 0.796 | 20.531 |
|  | rs9494710 | C | T | FALSE | -0.055 | -0.223 | 0.012 | 0.160 | 5.48E-06 | 0.163 | 20.663 |
|  | rs9973122 | T | A | TRUE | 0.074 | -0.241 | 0.016 | 0.268 | 5.24E-06 | 0.369 | 20.747 |
| Class Lentisphaeria(id.2250) | rs1002941 | G | A | FALSE | 0.105 | 0.042 | 0.023 | 0.173 | 6.85E-06 | 0.808 | 20.233 |
|  | rs11770843 | C | T | FALSE | 0.109 | -0.123 | 0.023 | 0.160 | 3.18E-06 | 0.441 | 21.707 |
|  | rs17114848 | G | A | FALSE | 0.152 | -0.239 | 0.032 | 0.250 | 2.62E-06 | 0.338 | 22.073 |
|  | rs2031282 | A | G | FALSE | 0.122 | 0.263 | 0.027 | 0.195 | 5.99E-06 | 0.179 | 20.491 |
|  | rs2731834 | C | G | TRUE | 0.109 | 0.246 | 0.024 | 0.174 | 3.86E-06 | 0.157 | 21.335 |
|  | rs2825714 | A | G | FALSE | -0.137 | -0.133 | 0.029 | 0.199 | 2.03E-06 | 0.504 | 22.569 |
|  | rs62570196 | C | T | FALSE | -0.216 | -0.062 | 0.044 | 0.369 | 8.72E-07 | 0.867 | 24.192 |
|  | rs72640280 | A | G | FALSE | 0.220 | 0.498 | 0.049 | 0.323 | 5.92E-06 | 0.123 | 20.513 |
|  | rs73113483 | T | A | TRUE | -0.131 | 0.233 | 0.029 | 0.229 | 5.50E-06 | 0.308 | 20.656 |
|  | rs77599476 | A | G | FALSE | 0.230 | 0.068 | 0.048 | 0.322 | 1.62E-06 | 0.833 | 23.002 |
| Class Melainabacteria(id.1589) | rs10148250 | G | A | FALSE | 0.086 | -0.034 | 0.019 | 0.158 | 8.31E-06 | 0.831 | 19.866 |
|  | rs10738747 | G | A | FALSE | 0.081 | 0.018 | 0.018 | 0.150 | 9.99E-06 | 0.903 | 19.514 |
|  | rs11150282 | T | C | FALSE | 0.099 | 0.093 | 0.020 | 0.155 | 5.07E-07 | 0.549 | 25.236 |
|  | rs113884518 | T | C | FALSE | -0.205 | 0.068 | 0.045 | 0.471 | 6.38E-06 | 0.885 | 20.371 |
|  | rs1221147 | T | A | TRUE | 0.124 | 0.398 | 0.028 | 0.210 | 8.83E-06 | 0.058 | 19.749 |
|  | rs16851659 | G | C | TRUE | -0.090 | 0.055 | 0.019 | 0.156 | 1.54E-06 | 0.726 | 23.103 |
|  | rs28678345 | T | C | FALSE | 0.215 | -0.097 | 0.047 | 0.349 | 5.05E-06 | 0.780 | 20.818 |
|  | rs367480 | G | A | FALSE | -0.084 | 0.193 | 0.019 | 0.155 | 6.54E-06 | 0.214 | 20.323 |
|  | rs4129395 | G | A | FALSE | 0.090 | 0.261 | 0.019 | 0.149 | 1.29E-06 | 0.079 | 23.440 |
|  | rs73074665 | A | T | TRUE | 0.166 | -0.004 | 0.036 | 0.300 | 2.97E-06 | 0.990 | 21.833 |
|  | rs789069 | A | C | FALSE | -0.104 | 0.098 | 0.023 | 0.213 | 9.90E-06 | 0.645 | 19.531 |
|  | rs79790072 | T | C | FALSE | 0.227 | -0.139 | 0.049 | 0.429 | 3.36E-06 | 0.745 | 21.599 |
|  | rs9864379 | T | C | FALSE | -0.160 | 0.513 | 0.029 | 0.210 | 4.76E-08 | 0.015 | 29.812 |
| Class Methanobacteria(id.119) | rs10202904 | T | G | FALSE | -0.122 | 0.015 | 0.024 | 0.151 | 2.30E-07 | 0.923 | 26.761 |
|  | rs10424197 | G | A | FALSE | -0.111 | -0.129 | 0.025 | 0.171 | 6.94E-06 | 0.451 | 20.211 |
|  | rs11018665 | A | T | TRUE | 0.111 | -0.159 | 0.025 | 0.173 | 8.61E-06 | 0.357 | 19.797 |
|  | rs12825290 | C | G | TRUE | -0.217 | -0.013 | 0.049 | 0.317 | 1.12E-05 | 0.967 | 19.296 |
|  | rs4257531 | G | A | FALSE | 0.164 | -0.412 | 0.036 | 0.244 | 6.57E-06 | 0.091 | 20.316 |
|  | rs56131665 | G | A | FALSE | 0.179 | -0.057 | 0.039 | 0.243 | 5.46E-06 | 0.813 | 20.669 |
|  | rs62241835 | G | T | FALSE | -0.203 | -0.757 | 0.042 | 0.519 | 1.14E-06 | 0.145 | 23.683 |
|  | rs6508769 | T | C | FALSE | 0.154 | 0.058 | 0.034 | 0.209 | 8.35E-06 | 0.783 | 19.856 |
|  | rs73068003 | G | T | FALSE | -0.158 | 0.133 | 0.035 | 0.250 | 6.95E-06 | 0.594 | 20.206 |
|  | rs73457410 | A | G | FALSE | 0.215 | 0.466 | 0.044 | 0.301 | 8.18E-07 | 0.122 | 24.316 |
|  | rs75208022 | C | T | FALSE | -0.227 | 0.103 | 0.049 | 0.253 | 3.16E-06 | 0.684 | 21.717 |
|  | rs894996 | C | A | FALSE | 0.217 | -0.519 | 0.045 | 0.286 | 1.35E-06 | 0.069 | 23.349 |
| Class Mollicutes(id.3920) | rs10108398 | G | A | FALSE | 0.077 | 0.138 | 0.015 | 0.166 | 5.85E-07 | 0.406 | 24.960 |
|  | rs12566890 | T | G | FALSE | -0.101 | 0.341 | 0.023 | 0.223 | 1.19E-05 | 0.126 | 19.176 |
|  | rs17214486 | C | A | FALSE | 0.061 | -0.128 | 0.014 | 0.159 | 6.89E-06 | 0.420 | 20.223 |
|  | rs2464826 | A | C | FALSE | 0.094 | -0.067 | 0.021 | 0.237 | 8.27E-06 | 0.778 | 19.874 |
|  | rs28537087 | G | A | FALSE | 0.082 | -0.050 | 0.019 | 0.173 | 1.31E-05 | 0.775 | 19.002 |
|  | rs3768491 | A | G | FALSE | -0.068 | 0.020 | 0.015 | 0.164 | 4.90E-06 | 0.902 | 20.875 |
|  | rs4885016 | T | C | FALSE | -0.082 | 0.210 | 0.018 | 0.221 | 6.41E-06 | 0.343 | 20.363 |
|  | rs6043847 | T | C | FALSE | -0.115 | 0.138 | 0.025 | 0.318 | 3.78E-06 | 0.664 | 21.375 |
|  | rs67374027 | A | T | TRUE | 0.075 | 0.193 | 0.015 | 0.174 | 1.17E-06 | 0.268 | 23.634 |
|  | rs72901605 | T | C | FALSE | -0.084 | -0.011 | 0.018 | 0.234 | 2.29E-06 | 0.964 | 22.338 |
|  | rs74603314 | T | C | FALSE | 0.222 | 0.190 | 0.046 | 0.380 | 1.69E-06 | 0.616 | 22.924 |
|  | rs78169027 | A | G | FALSE | -0.108 | -0.344 | 0.024 | 0.314 | 5.03E-06 | 0.274 | 20.824 |
| Class Negativicutes(id.2164) | rs10787507 | G | C | TRUE | -0.055 | 0.024 | 0.013 | 0.190 | 1.78E-05 | 0.900 | 18.411 |
|  | rs1135612 | G | A | FALSE | 0.053 | -0.111 | 0.012 | 0.182 | 8.77E-06 | 0.542 | 19.761 |
|  | rs13086907 | G | A | FALSE | 0.063 | 0.205 | 0.013 | 0.180 | 2.07E-06 | 0.256 | 22.532 |
|  | rs1447205 | G | C | TRUE | -0.050 | -0.027 | 0.011 | 0.155 | 3.62E-06 | 0.860 | 21.458 |
|  | rs1643968 | T | C | FALSE | -0.057 | 0.029 | 0.011 | 0.155 | 4.81E-07 | 0.852 | 25.339 |
|  | rs1649999 | A | G | FALSE | 0.075 | -0.347 | 0.017 | 0.252 | 6.81E-06 | 0.169 | 20.246 |
|  | rs2834062 | A | G | FALSE | 0.049 | -0.140 | 0.011 | 0.163 | 7.01E-06 | 0.392 | 20.190 |
|  | rs4722181 | T | G | FALSE | 0.050 | -0.085 | 0.011 | 0.149 | 2.15E-06 | 0.568 | 22.452 |
|  | rs60274479 | T | C | FALSE | -0.066 | -0.195 | 0.013 | 0.189 | 8.76E-07 | 0.303 | 24.182 |
|  | rs61249479 | A | C | FALSE | 0.078 | -0.290 | 0.017 | 0.206 | 4.06E-06 | 0.160 | 21.236 |
|  | rs71405394 | G | A | FALSE | -0.114 | -0.307 | 0.024 | 0.295 | 2.06E-06 | 0.298 | 22.539 |
|  | rs73232831 | G | A | FALSE | -0.152 | 0.657 | 0.031 | 0.398 | 1.43E-06 | 0.098 | 23.242 |
|  | rs9423647 | G | A | FALSE | 0.048 | 0.175 | 0.011 | 0.150 | 5.58E-06 | 0.242 | 20.628 |
| Class Verrucomicrobiae(id.4029) | rs11184341 | G | C | TRUE | 0.066 | 0.093 | 0.014 | 0.167 | 4.07E-06 | 0.577 | 21.230 |
|  | rs111862613 | T | C | FALSE | 0.091 | -0.015 | 0.020 | 0.203 | 4.03E-06 | 0.943 | 21.252 |
|  | rs117107102 | A | G | FALSE | 0.205 | 0.031 | 0.043 | 0.349 | 2.11E-06 | 0.928 | 22.493 |
|  | rs11729256 | T | C | FALSE | 0.075 | 0.259 | 0.015 | 0.197 | 5.95E-07 | 0.188 | 24.928 |
|  | rs12908520 | G | A | FALSE | 0.062 | -0.022 | 0.013 | 0.150 | 2.28E-06 | 0.885 | 22.340 |
|  | rs2602429 | C | T | FALSE | 0.075 | 0.054 | 0.016 | 0.170 | 1.74E-06 | 0.753 | 22.863 |
|  | rs4242783 | G | A | FALSE | 0.069 | 0.033 | 0.015 | 0.167 | 3.06E-06 | 0.843 | 21.781 |
|  | rs4936098 | A | G | FALSE | 0.065 | -0.126 | 0.014 | 0.156 | 1.81E-06 | 0.420 | 22.786 |
|  | rs61779207 | G | A | FALSE | -0.076 | -0.065 | 0.017 | 0.180 | 6.18E-06 | 0.718 | 20.432 |
|  | rs74542928 | T | C | FALSE | 0.112 | -0.234 | 0.024 | 0.343 | 2.09E-06 | 0.496 | 22.508 |
|  | rs9349825 | A | G | FALSE | -0.070 | 0.172 | 0.015 | 0.189 | 1.71E-06 | 0.362 | 22.898 |
|  | rs941682 | G | A | FALSE | -0.063 | -0.047 | 0.014 | 0.167 | 1.12E-05 | 0.777 | 19.290 |
| Order Actinomycetales(id.420) | rs2889192 | G | T | FALSE | 0.088 | -0.120 | 0.019 | 0.209 | 5.80E-06 | 0.565 | 20.554 |
|  | rs34583783 | G | T | FALSE | 0.124 | 0.014 | 0.026 | 0.314 | 2.86E-06 | 0.964 | 21.908 |
|  | rs35011108 | A | G | FALSE | 0.242 | -0.092 | 0.050 | 0.302 | 1.63E-06 | 0.761 | 22.987 |
|  | rs4073240 | G | A | FALSE | 0.075 | -0.202 | 0.016 | 0.153 | 5.29E-06 | 0.187 | 20.729 |
|  | rs58484246 | T | C | FALSE | 0.076 | -0.012 | 0.017 | 0.156 | 7.23E-06 | 0.941 | 20.133 |
| Order Bacillales(id.1674) | rs2889192 | G | T | FALSE | 0.088 | -0.120 | 0.019 | 0.209 | 5.80E-06 | 0.565 | 20.554 |
|  | rs34583783 | G | T | FALSE | 0.124 | 0.014 | 0.026 | 0.314 | 2.86E-06 | 0.964 | 21.908 |
|  | rs35011108 | A | G | FALSE | 0.242 | -0.092 | 0.050 | 0.302 | 1.63E-06 | 0.761 | 22.987 |
|  | rs4073240 | G | A | FALSE | 0.075 | -0.202 | 0.016 | 0.153 | 5.29E-06 | 0.187 | 20.729 |
|  | rs58484246 | T | C | FALSE | 0.076 | -0.012 | 0.017 | 0.156 | 7.23E-06 | 0.941 | 20.133 |
| Order Bacteroidales(id.913) | rs11146701 | A | G | FALSE | 0.047 | -0.156 | 0.011 | 0.194 | 7.03E-06 | 0.421 | 20.186 |
|  | rs12938514 | A | G | FALSE | -0.200 | 0.540 | 0.042 | 0.876 | 1.53E-06 | 0.537 | 23.106 |
|  | rs13291434 | T | C | FALSE | 0.069 | 0.037 | 0.015 | 0.214 | 3.53E-06 | 0.862 | 21.507 |
|  | rs17343978 | A | C | FALSE | -0.055 | 0.028 | 0.012 | 0.184 | 4.44E-06 | 0.879 | 21.067 |
|  | rs2032750 | T | C | FALSE | -0.051 | -0.005 | 0.011 | 0.150 | 1.94E-06 | 0.974 | 22.657 |
|  | rs4916508 | G | A | FALSE | -0.047 | -0.067 | 0.011 | 0.150 | 9.34E-06 | 0.655 | 19.641 |
|  | rs55773148 | G | A | FALSE | -0.122 | -0.513 | 0.024 | 0.330 | 2.86E-07 | 0.120 | 26.341 |
|  | rs62531359 | T | G | FALSE | 0.066 | -0.009 | 0.015 | 0.196 | 1.22E-05 | 0.964 | 19.138 |
|  | rs62575403 | C | T | FALSE | 0.140 | -0.474 | 0.031 | 0.378 | 6.75E-06 | 0.210 | 20.264 |
|  | rs72706335 | T | C | FALSE | -0.222 | 0.354 | 0.049 | 0.476 | 6.57E-06 | 0.457 | 20.315 |
|  | rs73846128 | A | G | FALSE | -0.065 | 0.129 | 0.013 | 0.208 | 1.18E-06 | 0.535 | 23.602 |
|  | rs7546249 | T | A | TRUE | -0.057 | -0.247 | 0.012 | 0.163 | 1.66E-06 | 0.130 | 22.948 |
|  | rs79585701 | A | C | FALSE | 0.065 | 0.242 | 0.015 | 0.213 | 1.54E-05 | 0.257 | 18.687 |
|  | rs929878 | C | T | FALSE | -0.055 | 0.212 | 0.012 | 0.182 | 6.37E-06 | 0.243 | 20.372 |
| Order Burkholderiales(id.2874) | rs1511453 | A | G | FALSE | 0.091 | -0.344 | 0.020 | 0.321 | 4.95E-06 | 0.284 | 20.856 |
|  | rs1928341 | A | G | FALSE | 0.051 | 0.027 | 0.011 | 0.151 | 4.40E-06 | 0.859 | 21.084 |
|  | rs2321387 | G | A | FALSE | -0.051 | 0.274 | 0.011 | 0.150 | 3.48E-06 | 0.069 | 21.532 |
|  | rs2367850 | G | C | TRUE | 0.063 | -0.053 | 0.013 | 0.183 | 1.24E-06 | 0.772 | 23.516 |
|  | rs2613606 | C | T | FALSE | -0.050 | -0.032 | 0.011 | 0.150 | 4.83E-06 | 0.834 | 20.904 |
|  | rs4033856 | C | T | FALSE | 0.083 | -0.363 | 0.017 | 0.264 | 6.49E-07 | 0.169 | 24.762 |
|  | rs6087811 | T | G | FALSE | -0.102 | -0.313 | 0.020 | 0.249 | 3.23E-07 | 0.208 | 26.108 |
|  | rs62191117 | A | G | FALSE | 0.068 | 0.013 | 0.013 | 0.185 | 2.85E-07 | 0.944 | 26.349 |
|  | rs62395635 | T | C | FALSE | 0.110 | -0.088 | 0.024 | 0.315 | 3.32E-06 | 0.780 | 21.621 |
|  | rs72747231 | C | G | TRUE | -0.134 | -0.280 | 0.029 | 0.353 | 4.23E-06 | 0.426 | 21.157 |
|  | rs75242906 | C | T | FALSE | -0.121 | -0.383 | 0.028 | 0.270 | 1.70E-05 | 0.156 | 18.498 |
|  | rs7638039 | T | C | FALSE | 0.058 | -0.227 | 0.013 | 0.173 | 4.55E-06 | 0.189 | 21.017 |
| Order Clostridiales(id.1863) | rs10774377 | A | G | FALSE | 0.052 | -0.043 | 0.011 | 0.150 | 4.36E-06 | 0.774 | 21.099 |
|  | rs112334273 | G | A | FALSE | 0.064 | -0.106 | 0.013 | 0.166 | 5.21E-07 | 0.524 | 25.186 |
|  | rs13105690 | T | C | FALSE | -0.053 | 0.019 | 0.012 | 0.166 | 8.08E-06 | 0.908 | 19.919 |
|  | rs13179700 | T | C | FALSE | 0.051 | -0.151 | 0.011 | 0.157 | 3.11E-06 | 0.334 | 21.746 |
|  | rs1842454 | G | A | FALSE | -0.054 | 0.041 | 0.013 | 0.191 | 1.96E-05 | 0.830 | 18.228 |
|  | rs2273429 | A | G | FALSE | -0.073 | -0.054 | 0.015 | 0.243 | 2.10E-06 | 0.824 | 22.497 |
|  | rs290772 | G | A | FALSE | 0.084 | 0.249 | 0.020 | 0.330 | 1.77E-05 | 0.452 | 18.423 |
|  | rs3792064 | G | A | FALSE | 0.080 | -0.280 | 0.018 | 0.329 | 1.27E-05 | 0.395 | 19.053 |
|  | rs6126494 | G | T | FALSE | -0.097 | -0.294 | 0.021 | 0.311 | 4.87E-06 | 0.344 | 20.886 |
|  | rs6442336 | C | T | FALSE | -0.055 | 0.120 | 0.012 | 0.170 | 1.03E-05 | 0.483 | 19.458 |
|  | rs6814436 | T | C | FALSE | 0.074 | -0.265 | 0.015 | 0.213 | 8.70E-07 | 0.214 | 24.197 |
|  | rs6815608 | T | C | FALSE | 0.104 | 0.248 | 0.021 | 0.208 | 7.98E-07 | 0.233 | 24.363 |
|  | rs6934062 | G | C | TRUE | 0.052 | -0.097 | 0.012 | 0.159 | 6.83E-06 | 0.543 | 20.240 |
|  | rs72738886 | T | C | FALSE | 0.087 | 0.129 | 0.019 | 0.279 | 5.49E-06 | 0.643 | 20.657 |
|  | rs72915163 | T | C | FALSE | -0.058 | -1.204 | 0.012 | 3.983 | 1.49E-06 | 0.763 | 23.165 |
|  | rs72915163 | T | C | FALSE | -0.058 | -0.135 | 0.012 | 0.182 | 1.49E-06 | 0.459 | 23.165 |
|  | rs76860606 | C | G | TRUE | 0.094 | 0.175 | 0.023 | 0.267 | 2.90E-05 | 0.512 | 17.481 |
| Order Coriobacteriales(id.810) | rs11250875 | T | C | FALSE | 0.061 | 0.342 | 0.013 | 0.180 | 3.49E-06 | 0.057 | 21.526 |
|  | rs11656361 | A | C | FALSE | 0.077 | -0.071 | 0.018 | 0.194 | 1.06E-05 | 0.713 | 19.394 |
|  | rs12974142 | G | A | FALSE | 0.079 | 0.502 | 0.018 | 0.290 | 8.31E-06 | 0.083 | 19.865 |
|  | rs13307134 | C | T | FALSE | 0.057 | 0.016 | 0.013 | 0.199 | 7.46E-06 | 0.937 | 20.072 |
|  | rs1397793 | G | A | FALSE | -0.050 | -0.063 | 0.011 | 0.164 | 9.15E-06 | 0.701 | 19.681 |
|  | rs1816223 | A | G | FALSE | -0.059 | -0.186 | 0.013 | 0.186 | 5.51E-06 | 0.318 | 20.652 |
|  | rs240104 | T | C | FALSE | -0.060 | 0.054 | 0.013 | 0.166 | 1.96E-06 | 0.745 | 22.630 |
|  | rs2442778 | G | A | FALSE | -0.116 | -0.140 | 0.026 | 0.338 | 6.72E-06 | 0.678 | 20.272 |
|  | rs3025411 | A | G | FALSE | 0.093 | 0.020 | 0.021 | 0.245 | 9.72E-06 | 0.934 | 19.566 |
|  | rs34739816 | G | T | FALSE | 0.097 | -0.171 | 0.021 | 0.317 | 3.37E-06 | 0.590 | 21.594 |
|  | rs62448869 | T | A | TRUE | -0.049 | -0.081 | 0.011 | 0.150 | 8.20E-06 | 0.588 | 19.891 |
|  | rs67561917 | A | G | FALSE | -0.071 | 0.086 | 0.015 | 0.194 | 3.56E-06 | 0.659 | 21.486 |
|  | rs719099 | A | G | FALSE | 0.078 | 0.104 | 0.016 | 0.251 | 5.86E-07 | 0.680 | 24.957 |
|  | rs76779974 | C | G | TRUE | 0.077 | -0.398 | 0.017 | 0.254 | 6.53E-06 | 0.118 | 20.326 |
|  | rs80046645 | C | G | TRUE | 0.256 | -0.621 | 0.056 | 0.380 | 5.31E-06 | 0.102 | 20.724 |
|  | rs8010111 | G | A | FALSE | -0.103 | -0.082 | 0.023 | 0.268 | 6.52E-06 | 0.759 | 20.328 |
| Order Desulfovibrionales(id.3156) | rs112381107 | C | T | FALSE | 0.210 | -0.084 | 0.046 | 0.314 | 4.28E-06 | 0.790 | 21.136 |
|  | rs11599763 | T | C | FALSE | -0.055 | -0.032 | 0.012 | 0.153 | 2.33E-06 | 0.836 | 22.305 |
|  | rs16851319 | G | C | TRUE | -0.073 | -0.244 | 0.015 | 0.197 | 1.36E-06 | 0.217 | 23.331 |
|  | rs17791387 | A | G | FALSE | -0.073 | 0.104 | 0.015 | 0.253 | 2.44E-06 | 0.680 | 22.210 |
|  | rs186073 | T | C | FALSE | 0.053 | -0.230 | 0.012 | 0.152 | 8.03E-06 | 0.131 | 19.931 |
|  | rs2692012 | A | G | FALSE | 0.112 | -0.302 | 0.025 | 0.329 | 9.56E-06 | 0.359 | 19.597 |
|  | rs2838334 | G | A | FALSE | 0.057 | 0.038 | 0.012 | 0.156 | 4.64E-06 | 0.809 | 20.982 |
|  | rs3935584 | C | T | FALSE | -0.052 | -0.259 | 0.012 | 0.149 | 5.87E-06 | 0.083 | 20.531 |
|  | rs4506934 | C | T | FALSE | -0.095 | -0.189 | 0.020 | 0.232 | 2.20E-06 | 0.415 | 22.416 |
|  | rs55744759 | A | G | FALSE | -0.077 | -0.039 | 0.017 | 0.234 | 5.92E-06 | 0.867 | 20.514 |
|  | rs6058181 | C | T | FALSE | 0.084 | -0.012 | 0.017 | 0.202 | 4.71E-07 | 0.954 | 25.379 |
|  | rs62020470 | A | G | FALSE | -0.057 | -0.161 | 0.013 | 0.193 | 9.38E-06 | 0.405 | 19.633 |
|  | rs9928243 | C | A | FALSE | -0.054 | -0.102 | 0.012 | 0.149 | 3.77E-06 | 0.495 | 21.378 |
| Order Enterobacteriales(id.3468) | rs11026530 | T | C | FALSE | 0.082 | 0.220 | 0.019 | 0.211 | 1.02E-05 | 0.297 | 19.471 |
|  | rs111229068 | A | T | TRUE | 0.111 | 0.216 | 0.024 | 0.278 | 4.79E-06 | 0.439 | 20.921 |
|  | rs2374343 | C | G | TRUE | 0.058 | -0.234 | 0.013 | 0.151 | 3.80E-06 | 0.122 | 21.365 |
|  | rs35673018 | G | A | FALSE | 0.090 | 0.054 | 0.020 | 0.261 | 9.29E-06 | 0.837 | 19.653 |
|  | rs4792380 | A | T | TRUE | 0.116 | -0.632 | 0.026 | 0.436 | 7.26E-06 | 0.147 | 20.125 |
|  | rs504442 | T | G | FALSE | 0.084 | 0.113 | 0.019 | 0.244 | 8.93E-06 | 0.644 | 19.728 |
|  | rs6092361 | A | G | FALSE | 0.060 | 0.304 | 0.013 | 0.156 | 3.05E-06 | 0.052 | 21.786 |
|  | rs61973590 | C | G | TRUE | -0.061 | 0.081 | 0.013 | 0.157 | 5.95E-06 | 0.603 | 20.504 |
|  | rs78143293 | A | G | FALSE | -0.085 | 0.039 | 0.017 | 0.228 | 6.39E-07 | 0.864 | 24.792 |
|  | rs79757635 | C | A | FALSE | 0.076 | 0.090 | 0.017 | 0.221 | 9.47E-06 | 0.683 | 19.615 |
|  | rs80319214 | C | G | TRUE | 0.099 | -0.305 | 0.022 | 0.288 | 4.48E-06 | 0.289 | 21.048 |
| Order Erysipelotrichales(id.2148) | rs10781552 | C | T | FALSE | -0.055 | 0.080 | 0.012 | 0.165 | 1.96E-06 | 0.629 | 22.633 |
|  | rs17530232 | A | G | FALSE | 0.103 | 0.276 | 0.022 | 0.333 | 4.49E-06 | 0.407 | 21.042 |
|  | rs1884466 | C | T | FALSE | -0.048 | 0.294 | 0.011 | 0.149 | 8.78E-06 | 0.049 | 19.760 |
|  | rs2300774 | G | A | FALSE | 0.052 | -0.113 | 0.011 | 0.150 | 9.18E-07 | 0.448 | 24.094 |
|  | rs290833 | T | G | FALSE | -0.050 | -0.020 | 0.011 | 0.150 | 7.98E-06 | 0.894 | 19.942 |
|  | rs35161940 | T | C | FALSE | -0.081 | 0.079 | 0.017 | 0.241 | 1.52E-06 | 0.742 | 23.118 |
|  | rs4078432 | C | T | FALSE | -0.061 | -0.184 | 0.013 | 0.198 | 5.31E-06 | 0.354 | 20.723 |
|  | rs56970041 | T | G | FALSE | 0.072 | -0.096 | 0.016 | 0.308 | 1.07E-05 | 0.756 | 19.385 |
|  | rs62504403 | C | T | FALSE | 0.068 | -0.039 | 0.013 | 0.188 | 1.00E-07 | 0.837 | 28.371 |
|  | rs7234058 | T | C | FALSE | -0.095 | 0.383 | 0.019 | 0.260 | 1.10E-06 | 0.141 | 23.744 |
|  | rs7826267 | T | G | FALSE | -0.084 | -0.081 | 0.020 | 0.305 | 2.51E-05 | 0.791 | 17.755 |
|  | rs8003149 | C | T | FALSE | 0.054 | 0.021 | 0.012 | 0.158 | 4.04E-06 | 0.896 | 21.248 |
|  | rs921306 | T | C | FALSE | 0.049 | 0.144 | 0.011 | 0.153 | 6.02E-06 | 0.345 | 20.481 |
| Order Gastranaerophilales(id.1591) | rs11150282 | T | C | FALSE | 0.098 | 0.093 | 0.020 | 0.155 | 6.25E-07 | 0.549 | 24.834 |
|  | rs113884518 | T | C | FALSE | -0.206 | 0.068 | 0.046 | 0.471 | 6.13E-06 | 0.885 | 20.446 |
|  | rs1221147 | T | A | TRUE | 0.126 | 0.398 | 0.028 | 0.210 | 6.06E-06 | 0.058 | 20.471 |
|  | rs16851659 | G | C | TRUE | -0.090 | 0.055 | 0.019 | 0.156 | 1.60E-06 | 0.726 | 23.025 |
|  | rs28678345 | T | C | FALSE | 0.213 | -0.097 | 0.047 | 0.349 | 6.17E-06 | 0.780 | 20.434 |
|  | rs367480 | G | A | FALSE | -0.084 | 0.193 | 0.019 | 0.155 | 6.00E-06 | 0.214 | 20.487 |
|  | rs4129395 | G | A | FALSE | 0.090 | 0.261 | 0.019 | 0.149 | 1.05E-06 | 0.079 | 23.826 |
|  | rs73074665 | A | T | TRUE | 0.165 | -0.004 | 0.036 | 0.300 | 3.76E-06 | 0.990 | 21.381 |
|  | rs789069 | A | C | FALSE | -0.104 | 0.098 | 0.023 | 0.213 | 8.94E-06 | 0.645 | 19.725 |
|  | rs79790072 | T | C | FALSE | 0.226 | -0.139 | 0.049 | 0.429 | 3.60E-06 | 0.745 | 21.466 |
|  | rs8028558 | A | G | FALSE | 0.083 | 0.037 | 0.019 | 0.154 | 9.53E-06 | 0.811 | 19.603 |
|  | rs9864379 | T | C | FALSE | -0.161 | 0.513 | 0.029 | 0.210 | 4.18E-08 | 0.015 | 30.065 |
| Order Lactobacillales(id.1800) | rs11110282 | A | G | FALSE | -0.102 | 0.332 | 0.022 | 0.340 | 2.60E-06 | 0.329 | 22.090 |
|  | rs111552159 | C | G | TRUE | 0.129 | 0.411 | 0.029 | 0.362 | 7.34E-06 | 0.257 | 20.102 |
|  | rs11627423 | C | A | FALSE | 0.050 | -0.014 | 0.011 | 0.154 | 5.38E-06 | 0.927 | 20.696 |
|  | rs11730038 | G | A | FALSE | -0.061 | -0.069 | 0.013 | 0.165 | 2.72E-06 | 0.673 | 22.005 |
|  | rs12797734 | T | C | FALSE | 0.057 | 0.333 | 0.013 | 0.173 | 6.98E-06 | 0.054 | 20.198 |
|  | rs1595463 | C | A | FALSE | 0.048 | -0.171 | 0.011 | 0.150 | 1.04E-05 | 0.255 | 19.443 |
|  | rs1962325 | C | G | TRUE | 0.055 | 0.110 | 0.012 | 0.157 | 2.01E-06 | 0.484 | 22.587 |
|  | rs2370083 | G | T | FALSE | -0.081 | -0.048 | 0.018 | 0.306 | 8.59E-06 | 0.876 | 19.802 |
|  | rs2952251 | A | G | FALSE | -0.063 | -0.105 | 0.012 | 0.189 | 4.02E-07 | 0.578 | 25.683 |
|  | rs34989881 | A | G | FALSE | 0.113 | 0.001 | 0.025 | 0.346 | 4.13E-06 | 0.998 | 21.201 |
|  | rs35344081 | G | A | FALSE | 0.064 | -0.238 | 0.013 | 0.168 | 4.55E-07 | 0.156 | 25.446 |
|  | rs4028634 | T | C | FALSE | 0.053 | -0.177 | 0.011 | 0.154 | 1.30E-06 | 0.252 | 23.428 |
|  | rs57872228 | C | T | FALSE | -0.069 | -0.095 | 0.015 | 0.229 | 2.82E-06 | 0.678 | 21.933 |
|  | rs74352383 | T | A | TRUE | -0.103 | -0.584 | 0.022 | 0.270 | 2.01E-06 | 0.031 | 22.583 |
|  | rs74663707 | C | T | FALSE | 0.098 | -0.111 | 0.022 | 0.303 | 1.22E-05 | 0.715 | 19.137 |
|  | rs76717940 | T | A | TRUE | 0.161 | -0.095 | 0.033 | 0.328 | 8.58E-07 | 0.772 | 24.224 |
|  | rs77558518 | A | G | FALSE | -0.106 | 0.023 | 0.022 | 0.254 | 1.90E-06 | 0.929 | 22.695 |
|  | rs78938557 | T | C | FALSE | 0.106 | 0.077 | 0.023 | 0.439 | 6.24E-06 | 0.861 | 20.412 |
|  | rs9581006 | C | T | FALSE | 0.226 | 0.294 | 0.047 | 0.386 | 1.49E-06 | 0.447 | 23.163 |
| Order Methanobacteriales(id.120) | rs10202904 | T | G | FALSE | -0.122 | 0.015 | 0.024 | 0.151 | 2.30E-07 | 0.923 | 26.761 |
|  | rs10424197 | G | A | FALSE | -0.111 | -0.129 | 0.025 | 0.171 | 6.94E-06 | 0.451 | 20.211 |
|  | rs11018665 | A | T | TRUE | 0.111 | -0.159 | 0.025 | 0.173 | 8.61E-06 | 0.357 | 19.797 |
|  | rs12825290 | C | G | TRUE | -0.217 | -0.013 | 0.049 | 0.317 | 1.12E-05 | 0.967 | 19.296 |
|  | rs4257531 | G | A | FALSE | 0.164 | -0.412 | 0.036 | 0.244 | 6.57E-06 | 0.091 | 20.316 |
|  | rs56131665 | G | A | FALSE | 0.179 | -0.057 | 0.039 | 0.243 | 5.46E-06 | 0.813 | 20.669 |
|  | rs62241835 | G | T | FALSE | -0.203 | -0.757 | 0.042 | 0.519 | 1.14E-06 | 0.145 | 23.683 |
|  | rs6508769 | T | C | FALSE | 0.154 | 0.058 | 0.034 | 0.209 | 8.35E-06 | 0.783 | 19.856 |
|  | rs73068003 | G | T | FALSE | -0.158 | 0.133 | 0.035 | 0.250 | 6.95E-06 | 0.594 | 20.206 |
|  | rs73457410 | A | G | FALSE | 0.215 | 0.466 | 0.044 | 0.301 | 8.18E-07 | 0.122 | 24.316 |
|  | rs75208022 | C | T | FALSE | -0.227 | 0.103 | 0.049 | 0.253 | 3.16E-06 | 0.684 | 21.717 |
|  | rs894996 | C | A | FALSE | 0.217 | -0.519 | 0.045 | 0.286 | 1.35E-06 | 0.069 | 23.349 |
| Order MollicutesRF9(id.11579) | rs10071529 | G | C | TRUE | 0.125 | 0.034 | 0.028 | 0.241 | 7.15E-06 | 0.887 | 20.154 |
|  | rs114184530 | T | C | FALSE | -0.155 | 0.181 | 0.034 | 0.267 | 5.48E-06 | 0.496 | 20.662 |
|  | rs11779863 | G | A | FALSE | -0.077 | 0.043 | 0.017 | 0.206 | 7.18E-06 | 0.833 | 20.146 |
|  | rs12566890 | T | G | FALSE | -0.103 | 0.341 | 0.024 | 0.223 | 1.99E-05 | 0.126 | 18.196 |
|  | rs13100746 | C | T | FALSE | 0.064 | 0.144 | 0.014 | 0.149 | 7.53E-06 | 0.335 | 20.055 |
|  | rs17235252 | T | C | FALSE | -0.122 | -0.120 | 0.026 | 0.231 | 1.79E-06 | 0.603 | 22.808 |
|  | rs3932485 | C | T | FALSE | 0.063 | 0.161 | 0.014 | 0.152 | 9.79E-06 | 0.288 | 19.551 |
|  | rs515984 | T | C | FALSE | -0.088 | 0.101 | 0.019 | 0.232 | 4.41E-06 | 0.663 | 21.078 |
|  | rs62188991 | G | C | TRUE | -0.111 | 0.297 | 0.024 | 0.320 | 4.36E-06 | 0.353 | 21.099 |
|  | rs638542 | G | A | FALSE | -0.071 | 0.024 | 0.016 | 0.164 | 6.96E-06 | 0.886 | 20.204 |
|  | rs739151 | C | G | TRUE | 0.065 | -0.016 | 0.014 | 0.149 | 3.02E-06 | 0.913 | 21.804 |
|  | rs74603314 | T | C | FALSE | 0.231 | 0.190 | 0.049 | 0.380 | 2.45E-06 | 0.616 | 22.207 |
|  | rs76373661 | G | A | FALSE | 0.091 | -0.076 | 0.020 | 0.204 | 7.67E-06 | 0.710 | 20.019 |
|  | rs7706512 | G | A | FALSE | 0.066 | 0.072 | 0.014 | 0.149 | 2.25E-06 | 0.626 | 22.371 |
|  | rs7801843 | A | G | FALSE | -0.087 | 0.072 | 0.019 | 0.208 | 7.81E-06 | 0.731 | 19.983 |
|  | rs949341 | G | A | FALSE | 0.066 | 0.087 | 0.015 | 0.166 | 8.19E-06 | 0.600 | 19.893 |
| Order NB1n(id.3953) | rs11251024 | G | A | FALSE | 0.104 | -0.317 | 0.021 | 0.168 | 4.65E-07 | 0.059 | 25.404 |
|  | rs11606187 | A | G | FALSE | -0.155 | -0.249 | 0.033 | 0.215 | 2.18E-06 | 0.248 | 22.432 |
|  | rs13219468 | G | C | TRUE | 0.115 | 0.221 | 0.024 | 0.165 | 1.13E-06 | 0.180 | 23.694 |
|  | rs13385922 | T | C | FALSE | 0.093 | 0.090 | 0.020 | 0.156 | 3.91E-06 | 0.564 | 21.311 |
|  | rs166849 | G | A | FALSE | 0.091 | -0.060 | 0.020 | 0.151 | 6.64E-06 | 0.693 | 20.293 |
|  | rs2172426 | C | T | FALSE | -0.102 | 0.140 | 0.020 | 0.152 | 2.85E-07 | 0.357 | 26.348 |
|  | rs267959 | A | G | FALSE | 0.099 | -0.035 | 0.021 | 0.166 | 2.42E-06 | 0.834 | 22.231 |
|  | rs2930903 | C | G | TRUE | 0.091 | 0.063 | 0.021 | 0.155 | 1.44E-05 | 0.684 | 18.810 |
|  | rs4383094 | T | C | FALSE | 0.149 | -0.026 | 0.032 | 0.215 | 3.28E-06 | 0.905 | 21.646 |
|  | rs55921101 | A | T | TRUE | -0.109 | 0.113 | 0.024 | 0.176 | 3.74E-06 | 0.519 | 21.392 |
|  | rs6051857 | A | G | FALSE | -0.134 | 0.017 | 0.029 | 0.193 | 4.55E-06 | 0.929 | 21.016 |
|  | rs60583455 | T | C | FALSE | 0.109 | 0.449 | 0.021 | 0.156 | 2.69E-07 | 0.004 | 26.460 |
|  | rs60775321 | T | C | FALSE | -0.096 | 0.041 | 0.021 | 0.165 | 6.77E-06 | 0.804 | 20.256 |
|  | rs72671304 | T | C | FALSE | 0.172 | 0.268 | 0.037 | 0.283 | 3.21E-06 | 0.344 | 21.688 |
|  | rs7911787 | G | T | FALSE | -0.223 | 0.035 | 0.047 | 0.422 | 2.10E-06 | 0.935 | 22.504 |
|  | rs9542068 | T | C | FALSE | 0.099 | 0.073 | 0.022 | 0.158 | 5.59E-06 | 0.642 | 20.625 |
| Order Pasteurellales(id.3688) | rs10840326 | C | G | TRUE | -0.072 | 0.092 | 0.015 | 0.155 | 1.52E-06 | 0.552 | 23.126 |
|  | rs10965428 | C | A | FALSE | -0.120 | -0.126 | 0.026 | 0.323 | 3.43E-06 | 0.697 | 21.561 |
|  | rs111582866 | G | A | FALSE | -0.114 | -0.056 | 0.026 | 0.265 | 8.81E-06 | 0.832 | 19.753 |
|  | rs12050685 | A | G | FALSE | -0.067 | 0.088 | 0.015 | 0.164 | 1.07E-05 | 0.594 | 19.385 |
|  | rs12191680 | C | G | TRUE | 0.102 | 0.096 | 0.020 | 0.245 | 2.18E-07 | 0.695 | 26.869 |
|  | rs16970009 | A | G | FALSE | 0.187 | -0.752 | 0.043 | 0.524 | 1.29E-05 | 0.151 | 19.027 |
|  | rs35510 | A | G | FALSE | 0.123 | 0.142 | 0.026 | 0.366 | 3.62E-06 | 0.698 | 21.456 |
|  | rs4822728 | T | C | FALSE | 0.069 | -0.224 | 0.015 | 0.149 | 4.23E-06 | 0.132 | 21.156 |
|  | rs6092684 | A | T | TRUE | 0.068 | -0.137 | 0.015 | 0.150 | 3.08E-06 | 0.361 | 21.769 |
|  | rs62568866 | T | A | TRUE | -0.118 | 0.071 | 0.026 | 0.243 | 5.29E-06 | 0.770 | 20.730 |
|  | rs6972479 | A | G | FALSE | -0.078 | 0.063 | 0.018 | 0.184 | 8.25E-06 | 0.732 | 19.878 |
|  | rs72756943 | G | A | FALSE | 0.140 | 0.629 | 0.030 | 0.298 | 3.91E-06 | 0.035 | 21.308 |
|  | rs73139353 | A | C | FALSE | -0.223 | 0.491 | 0.048 | 0.265 | 4.38E-06 | 0.064 | 21.092 |
|  | rs731534 | C | G | TRUE | -0.099 | -0.082 | 0.022 | 0.236 | 5.10E-06 | 0.729 | 20.798 |
|  | rs76022354 | C | T | FALSE | 0.243 | -0.247 | 0.050 | 0.341 | 1.21E-06 | 0.469 | 23.560 |
|  | rs78909003 | T | C | FALSE | -0.241 | -0.208 | 0.050 | 0.328 | 1.31E-06 | 0.525 | 23.415 |
|  | rs9382510 | C | T | FALSE | -0.088 | -0.122 | 0.017 | 0.169 | 2.12E-07 | 0.468 | 26.921 |
|  | rs9895850 | T | C | FALSE | -0.176 | 0.143 | 0.041 | 0.365 | 1.70E-05 | 0.695 | 18.497 |
|  | rs9938097 | T | C | FALSE | -0.071 | 0.044 | 0.016 | 0.153 | 6.94E-06 | 0.775 | 20.209 |
| Order Rhodospirillales(id.2667) | rs1035406 | G | A | FALSE | -0.115 | 0.137 | 0.025 | 0.231 | 3.73E-06 | 0.553 | 21.401 |
|  | rs11591293 | G | T | FALSE | 0.072 | -0.032 | 0.016 | 0.150 | 4.90E-06 | 0.831 | 20.876 |
|  | rs11630875 | T | C | FALSE | 0.095 | -0.200 | 0.020 | 0.223 | 2.93E-06 | 0.369 | 21.865 |
|  | rs13336560 | C | T | FALSE | -0.070 | -0.152 | 0.016 | 0.153 | 9.56E-06 | 0.319 | 19.598 |
|  | rs2162883 | C | T | FALSE | 0.090 | 0.270 | 0.019 | 0.197 | 3.10E-06 | 0.171 | 21.751 |
|  | rs3730086 | A | G | FALSE | 0.080 | 0.218 | 0.018 | 0.171 | 7.54E-06 | 0.201 | 20.051 |
|  | rs3754624 | C | T | FALSE | 0.094 | 0.031 | 0.020 | 0.195 | 2.09E-06 | 0.875 | 22.511 |
|  | rs4278423 | T | C | FALSE | 0.105 | 0.018 | 0.023 | 0.305 | 6.77E-06 | 0.953 | 20.256 |
|  | rs4822789 | G | C | TRUE | 0.073 | -0.041 | 0.016 | 0.153 | 5.06E-06 | 0.788 | 20.813 |
|  | rs55876211 | C | T | FALSE | -0.087 | 0.002 | 0.020 | 0.171 | 9.36E-06 | 0.989 | 19.638 |
|  | rs61933850 | G | A | FALSE | 0.165 | -0.232 | 0.036 | 0.222 | 4.87E-06 | 0.297 | 20.888 |
|  | rs7001029 | C | T | FALSE | 0.121 | -0.197 | 0.026 | 0.259 | 3.73E-06 | 0.446 | 21.397 |
|  | rs76784716 | A | G | FALSE | 0.136 | 0.104 | 0.028 | 0.237 | 1.72E-06 | 0.661 | 22.881 |
|  | rs77304857 | C | A | FALSE | -0.100 | 0.188 | 0.022 | 0.189 | 7.14E-06 | 0.319 | 20.156 |
|  | rs9813022 | A | G | FALSE | -0.083 | -0.169 | 0.016 | 0.154 | 3.25E-07 | 0.273 | 26.093 |
| Order Selenomonadales(id.2165) | rs10787507 | G | C | TRUE | -0.055 | 0.024 | 0.013 | 0.190 | 1.78E-05 | 0.900 | 18.411 |
|  | rs1135612 | G | A | FALSE | 0.053 | -0.111 | 0.012 | 0.182 | 8.77E-06 | 0.542 | 19.761 |
|  | rs13086907 | G | A | FALSE | 0.063 | 0.205 | 0.013 | 0.180 | 2.07E-06 | 0.256 | 22.532 |
|  | rs1447205 | G | C | TRUE | -0.050 | -0.027 | 0.011 | 0.155 | 3.62E-06 | 0.860 | 21.458 |
|  | rs1643968 | T | C | FALSE | -0.057 | 0.029 | 0.011 | 0.155 | 4.81E-07 | 0.852 | 25.339 |
|  | rs1649999 | A | G | FALSE | 0.075 | -0.347 | 0.017 | 0.252 | 6.81E-06 | 0.169 | 20.246 |
|  | rs2834062 | A | G | FALSE | 0.049 | -0.140 | 0.011 | 0.163 | 7.01E-06 | 0.392 | 20.190 |
|  | rs4722181 | T | G | FALSE | 0.050 | -0.085 | 0.011 | 0.149 | 2.15E-06 | 0.568 | 22.452 |
|  | rs60274479 | T | C | FALSE | -0.066 | -0.195 | 0.013 | 0.189 | 8.76E-07 | 0.303 | 24.182 |
|  | rs61249479 | A | C | FALSE | 0.078 | -0.290 | 0.017 | 0.206 | 4.06E-06 | 0.160 | 21.236 |
|  | rs71405394 | G | A | FALSE | -0.114 | -0.307 | 0.024 | 0.295 | 2.06E-06 | 0.298 | 22.539 |
|  | rs73232831 | G | A | FALSE | -0.152 | 0.657 | 0.031 | 0.398 | 1.43E-06 | 0.098 | 23.242 |
|  | rs9423647 | G | A | FALSE | 0.048 | 0.175 | 0.011 | 0.150 | 5.58E-06 | 0.242 | 20.628 |
| Order Verrucomicrobiales(id.4030) | rs11184341 | G | C | TRUE | 0.066 | 0.093 | 0.014 | 0.167 | 4.07E-06 | 0.577 | 21.230 |
|  | rs111862613 | T | C | FALSE | 0.091 | -0.015 | 0.020 | 0.203 | 4.03E-06 | 0.943 | 21.252 |
|  | rs117107102 | A | G | FALSE | 0.205 | 0.031 | 0.043 | 0.349 | 2.11E-06 | 0.928 | 22.493 |
|  | rs11729256 | T | C | FALSE | 0.075 | 0.259 | 0.015 | 0.197 | 5.95E-07 | 0.188 | 24.928 |
|  | rs12908520 | G | A | FALSE | 0.062 | -0.022 | 0.013 | 0.150 | 2.28E-06 | 0.885 | 22.340 |
|  | rs2602429 | C | T | FALSE | 0.075 | 0.054 | 0.016 | 0.170 | 1.74E-06 | 0.753 | 22.863 |
|  | rs4242783 | G | A | FALSE | 0.069 | 0.033 | 0.015 | 0.167 | 3.06E-06 | 0.843 | 21.781 |
|  | rs4936098 | A | G | FALSE | 0.065 | -0.126 | 0.014 | 0.156 | 1.81E-06 | 0.420 | 22.786 |
|  | rs61779207 | G | A | FALSE | -0.076 | -0.065 | 0.017 | 0.180 | 6.18E-06 | 0.718 | 20.432 |
|  | rs74542928 | T | C | FALSE | 0.112 | -0.234 | 0.024 | 0.343 | 2.09E-06 | 0.496 | 22.508 |
|  | rs9349825 | A | G | FALSE | -0.070 | 0.172 | 0.015 | 0.189 | 1.71E-06 | 0.362 | 22.898 |
|  | rs941682 | G | A | FALSE | -0.063 | -0.047 | 0.014 | 0.167 | 1.12E-05 | 0.777 | 19.290 |
| Order Victivallales(id.2254) | rs1002941 | G | A | FALSE | 0.105 | 0.042 | 0.023 | 0.173 | 6.85E-06 | 0.808 | 20.233 |
|  | rs11770843 | C | T | FALSE | 0.109 | -0.123 | 0.023 | 0.160 | 3.18E-06 | 0.441 | 21.707 |
|  | rs17114848 | G | A | FALSE | 0.152 | -0.239 | 0.032 | 0.250 | 2.62E-06 | 0.338 | 22.073 |
|  | rs2031282 | A | G | FALSE | 0.122 | 0.263 | 0.027 | 0.195 | 5.99E-06 | 0.179 | 20.491 |
|  | rs2731834 | C | G | TRUE | 0.109 | 0.246 | 0.024 | 0.174 | 3.86E-06 | 0.157 | 21.335 |
|  | rs2825714 | A | G | FALSE | -0.137 | -0.133 | 0.029 | 0.199 | 2.03E-06 | 0.504 | 22.569 |
|  | rs62570196 | C | T | FALSE | -0.216 | -0.062 | 0.044 | 0.369 | 8.72E-07 | 0.867 | 24.192 |
|  | rs72640280 | A | G | FALSE | 0.220 | 0.498 | 0.049 | 0.323 | 5.92E-06 | 0.123 | 20.513 |
|  | rs73113483 | T | A | TRUE | -0.131 | 0.233 | 0.029 | 0.229 | 5.50E-06 | 0.308 | 20.656 |
|  | rs77599476 | A | G | FALSE | 0.230 | 0.068 | 0.048 | 0.322 | 1.62E-06 | 0.833 | 23.002 |
| Phylum Bacteroidetes(id.905) | rs12938514 | A | G | FALSE | -0.201 | 0.540 | 0.042 | 0.876 | 1.34E-06 | 0.537 | 23.367 |
|  | rs13291434 | T | C | FALSE | 0.071 | 0.037 | 0.015 | 0.214 | 2.45E-06 | 0.862 | 22.206 |
|  | rs17343978 | A | C | FALSE | -0.056 | 0.028 | 0.012 | 0.184 | 3.78E-06 | 0.879 | 21.374 |
|  | rs2032750 | T | C | FALSE | -0.051 | -0.005 | 0.011 | 0.150 | 1.73E-06 | 0.974 | 22.878 |
|  | rs62531359 | T | G | FALSE | 0.066 | -0.009 | 0.015 | 0.196 | 1.14E-05 | 0.964 | 19.260 |
|  | rs62575403 | C | T | FALSE | 0.145 | -0.474 | 0.031 | 0.378 | 2.94E-06 | 0.210 | 21.854 |
|  | rs6586324 | T | C | FALSE | 0.048 | 0.143 | 0.011 | 0.150 | 5.86E-06 | 0.342 | 20.535 |
|  | rs72706335 | T | C | FALSE | -0.223 | 0.354 | 0.049 | 0.476 | 6.08E-06 | 0.457 | 20.464 |
|  | rs73512608 | G | A | FALSE | -0.123 | -0.513 | 0.024 | 0.330 | 1.99E-07 | 0.120 | 27.045 |
|  | rs73846128 | A | G | FALSE | -0.066 | 0.129 | 0.013 | 0.208 | 6.48E-07 | 0.535 | 24.765 |
|  | rs7546249 | T | A | TRUE | -0.057 | -0.247 | 0.012 | 0.163 | 1.53E-06 | 0.130 | 23.116 |
|  | rs7999780 | G | A | FALSE | 0.054 | 0.167 | 0.012 | 0.169 | 1.18E-05 | 0.324 | 19.200 |
|  | rs929878 | C | T | FALSE | -0.054 | 0.212 | 0.012 | 0.182 | 8.85E-06 | 0.243 | 19.745 |
| Phylum Cyanobacteria(id.1500) | rs12555298 | G | A | FALSE | 0.097 | -0.273 | 0.022 | 0.199 | 8.92E-06 | 0.170 | 19.730 |
|  | rs2314810 | C | G | TRUE | -0.218 | 0.581 | 0.046 | 0.354 | 2.63E-06 | 0.101 | 22.071 |
|  | rs2553290 | A | T | TRUE | 0.096 | 0.172 | 0.020 | 0.209 | 2.05E-06 | 0.411 | 22.549 |
|  | rs2585223 | T | C | FALSE | 0.111 | -0.045 | 0.025 | 0.226 | 6.95E-06 | 0.842 | 20.206 |
|  | rs584122 | C | T | FALSE | -0.152 | -0.160 | 0.033 | 0.320 | 3.42E-06 | 0.616 | 21.565 |
|  | rs61972390 | T | C | FALSE | 0.107 | -0.219 | 0.024 | 0.219 | 9.55E-06 | 0.317 | 19.600 |
|  | rs7148504 | G | T | FALSE | 0.080 | 0.210 | 0.018 | 0.151 | 5.85E-06 | 0.166 | 20.537 |
|  | rs76531781 | T | C | FALSE | -0.232 | -0.036 | 0.049 | 0.377 | 2.61E-06 | 0.924 | 22.085 |
|  | rs789068 | G | A | FALSE | -0.111 | 0.098 | 0.021 | 0.213 | 1.68E-07 | 0.645 | 27.366 |
|  | rs9864379 | T | C | FALSE | -0.139 | 0.513 | 0.027 | 0.210 | 2.18E-07 | 0.015 | 26.866 |
| Phylum Euryarchaeota(id.55) | rs10202904 | T | G | FALSE | -0.116 | 0.015 | 0.023 | 0.151 | 4.73E-07 | 0.923 | 25.371 |
|  | rs11022995 | G | A | FALSE | -0.104 | 0.025 | 0.023 | 0.149 | 5.85E-06 | 0.868 | 20.538 |
|  | rs34928225 | T | C | FALSE | 0.200 | -0.063 | 0.043 | 0.256 | 2.66E-06 | 0.806 | 22.051 |
|  | rs45498998 | G | A | FALSE | -0.132 | -0.259 | 0.029 | 0.205 | 6.42E-06 | 0.206 | 20.359 |
|  | rs56131665 | G | A | FALSE | 0.177 | -0.057 | 0.039 | 0.243 | 4.61E-06 | 0.813 | 20.993 |
|  | rs6064552 | T | C | FALSE | -0.124 | 0.059 | 0.028 | 0.192 | 7.37E-06 | 0.759 | 20.095 |
|  | rs6508769 | T | C | FALSE | 0.151 | 0.058 | 0.034 | 0.209 | 7.99E-06 | 0.783 | 19.941 |
|  | rs7015093 | G | A | FALSE | -0.118 | -0.108 | 0.026 | 0.170 | 7.92E-06 | 0.526 | 19.957 |
|  | rs73031978 | C | G | TRUE | -0.198 | -0.757 | 0.041 | 0.519 | 1.89E-06 | 0.145 | 22.709 |
|  | rs76029318 | T | C | FALSE | 0.215 | 0.483 | 0.044 | 0.303 | 9.55E-07 | 0.111 | 24.017 |
|  | rs7635189 | G | A | FALSE | 0.120 | -0.150 | 0.026 | 0.167 | 3.72E-06 | 0.371 | 21.402 |
|  | rs77658038 | A | C | FALSE | -0.160 | 0.186 | 0.034 | 0.186 | 2.62E-06 | 0.317 | 22.077 |
|  | rs894996 | C | A | FALSE | 0.204 | -0.519 | 0.044 | 0.286 | 3.72E-06 | 0.069 | 21.404 |
| Phylum Firmicutes(id.1672) | rs112334273 | G | A | FALSE | 0.063 | -0.106 | 0.013 | 0.166 | 8.46E-07 | 0.524 | 24.251 |
|  | rs12554342 | G | C | TRUE | -0.053 | 0.263 | 0.012 | 0.183 | 6.90E-06 | 0.149 | 20.221 |
|  | rs2273429 | A | G | FALSE | -0.070 | -0.054 | 0.015 | 0.243 | 4.49E-06 | 0.824 | 21.041 |
|  | rs2332027 | G | A | FALSE | -0.048 | -0.046 | 0.010 | 0.154 | 4.13E-06 | 0.767 | 21.201 |
|  | rs2547978 | A | G | FALSE | 0.047 | 0.049 | 0.011 | 0.153 | 9.29E-06 | 0.752 | 19.652 |
|  | rs3792064 | G | A | FALSE | 0.090 | -0.280 | 0.018 | 0.329 | 8.83E-07 | 0.395 | 24.168 |
|  | rs3852931 | C | T | FALSE | -0.048 | -0.096 | 0.010 | 0.157 | 4.40E-06 | 0.539 | 21.080 |
|  | rs4750583 | A | G | FALSE | 0.062 | 0.138 | 0.014 | 0.191 | 7.97E-06 | 0.470 | 19.944 |
|  | rs56199908 | T | C | FALSE | -0.186 | 0.730 | 0.041 | 0.331 | 5.74E-06 | 0.027 | 20.572 |
|  | rs6814436 | T | C | FALSE | 0.068 | -0.265 | 0.015 | 0.213 | 6.50E-06 | 0.214 | 20.334 |
|  | rs6815608 | T | C | FALSE | 0.094 | 0.248 | 0.021 | 0.208 | 9.09E-06 | 0.233 | 19.693 |
|  | rs7247191 | T | C | FALSE | -0.071 | 0.074 | 0.016 | 0.249 | 5.92E-06 | 0.768 | 20.515 |
|  | rs72738886 | T | C | FALSE | 0.086 | 0.129 | 0.019 | 0.279 | 5.60E-06 | 0.643 | 20.621 |
|  | rs72771021 | C | T | FALSE | -0.141 | 0.194 | 0.031 | 0.297 | 4.54E-06 | 0.514 | 21.022 |
|  | rs7557870 | T | A | TRUE | 0.099 | 0.190 | 0.019 | 0.312 | 1.36E-07 | 0.542 | 27.775 |
|  | rs7573799 | C | G | TRUE | -0.070 | 0.134 | 0.015 | 0.207 | 5.70E-06 | 0.518 | 20.587 |
|  | rs7975768 | C | G | TRUE | 0.051 | -0.163 | 0.011 | 0.150 | 2.68E-06 | 0.276 | 22.036 |
|  | rs8085381 | G | A | FALSE | 0.065 | 0.048 | 0.015 | 0.192 | 9.56E-06 | 0.805 | 19.597 |
| Phylum Lentisphaerae(id.2238) | rs1002941 | G | A | FALSE | 0.108 | 0.042 | 0.023 | 0.173 | 3.97E-06 | 0.808 | 21.280 |
|  | rs11770843 | C | T | FALSE | 0.112 | -0.123 | 0.023 | 0.160 | 1.88E-06 | 0.441 | 22.715 |
|  | rs17114848 | G | A | FALSE | 0.149 | -0.239 | 0.032 | 0.250 | 4.31E-06 | 0.338 | 21.120 |
|  | rs2031282 | A | G | FALSE | 0.120 | 0.263 | 0.027 | 0.195 | 8.38E-06 | 0.179 | 19.850 |
|  | rs2731834 | C | G | TRUE | 0.110 | 0.246 | 0.024 | 0.174 | 3.46E-06 | 0.157 | 21.544 |
|  | rs2825714 | A | G | FALSE | -0.138 | -0.133 | 0.029 | 0.199 | 1.74E-06 | 0.504 | 22.861 |
|  | rs60995569 | T | G | FALSE | -0.161 | -0.215 | 0.034 | 0.239 | 1.85E-06 | 0.368 | 22.743 |
|  | rs62570196 | C | T | FALSE | -0.217 | -0.062 | 0.044 | 0.369 | 7.78E-07 | 0.867 | 24.410 |
|  | rs72640280 | A | G | FALSE | 0.220 | 0.498 | 0.049 | 0.323 | 5.87E-06 | 0.123 | 20.530 |
|  | rs73113483 | T | A | TRUE | -0.132 | 0.233 | 0.029 | 0.229 | 5.01E-06 | 0.308 | 20.833 |
|  | rs77599476 | A | G | FALSE | 0.230 | 0.068 | 0.048 | 0.322 | 1.65E-06 | 0.833 | 22.964 |
| Phylum Proteobacteria(id.2375) | rs10750258 | A | C | FALSE | -0.049 | 0.229 | 0.011 | 0.154 | 5.67E-06 | 0.137 | 20.598 |
|  | rs11126162 | T | C | FALSE | -0.077 | -0.050 | 0.019 | 0.270 | 3.80E-05 | 0.854 | 16.971 |
|  | rs11715072 | G | A | FALSE | -0.052 | 0.221 | 0.012 | 0.162 | 6.61E-06 | 0.174 | 20.303 |
|  | rs12150865 | C | T | FALSE | 0.051 | 0.065 | 0.011 | 0.148 | 1.52E-06 | 0.663 | 23.118 |
|  | rs12467198 | C | T | FALSE | 0.050 | 0.009 | 0.011 | 0.150 | 7.69E-06 | 0.951 | 20.013 |
|  | rs2347697 | G | T | FALSE | 0.050 | 0.047 | 0.011 | 0.160 | 4.04E-06 | 0.769 | 21.247 |
|  | rs2532663 | G | A | FALSE | -0.126 | 0.340 | 0.026 | 0.249 | 1.13E-06 | 0.171 | 23.685 |
|  | rs312757 | C | G | TRUE | -0.052 | 0.155 | 0.011 | 0.151 | 4.57E-06 | 0.304 | 21.009 |
|  | rs3890996 | T | G | FALSE | -0.047 | 0.052 | 0.011 | 0.149 | 7.04E-06 | 0.730 | 20.183 |
|  | rs4340090 | C | T | FALSE | -0.067 | -0.186 | 0.015 | 0.228 | 1.32E-05 | 0.415 | 18.975 |
|  | rs6707783 | C | T | FALSE | 0.085 | -0.019 | 0.019 | 0.259 | 6.06E-06 | 0.941 | 20.470 |
|  | rs72771021 | C | T | FALSE | 0.142 | 0.194 | 0.031 | 0.297 | 4.58E-06 | 0.514 | 21.004 |
|  | rs74757828 | A | T | TRUE | 0.095 | -0.526 | 0.022 | 0.283 | 1.16E-05 | 0.063 | 19.232 |
|  | rs922773 | C | T | FALSE | -0.080 | 0.142 | 0.016 | 0.245 | 3.54E-07 | 0.562 | 25.928 |
| Phylum Tenericutes(id.3919) | rs10108398 | G | A | FALSE | 0.077 | 0.138 | 0.015 | 0.166 | 5.85E-07 | 0.406 | 24.960 |
|  | rs12566890 | T | G | FALSE | -0.101 | 0.341 | 0.023 | 0.223 | 1.19E-05 | 0.126 | 19.176 |
|  | rs17214486 | C | A | FALSE | 0.061 | -0.128 | 0.014 | 0.159 | 6.89E-06 | 0.420 | 20.223 |
|  | rs2464826 | A | C | FALSE | 0.094 | -0.067 | 0.021 | 0.237 | 8.27E-06 | 0.778 | 19.874 |
|  | rs28537087 | G | A | FALSE | 0.082 | -0.050 | 0.019 | 0.173 | 1.31E-05 | 0.775 | 19.002 |
|  | rs3768491 | A | G | FALSE | -0.068 | 0.020 | 0.015 | 0.164 | 4.90E-06 | 0.902 | 20.875 |
|  | rs4885016 | T | C | FALSE | -0.082 | 0.210 | 0.018 | 0.221 | 6.41E-06 | 0.343 | 20.363 |
|  | rs6043847 | T | C | FALSE | -0.115 | 0.138 | 0.025 | 0.318 | 3.78E-06 | 0.664 | 21.375 |
|  | rs67374027 | A | T | TRUE | 0.075 | 0.193 | 0.015 | 0.174 | 1.17E-06 | 0.268 | 23.634 |
|  | rs72901605 | T | C | FALSE | -0.084 | -0.011 | 0.018 | 0.234 | 2.29E-06 | 0.964 | 22.338 |
|  | rs74603314 | T | C | FALSE | 0.222 | 0.190 | 0.046 | 0.380 | 1.69E-06 | 0.616 | 22.924 |
|  | rs78169027 | A | G | FALSE | -0.108 | -0.344 | 0.024 | 0.314 | 5.03E-06 | 0.274 | 20.824 |
| Phylum Verrucomicrobia(id.3982) | rs11252894 | A | C | FALSE | 0.078 | 0.061 | 0.016 | 0.176 | 1.46E-06 | 0.728 | 23.203 |
|  | rs117107102 | A | G | FALSE | 0.204 | 0.031 | 0.043 | 0.349 | 1.77E-06 | 0.928 | 22.828 |
|  | rs11729256 | T | C | FALSE | 0.070 | 0.259 | 0.015 | 0.197 | 2.11E-06 | 0.188 | 22.495 |
|  | rs12512971 | A | C | FALSE | 0.171 | -0.150 | 0.040 | 0.279 | 1.73E-05 | 0.592 | 18.467 |
|  | rs12908520 | G | A | FALSE | 0.059 | -0.022 | 0.013 | 0.150 | 3.49E-06 | 0.885 | 21.524 |
|  | rs2602429 | C | T | FALSE | 0.076 | 0.054 | 0.015 | 0.170 | 6.40E-07 | 0.753 | 24.786 |
|  | rs3995795 | C | T | FALSE | 0.061 | -0.052 | 0.014 | 0.153 | 1.04E-05 | 0.737 | 19.431 |
|  | rs45598138 | C | A | FALSE | -0.144 | 0.088 | 0.031 | 0.469 | 2.45E-06 | 0.851 | 22.202 |
|  | rs61779207 | G | A | FALSE | -0.076 | -0.065 | 0.016 | 0.180 | 4.34E-06 | 0.718 | 21.109 |
|  | rs74542928 | T | C | FALSE | 0.116 | -0.234 | 0.023 | 0.343 | 5.32E-07 | 0.496 | 25.144 |
|  | rs76430504 | T | C | FALSE | -0.118 | 0.089 | 0.025 | 0.340 | 3.90E-06 | 0.794 | 21.313 |
|  | rs9349825 | A | G | FALSE | -0.066 | 0.172 | 0.014 | 0.189 | 4.48E-06 | 0.362 | 21.048 |
