# Supplementary material for: Investigating the causal impact of gut microbiota on glioblastoma: a bidirectional Mendelian randomization study
Source: BMC Genomics. 2023 Dec 18;24:784. doi: 10.1186/s12864-023-09885-2 (PMC10726622; doi:10.1186/s12864-023-09885-2)
Supplement: Supplementary file 2 — Addtiional file 2: TableS2. The detail information of SNP on Glioblastoma (exposure). [file 12864_2023_9885_MOESM2_ESM.docx]

TableS2. The detail information of SNP on Glioblastoma (exposure)

| Bacterial taxa (outcome) | SNP | Effect  allele | Other  allele | BETA | | SE | | P.val | | palindromic | F |
| --- | --- | --- | --- | --- | --- | --- | --- | --- | --- | --- | --- |
|  |  |  |  | exposure | outcome | exposure | outcome | exposure | outcome |  |  |
| genus Clostridiuminnocuumgroup(id.14397) | rs10513202 | G | A | 1.427 | 0.016 | 0.321 | 0.035 | 8.58E-06 | 0.650 | FALSE | 20 |
|  | rs11090513 | T | G | 0.798 | -0.034 | 0.175 | 0.026 | 5.00E-06 | 0.194 | FALSE | 21 |
|  | rs11230859 | A | G | -0.725 | 0.033 | 0.158 | 0.023 | 4.18E-06 | 0.149 | FALSE | 21 |
|  | rs12669698 | C | T | 1.127 | 0.016 | 0.255 | 0.035 | 9.73E-06 | 0.640 | FALSE | 20 |
|  | rs17145573 | A | G | 1.708 | 0.037 | 0.365 | 0.035 | 2.87E-06 | 0.291 | FALSE | 22 |
|  | rs389558 | T | C | -0.783 | 0.011 | 0.160 | 0.022 | 9.74E-07 | 0.619 | FALSE | 24 |
|  | rs491806 | C | A | -0.948 | -0.027 | 0.211 | 0.027 | 6.92E-06 | 0.320 | FALSE | 20 |
|  | rs529324 | A | G | -0.826 | 0.005 | 0.179 | 0.024 | 4.16E-06 | 0.841 | FALSE | 21 |
|  | rs7778345 | G | A | 0.730 | 0.007 | 0.163 | 0.022 | 7.05E-06 | 0.769 | FALSE | 20 |
| genus Eubacteriumbrachygroup(id.11296) | rs10513202 | G | A | 1.427 | 0.005 | 0.321 | 0.036 | 8.58E-06 | 0.899 | FALSE | 20 |
|  | rs11090513 | T | G | 0.798 | 0.003 | 0.175 | 0.026 | 5.00E-06 | 0.898 | FALSE | 21 |
|  | rs11230859 | A | G | -0.725 | -0.011 | 0.158 | 0.024 | 4.18E-06 | 0.650 | FALSE | 21 |
|  | rs12669698 | C | T | 1.127 | 0.005 | 0.255 | 0.036 | 9.73E-06 | 0.883 | FALSE | 20 |
|  | rs17145573 | A | G | 1.708 | 0.043 | 0.365 | 0.036 | 2.87E-06 | 0.231 | FALSE | 22 |
|  | rs389558 | T | C | -0.783 | -0.019 | 0.160 | 0.023 | 9.74E-07 | 0.397 | FALSE | 24 |
|  | rs491806 | C | A | -0.948 | 0.003 | 0.211 | 0.027 | 6.92E-06 | 0.902 | FALSE | 20 |
|  | rs529324 | A | G | -0.826 | 0.006 | 0.179 | 0.024 | 4.16E-06 | 0.792 | FALSE | 21 |
|  | rs7778345 | G | A | 0.730 | 0.025 | 0.163 | 0.023 | 7.05E-06 | 0.269 | FALSE | 20 |
| genus Eubacteriumcoprostanoligenesgroup(id.11375) | rs10513202 | G | A | 1.427 | 0.016 | 0.321 | 0.017 | 8.58E-06 | 0.359 | FALSE | 20 |
|  | rs11090513 | T | G | 0.798 | -0.006 | 0.175 | 0.013 | 5.00E-06 | 0.610 | FALSE | 21 |
|  | rs11230859 | A | G | -0.725 | -0.009 | 0.158 | 0.011 | 4.18E-06 | 0.403 | FALSE | 21 |
|  | rs12669698 | C | T | 1.127 | -0.027 | 0.255 | 0.017 | 9.73E-06 | 0.104 | FALSE | 20 |
|  | rs17145573 | A | G | 1.708 | 0.006 | 0.365 | 0.017 | 2.87E-06 | 0.715 | FALSE | 22 |
|  | rs389558 | T | C | -0.783 | 0.002 | 0.160 | 0.011 | 9.74E-07 | 0.858 | FALSE | 24 |
|  | rs491806 | C | A | -0.948 | -0.004 | 0.211 | 0.013 | 6.92E-06 | 0.750 | FALSE | 20 |
|  | rs529324 | A | G | -0.826 | 0.002 | 0.179 | 0.012 | 4.16E-06 | 0.860 | FALSE | 21 |
|  | rs7778345 | G | A | 0.730 | 0.016 | 0.163 | 0.011 | 7.05E-06 | 0.159 | FALSE | 20 |
| genus Eubacteriumeligensgroup(id.14372) | rs10513202 | G | A | 1.427 | -0.017 | 0.321 | 0.019 | 8.58E-06 | 0.361 | FALSE | 20 |
|  | rs11090513 | T | G | 0.798 | 0.021 | 0.175 | 0.014 | 5.00E-06 | 0.120 | FALSE | 21 |
|  | rs11230859 | A | G | -0.725 | 0.007 | 0.158 | 0.012 | 4.18E-06 | 0.585 | FALSE | 21 |
|  | rs12669698 | C | T | 1.127 | -0.005 | 0.255 | 0.018 | 9.73E-06 | 0.772 | FALSE | 20 |
|  | rs17145573 | A | G | 1.708 | -0.018 | 0.365 | 0.019 | 2.87E-06 | 0.348 | FALSE | 22 |
|  | rs389558 | T | C | -0.783 | -0.007 | 0.160 | 0.012 | 9.74E-07 | 0.555 | FALSE | 24 |
|  | rs491806 | C | A | -0.948 | 0.003 | 0.211 | 0.014 | 6.92E-06 | 0.844 | FALSE | 20 |
|  | rs529324 | A | G | -0.826 | -0.031 | 0.179 | 0.013 | 4.16E-06 | 0.013 | FALSE | 21 |
|  | rs7778345 | G | A | 0.730 | 0.007 | 0.163 | 0.012 | 7.05E-06 | 0.535 | FALSE | 20 |
| genus Eubacteriumfissicatenagroup(id.14373) | rs10513202 | G | A | 1.427 | -0.001 | 0.321 | 0.037 | 8.58E-06 | 0.976 | FALSE | 20 |
|  | rs11090513 | T | G | 0.798 | 0.032 | 0.175 | 0.028 | 5.00E-06 | 0.240 | FALSE | 21 |
|  | rs11230859 | A | G | -0.725 | 0.000 | 0.158 | 0.024 | 4.18E-06 | 0.999 | FALSE | 21 |
|  | rs12669698 | C | T | 1.127 | 0.057 | 0.255 | 0.036 | 9.73E-06 | 0.113 | FALSE | 20 |
|  | rs17145573 | A | G | 1.708 | -0.010 | 0.365 | 0.037 | 2.87E-06 | 0.793 | FALSE | 22 |
|  | rs389558 | T | C | -0.783 | 0.047 | 0.160 | 0.024 | 9.74E-07 | 0.049 | FALSE | 24 |
|  | rs491806 | C | A | -0.948 | 0.042 | 0.211 | 0.028 | 6.92E-06 | 0.141 | FALSE | 20 |
|  | rs529324 | A | G | -0.826 | -0.009 | 0.179 | 0.025 | 4.16E-06 | 0.717 | FALSE | 21 |
|  | rs7778345 | G | A | 0.730 | 0.078 | 0.163 | 0.023 | 7.05E-06 | 0.001 | FALSE | 20 |
| genus Eubacteriumhalliigroup(id.11338) | rs10513202 | G | A | 1.427 | -0.018 | 0.321 | 0.018 | 8.58E-06 | 0.314 | FALSE | 20 |
|  | rs11090513 | T | G | 0.798 | -0.001 | 0.175 | 0.013 | 5.00E-06 | 0.930 | FALSE | 21 |
|  | rs11230859 | A | G | -0.725 | -0.013 | 0.158 | 0.012 | 4.18E-06 | 0.246 | FALSE | 21 |
|  | rs12669698 | C | T | 1.127 | 0.002 | 0.255 | 0.017 | 9.73E-06 | 0.885 | FALSE | 20 |
|  | rs17145573 | A | G | 1.708 | 0.031 | 0.365 | 0.018 | 2.87E-06 | 0.080 | FALSE | 22 |
|  | rs389558 | T | C | -0.783 | -0.009 | 0.160 | 0.011 | 9.74E-07 | 0.413 | FALSE | 24 |
|  | rs491806 | C | A | -0.948 | 0.003 | 0.211 | 0.014 | 6.92E-06 | 0.821 | FALSE | 20 |
|  | rs529324 | A | G | -0.826 | -0.001 | 0.179 | 0.012 | 4.16E-06 | 0.943 | FALSE | 21 |
|  | rs7778345 | G | A | 0.730 | 0.021 | 0.163 | 0.011 | 7.05E-06 | 0.059 | FALSE | 20 |
| genus Eubacteriumnodatumgroup(id.11297) | rs10513202 | G | A | 1.427 | -0.033 | 0.321 | 0.040 | 8.58E-06 | 0.411 | FALSE | 20 |
|  | rs11090513 | T | G | 0.798 | -0.005 | 0.175 | 0.029 | 5.00E-06 | 0.865 | FALSE | 21 |
|  | rs11230859 | A | G | -0.725 | -0.008 | 0.158 | 0.026 | 4.18E-06 | 0.766 | FALSE | 21 |
|  | rs12669698 | C | T | 1.127 | -0.039 | 0.255 | 0.040 | 9.73E-06 | 0.322 | FALSE | 20 |
|  | rs17145573 | A | G | 1.708 | 0.048 | 0.365 | 0.041 | 2.87E-06 | 0.235 | FALSE | 22 |
|  | rs389558 | T | C | -0.783 | -0.015 | 0.160 | 0.025 | 9.74E-07 | 0.555 | FALSE | 24 |
|  | rs491806 | C | A | -0.948 | 0.005 | 0.211 | 0.030 | 6.92E-06 | 0.882 | FALSE | 20 |
|  | rs529324 | A | G | -0.826 | 0.018 | 0.179 | 0.027 | 4.16E-06 | 0.502 | FALSE | 21 |
|  | rs7778345 | G | A | 0.730 | 0.026 | 0.163 | 0.025 | 7.05E-06 | 0.290 | FALSE | 20 |
| genus Eubacteriumoxidoreducensgroup(id.11339) | rs10513202 | G | A | 1.427 | -0.048 | 0.321 | 0.031 | 8.58E-06 | 0.127 | FALSE | 20 |
|  | rs11090513 | T | G | 0.798 | 0.029 | 0.175 | 0.023 | 5.00E-06 | 0.204 | FALSE | 21 |
|  | rs11230859 | A | G | -0.725 | 0.005 | 0.158 | 0.020 | 4.18E-06 | 0.788 | FALSE | 21 |
|  | rs12669698 | C | T | 1.127 | 0.011 | 0.255 | 0.030 | 9.73E-06 | 0.722 | FALSE | 20 |
|  | rs17145573 | A | G | 1.708 | -0.040 | 0.365 | 0.030 | 2.87E-06 | 0.183 | FALSE | 22 |
|  | rs389558 | T | C | -0.783 | 0.000 | 0.160 | 0.020 | 9.74E-07 | 0.989 | FALSE | 24 |
|  | rs491806 | C | A | -0.948 | 0.052 | 0.211 | 0.024 | 6.92E-06 | 0.033 | FALSE | 20 |
|  | rs529324 | A | G | -0.826 | 0.007 | 0.179 | 0.021 | 4.16E-06 | 0.753 | FALSE | 21 |
|  | rs7778345 | G | A | 0.730 | 0.000 | 0.163 | 0.020 | 7.05E-06 | 0.985 | FALSE | 20 |
| genus Eubacteriumrectalegroup(id.14374) | rs10513202 | G | A | 1.427 | -0.003 | 0.321 | 0.017 | 8.58E-06 | 0.883 | FALSE | 20 |
|  | rs11090513 | T | G | 0.798 | -0.012 | 0.175 | 0.013 | 5.00E-06 | 0.353 | FALSE | 21 |
|  | rs11230859 | A | G | -0.725 | -0.009 | 0.158 | 0.011 | 4.18E-06 | 0.436 | FALSE | 21 |
|  | rs12669698 | C | T | 1.127 | -0.007 | 0.255 | 0.017 | 9.73E-06 | 0.658 | FALSE | 20 |
|  | rs17145573 | A | G | 1.708 | 0.018 | 0.365 | 0.017 | 2.87E-06 | 0.294 | FALSE | 22 |
|  | rs389558 | T | C | -0.783 | -0.009 | 0.160 | 0.011 | 9.74E-07 | 0.401 | FALSE | 24 |
|  | rs491806 | C | A | -0.948 | 0.013 | 0.211 | 0.013 | 6.92E-06 | 0.343 | FALSE | 20 |
|  | rs529324 | A | G | -0.826 | -0.004 | 0.179 | 0.012 | 4.16E-06 | 0.729 | FALSE | 21 |
|  | rs7778345 | G | A | 0.730 | -0.002 | 0.163 | 0.011 | 7.05E-06 | 0.886 | FALSE | 20 |
| genus Eubacteriumruminantiumgroup(id.11340) | rs10513202 | G | A | 1.427 | 0.023 | 0.321 | 0.026 | 8.58E-06 | 0.372 | FALSE | 20 |
|  | rs11090513 | T | G | 0.798 | 0.054 | 0.175 | 0.019 | 5.00E-06 | 0.005 | FALSE | 21 |
|  | rs11230859 | A | G | -0.725 | 0.011 | 0.158 | 0.017 | 4.18E-06 | 0.520 | FALSE | 21 |
|  | rs12669698 | C | T | 1.127 | 0.003 | 0.255 | 0.025 | 9.73E-06 | 0.890 | FALSE | 20 |
|  | rs17145573 | A | G | 1.708 | -0.005 | 0.365 | 0.026 | 2.87E-06 | 0.837 | FALSE | 22 |
|  | rs389558 | T | C | -0.783 | -0.002 | 0.160 | 0.017 | 9.74E-07 | 0.902 | FALSE | 24 |
|  | rs491806 | C | A | -0.948 | -0.017 | 0.211 | 0.020 | 6.92E-06 | 0.404 | FALSE | 20 |
|  | rs529324 | A | G | -0.826 | 0.023 | 0.179 | 0.017 | 4.16E-06 | 0.194 | FALSE | 21 |
|  | rs7778345 | G | A | 0.730 | 0.025 | 0.163 | 0.017 | 7.05E-06 | 0.134 | FALSE | 20 |
| genus Eubacteriumventriosumgroup(id.11341) | rs10513202 | G | A | 1.427 | -0.015 | 0.321 | 0.019 | 8.58E-06 | 0.408 | FALSE | 20 |
|  | rs11090513 | T | G | 0.798 | -0.001 | 0.175 | 0.013 | 5.00E-06 | 0.951 | FALSE | 21 |
|  | rs11230859 | A | G | -0.725 | 0.000 | 0.158 | 0.012 | 4.18E-06 | 0.968 | FALSE | 21 |
|  | rs12669698 | C | T | 1.127 | 0.008 | 0.255 | 0.018 | 9.73E-06 | 0.648 | FALSE | 20 |
|  | rs17145573 | A | G | 1.708 | -0.013 | 0.365 | 0.018 | 2.87E-06 | 0.488 | FALSE | 22 |
|  | rs389558 | T | C | -0.783 | -0.004 | 0.160 | 0.012 | 9.74E-07 | 0.749 | FALSE | 24 |
|  | rs491806 | C | A | -0.948 | 0.022 | 0.211 | 0.014 | 6.92E-06 | 0.112 | FALSE | 20 |
|  | rs529324 | A | G | -0.826 | -0.019 | 0.179 | 0.012 | 4.16E-06 | 0.117 | FALSE | 21 |
|  | rs7778345 | G | A | 0.730 | 0.000 | 0.163 | 0.012 | 7.05E-06 | 0.973 | FALSE | 20 |
| genus Eubacteriumxylanophilumgroup(id.14375) | rs10513202 | G | A | 1.427 | -0.042 | 0.321 | 0.020 | 8.58E-06 | 0.035 | FALSE | 20 |
|  | rs11090513 | T | G | 0.798 | 0.019 | 0.175 | 0.014 | 5.00E-06 | 0.181 | FALSE | 21 |
|  | rs11230859 | A | G | -0.725 | -0.007 | 0.158 | 0.013 | 4.18E-06 | 0.567 | FALSE | 21 |
|  | rs12669698 | C | T | 1.127 | -0.012 | 0.255 | 0.020 | 9.73E-06 | 0.552 | FALSE | 20 |
|  | rs17145573 | A | G | 1.708 | -0.014 | 0.365 | 0.020 | 2.87E-06 | 0.494 | FALSE | 22 |
|  | rs389558 | T | C | -0.783 | -0.014 | 0.160 | 0.013 | 9.74E-07 | 0.257 | FALSE | 24 |
|  | rs491806 | C | A | -0.948 | 0.010 | 0.211 | 0.015 | 6.92E-06 | 0.501 | FALSE | 20 |
|  | rs529324 | A | G | -0.826 | 0.012 | 0.179 | 0.013 | 4.16E-06 | 0.359 | FALSE | 21 |
|  | rs7778345 | G | A | 0.730 | 0.010 | 0.163 | 0.012 | 7.05E-06 | 0.408 | FALSE | 20 |
| genus Ruminococcusgauvreauiigroup(id.11342) | rs10513202 | G | A | 1.427 | -0.019 | 0.321 | 0.020 | 8.58E-06 | 0.329 | FALSE | 20 |
|  | rs11090513 | T | G | 0.798 | 0.020 | 0.175 | 0.014 | 5.00E-06 | 0.162 | FALSE | 21 |
|  | rs11230859 | A | G | -0.725 | -0.011 | 0.158 | 0.013 | 4.18E-06 | 0.399 | FALSE | 21 |
|  | rs12669698 | C | T | 1.127 | 0.019 | 0.255 | 0.019 | 9.73E-06 | 0.314 | FALSE | 20 |
|  | rs17145573 | A | G | 1.708 | 0.003 | 0.365 | 0.020 | 2.87E-06 | 0.874 | FALSE | 22 |
|  | rs389558 | T | C | -0.783 | 0.005 | 0.160 | 0.013 | 9.74E-07 | 0.699 | FALSE | 24 |
|  | rs491806 | C | A | -0.948 | 0.000 | 0.211 | 0.015 | 6.92E-06 | 0.985 | FALSE | 20 |
|  | rs529324 | A | G | -0.826 | -0.009 | 0.179 | 0.013 | 4.16E-06 | 0.500 | FALSE | 21 |
|  | rs7778345 | G | A | 0.730 | 0.008 | 0.163 | 0.013 | 7.05E-06 | 0.506 | FALSE | 20 |
| genus Ruminococcusgnavusgroup(id.14376) | rs10513202 | G | A | 1.427 | 0.051 | 0.321 | 0.029 | 8.58E-06 | 0.074 | FALSE | 20 |
|  | rs11090513 | T | G | 0.798 | -0.009 | 0.175 | 0.021 | 5.00E-06 | 0.658 | FALSE | 21 |
|  | rs11230859 | A | G | -0.725 | 0.008 | 0.158 | 0.018 | 4.18E-06 | 0.662 | FALSE | 21 |
|  | rs12669698 | C | T | 1.127 | -0.039 | 0.255 | 0.027 | 9.73E-06 | 0.150 | FALSE | 20 |
|  | rs17145573 | A | G | 1.708 | 0.013 | 0.365 | 0.028 | 2.87E-06 | 0.658 | FALSE | 22 |
|  | rs389558 | T | C | -0.783 | 0.012 | 0.160 | 0.018 | 9.74E-07 | 0.519 | FALSE | 24 |
|  | rs491806 | C | A | -0.948 | 0.006 | 0.211 | 0.022 | 6.92E-06 | 0.775 | FALSE | 20 |
|  | rs529324 | A | G | -0.826 | 0.014 | 0.179 | 0.019 | 4.16E-06 | 0.470 | FALSE | 21 |
|  | rs7778345 | G | A | 0.730 | 0.008 | 0.163 | 0.018 | 7.05E-06 | 0.665 | FALSE | 20 |
| genus Ruminococcustorquesgroup(id.14377) | rs10513202 | G | A | 1.427 | -0.020 | 0.321 | 0.017 | 8.58E-06 | 0.250 | FALSE | 20 |
|  | rs11090513 | T | G | 0.798 | -0.016 | 0.175 | 0.012 | 5.00E-06 | 0.195 | FALSE | 21 |
|  | rs11230859 | A | G | -0.725 | -0.008 | 0.158 | 0.011 | 4.18E-06 | 0.446 | FALSE | 21 |
|  | rs12669698 | C | T | 1.127 | 0.035 | 0.255 | 0.017 | 9.73E-06 | 0.035 | FALSE | 20 |
|  | rs17145573 | A | G | 1.708 | 0.012 | 0.365 | 0.017 | 2.87E-06 | 0.483 | FALSE | 22 |
|  | rs389558 | T | C | -0.783 | -0.005 | 0.160 | 0.011 | 9.74E-07 | 0.654 | FALSE | 24 |
|  | rs491806 | C | A | -0.948 | -0.029 | 0.211 | 0.013 | 6.92E-06 | 0.024 | FALSE | 20 |
|  | rs529324 | A | G | -0.826 | -0.012 | 0.179 | 0.011 | 4.16E-06 | 0.316 | FALSE | 21 |
|  | rs7778345 | G | A | 0.730 | 0.015 | 0.163 | 0.011 | 7.05E-06 | 0.182 | FALSE | 20 |
| genus.Actinomyces(id.423) | rs10513202 | G | A | 1.427 | 0.015 | 0.321 | 0.026 | 8.58E-06 | 0.572 | FALSE | 20 |
|  | rs11090513 | T | G | 0.798 | -0.032 | 0.175 | 0.019 | 5.00E-06 | 0.093 | FALSE | 21 |
|  | rs11230859 | A | G | -0.725 | -0.007 | 0.158 | 0.017 | 4.18E-06 | 0.686 | FALSE | 21 |
|  | rs12669698 | C | T | 1.127 | 0.015 | 0.255 | 0.027 | 9.73E-06 | 0.572 | FALSE | 20 |
|  | rs17145573 | A | G | 1.708 | -0.015 | 0.365 | 0.027 | 2.87E-06 | 0.577 | FALSE | 22 |
|  | rs389558 | T | C | -0.783 | -0.003 | 0.160 | 0.016 | 9.74E-07 | 0.876 | FALSE | 24 |
|  | rs491806 | C | A | -0.948 | -0.006 | 0.211 | 0.020 | 6.92E-06 | 0.773 | FALSE | 20 |
|  | rs529324 | A | G | -0.826 | -0.013 | 0.179 | 0.018 | 4.16E-06 | 0.450 | FALSE | 21 |
|  | rs7778345 | G | A | 0.730 | -0.019 | 0.163 | 0.017 | 7.05E-06 | 0.266 | FALSE | 20 |
| genus.Adlercreutzia(id.812) | rs10513202 | G | A | 1.427 | -0.005 | 0.321 | 0.025 | 8.58E-06 | 0.842 | FALSE | 20 |
|  | rs11090513 | T | G | 0.798 | -0.002 | 0.175 | 0.018 | 5.00E-06 | 0.897 | FALSE | 21 |
|  | rs11230859 | A | G | -0.725 | 0.015 | 0.158 | 0.016 | 4.18E-06 | 0.344 | FALSE | 21 |
|  | rs12669698 | C | T | 1.127 | 0.032 | 0.255 | 0.025 | 9.73E-06 | 0.201 | FALSE | 20 |
|  | rs17145573 | A | G | 1.708 | -0.012 | 0.365 | 0.025 | 2.87E-06 | 0.647 | FALSE | 22 |
|  | rs389558 | T | C | -0.783 | 0.006 | 0.160 | 0.016 | 9.74E-07 | 0.706 | FALSE | 24 |
|  | rs491806 | C | A | -0.948 | 0.006 | 0.211 | 0.019 | 6.92E-06 | 0.755 | FALSE | 20 |
|  | rs529324 | A | G | -0.826 | 0.017 | 0.179 | 0.017 | 4.16E-06 | 0.319 | FALSE | 21 |
|  | rs7778345 | G | A | 0.730 | 0.017 | 0.163 | 0.016 | 7.05E-06 | 0.286 | FALSE | 20 |
| genus.Akkermansia(id.4037) | rs10513202 | G | A | 1.427 | -0.016 | 0.321 | 0.021 | 8.58E-06 | 0.445 | FALSE | 20 |
|  | rs11090513 | T | G | 0.798 | 0.013 | 0.175 | 0.015 | 5.00E-06 | 0.406 | FALSE | 21 |
|  | rs11230859 | A | G | -0.725 | 0.015 | 0.158 | 0.014 | 4.18E-06 | 0.286 | FALSE | 21 |
|  | rs12669698 | C | T | 1.127 | 0.003 | 0.255 | 0.020 | 9.73E-06 | 0.874 | FALSE | 20 |
|  | rs17145573 | A | G | 1.708 | 0.003 | 0.365 | 0.021 | 2.87E-06 | 0.871 | FALSE | 22 |
|  | rs389558 | T | C | -0.783 | 0.005 | 0.160 | 0.013 | 9.74E-07 | 0.692 | FALSE | 24 |
|  | rs491806 | C | A | -0.948 | 0.014 | 0.211 | 0.016 | 6.92E-06 | 0.394 | FALSE | 20 |
|  | rs529324 | A | G | -0.826 | -0.004 | 0.179 | 0.014 | 4.16E-06 | 0.755 | FALSE | 21 |
|  | rs7778345 | G | A | 0.730 | 0.011 | 0.163 | 0.013 | 7.05E-06 | 0.387 | FALSE | 20 |
| genus.Alistipes(id.968) | rs10513202 | G | A | 1.427 | -0.007 | 0.321 | 0.017 | 8.58E-06 | 0.699 | FALSE | 20 |
|  | rs11090513 | T | G | 0.798 | 0.008 | 0.175 | 0.013 | 5.00E-06 | 0.538 | FALSE | 21 |
|  | rs11230859 | A | G | -0.725 | -0.005 | 0.158 | 0.011 | 4.18E-06 | 0.639 | FALSE | 21 |
|  | rs12669698 | C | T | 1.127 | 0.017 | 0.255 | 0.017 | 9.73E-06 | 0.315 | FALSE | 20 |
|  | rs17145573 | A | G | 1.708 | -0.022 | 0.365 | 0.017 | 2.87E-06 | 0.203 | FALSE | 22 |
|  | rs389558 | T | C | -0.783 | 0.002 | 0.160 | 0.011 | 9.74E-07 | 0.832 | FALSE | 24 |
|  | rs491806 | C | A | -0.948 | 0.002 | 0.211 | 0.013 | 6.92E-06 | 0.890 | FALSE | 20 |
|  | rs529324 | A | G | -0.826 | 0.006 | 0.179 | 0.012 | 4.16E-06 | 0.578 | FALSE | 21 |
|  | rs7778345 | G | A | 0.730 | -0.003 | 0.163 | 0.011 | 7.05E-06 | 0.799 | FALSE | 20 |
| genus.Allisonella(id.2174) | rs10513202 | G | A | 1.427 | -0.043 | 0.321 | 0.041 | 8.58E-06 | 0.295 | FALSE | 20 |
|  | rs11090513 | T | G | 0.798 | -0.017 | 0.175 | 0.029 | 5.00E-06 | 0.553 | FALSE | 21 |
|  | rs11230859 | A | G | -0.725 | -0.052 | 0.158 | 0.026 | 4.18E-06 | 0.047 | FALSE | 21 |
|  | rs12669698 | C | T | 1.127 | 0.075 | 0.255 | 0.039 | 9.73E-06 | 0.053 | FALSE | 20 |
|  | rs17145573 | A | G | 1.708 | 0.036 | 0.365 | 0.039 | 2.87E-06 | 0.361 | FALSE | 22 |
|  | rs389558 | T | C | -0.783 | -0.022 | 0.160 | 0.025 | 9.74E-07 | 0.392 | FALSE | 24 |
|  | rs491806 | C | A | -0.948 | -0.012 | 0.211 | 0.031 | 6.92E-06 | 0.694 | FALSE | 20 |
|  | rs529324 | A | G | -0.826 | 0.023 | 0.179 | 0.027 | 4.16E-06 | 0.394 | FALSE | 21 |
|  | rs7778345 | G | A | 0.730 | -0.014 | 0.163 | 0.026 | 7.05E-06 | 0.598 | FALSE | 20 |
| genus.Alloprevotella(id.961) | rs10513202 | G | A | 1.427 | -0.017 | 0.321 | 0.042 | 8.58E-06 | 0.687 | FALSE | 20 |
|  | rs11230859 | A | G | -0.725 | -0.006 | 0.158 | 0.027 | 4.18E-06 | 0.832 | FALSE | 21 |
|  | rs12669698 | C | T | 1.127 | 0.042 | 0.255 | 0.039 | 9.73E-06 | 0.276 | FALSE | 20 |
|  | rs491806 | C | A | -0.948 | 0.073 | 0.211 | 0.032 | 6.92E-06 | 0.023 | FALSE | 20 |
|  | rs529324 | A | G | -0.826 | 0.050 | 0.179 | 0.028 | 4.16E-06 | 0.073 | FALSE | 21 |
|  | rs7778345 | G | A | 0.730 | -0.001 | 0.163 | 0.027 | 7.05E-06 | 0.968 | FALSE | 20 |
| genus.Anaerofilum(id.2053) | rs10513202 | G | A | 1.427 | 0.043 | 0.321 | 0.033 | 8.58E-06 | 0.188 | FALSE | 20 |
|  | rs11090513 | T | G | 0.798 | 0.007 | 0.175 | 0.023 | 5.00E-06 | 0.763 | FALSE | 21 |
|  | rs11230859 | A | G | -0.725 | -0.008 | 0.158 | 0.021 | 4.18E-06 | 0.713 | FALSE | 21 |
|  | rs12669698 | C | T | 1.127 | 0.030 | 0.255 | 0.032 | 9.73E-06 | 0.342 | FALSE | 20 |
|  | rs17145573 | A | G | 1.708 | 0.012 | 0.365 | 0.033 | 2.87E-06 | 0.721 | FALSE | 22 |
|  | rs389558 | T | C | -0.783 | -0.005 | 0.160 | 0.020 | 9.74E-07 | 0.793 | FALSE | 24 |
|  | rs491806 | C | A | -0.948 | -0.056 | 0.211 | 0.025 | 6.92E-06 | 0.023 | FALSE | 20 |
|  | rs529324 | A | G | -0.826 | -0.033 | 0.179 | 0.022 | 4.16E-06 | 0.130 | FALSE | 21 |
|  | rs7778345 | G | A | 0.730 | -0.003 | 0.163 | 0.020 | 7.05E-06 | 0.880 | FALSE | 20 |
| genus.Anaerostipes(id.1991) | rs10513202 | G | A | 1.427 | -0.012 | 0.321 | 0.017 | 8.58E-06 | 0.484 | FALSE | 20 |
|  | rs11090513 | T | G | 0.798 | -0.008 | 0.175 | 0.013 | 5.00E-06 | 0.557 | FALSE | 21 |
|  | rs11230859 | A | G | -0.725 | -0.014 | 0.158 | 0.011 | 4.18E-06 | 0.235 | FALSE | 21 |
|  | rs12669698 | C | T | 1.127 | 0.008 | 0.255 | 0.017 | 9.73E-06 | 0.656 | FALSE | 20 |
|  | rs17145573 | A | G | 1.708 | 0.006 | 0.365 | 0.017 | 2.87E-06 | 0.728 | FALSE | 22 |
|  | rs389558 | T | C | -0.783 | 0.008 | 0.160 | 0.011 | 9.74E-07 | 0.491 | FALSE | 24 |
|  | rs491806 | C | A | -0.948 | -0.010 | 0.211 | 0.013 | 6.92E-06 | 0.473 | FALSE | 20 |
|  | rs529324 | A | G | -0.826 | 0.009 | 0.179 | 0.012 | 4.16E-06 | 0.463 | FALSE | 21 |
|  | rs7778345 | G | A | 0.730 | -0.010 | 0.163 | 0.011 | 7.05E-06 | 0.389 | FALSE | 20 |
| genus.Anaerotruncus(id.2054) | rs10513202 | G | A | 1.427 | 0.015 | 0.321 | 0.018 | 8.58E-06 | 0.397 | FALSE | 20 |
|  | rs11090513 | T | G | 0.798 | -0.002 | 0.175 | 0.013 | 5.00E-06 | 0.891 | FALSE | 21 |
|  | rs11230859 | A | G | -0.725 | 0.011 | 0.158 | 0.012 | 4.18E-06 | 0.337 | FALSE | 21 |
|  | rs12669698 | C | T | 1.127 | -0.019 | 0.255 | 0.017 | 9.73E-06 | 0.273 | FALSE | 20 |
|  | rs17145573 | A | G | 1.708 | 0.012 | 0.365 | 0.018 | 2.87E-06 | 0.489 | FALSE | 22 |
|  | rs389558 | T | C | -0.783 | -0.005 | 0.160 | 0.011 | 9.74E-07 | 0.678 | FALSE | 24 |
|  | rs491806 | C | A | -0.948 | 0.009 | 0.211 | 0.014 | 6.92E-06 | 0.521 | FALSE | 20 |
|  | rs529324 | A | G | -0.826 | -0.009 | 0.179 | 0.012 | 4.16E-06 | 0.440 | FALSE | 21 |
|  | rs7778345 | G | A | 0.730 | 0.023 | 0.163 | 0.011 | 7.05E-06 | 0.043 | FALSE | 20 |
| genus.Bacteroides(id.918) | rs10513202 | G | A | 1.427 | -0.013 | 0.321 | 0.017 | 8.58E-06 | 0.424 | FALSE | 20 |
|  | rs11090513 | T | G | 0.798 | -0.013 | 0.175 | 0.012 | 5.00E-06 | 0.310 | FALSE | 21 |
|  | rs11230859 | A | G | -0.725 | -0.009 | 0.158 | 0.011 | 4.18E-06 | 0.391 | FALSE | 21 |
|  | rs12669698 | C | T | 1.127 | 0.009 | 0.255 | 0.016 | 9.73E-06 | 0.570 | FALSE | 20 |
|  | rs17145573 | A | G | 1.708 | -0.020 | 0.365 | 0.017 | 2.87E-06 | 0.244 | FALSE | 22 |
|  | rs389558 | T | C | -0.783 | -0.004 | 0.160 | 0.011 | 9.74E-07 | 0.743 | FALSE | 24 |
|  | rs491806 | C | A | -0.948 | -0.023 | 0.211 | 0.013 | 6.92E-06 | 0.076 | FALSE | 20 |
|  | rs529324 | A | G | -0.826 | -0.012 | 0.179 | 0.011 | 4.16E-06 | 0.307 | FALSE | 21 |
|  | rs7778345 | G | A | 0.730 | -0.003 | 0.163 | 0.011 | 7.05E-06 | 0.768 | FALSE | 20 |
| genus.Barnesiella(id.944) | rs10513202 | G | A | 1.427 | -0.027 | 0.321 | 0.019 | 8.58E-06 | 0.153 | FALSE | 20 |
|  | rs11090513 | T | G | 0.798 | -0.002 | 0.175 | 0.014 | 5.00E-06 | 0.867 | FALSE | 21 |
|  | rs11230859 | A | G | -0.725 | 0.000 | 0.158 | 0.012 | 4.18E-06 | 0.996 | FALSE | 21 |
|  | rs12669698 | C | T | 1.127 | 0.008 | 0.255 | 0.019 | 9.73E-06 | 0.661 | FALSE | 20 |
|  | rs17145573 | A | G | 1.708 | 0.007 | 0.365 | 0.019 | 2.87E-06 | 0.732 | FALSE | 22 |
|  | rs389558 | T | C | -0.783 | 0.020 | 0.160 | 0.012 | 9.74E-07 | 0.092 | FALSE | 24 |
|  | rs491806 | C | A | -0.948 | 0.000 | 0.211 | 0.015 | 6.92E-06 | 0.976 | FALSE | 20 |
|  | rs529324 | A | G | -0.826 | -0.013 | 0.179 | 0.013 | 4.16E-06 | 0.310 | FALSE | 21 |
|  | rs7778345 | G | A | 0.730 | 0.006 | 0.163 | 0.012 | 7.05E-06 | 0.625 | FALSE | 20 |
| genus.Bifidobacterium(id.436) | rs10513202 | G | A | 1.427 | 0.002 | 0.321 | 0.019 | 8.58E-06 | 0.912 | FALSE | 20 |
|  | rs11090513 | T | G | 0.798 | -0.015 | 0.175 | 0.014 | 5.00E-06 | 0.285 | FALSE | 21 |
|  | rs11230859 | A | G | -0.725 | -0.001 | 0.158 | 0.012 | 4.18E-06 | 0.960 | FALSE | 21 |
|  | rs12669698 | C | T | 1.127 | -0.027 | 0.255 | 0.018 | 9.73E-06 | 0.129 | FALSE | 20 |
|  | rs17145573 | A | G | 1.708 | 0.041 | 0.365 | 0.019 | 2.87E-06 | 0.027 | FALSE | 22 |
|  | rs389558 | T | C | -0.783 | -0.001 | 0.160 | 0.012 | 9.74E-07 | 0.941 | FALSE | 24 |
|  | rs491806 | C | A | -0.948 | -0.008 | 0.211 | 0.014 | 6.92E-06 | 0.597 | FALSE | 20 |
|  | rs529324 | A | G | -0.826 | -0.030 | 0.179 | 0.013 | 4.16E-06 | 0.018 | FALSE | 21 |
|  | rs7778345 | G | A | 0.730 | -0.005 | 0.163 | 0.012 | 7.05E-06 | 0.659 | FALSE | 20 |
| genus.Bilophila(id.3170) | rs10513202 | G | A | 1.427 | 0.024 | 0.321 | 0.020 | 8.58E-06 | 0.236 | FALSE | 20 |
|  | rs11090513 | T | G | 0.798 | 0.000 | 0.175 | 0.015 | 5.00E-06 | 0.999 | FALSE | 21 |
|  | rs11230859 | A | G | -0.725 | -0.001 | 0.158 | 0.013 | 4.18E-06 | 0.965 | FALSE | 21 |
|  | rs12669698 | C | T | 1.127 | 0.001 | 0.255 | 0.019 | 9.73E-06 | 0.947 | FALSE | 20 |
|  | rs17145573 | A | G | 1.708 | -0.017 | 0.365 | 0.020 | 2.87E-06 | 0.407 | FALSE | 22 |
|  | rs389558 | T | C | -0.783 | 0.014 | 0.160 | 0.013 | 9.74E-07 | 0.283 | FALSE | 24 |
|  | rs491806 | C | A | -0.948 | -0.005 | 0.211 | 0.015 | 6.92E-06 | 0.727 | FALSE | 20 |
|  | rs529324 | A | G | -0.826 | 0.002 | 0.179 | 0.014 | 4.16E-06 | 0.868 | FALSE | 21 |
|  | rs7778345 | G | A | 0.730 | 0.011 | 0.163 | 0.013 | 7.05E-06 | 0.409 | FALSE | 20 |
| genus.Blautia(id.1992) | rs10513202 | G | A | 1.427 | -0.025 | 0.321 | 0.017 | 8.58E-06 | 0.135 | FALSE | 20 |
|  | rs11090513 | T | G | 0.798 | -0.005 | 0.175 | 0.012 | 5.00E-06 | 0.710 | FALSE | 21 |
|  | rs11230859 | A | G | -0.725 | -0.007 | 0.158 | 0.011 | 4.18E-06 | 0.520 | FALSE | 21 |
|  | rs12669698 | C | T | 1.127 | -0.008 | 0.255 | 0.016 | 9.73E-06 | 0.620 | FALSE | 20 |
|  | rs17145573 | A | G | 1.708 | 0.030 | 0.365 | 0.017 | 2.87E-06 | 0.077 | FALSE | 22 |
|  | rs389558 | T | C | -0.783 | -0.006 | 0.160 | 0.011 | 9.74E-07 | 0.594 | FALSE | 24 |
|  | rs491806 | C | A | -0.948 | 0.001 | 0.211 | 0.013 | 6.92E-06 | 0.912 | FALSE | 20 |
|  | rs529324 | A | G | -0.826 | -0.004 | 0.179 | 0.011 | 4.16E-06 | 0.695 | FALSE | 21 |
|  | rs7778345 | G | A | 0.730 | 0.000 | 0.163 | 0.011 | 7.05E-06 | 0.980 | FALSE | 20 |
| genus.Butyricicoccus(id.2055) | rs10513202 | G | A | 1.427 | -0.006 | 0.321 | 0.017 | 8.58E-06 | 0.717 | FALSE | 20 |
|  | rs11090513 | T | G | 0.798 | -0.007 | 0.175 | 0.013 | 5.00E-06 | 0.588 | FALSE | 21 |
|  | rs11230859 | A | G | -0.725 | -0.001 | 0.158 | 0.011 | 4.18E-06 | 0.905 | FALSE | 21 |
|  | rs12669698 | C | T | 1.127 | 0.002 | 0.255 | 0.017 | 9.73E-06 | 0.895 | FALSE | 20 |
|  | rs17145573 | A | G | 1.708 | 0.003 | 0.365 | 0.017 | 2.87E-06 | 0.883 | FALSE | 22 |
|  | rs389558 | T | C | -0.783 | 0.018 | 0.160 | 0.011 | 9.74E-07 | 0.092 | FALSE | 24 |
|  | rs491806 | C | A | -0.948 | -0.008 | 0.211 | 0.013 | 6.92E-06 | 0.531 | FALSE | 20 |
|  | rs529324 | A | G | -0.826 | 0.000 | 0.179 | 0.012 | 4.16E-06 | 0.998 | FALSE | 21 |
|  | rs7778345 | G | A | 0.730 | 0.009 | 0.163 | 0.011 | 7.05E-06 | 0.399 | FALSE | 20 |
| genus.Butyricimonas(id.945) | rs10513202 | G | A | 1.427 | -0.001 | 0.321 | 0.022 | 8.58E-06 | 0.953 | FALSE | 20 |
|  | rs11090513 | T | G | 0.798 | -0.005 | 0.175 | 0.016 | 5.00E-06 | 0.765 | FALSE | 21 |
|  | rs11230859 | A | G | -0.725 | -0.006 | 0.158 | 0.014 | 4.18E-06 | 0.655 | FALSE | 21 |
|  | rs12669698 | C | T | 1.127 | 0.028 | 0.255 | 0.022 | 9.73E-06 | 0.196 | FALSE | 20 |
|  | rs17145573 | A | G | 1.708 | -0.032 | 0.365 | 0.022 | 2.87E-06 | 0.150 | FALSE | 22 |
|  | rs389558 | T | C | -0.783 | 0.001 | 0.160 | 0.014 | 9.74E-07 | 0.941 | FALSE | 24 |
|  | rs491806 | C | A | -0.948 | 0.013 | 0.211 | 0.017 | 6.92E-06 | 0.439 | FALSE | 20 |
|  | rs529324 | A | G | -0.826 | -0.010 | 0.179 | 0.015 | 4.16E-06 | 0.518 | FALSE | 21 |
|  | rs7778345 | G | A | 0.730 | 0.009 | 0.163 | 0.014 | 7.05E-06 | 0.501 | FALSE | 20 |
| genus.Butyrivibrio(id.1993) | rs10513202 | G | A | 1.427 | 0.061 | 0.321 | 0.038 | 8.58E-06 | 0.113 | FALSE | 20 |
|  | rs11090513 | T | G | 0.798 | 0.002 | 0.175 | 0.028 | 5.00E-06 | 0.941 | FALSE | 21 |
|  | rs11230859 | A | G | -0.725 | 0.028 | 0.158 | 0.025 | 4.18E-06 | 0.266 | FALSE | 21 |
|  | rs12669698 | C | T | 1.127 | 0.090 | 0.255 | 0.038 | 9.73E-06 | 0.019 | FALSE | 20 |
|  | rs17145573 | A | G | 1.708 | -0.007 | 0.365 | 0.039 | 2.87E-06 | 0.855 | FALSE | 22 |
|  | rs389558 | T | C | -0.783 | -0.021 | 0.160 | 0.025 | 9.74E-07 | 0.392 | FALSE | 24 |
|  | rs491806 | C | A | -0.948 | -0.022 | 0.211 | 0.029 | 6.92E-06 | 0.440 | FALSE | 20 |
|  | rs529324 | A | G | -0.826 | 0.026 | 0.179 | 0.026 | 4.16E-06 | 0.325 | FALSE | 21 |
|  | rs7778345 | G | A | 0.730 | 0.020 | 0.163 | 0.024 | 7.05E-06 | 0.411 | FALSE | 20 |
| genus.CandidatusSoleaferrea(id.11350) | rs10513202 | G | A | 1.427 | 0.029 | 0.321 | 0.029 | 8.58E-06 | 0.313 | FALSE | 20 |
|  | rs11090513 | T | G | 0.798 | -0.003 | 0.175 | 0.021 | 5.00E-06 | 0.883 | FALSE | 21 |
|  | rs11230859 | A | G | -0.725 | -0.022 | 0.158 | 0.019 | 4.18E-06 | 0.250 | FALSE | 21 |
|  | rs12669698 | C | T | 1.127 | 0.026 | 0.255 | 0.029 | 9.73E-06 | 0.376 | FALSE | 20 |
|  | rs17145573 | A | G | 1.708 | -0.056 | 0.365 | 0.029 | 2.87E-06 | 0.055 | FALSE | 22 |
|  | rs389558 | T | C | -0.783 | 0.001 | 0.160 | 0.018 | 9.74E-07 | 0.956 | FALSE | 24 |
|  | rs491806 | C | A | -0.948 | 0.015 | 0.211 | 0.022 | 6.92E-06 | 0.509 | FALSE | 20 |
|  | rs529324 | A | G | -0.826 | -0.031 | 0.179 | 0.020 | 4.16E-06 | 0.118 | FALSE | 21 |
|  | rs7778345 | G | A | 0.730 | -0.022 | 0.163 | 0.018 | 7.05E-06 | 0.228 | FALSE | 20 |
| genus.Catenibacterium(id.2153) | rs10513202 | G | A | 1.427 | 0.030 | 0.321 | 0.040 | 8.58E-06 | 0.455 | FALSE | 20 |
|  | rs11090513 | T | G | 0.798 | -0.054 | 0.175 | 0.029 | 5.00E-06 | 0.062 | FALSE | 21 |
|  | rs11230859 | A | G | -0.725 | -0.022 | 0.158 | 0.026 | 4.18E-06 | 0.387 | FALSE | 21 |
|  | rs12669698 | C | T | 1.127 | 0.001 | 0.255 | 0.037 | 9.73E-06 | 0.986 | FALSE | 20 |
|  | rs17145573 | A | G | 1.708 | -0.052 | 0.365 | 0.039 | 2.87E-06 | 0.184 | FALSE | 22 |
|  | rs389558 | T | C | -0.783 | 0.058 | 0.160 | 0.025 | 9.74E-07 | 0.020 | FALSE | 24 |
|  | rs491806 | C | A | -0.948 | 0.005 | 0.211 | 0.031 | 6.92E-06 | 0.864 | FALSE | 20 |
|  | rs529324 | A | G | -0.826 | -0.022 | 0.179 | 0.027 | 4.16E-06 | 0.401 | FALSE | 21 |
|  | rs7778345 | G | A | 0.730 | 0.043 | 0.163 | 0.026 | 7.05E-06 | 0.097 | FALSE | 20 |
| genus.ChristensenellaceaeR.7group(id.11283) | rs10513202 | G | A | 1.427 | -0.014 | 0.321 | 0.018 | 8.58E-06 | 0.426 | FALSE | 20 |
|  | rs11090513 | T | G | 0.798 | 0.007 | 0.175 | 0.013 | 5.00E-06 | 0.565 | FALSE | 21 |
|  | rs11230859 | A | G | -0.725 | 0.014 | 0.158 | 0.012 | 4.18E-06 | 0.232 | FALSE | 21 |
|  | rs12669698 | C | T | 1.127 | 0.000 | 0.255 | 0.017 | 9.73E-06 | 0.991 | FALSE | 20 |
|  | rs17145573 | A | G | 1.708 | -0.013 | 0.365 | 0.018 | 2.87E-06 | 0.469 | FALSE | 22 |
|  | rs389558 | T | C | -0.783 | -0.007 | 0.160 | 0.011 | 9.74E-07 | 0.526 | FALSE | 24 |
|  | rs491806 | C | A | -0.948 | -0.009 | 0.211 | 0.014 | 6.92E-06 | 0.515 | FALSE | 20 |
|  | rs529324 | A | G | -0.826 | 0.008 | 0.179 | 0.012 | 4.16E-06 | 0.507 | FALSE | 21 |
|  | rs7778345 | G | A | 0.730 | 0.008 | 0.163 | 0.011 | 7.05E-06 | 0.491 | FALSE | 20 |
| genus.Clostridiumsensustricto1(id.1873) | rs10513202 | G | A | 1.427 | -0.002 | 0.321 | 0.019 | 8.58E-06 | 0.913 | FALSE | 20 |
|  | rs11090513 | T | G | 0.798 | -0.007 | 0.175 | 0.014 | 5.00E-06 | 0.636 | FALSE | 21 |
|  | rs11230859 | A | G | -0.725 | 0.000 | 0.158 | 0.012 | 4.18E-06 | 0.968 | FALSE | 21 |
|  | rs12669698 | C | T | 1.127 | -0.005 | 0.255 | 0.019 | 9.73E-06 | 0.772 | FALSE | 20 |
|  | rs17145573 | A | G | 1.708 | -0.026 | 0.365 | 0.019 | 2.87E-06 | 0.174 | FALSE | 22 |
|  | rs389558 | T | C | -0.783 | 0.008 | 0.160 | 0.012 | 9.74E-07 | 0.527 | FALSE | 24 |
|  | rs491806 | C | A | -0.948 | 0.013 | 0.211 | 0.015 | 6.92E-06 | 0.364 | FALSE | 20 |
|  | rs529324 | A | G | -0.826 | 0.000 | 0.179 | 0.013 | 4.16E-06 | 0.983 | FALSE | 21 |
|  | rs7778345 | G | A | 0.730 | 0.016 | 0.163 | 0.012 | 7.05E-06 | 0.199 | FALSE | 20 |
| genus.Collinsella(id.815) | rs10513202 | G | A | 1.427 | -0.001 | 0.321 | 0.019 | 8.58E-06 | 0.957 | FALSE | 20 |
|  | rs11090513 | T | G | 0.798 | -0.021 | 0.175 | 0.014 | 5.00E-06 | 0.123 | FALSE | 21 |
|  | rs11230859 | A | G | -0.725 | -0.004 | 0.158 | 0.012 | 4.18E-06 | 0.768 | FALSE | 21 |
|  | rs12669698 | C | T | 1.127 | 0.012 | 0.255 | 0.018 | 9.73E-06 | 0.506 | FALSE | 20 |
|  | rs17145573 | A | G | 1.708 | 0.030 | 0.365 | 0.019 | 2.87E-06 | 0.115 | FALSE | 22 |
|  | rs389558 | T | C | -0.783 | 0.019 | 0.160 | 0.012 | 9.74E-07 | 0.117 | FALSE | 24 |
|  | rs491806 | C | A | -0.948 | -0.007 | 0.211 | 0.014 | 6.92E-06 | 0.621 | FALSE | 20 |
|  | rs529324 | A | G | -0.826 | 0.008 | 0.179 | 0.013 | 4.16E-06 | 0.530 | FALSE | 21 |
|  | rs7778345 | G | A | 0.730 | -0.002 | 0.163 | 0.012 | 7.05E-06 | 0.859 | FALSE | 20 |
| genus.Coprobacter(id.949) | rs10513202 | G | A | 1.427 | -0.018 | 0.321 | 0.028 | 8.58E-06 | 0.529 | FALSE | 20 |
|  | rs11090513 | T | G | 0.798 | 0.023 | 0.175 | 0.020 | 5.00E-06 | 0.267 | FALSE | 21 |
|  | rs11230859 | A | G | -0.725 | 0.002 | 0.158 | 0.018 | 4.18E-06 | 0.915 | FALSE | 21 |
|  | rs12669698 | C | T | 1.127 | 0.017 | 0.255 | 0.027 | 9.73E-06 | 0.538 | FALSE | 20 |
|  | rs17145573 | A | G | 1.708 | -0.045 | 0.365 | 0.028 | 2.87E-06 | 0.105 | FALSE | 22 |
|  | rs389558 | T | C | -0.783 | -0.003 | 0.160 | 0.018 | 9.74E-07 | 0.852 | FALSE | 24 |
|  | rs491806 | C | A | -0.948 | -0.017 | 0.211 | 0.021 | 6.92E-06 | 0.410 | FALSE | 20 |
|  | rs529324 | A | G | -0.826 | 0.042 | 0.179 | 0.019 | 4.16E-06 | 0.025 | FALSE | 21 |
|  | rs7778345 | G | A | 0.730 | -0.005 | 0.163 | 0.017 | 7.05E-06 | 0.758 | FALSE | 20 |
| genus.Coprococcus1(id.11301) | rs10513202 | G | A | 1.427 | -0.035 | 0.321 | 0.018 | 8.58E-06 | 0.049 | FALSE | 20 |
|  | rs11090513 | T | G | 0.798 | 0.006 | 0.175 | 0.013 | 5.00E-06 | 0.627 | FALSE | 21 |
|  | rs11230859 | A | G | -0.725 | 0.007 | 0.158 | 0.011 | 4.18E-06 | 0.553 | FALSE | 21 |
|  | rs12669698 | C | T | 1.127 | 0.000 | 0.255 | 0.017 | 9.73E-06 | 0.997 | FALSE | 20 |
|  | rs17145573 | A | G | 1.708 | -0.004 | 0.365 | 0.018 | 2.87E-06 | 0.829 | FALSE | 22 |
|  | rs389558 | T | C | -0.783 | -0.001 | 0.160 | 0.011 | 9.74E-07 | 0.937 | FALSE | 24 |
|  | rs491806 | C | A | -0.948 | -0.019 | 0.211 | 0.013 | 6.92E-06 | 0.167 | FALSE | 20 |
|  | rs529324 | A | G | -0.826 | 0.010 | 0.179 | 0.012 | 4.16E-06 | 0.397 | FALSE | 21 |
|  | rs7778345 | G | A | 0.730 | -0.004 | 0.163 | 0.011 | 7.05E-06 | 0.704 | FALSE | 20 |
| genus.Coprococcus2(id.11302) | rs10513202 | G | A | 1.427 | -0.035 | 0.321 | 0.021 | 8.58E-06 | 0.105 | FALSE | 20 |
|  | rs11090513 | T | G | 0.798 | 0.007 | 0.175 | 0.016 | 5.00E-06 | 0.664 | FALSE | 21 |
|  | rs11230859 | A | G | -0.725 | 0.000 | 0.158 | 0.014 | 4.18E-06 | 0.988 | FALSE | 21 |
|  | rs12669698 | C | T | 1.127 | -0.020 | 0.255 | 0.021 | 9.73E-06 | 0.340 | FALSE | 20 |
|  | rs17145573 | A | G | 1.708 | -0.021 | 0.365 | 0.021 | 2.87E-06 | 0.319 | FALSE | 22 |
|  | rs389558 | T | C | -0.783 | -0.012 | 0.160 | 0.014 | 9.74E-07 | 0.364 | FALSE | 24 |
|  | rs491806 | C | A | -0.948 | 0.009 | 0.211 | 0.016 | 6.92E-06 | 0.595 | FALSE | 20 |
|  | rs529324 | A | G | -0.826 | -0.025 | 0.179 | 0.014 | 4.16E-06 | 0.085 | FALSE | 21 |
|  | rs7778345 | G | A | 0.730 | 0.022 | 0.163 | 0.014 | 7.05E-06 | 0.099 | FALSE | 20 |
| genus.Coprococcus3(id.11303) | rs10513202 | G | A | 1.427 | -0.034 | 0.321 | 0.018 | 8.58E-06 | 0.059 | FALSE | 20 |
|  | rs11090513 | T | G | 0.798 | -0.014 | 0.175 | 0.013 | 5.00E-06 | 0.276 | FALSE | 21 |
|  | rs11230859 | A | G | -0.725 | -0.011 | 0.158 | 0.012 | 4.18E-06 | 0.340 | FALSE | 21 |
|  | rs12669698 | C | T | 1.127 | 0.003 | 0.255 | 0.018 | 9.73E-06 | 0.845 | FALSE | 20 |
|  | rs17145573 | A | G | 1.708 | 0.016 | 0.365 | 0.018 | 2.87E-06 | 0.388 | FALSE | 22 |
|  | rs389558 | T | C | -0.783 | 0.003 | 0.160 | 0.011 | 9.74E-07 | 0.797 | FALSE | 24 |
|  | rs491806 | C | A | -0.948 | -0.001 | 0.211 | 0.014 | 6.92E-06 | 0.949 | FALSE | 20 |
|  | rs529324 | A | G | -0.826 | 0.007 | 0.179 | 0.012 | 4.16E-06 | 0.579 | FALSE | 21 |
|  | rs7778345 | G | A | 0.730 | 0.013 | 0.163 | 0.012 | 7.05E-06 | 0.252 | FALSE | 20 |
| genus.DefluviitaleaceaeUCG011(id.11287) | rs10513202 | G | A | 1.427 | -0.038 | 0.321 | 0.025 | 8.58E-06 | 0.136 | FALSE | 20 |
|  | rs11090513 | T | G | 0.798 | 0.015 | 0.175 | 0.018 | 5.00E-06 | 0.405 | FALSE | 21 |
|  | rs11230859 | A | G | -0.725 | 0.044 | 0.158 | 0.016 | 4.18E-06 | 0.007 | FALSE | 21 |
|  | rs12669698 | C | T | 1.127 | -0.011 | 0.255 | 0.025 | 9.73E-06 | 0.653 | FALSE | 20 |
|  | rs17145573 | A | G | 1.708 | -0.013 | 0.365 | 0.026 | 2.87E-06 | 0.612 | FALSE | 22 |
|  | rs389558 | T | C | -0.783 | -0.018 | 0.160 | 0.016 | 9.74E-07 | 0.254 | FALSE | 24 |
|  | rs491806 | C | A | -0.948 | 0.034 | 0.211 | 0.019 | 6.92E-06 | 0.077 | FALSE | 20 |
|  | rs529324 | A | G | -0.826 | -0.034 | 0.179 | 0.017 | 4.16E-06 | 0.042 | FALSE | 21 |
|  | rs7778345 | G | A | 0.730 | -0.005 | 0.163 | 0.016 | 7.05E-06 | 0.740 | FALSE | 20 |
| genus.Desulfovibrio(id.3173) | rs10513202 | G | A | 1.427 | 0.023 | 0.321 | 0.024 | 8.58E-06 | 0.339 | FALSE | 20 |
|  | rs11090513 | T | G | 0.798 | -0.018 | 0.175 | 0.017 | 5.00E-06 | 0.304 | FALSE | 21 |
|  | rs11230859 | A | G | -0.725 | -0.005 | 0.158 | 0.015 | 4.18E-06 | 0.762 | FALSE | 21 |
|  | rs12669698 | C | T | 1.127 | -0.021 | 0.255 | 0.023 | 9.73E-06 | 0.372 | FALSE | 20 |
|  | rs17145573 | A | G | 1.708 | -0.031 | 0.365 | 0.024 | 2.87E-06 | 0.195 | FALSE | 22 |
|  | rs389558 | T | C | -0.783 | 0.006 | 0.160 | 0.015 | 9.74E-07 | 0.671 | FALSE | 24 |
|  | rs491806 | C | A | -0.948 | 0.006 | 0.211 | 0.018 | 6.92E-06 | 0.753 | FALSE | 20 |
|  | rs529324 | A | G | -0.826 | 0.014 | 0.179 | 0.016 | 4.16E-06 | 0.392 | FALSE | 21 |
|  | rs7778345 | G | A | 0.730 | 0.008 | 0.163 | 0.015 | 7.05E-06 | 0.579 | FALSE | 20 |
| genus.Dialister(id.2183) | rs10513202 | G | A | 1.427 | 0.013 | 0.321 | 0.021 | 8.58E-06 | 0.523 | FALSE | 20 |
|  | rs11090513 | T | G | 0.798 | -0.009 | 0.175 | 0.015 | 5.00E-06 | 0.541 | FALSE | 21 |
|  | rs11230859 | A | G | -0.725 | 0.000 | 0.158 | 0.013 | 4.18E-06 | 0.987 | FALSE | 21 |
|  | rs12669698 | C | T | 1.127 | -0.021 | 0.255 | 0.020 | 9.73E-06 | 0.299 | FALSE | 20 |
|  | rs17145573 | A | G | 1.708 | -0.025 | 0.365 | 0.021 | 2.87E-06 | 0.235 | FALSE | 22 |
|  | rs389558 | T | C | -0.783 | 0.009 | 0.160 | 0.013 | 9.74E-07 | 0.511 | FALSE | 24 |
|  | rs491806 | C | A | -0.948 | -0.004 | 0.211 | 0.016 | 6.92E-06 | 0.785 | FALSE | 20 |
|  | rs529324 | A | G | -0.826 | 0.023 | 0.179 | 0.014 | 4.16E-06 | 0.096 | FALSE | 21 |
|  | rs7778345 | G | A | 0.730 | 0.003 | 0.163 | 0.013 | 7.05E-06 | 0.798 | FALSE | 20 |
| genus.Dorea(id.1997) | rs10513202 | G | A | 1.427 | 0.002 | 0.321 | 0.017 | 8.58E-06 | 0.903 | FALSE | 20 |
|  | rs11090513 | T | G | 0.798 | 0.000 | 0.175 | 0.013 | 5.00E-06 | 0.992 | FALSE | 21 |
|  | rs11230859 | A | G | -0.725 | -0.022 | 0.158 | 0.011 | 4.18E-06 | 0.044 | FALSE | 21 |
|  | rs12669698 | C | T | 1.127 | -0.003 | 0.255 | 0.017 | 9.73E-06 | 0.878 | FALSE | 20 |
|  | rs17145573 | A | G | 1.708 | 0.022 | 0.365 | 0.017 | 2.87E-06 | 0.208 | FALSE | 22 |
|  | rs389558 | T | C | -0.783 | 0.003 | 0.160 | 0.011 | 9.74E-07 | 0.788 | FALSE | 24 |
|  | rs491806 | C | A | -0.948 | 0.015 | 0.211 | 0.013 | 6.92E-06 | 0.243 | FALSE | 20 |
|  | rs529324 | A | G | -0.826 | 0.007 | 0.179 | 0.012 | 4.16E-06 | 0.550 | FALSE | 21 |
|  | rs7778345 | G | A | 0.730 | 0.021 | 0.163 | 0.011 | 7.05E-06 | 0.061 | FALSE | 20 |
| genus.Eggerthella(id.819) | rs10513202 | G | A | 1.427 | -0.039 | 0.321 | 0.032 | 8.58E-06 | 0.223 | FALSE | 20 |
|  | rs11090513 | T | G | 0.798 | -0.035 | 0.175 | 0.023 | 5.00E-06 | 0.126 | FALSE | 21 |
|  | rs11230859 | A | G | -0.725 | 0.024 | 0.158 | 0.021 | 4.18E-06 | 0.239 | FALSE | 21 |
|  | rs12669698 | C | T | 1.127 | -0.022 | 0.255 | 0.031 | 9.73E-06 | 0.470 | FALSE | 20 |
|  | rs17145573 | A | G | 1.708 | 0.012 | 0.365 | 0.032 | 2.87E-06 | 0.703 | FALSE | 22 |
|  | rs389558 | T | C | -0.783 | 0.022 | 0.160 | 0.020 | 9.74E-07 | 0.271 | FALSE | 24 |
|  | rs491806 | C | A | -0.948 | -0.054 | 0.211 | 0.024 | 6.92E-06 | 0.027 | FALSE | 20 |
|  | rs529324 | A | G | -0.826 | 0.002 | 0.179 | 0.021 | 4.16E-06 | 0.936 | FALSE | 21 |
|  | rs7778345 | G | A | 0.730 | -0.008 | 0.163 | 0.020 | 7.05E-06 | 0.707 | FALSE | 20 |
| genus.Eisenbergiella(id.11304) | rs10513202 | G | A | 1.427 | -0.050 | 0.321 | 0.031 | 8.58E-06 | 0.099 | FALSE | 20 |
|  | rs11090513 | T | G | 0.798 | 0.011 | 0.175 | 0.022 | 5.00E-06 | 0.603 | FALSE | 21 |
|  | rs11230859 | A | G | -0.725 | 0.009 | 0.158 | 0.020 | 4.18E-06 | 0.647 | FALSE | 21 |
|  | rs12669698 | C | T | 1.127 | 0.023 | 0.255 | 0.030 | 9.73E-06 | 0.428 | FALSE | 20 |
|  | rs17145573 | A | G | 1.708 | 0.033 | 0.365 | 0.031 | 2.87E-06 | 0.284 | FALSE | 22 |
|  | rs389558 | T | C | -0.783 | 0.013 | 0.160 | 0.019 | 9.74E-07 | 0.486 | FALSE | 24 |
|  | rs491806 | C | A | -0.948 | 0.070 | 0.211 | 0.023 | 6.92E-06 | 0.003 | FALSE | 20 |
|  | rs529324 | A | G | -0.826 | -0.005 | 0.179 | 0.020 | 4.16E-06 | 0.825 | FALSE | 21 |
|  | rs7778345 | G | A | 0.730 | -0.006 | 0.163 | 0.019 | 7.05E-06 | 0.769 | FALSE | 20 |
| genus.Enterorhabdus(id.820) | rs10513202 | G | A | 1.427 | -0.051 | 0.321 | 0.026 | 8.58E-06 | 0.054 | FALSE | 20 |
|  | rs11090513 | T | G | 0.798 | 0.011 | 0.175 | 0.019 | 5.00E-06 | 0.560 | FALSE | 21 |
|  | rs11230859 | A | G | -0.725 | -0.006 | 0.158 | 0.017 | 4.18E-06 | 0.710 | FALSE | 21 |
|  | rs12669698 | C | T | 1.127 | 0.023 | 0.255 | 0.026 | 9.73E-06 | 0.391 | FALSE | 20 |
|  | rs17145573 | A | G | 1.708 | -0.038 | 0.365 | 0.027 | 2.87E-06 | 0.151 | FALSE | 22 |
|  | rs389558 | T | C | -0.783 | 0.005 | 0.160 | 0.016 | 9.74E-07 | 0.776 | FALSE | 24 |
|  | rs491806 | C | A | -0.948 | 0.010 | 0.211 | 0.020 | 6.92E-06 | 0.618 | FALSE | 20 |
|  | rs529324 | A | G | -0.826 | 0.000 | 0.179 | 0.018 | 4.16E-06 | 0.996 | FALSE | 21 |
|  | rs7778345 | G | A | 0.730 | 0.007 | 0.163 | 0.017 | 7.05E-06 | 0.690 | FALSE | 20 |
| genus.Erysipelatoclostridium(id.11381) | rs10513202 | G | A | 1.427 | 0.018 | 0.321 | 0.023 | 8.58E-06 | 0.426 | FALSE | 20 |
|  | rs11090513 | T | G | 0.798 | 0.002 | 0.175 | 0.017 | 5.00E-06 | 0.912 | FALSE | 21 |
|  | rs11230859 | A | G | -0.725 | -0.001 | 0.158 | 0.015 | 4.18E-06 | 0.921 | FALSE | 21 |
|  | rs12669698 | C | T | 1.127 | -0.052 | 0.255 | 0.023 | 9.73E-06 | 0.021 | FALSE | 20 |
|  | rs17145573 | A | G | 1.708 | -0.024 | 0.365 | 0.023 | 2.87E-06 | 0.298 | FALSE | 22 |
|  | rs389558 | T | C | -0.783 | 0.019 | 0.160 | 0.015 | 9.74E-07 | 0.199 | FALSE | 24 |
|  | rs491806 | C | A | -0.948 | 0.004 | 0.211 | 0.018 | 6.92E-06 | 0.807 | FALSE | 20 |
|  | rs529324 | A | G | -0.826 | 0.007 | 0.179 | 0.016 | 4.16E-06 | 0.669 | FALSE | 21 |
|  | rs7778345 | G | A | 0.730 | -0.011 | 0.163 | 0.015 | 7.05E-06 | 0.460 | FALSE | 20 |
| genus.ErysipelotrichaceaeUCG003(id.11384) | rs10513202 | G | A | 1.427 | -0.046 | 0.321 | 0.019 | 8.58E-06 | 0.014 | FALSE | 20 |
|  | rs11090513 | T | G | 0.798 | 0.020 | 0.175 | 0.014 | 5.00E-06 | 0.147 | FALSE | 21 |
|  | rs11230859 | A | G | -0.725 | -0.006 | 0.158 | 0.012 | 4.18E-06 | 0.617 | FALSE | 21 |
|  | rs12669698 | C | T | 1.127 | 0.017 | 0.255 | 0.018 | 9.73E-06 | 0.359 | FALSE | 20 |
|  | rs17145573 | A | G | 1.708 | -0.019 | 0.365 | 0.019 | 2.87E-06 | 0.295 | FALSE | 22 |
|  | rs389558 | T | C | -0.783 | -0.019 | 0.160 | 0.012 | 9.74E-07 | 0.102 | FALSE | 24 |
|  | rs491806 | C | A | -0.948 | -0.001 | 0.211 | 0.014 | 6.92E-06 | 0.947 | FALSE | 20 |
|  | rs529324 | A | G | -0.826 | -0.017 | 0.179 | 0.013 | 4.16E-06 | 0.175 | FALSE | 21 |
|  | rs7778345 | G | A | 0.730 | -0.003 | 0.163 | 0.012 | 7.05E-06 | 0.773 | FALSE | 20 |
| genus.Escherichia.Shigella(id.3504) | rs10513202 | G | A | 1.427 | 0.021 | 0.321 | 0.021 | 8.58E-06 | 0.322 | FALSE | 20 |
|  | rs11090513 | T | G | 0.798 | 0.006 | 0.175 | 0.015 | 5.00E-06 | 0.679 | FALSE | 21 |
|  | rs11230859 | A | G | -0.725 | 0.026 | 0.158 | 0.014 | 4.18E-06 | 0.054 | FALSE | 21 |
|  | rs12669698 | C | T | 1.127 | -0.024 | 0.255 | 0.020 | 9.73E-06 | 0.240 | FALSE | 20 |
|  | rs17145573 | A | G | 1.708 | 0.027 | 0.365 | 0.021 | 2.87E-06 | 0.183 | FALSE | 22 |
|  | rs389558 | T | C | -0.783 | 0.009 | 0.160 | 0.013 | 9.74E-07 | 0.485 | FALSE | 24 |
|  | rs491806 | C | A | -0.948 | -0.012 | 0.211 | 0.016 | 6.92E-06 | 0.466 | FALSE | 20 |
|  | rs529324 | A | G | -0.826 | 0.020 | 0.179 | 0.014 | 4.16E-06 | 0.144 | FALSE | 21 |
|  | rs7778345 | G | A | 0.730 | 0.006 | 0.163 | 0.013 | 7.05E-06 | 0.631 | FALSE | 20 |
| genus.Faecalibacterium(id.2057) | rs10513202 | G | A | 1.427 | -0.018 | 0.321 | 0.017 | 8.58E-06 | 0.285 | FALSE | 20 |
|  | rs11090513 | T | G | 0.798 | 0.009 | 0.175 | 0.012 | 5.00E-06 | 0.469 | FALSE | 21 |
|  | rs11230859 | A | G | -0.725 | -0.012 | 0.158 | 0.011 | 4.18E-06 | 0.281 | FALSE | 21 |
|  | rs12669698 | C | T | 1.127 | -0.001 | 0.255 | 0.016 | 9.73E-06 | 0.957 | FALSE | 20 |
|  | rs17145573 | A | G | 1.708 | 0.006 | 0.365 | 0.017 | 2.87E-06 | 0.731 | FALSE | 22 |
|  | rs389558 | T | C | -0.783 | 0.004 | 0.160 | 0.011 | 9.74E-07 | 0.728 | FALSE | 24 |
|  | rs491806 | C | A | -0.948 | 0.004 | 0.211 | 0.013 | 6.92E-06 | 0.736 | FALSE | 20 |
|  | rs529324 | A | G | -0.826 | -0.013 | 0.179 | 0.011 | 4.16E-06 | 0.243 | FALSE | 21 |
|  | rs7778345 | G | A | 0.730 | -0.005 | 0.163 | 0.011 | 7.05E-06 | 0.624 | FALSE | 20 |
| genus.FamilyXIIIAD3011group(id.11293) | rs10513202 | G | A | 1.427 | -0.019 | 0.321 | 0.019 | 8.58E-06 | 0.310 | FALSE | 20 |
|  | rs11090513 | T | G | 0.798 | 0.011 | 0.175 | 0.014 | 5.00E-06 | 0.422 | FALSE | 21 |
|  | rs11230859 | A | G | -0.725 | 0.001 | 0.158 | 0.012 | 4.18E-06 | 0.952 | FALSE | 21 |
|  | rs12669698 | C | T | 1.127 | -0.009 | 0.255 | 0.019 | 9.73E-06 | 0.623 | FALSE | 20 |
|  | rs17145573 | A | G | 1.708 | -0.019 | 0.365 | 0.019 | 2.87E-06 | 0.319 | FALSE | 22 |
|  | rs389558 | T | C | -0.783 | -0.005 | 0.160 | 0.012 | 9.74E-07 | 0.690 | FALSE | 24 |
|  | rs491806 | C | A | -0.948 | 0.022 | 0.211 | 0.015 | 6.92E-06 | 0.140 | FALSE | 20 |
|  | rs529324 | A | G | -0.826 | 0.022 | 0.179 | 0.013 | 4.16E-06 | 0.081 | FALSE | 21 |
|  | rs7778345 | G | A | 0.730 | -0.002 | 0.163 | 0.012 | 7.05E-06 | 0.858 | FALSE | 20 |
| genus.FamilyXIIIUCG001(id.11294) | rs10513202 | G | A | 1.427 | 0.002 | 0.321 | 0.020 | 8.58E-06 | 0.928 | FALSE | 20 |
|  | rs11090513 | T | G | 0.798 | 0.007 | 0.175 | 0.015 | 5.00E-06 | 0.620 | FALSE | 21 |
|  | rs11230859 | A | G | -0.725 | -0.002 | 0.158 | 0.013 | 4.18E-06 | 0.866 | FALSE | 21 |
|  | rs12669698 | C | T | 1.127 | -0.008 | 0.255 | 0.020 | 9.73E-06 | 0.706 | FALSE | 20 |
|  | rs17145573 | A | G | 1.708 | 0.019 | 0.365 | 0.020 | 2.87E-06 | 0.344 | FALSE | 22 |
|  | rs389558 | T | C | -0.783 | 0.006 | 0.160 | 0.013 | 9.74E-07 | 0.638 | FALSE | 24 |
|  | rs491806 | C | A | -0.948 | 0.006 | 0.211 | 0.015 | 6.92E-06 | 0.687 | FALSE | 20 |
|  | rs529324 | A | G | -0.826 | 0.008 | 0.179 | 0.014 | 4.16E-06 | 0.547 | FALSE | 21 |
|  | rs7778345 | G | A | 0.730 | 0.006 | 0.163 | 0.013 | 7.05E-06 | 0.636 | FALSE | 20 |
| genus.Flavonifractor(id.2059) | rs10513202 | G | A | 1.427 | 0.029 | 0.321 | 0.022 | 8.58E-06 | 0.190 | FALSE | 20 |
|  | rs11090513 | T | G | 0.798 | -0.021 | 0.175 | 0.016 | 5.00E-06 | 0.195 | FALSE | 21 |
|  | rs11230859 | A | G | -0.725 | 0.002 | 0.158 | 0.014 | 4.18E-06 | 0.902 | FALSE | 21 |
|  | rs12669698 | C | T | 1.127 | -0.012 | 0.255 | 0.021 | 9.73E-06 | 0.584 | FALSE | 20 |
|  | rs17145573 | A | G | 1.708 | 0.009 | 0.365 | 0.022 | 2.87E-06 | 0.685 | FALSE | 22 |
|  | rs389558 | T | C | -0.783 | 0.013 | 0.160 | 0.014 | 9.74E-07 | 0.358 | FALSE | 24 |
|  | rs491806 | C | A | -0.948 | -0.013 | 0.211 | 0.017 | 6.92E-06 | 0.456 | FALSE | 20 |
|  | rs529324 | A | G | -0.826 | -0.002 | 0.179 | 0.015 | 4.16E-06 | 0.880 | FALSE | 21 |
|  | rs7778345 | G | A | 0.730 | -0.006 | 0.163 | 0.014 | 7.05E-06 | 0.676 | FALSE | 20 |
| genus.Fusicatenibacter(id.11305) | rs10513202 | G | A | 1.427 | -0.004 | 0.321 | 0.017 | 8.58E-06 | 0.816 | FALSE | 20 |
|  | rs11090513 | T | G | 0.798 | 0.003 | 0.175 | 0.013 | 5.00E-06 | 0.788 | FALSE | 21 |
|  | rs11230859 | A | G | -0.725 | -0.008 | 0.158 | 0.011 | 4.18E-06 | 0.461 | FALSE | 21 |
|  | rs12669698 | C | T | 1.127 | 0.014 | 0.255 | 0.017 | 9.73E-06 | 0.401 | FALSE | 20 |
|  | rs17145573 | A | G | 1.708 | 0.000 | 0.365 | 0.017 | 2.87E-06 | 0.982 | FALSE | 22 |
|  | rs389558 | T | C | -0.783 | 0.005 | 0.160 | 0.011 | 9.74E-07 | 0.664 | FALSE | 24 |
|  | rs491806 | C | A | -0.948 | 0.008 | 0.211 | 0.013 | 6.92E-06 | 0.568 | FALSE | 20 |
|  | rs529324 | A | G | -0.826 | 0.014 | 0.179 | 0.012 | 4.16E-06 | 0.224 | FALSE | 21 |
|  | rs7778345 | G | A | 0.730 | -0.003 | 0.163 | 0.011 | 7.05E-06 | 0.764 | FALSE | 20 |
| genus.Gordonibacter(id.821) | rs10513202 | G | A | 1.427 | -0.032 | 0.321 | 0.037 | 8.58E-06 | 0.394 | FALSE | 20 |
|  | rs11090513 | T | G | 0.798 | 0.011 | 0.175 | 0.027 | 5.00E-06 | 0.673 | FALSE | 21 |
|  | rs11230859 | A | G | -0.725 | 0.051 | 0.158 | 0.024 | 4.18E-06 | 0.035 | FALSE | 21 |
|  | rs12669698 | C | T | 1.127 | 0.016 | 0.255 | 0.037 | 9.73E-06 | 0.664 | FALSE | 20 |
|  | rs17145573 | A | G | 1.708 | 0.002 | 0.365 | 0.037 | 2.87E-06 | 0.955 | FALSE | 22 |
|  | rs389558 | T | C | -0.783 | -0.007 | 0.160 | 0.023 | 9.74E-07 | 0.768 | FALSE | 24 |
|  | rs491806 | C | A | -0.948 | 0.030 | 0.211 | 0.029 | 6.92E-06 | 0.287 | FALSE | 20 |
|  | rs529324 | A | G | -0.826 | -0.013 | 0.179 | 0.025 | 4.16E-06 | 0.606 | FALSE | 21 |
|  | rs7778345 | G | A | 0.730 | -0.030 | 0.163 | 0.024 | 7.05E-06 | 0.197 | FALSE | 20 |
| genus.Haemophilus(id.3698) | rs10513202 | G | A | 1.427 | 0.012 | 0.321 | 0.024 | 8.58E-06 | 0.606 | FALSE | 20 |
|  | rs11090513 | T | G | 0.798 | -0.002 | 0.175 | 0.018 | 5.00E-06 | 0.914 | FALSE | 21 |
|  | rs11230859 | A | G | -0.725 | -0.002 | 0.158 | 0.015 | 4.18E-06 | 0.874 | FALSE | 21 |
|  | rs12669698 | C | T | 1.127 | 0.023 | 0.255 | 0.023 | 9.73E-06 | 0.308 | FALSE | 20 |
|  | rs17145573 | A | G | 1.708 | -0.004 | 0.365 | 0.024 | 2.87E-06 | 0.852 | FALSE | 22 |
|  | rs389558 | T | C | -0.783 | -0.030 | 0.160 | 0.015 | 9.74E-07 | 0.050 | FALSE | 24 |
|  | rs491806 | C | A | -0.948 | 0.023 | 0.211 | 0.018 | 6.92E-06 | 0.217 | FALSE | 20 |
|  | rs529324 | A | G | -0.826 | 0.017 | 0.179 | 0.016 | 4.16E-06 | 0.288 | FALSE | 21 |
|  | rs7778345 | G | A | 0.730 | -0.003 | 0.163 | 0.015 | 7.05E-06 | 0.821 | FALSE | 20 |
| genus.Holdemanella(id.11393) | rs10513202 | G | A | 1.427 | -0.007 | 0.321 | 0.026 | 8.58E-06 | 0.778 | FALSE | 20 |
|  | rs11090513 | T | G | 0.798 | -0.019 | 0.175 | 0.019 | 5.00E-06 | 0.307 | FALSE | 21 |
|  | rs11230859 | A | G | -0.725 | -0.011 | 0.158 | 0.017 | 4.18E-06 | 0.502 | FALSE | 21 |
|  | rs12669698 | C | T | 1.127 | 0.015 | 0.255 | 0.025 | 9.73E-06 | 0.551 | FALSE | 20 |
|  | rs17145573 | A | G | 1.708 | 0.004 | 0.365 | 0.026 | 2.87E-06 | 0.892 | FALSE | 22 |
|  | rs389558 | T | C | -0.783 | -0.019 | 0.160 | 0.017 | 9.74E-07 | 0.240 | FALSE | 24 |
|  | rs491806 | C | A | -0.948 | -0.023 | 0.211 | 0.020 | 6.92E-06 | 0.242 | FALSE | 20 |
|  | rs529324 | A | G | -0.826 | 0.005 | 0.179 | 0.017 | 4.16E-06 | 0.765 | FALSE | 21 |
|  | rs7778345 | G | A | 0.730 | -0.014 | 0.163 | 0.017 | 7.05E-06 | 0.390 | FALSE | 20 |
| genus.Holdemania(id.2157) | rs10513202 | G | A | 1.427 | 0.007 | 0.321 | 0.024 | 8.58E-06 | 0.757 | FALSE | 20 |
|  | rs11090513 | T | G | 0.798 | -0.001 | 0.175 | 0.017 | 5.00E-06 | 0.956 | FALSE | 21 |
|  | rs11230859 | A | G | -0.725 | -0.001 | 0.158 | 0.016 | 4.18E-06 | 0.935 | FALSE | 21 |
|  | rs12669698 | C | T | 1.127 | 0.002 | 0.255 | 0.024 | 9.73E-06 | 0.929 | FALSE | 20 |
|  | rs17145573 | A | G | 1.708 | 0.005 | 0.365 | 0.024 | 2.87E-06 | 0.821 | FALSE | 22 |
|  | rs389558 | T | C | -0.783 | 0.024 | 0.160 | 0.015 | 9.74E-07 | 0.109 | FALSE | 24 |
|  | rs491806 | C | A | -0.948 | 0.015 | 0.211 | 0.018 | 6.92E-06 | 0.415 | FALSE | 20 |
|  | rs529324 | A | G | -0.826 | 0.011 | 0.179 | 0.016 | 4.16E-06 | 0.509 | FALSE | 21 |
|  | rs7778345 | G | A | 0.730 | 0.015 | 0.163 | 0.015 | 7.05E-06 | 0.326 | FALSE | 20 |
| genus.Howardella(id.2000) | rs10513202 | G | A | 1.427 | 0.026 | 0.321 | 0.037 | 8.58E-06 | 0.479 | FALSE | 20 |
|  | rs11090513 | T | G | 0.798 | 0.021 | 0.175 | 0.027 | 5.00E-06 | 0.441 | FALSE | 21 |
|  | rs11230859 | A | G | -0.725 | -0.029 | 0.158 | 0.024 | 4.18E-06 | 0.223 | FALSE | 21 |
|  | rs12669698 | C | T | 1.127 | -0.003 | 0.255 | 0.036 | 9.73E-06 | 0.934 | FALSE | 20 |
|  | rs17145573 | A | G | 1.708 | 0.000 | 0.365 | 0.037 | 2.87E-06 | 0.999 | FALSE | 22 |
|  | rs389558 | T | C | -0.783 | -0.014 | 0.160 | 0.024 | 9.74E-07 | 0.566 | FALSE | 24 |
|  | rs491806 | C | A | -0.948 | -0.011 | 0.211 | 0.028 | 6.92E-06 | 0.692 | FALSE | 20 |
|  | rs529324 | A | G | -0.826 | 0.005 | 0.179 | 0.025 | 4.16E-06 | 0.845 | FALSE | 21 |
|  | rs7778345 | G | A | 0.730 | -0.042 | 0.163 | 0.024 | 7.05E-06 | 0.078 | FALSE | 20 |
| genus.Hungatella(id.11306) | rs10513202 | G | A | 1.427 | -0.017 | 0.321 | 0.035 | 8.58E-06 | 0.621 | FALSE | 20 |
|  | rs11090513 | T | G | 0.798 | -0.014 | 0.175 | 0.026 | 5.00E-06 | 0.594 | FALSE | 21 |
|  | rs11230859 | A | G | -0.725 | 0.013 | 0.158 | 0.023 | 4.18E-06 | 0.560 | FALSE | 21 |
|  | rs12669698 | C | T | 1.127 | 0.018 | 0.255 | 0.034 | 9.73E-06 | 0.591 | FALSE | 20 |
|  | rs17145573 | A | G | 1.708 | 0.000 | 0.365 | 0.035 | 2.87E-06 | 0.998 | FALSE | 22 |
|  | rs389558 | T | C | -0.783 | -0.002 | 0.160 | 0.023 | 9.74E-07 | 0.929 | FALSE | 24 |
|  | rs491806 | C | A | -0.948 | 0.015 | 0.211 | 0.027 | 6.92E-06 | 0.586 | FALSE | 20 |
|  | rs529324 | A | G | -0.826 | -0.029 | 0.179 | 0.024 | 4.16E-06 | 0.210 | FALSE | 21 |
|  | rs7778345 | G | A | 0.730 | 0.008 | 0.163 | 0.022 | 7.05E-06 | 0.730 | FALSE | 20 |
| genus.Intestinibacter(id.11345) | rs10513202 | G | A | 1.427 | 0.012 | 0.321 | 0.021 | 8.58E-06 | 0.569 | FALSE | 20 |
|  | rs11090513 | T | G | 0.798 | -0.010 | 0.175 | 0.015 | 5.00E-06 | 0.505 | FALSE | 21 |
|  | rs11230859 | A | G | -0.725 | 0.010 | 0.158 | 0.013 | 4.18E-06 | 0.446 | FALSE | 21 |
|  | rs12669698 | C | T | 1.127 | 0.023 | 0.255 | 0.020 | 9.73E-06 | 0.262 | FALSE | 20 |
|  | rs17145573 | A | G | 1.708 | 0.010 | 0.365 | 0.021 | 2.87E-06 | 0.625 | FALSE | 22 |
|  | rs389558 | T | C | -0.783 | 0.007 | 0.160 | 0.013 | 9.74E-07 | 0.582 | FALSE | 24 |
|  | rs491806 | C | A | -0.948 | -0.007 | 0.211 | 0.016 | 6.92E-06 | 0.635 | FALSE | 20 |
|  | rs529324 | A | G | -0.826 | -0.003 | 0.179 | 0.014 | 4.16E-06 | 0.836 | FALSE | 21 |
|  | rs7778345 | G | A | 0.730 | 0.029 | 0.163 | 0.013 | 7.05E-06 | 0.028 | FALSE | 20 |
| genus.Intestinimonas(id.2062) | rs10513202 | G | A | 1.427 | -0.036 | 0.321 | 0.021 | 8.58E-06 | 0.083 | FALSE | 20 |
|  | rs11090513 | T | G | 0.798 | 0.008 | 0.175 | 0.015 | 5.00E-06 | 0.612 | FALSE | 21 |
|  | rs11230859 | A | G | -0.725 | 0.002 | 0.158 | 0.013 | 4.18E-06 | 0.864 | FALSE | 21 |
|  | rs12669698 | C | T | 1.127 | 0.002 | 0.255 | 0.020 | 9.73E-06 | 0.924 | FALSE | 20 |
|  | rs17145573 | A | G | 1.708 | -0.001 | 0.365 | 0.021 | 2.87E-06 | 0.945 | FALSE | 22 |
|  | rs389558 | T | C | -0.783 | 0.013 | 0.160 | 0.013 | 9.74E-07 | 0.336 | FALSE | 24 |
|  | rs491806 | C | A | -0.948 | 0.004 | 0.211 | 0.016 | 6.92E-06 | 0.796 | FALSE | 20 |
|  | rs529324 | A | G | -0.826 | -0.019 | 0.179 | 0.014 | 4.16E-06 | 0.172 | FALSE | 21 |
|  | rs7778345 | G | A | 0.730 | -0.007 | 0.163 | 0.013 | 7.05E-06 | 0.579 | FALSE | 20 |
| genus.Lachnoclostridium(id.11308) | rs10513202 | G | A | 1.427 | -0.007 | 0.321 | 0.017 | 8.58E-06 | 0.700 | FALSE | 20 |
|  | rs11090513 | T | G | 0.798 | -0.021 | 0.175 | 0.012 | 5.00E-06 | 0.099 | FALSE | 21 |
|  | rs11230859 | A | G | -0.725 | 0.001 | 0.158 | 0.011 | 4.18E-06 | 0.921 | FALSE | 21 |
|  | rs12669698 | C | T | 1.127 | 0.022 | 0.255 | 0.016 | 9.73E-06 | 0.176 | FALSE | 20 |
|  | rs17145573 | A | G | 1.708 | 0.004 | 0.365 | 0.017 | 2.87E-06 | 0.835 | FALSE | 22 |
|  | rs389558 | T | C | -0.783 | 0.010 | 0.160 | 0.011 | 9.74E-07 | 0.378 | FALSE | 24 |
|  | rs491806 | C | A | -0.948 | -0.007 | 0.211 | 0.013 | 6.92E-06 | 0.598 | FALSE | 20 |
|  | rs529324 | A | G | -0.826 | -0.001 | 0.179 | 0.011 | 4.16E-06 | 0.931 | FALSE | 21 |
|  | rs7778345 | G | A | 0.730 | 0.003 | 0.163 | 0.011 | 7.05E-06 | 0.803 | FALSE | 20 |
| genus.Lachnospira(id.2004) | rs10513202 | G | A | 1.427 | 0.025 | 0.321 | 0.018 | 8.58E-06 | 0.160 | FALSE | 20 |
|  | rs11090513 | T | G | 0.798 | -0.004 | 0.175 | 0.013 | 5.00E-06 | 0.735 | FALSE | 21 |
|  | rs11230859 | A | G | -0.725 | 0.001 | 0.158 | 0.012 | 4.18E-06 | 0.909 | FALSE | 21 |
|  | rs12669698 | C | T | 1.127 | 0.014 | 0.255 | 0.017 | 9.73E-06 | 0.398 | FALSE | 20 |
|  | rs17145573 | A | G | 1.708 | -0.015 | 0.365 | 0.018 | 2.87E-06 | 0.382 | FALSE | 22 |
|  | rs389558 | T | C | -0.783 | -0.003 | 0.160 | 0.011 | 9.74E-07 | 0.761 | FALSE | 24 |
|  | rs491806 | C | A | -0.948 | 0.004 | 0.211 | 0.014 | 6.92E-06 | 0.751 | FALSE | 20 |
|  | rs529324 | A | G | -0.826 | 0.002 | 0.179 | 0.012 | 4.16E-06 | 0.867 | FALSE | 21 |
|  | rs7778345 | G | A | 0.730 | -0.006 | 0.163 | 0.011 | 7.05E-06 | 0.580 | FALSE | 20 |
| genus.LachnospiraceaeFCS020group(id.11314) | rs10513202 | G | A | 1.427 | -0.020 | 0.321 | 0.020 | 8.58E-06 | 0.309 | FALSE | 20 |
|  | rs11090513 | T | G | 0.798 | -0.001 | 0.175 | 0.014 | 5.00E-06 | 0.966 | FALSE | 21 |
|  | rs11230859 | A | G | -0.725 | -0.006 | 0.158 | 0.013 | 4.18E-06 | 0.618 | FALSE | 21 |
|  | rs12669698 | C | T | 1.127 | -0.003 | 0.255 | 0.019 | 9.73E-06 | 0.892 | FALSE | 20 |
|  | rs17145573 | A | G | 1.708 | 0.012 | 0.365 | 0.019 | 2.87E-06 | 0.522 | FALSE | 22 |
|  | rs389558 | T | C | -0.783 | 0.015 | 0.160 | 0.012 | 9.74E-07 | 0.236 | FALSE | 24 |
|  | rs491806 | C | A | -0.948 | -0.015 | 0.211 | 0.015 | 6.92E-06 | 0.327 | FALSE | 20 |
|  | rs529324 | A | G | -0.826 | -0.012 | 0.179 | 0.013 | 4.16E-06 | 0.372 | FALSE | 21 |
|  | rs7778345 | G | A | 0.730 | 0.009 | 0.163 | 0.012 | 7.05E-06 | 0.458 | FALSE | 20 |
| genus.LachnospiraceaeNC2004group(id.11316) | rs10513202 | G | A | 1.427 | -0.039 | 0.321 | 0.029 | 8.58E-06 | 0.172 | FALSE | 20 |
|  | rs11090513 | T | G | 0.798 | 0.012 | 0.175 | 0.021 | 5.00E-06 | 0.564 | FALSE | 21 |
|  | rs11230859 | A | G | -0.725 | 0.014 | 0.158 | 0.019 | 4.18E-06 | 0.447 | FALSE | 21 |
|  | rs12669698 | C | T | 1.127 | 0.006 | 0.255 | 0.027 | 9.73E-06 | 0.814 | FALSE | 20 |
|  | rs17145573 | A | G | 1.708 | -0.049 | 0.365 | 0.028 | 2.87E-06 | 0.081 | FALSE | 22 |
|  | rs389558 | T | C | -0.783 | -0.017 | 0.160 | 0.018 | 9.74E-07 | 0.351 | FALSE | 24 |
|  | rs491806 | C | A | -0.948 | 0.020 | 0.211 | 0.022 | 6.92E-06 | 0.380 | FALSE | 20 |
|  | rs529324 | A | G | -0.826 | -0.014 | 0.179 | 0.019 | 4.16E-06 | 0.468 | FALSE | 21 |
|  | rs7778345 | G | A | 0.730 | -0.012 | 0.163 | 0.019 | 7.05E-06 | 0.527 | FALSE | 20 |
| genus.LachnospiraceaeND3007group(id.11317) | rs10513202 | G | A | 1.427 | -0.014 | 0.321 | 0.018 | 8.58E-06 | 0.435 | FALSE | 20 |
|  | rs11090513 | T | G | 0.798 | -0.001 | 0.175 | 0.013 | 5.00E-06 | 0.968 | FALSE | 21 |
|  | rs11230859 | A | G | -0.725 | 0.004 | 0.158 | 0.012 | 4.18E-06 | 0.710 | FALSE | 21 |
|  | rs12669698 | C | T | 1.127 | 0.027 | 0.255 | 0.018 | 9.73E-06 | 0.133 | FALSE | 20 |
|  | rs17145573 | A | G | 1.708 | 0.022 | 0.365 | 0.018 | 2.87E-06 | 0.233 | FALSE | 22 |
|  | rs389558 | T | C | -0.783 | -0.013 | 0.160 | 0.012 | 9.74E-07 | 0.261 | FALSE | 24 |
|  | rs491806 | C | A | -0.948 | 0.015 | 0.211 | 0.014 | 6.92E-06 | 0.275 | FALSE | 20 |
|  | rs529324 | A | G | -0.826 | -0.014 | 0.179 | 0.012 | 4.16E-06 | 0.258 | FALSE | 21 |
|  | rs7778345 | G | A | 0.730 | -0.025 | 0.163 | 0.012 | 7.05E-06 | 0.037 | FALSE | 20 |
| genus.LachnospiraceaeNK4A136group(id.11319) | rs10513202 | G | A | 1.427 | 0.005 | 0.321 | 0.017 | 8.58E-06 | 0.773 | FALSE | 20 |
|  | rs11090513 | T | G | 0.798 | 0.025 | 0.175 | 0.013 | 5.00E-06 | 0.050 | FALSE | 21 |
|  | rs11230859 | A | G | -0.725 | 0.004 | 0.158 | 0.011 | 4.18E-06 | 0.714 | FALSE | 21 |
|  | rs12669698 | C | T | 1.127 | 0.014 | 0.255 | 0.017 | 9.73E-06 | 0.397 | FALSE | 20 |
|  | rs17145573 | A | G | 1.708 | -0.020 | 0.365 | 0.017 | 2.87E-06 | 0.249 | FALSE | 22 |
|  | rs389558 | T | C | -0.783 | -0.013 | 0.160 | 0.011 | 9.74E-07 | 0.254 | FALSE | 24 |
|  | rs491806 | C | A | -0.948 | -0.010 | 0.211 | 0.013 | 6.92E-06 | 0.441 | FALSE | 20 |
|  | rs529324 | A | G | -0.826 | 0.010 | 0.179 | 0.012 | 4.16E-06 | 0.369 | FALSE | 21 |
|  | rs7778345 | G | A | 0.730 | -0.002 | 0.163 | 0.011 | 7.05E-06 | 0.881 | FALSE | 20 |
| genus.LachnospiraceaeUCG001(id.11321) | rs10513202 | G | A | 1.427 | 0.028 | 0.321 | 0.023 | 8.58E-06 | 0.220 | FALSE | 20 |
|  | rs11090513 | T | G | 0.798 | -0.010 | 0.175 | 0.016 | 5.00E-06 | 0.531 | FALSE | 21 |
|  | rs11230859 | A | G | -0.725 | -0.001 | 0.158 | 0.015 | 4.18E-06 | 0.950 | FALSE | 21 |
|  | rs12669698 | C | T | 1.127 | -0.001 | 0.255 | 0.022 | 9.73E-06 | 0.976 | FALSE | 20 |
|  | rs17145573 | A | G | 1.708 | 0.015 | 0.365 | 0.023 | 2.87E-06 | 0.508 | FALSE | 22 |
|  | rs389558 | T | C | -0.783 | -0.019 | 0.160 | 0.014 | 9.74E-07 | 0.176 | FALSE | 24 |
|  | rs491806 | C | A | -0.948 | 0.006 | 0.211 | 0.017 | 6.92E-06 | 0.734 | FALSE | 20 |
|  | rs529324 | A | G | -0.826 | -0.009 | 0.179 | 0.015 | 4.16E-06 | 0.547 | FALSE | 21 |
|  | rs7778345 | G | A | 0.730 | -0.011 | 0.163 | 0.014 | 7.05E-06 | 0.438 | FALSE | 20 |
| genus.LachnospiraceaeUCG004(id.11324) | rs10513202 | G | A | 1.427 | -0.006 | 0.321 | 0.019 | 8.58E-06 | 0.765 | FALSE | 20 |
|  | rs11090513 | T | G | 0.798 | 0.002 | 0.175 | 0.014 | 5.00E-06 | 0.882 | FALSE | 21 |
|  | rs11230859 | A | G | -0.725 | 0.017 | 0.158 | 0.012 | 4.18E-06 | 0.166 | FALSE | 21 |
|  | rs12669698 | C | T | 1.127 | -0.025 | 0.255 | 0.018 | 9.73E-06 | 0.162 | FALSE | 20 |
|  | rs17145573 | A | G | 1.708 | -0.017 | 0.365 | 0.019 | 2.87E-06 | 0.379 | FALSE | 22 |
|  | rs389558 | T | C | -0.783 | 0.000 | 0.160 | 0.012 | 9.74E-07 | 0.998 | FALSE | 24 |
|  | rs491806 | C | A | -0.948 | 0.001 | 0.211 | 0.014 | 6.92E-06 | 0.968 | FALSE | 20 |
|  | rs529324 | A | G | -0.826 | -0.017 | 0.179 | 0.013 | 4.16E-06 | 0.180 | FALSE | 21 |
|  | rs7778345 | G | A | 0.730 | -0.002 | 0.163 | 0.012 | 7.05E-06 | 0.866 | FALSE | 20 |
| genus.LachnospiraceaeUCG008(id.11328) | rs10513202 | G | A | 1.427 | 0.004 | 0.321 | 0.028 | 8.58E-06 | 0.881 | FALSE | 20 |
|  | rs11090513 | T | G | 0.798 | 0.025 | 0.175 | 0.020 | 5.00E-06 | 0.206 | FALSE | 21 |
|  | rs11230859 | A | G | -0.725 | -0.025 | 0.158 | 0.018 | 4.18E-06 | 0.149 | FALSE | 21 |
|  | rs12669698 | C | T | 1.127 | 0.007 | 0.255 | 0.027 | 9.73E-06 | 0.784 | FALSE | 20 |
|  | rs17145573 | A | G | 1.708 | -0.031 | 0.365 | 0.027 | 2.87E-06 | 0.252 | FALSE | 22 |
|  | rs389558 | T | C | -0.783 | 0.013 | 0.160 | 0.017 | 9.74E-07 | 0.450 | FALSE | 24 |
|  | rs491806 | C | A | -0.948 | 0.027 | 0.211 | 0.021 | 6.92E-06 | 0.205 | FALSE | 20 |
|  | rs529324 | A | G | -0.826 | 0.012 | 0.179 | 0.018 | 4.16E-06 | 0.501 | FALSE | 21 |
|  | rs7778345 | G | A | 0.730 | -0.016 | 0.163 | 0.017 | 7.05E-06 | 0.355 | FALSE | 20 |
| genus.LachnospiraceaeUCG010(id.11330) | rs10513202 | G | A | 1.427 | -0.035 | 0.321 | 0.020 | 8.58E-06 | 0.079 | FALSE | 20 |
|  | rs11090513 | T | G | 0.798 | 0.009 | 0.175 | 0.015 | 5.00E-06 | 0.518 | FALSE | 21 |
|  | rs11230859 | A | G | -0.725 | -0.004 | 0.158 | 0.013 | 4.18E-06 | 0.737 | FALSE | 21 |
|  | rs12669698 | C | T | 1.127 | -0.014 | 0.255 | 0.019 | 9.73E-06 | 0.481 | FALSE | 20 |
|  | rs17145573 | A | G | 1.708 | -0.008 | 0.365 | 0.020 | 2.87E-06 | 0.671 | FALSE | 22 |
|  | rs389558 | T | C | -0.783 | -0.023 | 0.160 | 0.013 | 9.74E-07 | 0.074 | FALSE | 24 |
|  | rs491806 | C | A | -0.948 | 0.006 | 0.211 | 0.015 | 6.92E-06 | 0.692 | FALSE | 20 |
|  | rs529324 | A | G | -0.826 | -0.003 | 0.179 | 0.013 | 4.16E-06 | 0.808 | FALSE | 21 |
|  | rs7778345 | G | A | 0.730 | -0.014 | 0.163 | 0.013 | 7.05E-06 | 0.271 | FALSE | 20 |
| genus.Lactobacillus(id.1837) | rs10513202 | G | A | 1.427 | 0.039 | 0.321 | 0.028 | 8.58E-06 | 0.161 | FALSE | 20 |
|  | rs11090513 | T | G | 0.798 | -0.008 | 0.175 | 0.020 | 5.00E-06 | 0.684 | FALSE | 21 |
|  | rs11230859 | A | G | -0.725 | -0.010 | 0.158 | 0.018 | 4.18E-06 | 0.592 | FALSE | 21 |
|  | rs12669698 | C | T | 1.127 | 0.005 | 0.255 | 0.027 | 9.73E-06 | 0.847 | FALSE | 20 |
|  | rs17145573 | A | G | 1.708 | 0.027 | 0.365 | 0.027 | 2.87E-06 | 0.314 | FALSE | 22 |
|  | rs389558 | T | C | -0.783 | 0.010 | 0.160 | 0.017 | 9.74E-07 | 0.569 | FALSE | 24 |
|  | rs491806 | C | A | -0.948 | -0.021 | 0.211 | 0.021 | 6.92E-06 | 0.320 | FALSE | 20 |
|  | rs529324 | A | G | -0.826 | 0.005 | 0.179 | 0.018 | 4.16E-06 | 0.794 | FALSE | 21 |
|  | rs7778345 | G | A | 0.730 | -0.003 | 0.163 | 0.018 | 7.05E-06 | 0.880 | FALSE | 20 |
| genus.Lactococcus(id.1851) | rs10513202 | G | A | 1.427 | -0.051 | 0.321 | 0.036 | 8.58E-06 | 0.159 | FALSE | 20 |
|  | rs11090513 | T | G | 0.798 | -0.008 | 0.175 | 0.027 | 5.00E-06 | 0.763 | FALSE | 21 |
|  | rs11230859 | A | G | -0.725 | 0.006 | 0.158 | 0.023 | 4.18E-06 | 0.783 | FALSE | 21 |
|  | rs12669698 | C | T | 1.127 | -0.004 | 0.255 | 0.035 | 9.73E-06 | 0.911 | FALSE | 20 |
|  | rs17145573 | A | G | 1.708 | -0.018 | 0.365 | 0.036 | 2.87E-06 | 0.628 | FALSE | 22 |
|  | rs389558 | T | C | -0.783 | 0.000 | 0.160 | 0.023 | 9.74E-07 | 0.985 | FALSE | 24 |
|  | rs491806 | C | A | -0.948 | 0.005 | 0.211 | 0.027 | 6.92E-06 | 0.859 | FALSE | 20 |
|  | rs529324 | A | G | -0.826 | 0.033 | 0.179 | 0.024 | 4.16E-06 | 0.168 | FALSE | 21 |
|  | rs7778345 | G | A | 0.730 | 0.008 | 0.163 | 0.023 | 7.05E-06 | 0.718 | FALSE | 20 |
| genus.Marvinbryantia(id.2005) | rs10513202 | G | A | 1.427 | -0.017 | 0.321 | 0.021 | 8.58E-06 | 0.412 | FALSE | 20 |
|  | rs11090513 | T | G | 0.798 | 0.030 | 0.175 | 0.015 | 5.00E-06 | 0.046 | FALSE | 21 |
|  | rs11230859 | A | G | -0.725 | -0.005 | 0.158 | 0.014 | 4.18E-06 | 0.713 | FALSE | 21 |
|  | rs12669698 | C | T | 1.127 | 0.034 | 0.255 | 0.021 | 9.73E-06 | 0.108 | FALSE | 20 |
|  | rs17145573 | A | G | 1.708 | -0.012 | 0.365 | 0.021 | 2.87E-06 | 0.579 | FALSE | 22 |
|  | rs389558 | T | C | -0.783 | 0.018 | 0.160 | 0.013 | 9.74E-07 | 0.160 | FALSE | 24 |
|  | rs491806 | C | A | -0.948 | -0.009 | 0.211 | 0.016 | 6.92E-06 | 0.581 | FALSE | 20 |
|  | rs529324 | A | G | -0.826 | 0.008 | 0.179 | 0.014 | 4.16E-06 | 0.584 | FALSE | 21 |
|  | rs7778345 | G | A | 0.730 | 0.005 | 0.163 | 0.013 | 7.05E-06 | 0.707 | FALSE | 20 |
| genus.Methanobrevibacter(id.123) | rs10513202 | G | A | 1.427 | 0.012 | 0.321 | 0.037 | 8.58E-06 | 0.754 | FALSE | 20 |
|  | rs11090513 | T | G | 0.798 | -0.005 | 0.175 | 0.029 | 5.00E-06 | 0.852 | FALSE | 21 |
|  | rs11230859 | A | G | -0.725 | 0.003 | 0.158 | 0.025 | 4.18E-06 | 0.913 | FALSE | 21 |
|  | rs12669698 | C | T | 1.127 | -0.021 | 0.255 | 0.038 | 9.73E-06 | 0.580 | FALSE | 20 |
|  | rs17145573 | A | G | 1.708 | -0.033 | 0.365 | 0.039 | 2.87E-06 | 0.393 | FALSE | 22 |
|  | rs389558 | T | C | -0.783 | 0.027 | 0.160 | 0.025 | 9.74E-07 | 0.271 | FALSE | 24 |
|  | rs491806 | C | A | -0.948 | 0.019 | 0.211 | 0.029 | 6.92E-06 | 0.519 | FALSE | 20 |
|  | rs529324 | A | G | -0.826 | 0.000 | 0.179 | 0.026 | 4.16E-06 | 0.993 | FALSE | 21 |
|  | rs7778345 | G | A | 0.730 | -0.033 | 0.163 | 0.024 | 7.05E-06 | 0.167 | FALSE | 20 |
| genus.Odoribacter(id.952) | rs10513202 | G | A | 1.427 | 0.028 | 0.321 | 0.019 | 8.58E-06 | 0.144 | FALSE | 20 |
|  | rs11090513 | T | G | 0.798 | 0.005 | 0.175 | 0.014 | 5.00E-06 | 0.738 | FALSE | 21 |
|  | rs11230859 | A | G | -0.725 | -0.003 | 0.158 | 0.012 | 4.18E-06 | 0.791 | FALSE | 21 |
|  | rs12669698 | C | T | 1.127 | 0.016 | 0.255 | 0.018 | 9.73E-06 | 0.382 | FALSE | 20 |
|  | rs17145573 | A | G | 1.708 | -0.029 | 0.365 | 0.019 | 2.87E-06 | 0.124 | FALSE | 22 |
|  | rs389558 | T | C | -0.783 | 0.004 | 0.160 | 0.012 | 9.74E-07 | 0.756 | FALSE | 24 |
|  | rs491806 | C | A | -0.948 | 0.002 | 0.211 | 0.014 | 6.92E-06 | 0.903 | FALSE | 20 |
|  | rs529324 | A | G | -0.826 | -0.005 | 0.179 | 0.013 | 4.16E-06 | 0.672 | FALSE | 21 |
|  | rs7778345 | G | A | 0.730 | -0.007 | 0.163 | 0.012 | 7.05E-06 | 0.561 | FALSE | 20 |
| genus.Olsenella(id.822) | rs10513202 | G | A | 1.427 | -0.024 | 0.321 | 0.038 | 8.58E-06 | 0.528 | FALSE | 20 |
|  | rs11090513 | T | G | 0.798 | -0.018 | 0.175 | 0.027 | 5.00E-06 | 0.490 | FALSE | 21 |
|  | rs11230859 | A | G | -0.725 | 0.012 | 0.158 | 0.024 | 4.18E-06 | 0.621 | FALSE | 21 |
|  | rs12669698 | C | T | 1.127 | -0.041 | 0.255 | 0.038 | 9.73E-06 | 0.276 | FALSE | 20 |
|  | rs17145573 | A | G | 1.708 | 0.021 | 0.365 | 0.038 | 2.87E-06 | 0.577 | FALSE | 22 |
|  | rs389558 | T | C | -0.783 | 0.012 | 0.160 | 0.023 | 9.74E-07 | 0.601 | FALSE | 24 |
|  | rs491806 | C | A | -0.948 | 0.005 | 0.211 | 0.028 | 6.92E-06 | 0.856 | FALSE | 20 |
|  | rs529324 | A | G | -0.826 | 0.018 | 0.179 | 0.025 | 4.16E-06 | 0.480 | FALSE | 21 |
|  | rs7778345 | G | A | 0.730 | 0.040 | 0.163 | 0.024 | 7.05E-06 | 0.089 | FALSE | 20 |
| genus.Oscillibacter(id.2063) | rs10513202 | G | A | 1.427 | 0.020 | 0.321 | 0.024 | 8.58E-06 | 0.402 | FALSE | 20 |
|  | rs11090513 | T | G | 0.798 | 0.001 | 0.175 | 0.018 | 5.00E-06 | 0.936 | FALSE | 21 |
|  | rs11230859 | A | G | -0.725 | 0.001 | 0.158 | 0.016 | 4.18E-06 | 0.965 | FALSE | 21 |
|  | rs12669698 | C | T | 1.127 | 0.045 | 0.255 | 0.024 | 9.73E-06 | 0.064 | FALSE | 20 |
|  | rs17145573 | A | G | 1.708 | 0.000 | 0.365 | 0.024 | 2.87E-06 | 0.990 | FALSE | 22 |
|  | rs389558 | T | C | -0.783 | 0.001 | 0.160 | 0.015 | 9.74E-07 | 0.948 | FALSE | 24 |
|  | rs491806 | C | A | -0.948 | -0.022 | 0.211 | 0.018 | 6.92E-06 | 0.232 | FALSE | 20 |
|  | rs529324 | A | G | -0.826 | 0.013 | 0.179 | 0.016 | 4.16E-06 | 0.413 | FALSE | 21 |
|  | rs7778345 | G | A | 0.730 | 0.015 | 0.163 | 0.015 | 7.05E-06 | 0.334 | FALSE | 20 |
| genus.Oscillospira(id.2064) | rs10513202 | G | A | 1.427 | -0.021 | 0.321 | 0.023 | 8.58E-06 | 0.360 | FALSE | 20 |
|  | rs11090513 | T | G | 0.798 | -0.022 | 0.175 | 0.017 | 5.00E-06 | 0.190 | FALSE | 21 |
|  | rs11230859 | A | G | -0.725 | -0.002 | 0.158 | 0.015 | 4.18E-06 | 0.893 | FALSE | 21 |
|  | rs12669698 | C | T | 1.127 | -0.013 | 0.255 | 0.022 | 9.73E-06 | 0.554 | FALSE | 20 |
|  | rs17145573 | A | G | 1.708 | -0.007 | 0.365 | 0.023 | 2.87E-06 | 0.742 | FALSE | 22 |
|  | rs389558 | T | C | -0.783 | -0.008 | 0.160 | 0.014 | 9.74E-07 | 0.561 | FALSE | 24 |
|  | rs491806 | C | A | -0.948 | -0.013 | 0.211 | 0.017 | 6.92E-06 | 0.444 | FALSE | 20 |
|  | rs529324 | A | G | -0.826 | 0.008 | 0.179 | 0.015 | 4.16E-06 | 0.577 | FALSE | 21 |
|  | rs7778345 | G | A | 0.730 | -0.010 | 0.163 | 0.014 | 7.05E-06 | 0.470 | FALSE | 20 |
| genus.Oxalobacter(id.2978) | rs10513202 | G | A | 1.427 | 0.026 | 0.321 | 0.034 | 8.58E-06 | 0.437 | FALSE | 20 |
|  | rs11090513 | T | G | 0.798 | 0.046 | 0.175 | 0.024 | 5.00E-06 | 0.060 | FALSE | 21 |
|  | rs11230859 | A | G | -0.725 | 0.003 | 0.158 | 0.022 | 4.18E-06 | 0.878 | FALSE | 21 |
|  | rs12669698 | C | T | 1.127 | 0.000 | 0.255 | 0.033 | 9.73E-06 | 0.997 | FALSE | 20 |
|  | rs17145573 | A | G | 1.708 | 0.046 | 0.365 | 0.034 | 2.87E-06 | 0.175 | FALSE | 22 |
|  | rs389558 | T | C | -0.783 | -0.004 | 0.160 | 0.021 | 9.74E-07 | 0.854 | FALSE | 24 |
|  | rs491806 | C | A | -0.948 | 0.028 | 0.211 | 0.026 | 6.92E-06 | 0.271 | FALSE | 20 |
|  | rs529324 | A | G | -0.826 | 0.016 | 0.179 | 0.022 | 4.16E-06 | 0.473 | FALSE | 21 |
|  | rs7778345 | G | A | 0.730 | 0.013 | 0.163 | 0.021 | 7.05E-06 | 0.528 | FALSE | 20 |
| genus.Parabacteroides(id.954) | rs10513202 | G | A | 1.427 | -0.004 | 0.321 | 0.017 | 8.58E-06 | 0.818 | FALSE | 20 |
|  | rs11090513 | T | G | 0.798 | 0.008 | 0.175 | 0.013 | 5.00E-06 | 0.509 | FALSE | 21 |
|  | rs11230859 | A | G | -0.725 | 0.006 | 0.158 | 0.011 | 4.18E-06 | 0.591 | FALSE | 21 |
|  | rs12669698 | C | T | 1.127 | 0.015 | 0.255 | 0.017 | 9.73E-06 | 0.366 | FALSE | 20 |
|  | rs17145573 | A | G | 1.708 | 0.007 | 0.365 | 0.017 | 2.87E-06 | 0.680 | FALSE | 22 |
|  | rs389558 | T | C | -0.783 | 0.012 | 0.160 | 0.011 | 9.74E-07 | 0.275 | FALSE | 24 |
|  | rs491806 | C | A | -0.948 | -0.010 | 0.211 | 0.013 | 6.92E-06 | 0.468 | FALSE | 20 |
|  | rs529324 | A | G | -0.826 | 0.007 | 0.179 | 0.012 | 4.16E-06 | 0.567 | FALSE | 21 |
|  | rs7778345 | G | A | 0.730 | -0.010 | 0.163 | 0.011 | 7.05E-06 | 0.367 | FALSE | 20 |
| genus.Paraprevotella(id.962) | rs10513202 | G | A | 1.427 | 0.024 | 0.321 | 0.027 | 8.58E-06 | 0.364 | FALSE | 20 |
|  | rs11090513 | T | G | 0.798 | 0.011 | 0.175 | 0.020 | 5.00E-06 | 0.567 | FALSE | 21 |
|  | rs11230859 | A | G | -0.725 | 0.001 | 0.158 | 0.017 | 4.18E-06 | 0.946 | FALSE | 21 |
|  | rs12669698 | C | T | 1.127 | -0.013 | 0.255 | 0.025 | 9.73E-06 | 0.614 | FALSE | 20 |
|  | rs17145573 | A | G | 1.708 | -0.021 | 0.365 | 0.026 | 2.87E-06 | 0.430 | FALSE | 22 |
|  | rs389558 | T | C | -0.783 | 0.000 | 0.160 | 0.017 | 9.74E-07 | 0.979 | FALSE | 24 |
|  | rs491806 | C | A | -0.948 | -0.018 | 0.211 | 0.021 | 6.92E-06 | 0.375 | FALSE | 20 |
|  | rs529324 | A | G | -0.826 | 0.011 | 0.179 | 0.018 | 4.16E-06 | 0.557 | FALSE | 21 |
|  | rs7778345 | G | A | 0.730 | -0.006 | 0.163 | 0.017 | 7.05E-06 | 0.732 | FALSE | 20 |
| genus.Parasutterella(id.2892) | rs10513202 | G | A | 1.427 | 0.035 | 0.321 | 0.021 | 8.58E-06 | 0.097 | FALSE | 20 |
|  | rs11090513 | T | G | 0.798 | -0.022 | 0.175 | 0.016 | 5.00E-06 | 0.152 | FALSE | 21 |
|  | rs11230859 | A | G | -0.725 | 0.000 | 0.158 | 0.014 | 4.18E-06 | 0.979 | FALSE | 21 |
|  | rs12669698 | C | T | 1.127 | 0.053 | 0.255 | 0.021 | 9.73E-06 | 0.011 | FALSE | 20 |
|  | rs17145573 | A | G | 1.708 | 0.011 | 0.365 | 0.021 | 2.87E-06 | 0.616 | FALSE | 22 |
|  | rs389558 | T | C | -0.783 | -0.011 | 0.160 | 0.014 | 9.74E-07 | 0.403 | FALSE | 24 |
|  | rs491806 | C | A | -0.948 | -0.047 | 0.211 | 0.016 | 6.92E-06 | 0.004 | FALSE | 20 |
|  | rs529324 | A | G | -0.826 | -0.004 | 0.179 | 0.014 | 4.16E-06 | 0.792 | FALSE | 21 |
|  | rs7778345 | G | A | 0.730 | -0.020 | 0.163 | 0.014 | 7.05E-06 | 0.133 | FALSE | 20 |
| genus.Peptococcus(id.2037) | rs10513202 | G | A | 1.427 | -0.016 | 0.321 | 0.031 | 8.58E-06 | 0.594 | FALSE | 20 |
|  | rs11090513 | T | G | 0.798 | 0.007 | 0.175 | 0.022 | 5.00E-06 | 0.766 | FALSE | 21 |
|  | rs11230859 | A | G | -0.725 | -0.002 | 0.158 | 0.020 | 4.18E-06 | 0.922 | FALSE | 21 |
|  | rs12669698 | C | T | 1.127 | -0.055 | 0.255 | 0.031 | 9.73E-06 | 0.072 | FALSE | 20 |
|  | rs17145573 | A | G | 1.708 | 0.036 | 0.365 | 0.031 | 2.87E-06 | 0.235 | FALSE | 22 |
|  | rs389558 | T | C | -0.783 | 0.004 | 0.160 | 0.019 | 9.74E-07 | 0.854 | FALSE | 24 |
|  | rs491806 | C | A | -0.948 | -0.008 | 0.211 | 0.023 | 6.92E-06 | 0.730 | FALSE | 20 |
|  | rs529324 | A | G | -0.826 | -0.012 | 0.179 | 0.020 | 4.16E-06 | 0.563 | FALSE | 21 |
|  | rs7778345 | G | A | 0.730 | 0.022 | 0.163 | 0.019 | 7.05E-06 | 0.259 | FALSE | 20 |
| genus.Phascolarctobacterium(id.2168) | rs10513202 | G | A | 1.427 | 0.031 | 0.321 | 0.022 | 8.58E-06 | 0.153 | FALSE | 20 |
|  | rs11090513 | T | G | 0.798 | 0.002 | 0.175 | 0.016 | 5.00E-06 | 0.917 | FALSE | 21 |
|  | rs11230859 | A | G | -0.725 | 0.001 | 0.158 | 0.014 | 4.18E-06 | 0.940 | FALSE | 21 |
|  | rs12669698 | C | T | 1.127 | 0.020 | 0.255 | 0.021 | 9.73E-06 | 0.325 | FALSE | 20 |
|  | rs17145573 | A | G | 1.708 | -0.065 | 0.365 | 0.021 | 2.87E-06 | 0.003 | FALSE | 22 |
|  | rs389558 | T | C | -0.783 | -0.014 | 0.160 | 0.014 | 9.74E-07 | 0.313 | FALSE | 24 |
|  | rs491806 | C | A | -0.948 | -0.012 | 0.211 | 0.016 | 6.92E-06 | 0.474 | FALSE | 20 |
|  | rs529324 | A | G | -0.826 | 0.015 | 0.179 | 0.015 | 4.16E-06 | 0.297 | FALSE | 21 |
|  | rs7778345 | G | A | 0.730 | 0.012 | 0.163 | 0.014 | 7.05E-06 | 0.373 | FALSE | 20 |
| genus.Prevotella7(id.11182) | rs10513202 | G | A | 1.427 | -0.019 | 0.321 | 0.038 | 8.58E-06 | 0.621 | FALSE | 20 |
|  | rs11090513 | T | G | 0.798 | -0.007 | 0.175 | 0.028 | 5.00E-06 | 0.793 | FALSE | 21 |
|  | rs11230859 | A | G | -0.725 | 0.022 | 0.158 | 0.025 | 4.18E-06 | 0.377 | FALSE | 21 |
|  | rs12669698 | C | T | 1.127 | -0.039 | 0.255 | 0.038 | 9.73E-06 | 0.297 | FALSE | 20 |
|  | rs17145573 | A | G | 1.708 | -0.072 | 0.365 | 0.039 | 2.87E-06 | 0.066 | FALSE | 22 |
|  | rs389558 | T | C | -0.783 | 0.020 | 0.160 | 0.025 | 9.74E-07 | 0.430 | FALSE | 24 |
|  | rs491806 | C | A | -0.948 | 0.040 | 0.211 | 0.029 | 6.92E-06 | 0.171 | FALSE | 20 |
|  | rs529324 | A | G | -0.826 | 0.051 | 0.179 | 0.026 | 4.16E-06 | 0.048 | FALSE | 21 |
|  | rs7778345 | G | A | 0.730 | 0.010 | 0.163 | 0.024 | 7.05E-06 | 0.691 | FALSE | 20 |
| genus.Prevotella9(id.11183) | rs10513202 | G | A | 1.427 | 0.039 | 0.321 | 0.022 | 8.58E-06 | 0.084 | FALSE | 20 |
|  | rs11090513 | T | G | 0.798 | -0.003 | 0.175 | 0.017 | 5.00E-06 | 0.864 | FALSE | 21 |
|  | rs11230859 | A | G | -0.725 | -0.025 | 0.158 | 0.015 | 4.18E-06 | 0.090 | FALSE | 21 |
|  | rs12669698 | C | T | 1.127 | -0.034 | 0.255 | 0.021 | 9.73E-06 | 0.106 | FALSE | 20 |
|  | rs17145573 | A | G | 1.708 | 0.001 | 0.365 | 0.022 | 2.87E-06 | 0.955 | FALSE | 22 |
|  | rs389558 | T | C | -0.783 | 0.010 | 0.160 | 0.014 | 9.74E-07 | 0.509 | FALSE | 24 |
|  | rs491806 | C | A | -0.948 | -0.007 | 0.211 | 0.017 | 6.92E-06 | 0.676 | FALSE | 20 |
|  | rs529324 | A | G | -0.826 | 0.006 | 0.179 | 0.015 | 4.16E-06 | 0.703 | FALSE | 21 |
|  | rs7778345 | G | A | 0.730 | 0.031 | 0.163 | 0.015 | 7.05E-06 | 0.031 | FALSE | 20 |
| genus.RikenellaceaeRC9gutgroup(id.11191) | rs10513202 | G | A | 1.427 | -0.021 | 0.321 | 0.040 | 8.58E-06 | 0.602 | FALSE | 20 |
|  | rs11090513 | T | G | 0.798 | 0.006 | 0.175 | 0.029 | 5.00E-06 | 0.847 | FALSE | 21 |
|  | rs11230859 | A | G | -0.725 | 0.005 | 0.158 | 0.026 | 4.18E-06 | 0.850 | FALSE | 21 |
|  | rs12669698 | C | T | 1.127 | -0.080 | 0.255 | 0.039 | 9.73E-06 | 0.039 | FALSE | 20 |
|  | rs17145573 | A | G | 1.708 | 0.037 | 0.365 | 0.040 | 2.87E-06 | 0.346 | FALSE | 22 |
|  | rs389558 | T | C | -0.783 | 0.000 | 0.160 | 0.025 | 9.74E-07 | 0.986 | FALSE | 24 |
|  | rs491806 | C | A | -0.948 | -0.020 | 0.211 | 0.030 | 6.92E-06 | 0.502 | FALSE | 20 |
|  | rs529324 | A | G | -0.826 | 0.034 | 0.179 | 0.027 | 4.16E-06 | 0.203 | FALSE | 21 |
|  | rs7778345 | G | A | 0.730 | 0.048 | 0.163 | 0.025 | 7.05E-06 | 0.056 | FALSE | 20 |
| genus.Romboutsia(id.11347) | rs10513202 | G | A | 1.427 | 0.003 | 0.321 | 0.019 | 8.58E-06 | 0.887 | FALSE | 20 |
|  | rs11090513 | T | G | 0.798 | 0.002 | 0.175 | 0.014 | 5.00E-06 | 0.897 | FALSE | 21 |
|  | rs11230859 | A | G | -0.725 | -0.011 | 0.158 | 0.012 | 4.18E-06 | 0.391 | FALSE | 21 |
|  | rs12669698 | C | T | 1.127 | 0.016 | 0.255 | 0.019 | 9.73E-06 | 0.377 | FALSE | 20 |
|  | rs17145573 | A | G | 1.708 | 0.003 | 0.365 | 0.019 | 2.87E-06 | 0.867 | FALSE | 22 |
|  | rs389558 | T | C | -0.783 | 0.006 | 0.160 | 0.012 | 9.74E-07 | 0.617 | FALSE | 24 |
|  | rs491806 | C | A | -0.948 | -0.002 | 0.211 | 0.015 | 6.92E-06 | 0.900 | FALSE | 20 |
|  | rs529324 | A | G | -0.826 | 0.010 | 0.179 | 0.013 | 4.16E-06 | 0.422 | FALSE | 21 |
|  | rs7778345 | G | A | 0.730 | 0.020 | 0.163 | 0.012 | 7.05E-06 | 0.095 | FALSE | 20 |
| genus.Roseburia(id.2012) | rs10513202 | G | A | 1.427 | -0.026 | 0.321 | 0.017 | 8.58E-06 | 0.124 | FALSE | 20 |
|  | rs11090513 | T | G | 0.798 | -0.022 | 0.175 | 0.012 | 5.00E-06 | 0.078 | FALSE | 21 |
|  | rs11230859 | A | G | -0.725 | -0.013 | 0.158 | 0.011 | 4.18E-06 | 0.242 | FALSE | 21 |
|  | rs12669698 | C | T | 1.127 | 0.030 | 0.255 | 0.016 | 9.73E-06 | 0.067 | FALSE | 20 |
|  | rs17145573 | A | G | 1.708 | -0.026 | 0.365 | 0.017 | 2.87E-06 | 0.131 | FALSE | 22 |
|  | rs389558 | T | C | -0.783 | 0.017 | 0.160 | 0.011 | 9.74E-07 | 0.110 | FALSE | 24 |
|  | rs491806 | C | A | -0.948 | -0.002 | 0.211 | 0.013 | 6.92E-06 | 0.848 | FALSE | 20 |
|  | rs529324 | A | G | -0.826 | 0.003 | 0.179 | 0.011 | 4.16E-06 | 0.807 | FALSE | 21 |
|  | rs7778345 | G | A | 0.730 | -0.012 | 0.163 | 0.011 | 7.05E-06 | 0.279 | FALSE | 20 |
| genus.Ruminiclostridium5(id.11355) | rs10513202 | G | A | 1.427 | -0.015 | 0.321 | 0.017 | 8.58E-06 | 0.378 | FALSE | 20 |
|  | rs11090513 | T | G | 0.798 | -0.005 | 0.175 | 0.013 | 5.00E-06 | 0.714 | FALSE | 21 |
|  | rs11230859 | A | G | -0.725 | 0.008 | 0.158 | 0.011 | 4.18E-06 | 0.500 | FALSE | 21 |
|  | rs12669698 | C | T | 1.127 | 0.000 | 0.255 | 0.017 | 9.73E-06 | 0.987 | FALSE | 20 |
|  | rs17145573 | A | G | 1.708 | -0.005 | 0.365 | 0.017 | 2.87E-06 | 0.772 | FALSE | 22 |
|  | rs389558 | T | C | -0.783 | 0.012 | 0.160 | 0.011 | 9.74E-07 | 0.288 | FALSE | 24 |
|  | rs491806 | C | A | -0.948 | 0.009 | 0.211 | 0.013 | 6.92E-06 | 0.488 | FALSE | 20 |
|  | rs529324 | A | G | -0.826 | 0.007 | 0.179 | 0.012 | 4.16E-06 | 0.537 | FALSE | 21 |
|  | rs7778345 | G | A | 0.730 | 0.000 | 0.163 | 0.011 | 7.05E-06 | 0.965 | FALSE | 20 |
| genus.Ruminiclostridium6(id.11356) | rs10513202 | G | A | 1.427 | 0.004 | 0.321 | 0.020 | 8.58E-06 | 0.832 | FALSE | 20 |
|  | rs11090513 | T | G | 0.798 | 0.030 | 0.175 | 0.014 | 5.00E-06 | 0.039 | FALSE | 21 |
|  | rs11230859 | A | G | -0.725 | -0.002 | 0.158 | 0.013 | 4.18E-06 | 0.863 | FALSE | 21 |
|  | rs12669698 | C | T | 1.127 | -0.007 | 0.255 | 0.019 | 9.73E-06 | 0.702 | FALSE | 20 |
|  | rs17145573 | A | G | 1.708 | -0.042 | 0.365 | 0.020 | 2.87E-06 | 0.036 | FALSE | 22 |
|  | rs389558 | T | C | -0.783 | 0.010 | 0.160 | 0.013 | 9.74E-07 | 0.413 | FALSE | 24 |
|  | rs491806 | C | A | -0.948 | -0.013 | 0.211 | 0.015 | 6.92E-06 | 0.399 | FALSE | 20 |
|  | rs529324 | A | G | -0.826 | -0.014 | 0.179 | 0.013 | 4.16E-06 | 0.284 | FALSE | 21 |
|  | rs7778345 | G | A | 0.730 | -0.008 | 0.163 | 0.012 | 7.05E-06 | 0.546 | FALSE | 20 |
| genus.Ruminiclostridium9(id.11357) | rs10513202 | G | A | 1.427 | 0.003 | 0.321 | 0.018 | 8.58E-06 | 0.858 | FALSE | 20 |
|  | rs11090513 | T | G | 0.798 | -0.013 | 0.175 | 0.013 | 5.00E-06 | 0.301 | FALSE | 21 |
|  | rs11230859 | A | G | -0.725 | -0.006 | 0.158 | 0.011 | 4.18E-06 | 0.627 | FALSE | 21 |
|  | rs12669698 | C | T | 1.127 | -0.010 | 0.255 | 0.017 | 9.73E-06 | 0.577 | FALSE | 20 |
|  | rs17145573 | A | G | 1.708 | -0.008 | 0.365 | 0.018 | 2.87E-06 | 0.662 | FALSE | 22 |
|  | rs389558 | T | C | -0.783 | -0.002 | 0.160 | 0.011 | 9.74E-07 | 0.870 | FALSE | 24 |
|  | rs491806 | C | A | -0.948 | 0.001 | 0.211 | 0.013 | 6.92E-06 | 0.946 | FALSE | 20 |
|  | rs529324 | A | G | -0.826 | 0.004 | 0.179 | 0.012 | 4.16E-06 | 0.749 | FALSE | 21 |
|  | rs7778345 | G | A | 0.730 | -0.001 | 0.163 | 0.011 | 7.05E-06 | 0.932 | FALSE | 20 |
| genus.RuminococcaceaeNK4A214group(id.11358) | rs10513202 | G | A | 1.427 | 0.004 | 0.321 | 0.018 | 8.58E-06 | 0.814 | FALSE | 20 |
|  | rs11090513 | T | G | 0.798 | 0.023 | 0.175 | 0.013 | 5.00E-06 | 0.095 | FALSE | 21 |
|  | rs11230859 | A | G | -0.725 | 0.007 | 0.158 | 0.012 | 4.18E-06 | 0.587 | FALSE | 21 |
|  | rs12669698 | C | T | 1.127 | -0.009 | 0.255 | 0.018 | 9.73E-06 | 0.627 | FALSE | 20 |
|  | rs17145573 | A | G | 1.708 | -0.002 | 0.365 | 0.018 | 2.87E-06 | 0.934 | FALSE | 22 |
|  | rs389558 | T | C | -0.783 | 0.002 | 0.160 | 0.012 | 9.74E-07 | 0.851 | FALSE | 24 |
|  | rs491806 | C | A | -0.948 | 0.022 | 0.211 | 0.014 | 6.92E-06 | 0.117 | FALSE | 20 |
|  | rs529324 | A | G | -0.826 | -0.001 | 0.179 | 0.012 | 4.16E-06 | 0.910 | FALSE | 21 |
|  | rs7778345 | G | A | 0.730 | 0.003 | 0.163 | 0.012 | 7.05E-06 | 0.807 | FALSE | 20 |
| genus.RuminococcaceaeUCG002(id.11360) | rs10513202 | G | A | 1.427 | 0.001 | 0.321 | 0.017 | 8.58E-06 | 0.942 | FALSE | 20 |
|  | rs11090513 | T | G | 0.798 | 0.022 | 0.175 | 0.013 | 5.00E-06 | 0.091 | FALSE | 21 |
|  | rs11230859 | A | G | -0.725 | 0.007 | 0.158 | 0.011 | 4.18E-06 | 0.531 | FALSE | 21 |
|  | rs12669698 | C | T | 1.127 | -0.009 | 0.255 | 0.017 | 9.73E-06 | 0.584 | FALSE | 20 |
|  | rs17145573 | A | G | 1.708 | -0.043 | 0.365 | 0.017 | 2.87E-06 | 0.014 | FALSE | 22 |
|  | rs389558 | T | C | -0.783 | 0.007 | 0.160 | 0.011 | 9.74E-07 | 0.542 | FALSE | 24 |
|  | rs491806 | C | A | -0.948 | 0.002 | 0.211 | 0.013 | 6.92E-06 | 0.861 | FALSE | 20 |
|  | rs529324 | A | G | -0.826 | 0.009 | 0.179 | 0.012 | 4.16E-06 | 0.440 | FALSE | 21 |
|  | rs7778345 | G | A | 0.730 | 0.002 | 0.163 | 0.011 | 7.05E-06 | 0.885 | FALSE | 20 |
| genus.RuminococcaceaeUCG003(id.11361) | rs10513202 | G | A | 1.427 | 0.018 | 0.321 | 0.019 | 8.58E-06 | 0.351 | FALSE | 20 |
|  | rs11090513 | T | G | 0.798 | 0.009 | 0.175 | 0.014 | 5.00E-06 | 0.499 | FALSE | 21 |
|  | rs11230859 | A | G | -0.725 | -0.001 | 0.158 | 0.012 | 4.18E-06 | 0.966 | FALSE | 21 |
|  | rs12669698 | C | T | 1.127 | 0.015 | 0.255 | 0.018 | 9.73E-06 | 0.415 | FALSE | 20 |
|  | rs17145573 | A | G | 1.708 | -0.029 | 0.365 | 0.019 | 2.87E-06 | 0.124 | FALSE | 22 |
|  | rs389558 | T | C | -0.783 | 0.009 | 0.160 | 0.012 | 9.74E-07 | 0.440 | FALSE | 24 |
|  | rs491806 | C | A | -0.948 | 0.004 | 0.211 | 0.014 | 6.92E-06 | 0.782 | FALSE | 20 |
|  | rs529324 | A | G | -0.826 | -0.002 | 0.179 | 0.013 | 4.16E-06 | 0.857 | FALSE | 21 |
|  | rs7778345 | G | A | 0.730 | 0.008 | 0.163 | 0.012 | 7.05E-06 | 0.528 | FALSE | 20 |
| genus.RuminococcaceaeUCG004(id.11362) | rs10513202 | G | A | 1.427 | -0.003 | 0.321 | 0.024 | 8.58E-06 | 0.904 | FALSE | 20 |
|  | rs11090513 | T | G | 0.798 | 0.003 | 0.175 | 0.017 | 5.00E-06 | 0.842 | FALSE | 21 |
|  | rs11230859 | A | G | -0.725 | -0.024 | 0.158 | 0.015 | 4.18E-06 | 0.107 | FALSE | 21 |
|  | rs12669698 | C | T | 1.127 | 0.020 | 0.255 | 0.023 | 9.73E-06 | 0.374 | FALSE | 20 |
|  | rs17145573 | A | G | 1.708 | -0.003 | 0.365 | 0.024 | 2.87E-06 | 0.915 | FALSE | 22 |
|  | rs389558 | T | C | -0.783 | 0.012 | 0.160 | 0.015 | 9.74E-07 | 0.397 | FALSE | 24 |
|  | rs491806 | C | A | -0.948 | 0.023 | 0.211 | 0.018 | 6.92E-06 | 0.207 | FALSE | 20 |
|  | rs529324 | A | G | -0.826 | 0.008 | 0.179 | 0.016 | 4.16E-06 | 0.602 | FALSE | 21 |
|  | rs7778345 | G | A | 0.730 | 0.024 | 0.163 | 0.015 | 7.05E-06 | 0.105 | FALSE | 20 |
| genus.RuminococcaceaeUCG005(id.11363) | rs10513202 | G | A | 1.427 | 0.029 | 0.321 | 0.018 | 8.58E-06 | 0.111 | FALSE | 20 |
|  | rs11090513 | T | G | 0.798 | 0.004 | 0.175 | 0.013 | 5.00E-06 | 0.751 | FALSE | 21 |
|  | rs11230859 | A | G | -0.725 | 0.004 | 0.158 | 0.012 | 4.18E-06 | 0.716 | FALSE | 21 |
|  | rs12669698 | C | T | 1.127 | -0.014 | 0.255 | 0.017 | 9.73E-06 | 0.417 | FALSE | 20 |
|  | rs17145573 | A | G | 1.708 | -0.019 | 0.365 | 0.018 | 2.87E-06 | 0.282 | FALSE | 22 |
|  | rs389558 | T | C | -0.783 | 0.013 | 0.160 | 0.011 | 9.74E-07 | 0.244 | FALSE | 24 |
|  | rs491806 | C | A | -0.948 | 0.015 | 0.211 | 0.014 | 6.92E-06 | 0.281 | FALSE | 20 |
|  | rs529324 | A | G | -0.826 | 0.001 | 0.179 | 0.012 | 4.16E-06 | 0.944 | FALSE | 21 |
|  | rs7778345 | G | A | 0.730 | 0.020 | 0.163 | 0.011 | 7.05E-06 | 0.077 | FALSE | 20 |
| genus.RuminococcaceaeUCG009(id.11366) | rs10513202 | G | A | 1.427 | 0.066 | 0.321 | 0.027 | 8.58E-06 | 0.013 | FALSE | 20 |
|  | rs11090513 | T | G | 0.798 | -0.008 | 0.175 | 0.019 | 5.00E-06 | 0.671 | FALSE | 21 |
|  | rs11230859 | A | G | -0.725 | -0.007 | 0.158 | 0.017 | 4.18E-06 | 0.665 | FALSE | 21 |
|  | rs12669698 | C | T | 1.127 | -0.027 | 0.255 | 0.026 | 9.73E-06 | 0.305 | FALSE | 20 |
|  | rs17145573 | A | G | 1.708 | 0.024 | 0.365 | 0.026 | 2.87E-06 | 0.356 | FALSE | 22 |
|  | rs389558 | T | C | -0.783 | 0.020 | 0.160 | 0.017 | 9.74E-07 | 0.238 | FALSE | 24 |
|  | rs491806 | C | A | -0.948 | -0.016 | 0.211 | 0.020 | 6.92E-06 | 0.429 | FALSE | 20 |
|  | rs529324 | A | G | -0.826 | 0.002 | 0.179 | 0.018 | 4.16E-06 | 0.889 | FALSE | 21 |
|  | rs7778345 | G | A | 0.730 | 0.002 | 0.163 | 0.017 | 7.05E-06 | 0.883 | FALSE | 20 |
| genus.RuminococcaceaeUCG010(id.11367) | rs10513202 | G | A | 1.427 | 0.016 | 0.321 | 0.020 | 8.58E-06 | 0.429 | FALSE | 20 |
|  | rs11090513 | T | G | 0.798 | 0.019 | 0.175 | 0.015 | 5.00E-06 | 0.201 | FALSE | 21 |
|  | rs11230859 | A | G | -0.725 | 0.016 | 0.158 | 0.013 | 4.18E-06 | 0.221 | FALSE | 21 |
|  | rs12669698 | C | T | 1.127 | -0.008 | 0.255 | 0.020 | 9.73E-06 | 0.694 | FALSE | 20 |
|  | rs17145573 | A | G | 1.708 | -0.022 | 0.365 | 0.020 | 2.87E-06 | 0.267 | FALSE | 22 |
|  | rs389558 | T | C | -0.783 | 0.017 | 0.160 | 0.013 | 9.74E-07 | 0.194 | FALSE | 24 |
|  | rs491806 | C | A | -0.948 | 0.030 | 0.211 | 0.015 | 6.92E-06 | 0.053 | FALSE | 20 |
|  | rs529324 | A | G | -0.826 | -0.003 | 0.179 | 0.014 | 4.16E-06 | 0.804 | FALSE | 21 |
|  | rs7778345 | G | A | 0.730 | -0.005 | 0.163 | 0.013 | 7.05E-06 | 0.695 | FALSE | 20 |
| genus.RuminococcaceaeUCG011(id.11368) | rs10513202 | G | A | 1.427 | -0.021 | 0.321 | 0.038 | 8.58E-06 | 0.578 | FALSE | 20 |
|  | rs11090513 | T | G | 0.798 | 0.026 | 0.175 | 0.027 | 5.00E-06 | 0.339 | FALSE | 21 |
|  | rs11230859 | A | G | -0.725 | 0.001 | 0.158 | 0.025 | 4.18E-06 | 0.972 | FALSE | 21 |
|  | rs12669698 | C | T | 1.127 | 0.019 | 0.255 | 0.038 | 9.73E-06 | 0.615 | FALSE | 20 |
|  | rs17145573 | A | G | 1.708 | 0.018 | 0.365 | 0.039 | 2.87E-06 | 0.647 | FALSE | 22 |
|  | rs389558 | T | C | -0.783 | 0.007 | 0.160 | 0.024 | 9.74E-07 | 0.762 | FALSE | 24 |
|  | rs491806 | C | A | -0.948 | -0.026 | 0.211 | 0.029 | 6.92E-06 | 0.361 | FALSE | 20 |
|  | rs529324 | A | G | -0.826 | -0.032 | 0.179 | 0.025 | 4.16E-06 | 0.211 | FALSE | 21 |
|  | rs7778345 | G | A | 0.730 | -0.021 | 0.163 | 0.024 | 7.05E-06 | 0.381 | FALSE | 20 |
| genus.RuminococcaceaeUCG013(id.11370) | rs10513202 | G | A | 1.427 | -0.025 | 0.321 | 0.018 | 8.58E-06 | 0.161 | FALSE | 20 |
|  | rs11090513 | T | G | 0.798 | 0.018 | 0.175 | 0.013 | 5.00E-06 | 0.163 | FALSE | 21 |
|  | rs11230859 | A | G | -0.725 | -0.009 | 0.158 | 0.011 | 4.18E-06 | 0.456 | FALSE | 21 |
|  | rs12669698 | C | T | 1.127 | -0.019 | 0.255 | 0.017 | 9.73E-06 | 0.279 | FALSE | 20 |
|  | rs17145573 | A | G | 1.708 | 0.005 | 0.365 | 0.018 | 2.87E-06 | 0.772 | FALSE | 22 |
|  | rs389558 | T | C | -0.783 | -0.008 | 0.160 | 0.011 | 9.74E-07 | 0.494 | FALSE | 24 |
|  | rs491806 | C | A | -0.948 | 0.040 | 0.211 | 0.013 | 6.92E-06 | 0.003 | FALSE | 20 |
|  | rs529324 | A | G | -0.826 | 0.008 | 0.179 | 0.012 | 4.16E-06 | 0.486 | FALSE | 21 |
|  | rs7778345 | G | A | 0.730 | -0.009 | 0.163 | 0.011 | 7.05E-06 | 0.419 | FALSE | 20 |
| genus.RuminococcaceaeUCG014(id.11371) | rs10513202 | G | A | 1.427 | -0.025 | 0.321 | 0.019 | 8.58E-06 | 0.181 | FALSE | 20 |
|  | rs11090513 | T | G | 0.798 | -0.008 | 0.175 | 0.014 | 5.00E-06 | 0.545 | FALSE | 21 |
|  | rs11230859 | A | G | -0.725 | 0.000 | 0.158 | 0.012 | 4.18E-06 | 0.987 | FALSE | 21 |
|  | rs12669698 | C | T | 1.127 | -0.009 | 0.255 | 0.019 | 9.73E-06 | 0.630 | FALSE | 20 |
|  | rs17145573 | A | G | 1.708 | -0.007 | 0.365 | 0.019 | 2.87E-06 | 0.730 | FALSE | 22 |
|  | rs389558 | T | C | -0.783 | -0.007 | 0.160 | 0.012 | 9.74E-07 | 0.552 | FALSE | 24 |
|  | rs491806 | C | A | -0.948 | 0.013 | 0.211 | 0.014 | 6.92E-06 | 0.387 | FALSE | 20 |
|  | rs529324 | A | G | -0.826 | -0.010 | 0.179 | 0.013 | 4.16E-06 | 0.438 | FALSE | 21 |
|  | rs7778345 | G | A | 0.730 | 0.005 | 0.163 | 0.012 | 7.05E-06 | 0.691 | FALSE | 20 |
| genus.Ruminococcus1(id.11373) | rs10513202 | G | A | 1.427 | 0.012 | 0.321 | 0.018 | 8.58E-06 | 0.489 | FALSE | 20 |
|  | rs11090513 | T | G | 0.798 | -0.006 | 0.175 | 0.013 | 5.00E-06 | 0.635 | FALSE | 21 |
|  | rs11230859 | A | G | -0.725 | 0.005 | 0.158 | 0.011 | 4.18E-06 | 0.665 | FALSE | 21 |
|  | rs12669698 | C | T | 1.127 | 0.017 | 0.255 | 0.017 | 9.73E-06 | 0.320 | FALSE | 20 |
|  | rs17145573 | A | G | 1.708 | 0.001 | 0.365 | 0.018 | 2.87E-06 | 0.974 | FALSE | 22 |
|  | rs389558 | T | C | -0.783 | -0.012 | 0.160 | 0.011 | 9.74E-07 | 0.302 | FALSE | 24 |
|  | rs491806 | C | A | -0.948 | -0.003 | 0.211 | 0.013 | 6.92E-06 | 0.819 | FALSE | 20 |
|  | rs529324 | A | G | -0.826 | 0.016 | 0.179 | 0.012 | 4.16E-06 | 0.184 | FALSE | 21 |
|  | rs7778345 | G | A | 0.730 | -0.010 | 0.163 | 0.011 | 7.05E-06 | 0.379 | FALSE | 20 |
| genus.Ruminococcus2(id.11374) | rs10513202 | G | A | 1.427 | -0.007 | 0.321 | 0.018 | 8.58E-06 | 0.707 | FALSE | 20 |
|  | rs11090513 | T | G | 0.798 | 0.002 | 0.175 | 0.013 | 5.00E-06 | 0.893 | FALSE | 21 |
|  | rs11230859 | A | G | -0.725 | -0.008 | 0.158 | 0.012 | 4.18E-06 | 0.489 | FALSE | 21 |
|  | rs12669698 | C | T | 1.127 | -0.029 | 0.255 | 0.018 | 9.73E-06 | 0.106 | FALSE | 20 |
|  | rs17145573 | A | G | 1.708 | 0.002 | 0.365 | 0.018 | 2.87E-06 | 0.920 | FALSE | 22 |
|  | rs389558 | T | C | -0.783 | -0.015 | 0.160 | 0.012 | 9.74E-07 | 0.197 | FALSE | 24 |
|  | rs491806 | C | A | -0.948 | 0.005 | 0.211 | 0.014 | 6.92E-06 | 0.719 | FALSE | 20 |
|  | rs529324 | A | G | -0.826 | 0.011 | 0.179 | 0.012 | 4.16E-06 | 0.371 | FALSE | 21 |
|  | rs7778345 | G | A | 0.730 | -0.013 | 0.163 | 0.012 | 7.05E-06 | 0.281 | FALSE | 20 |
| genus.Sellimonas(id.14369) | rs10513202 | G | A | 1.427 | -0.058 | 0.321 | 0.040 | 8.58E-06 | 0.154 | FALSE | 20 |
|  | rs11090513 | T | G | 0.798 | -0.041 | 0.175 | 0.029 | 5.00E-06 | 0.155 | FALSE | 21 |
|  | rs11230859 | A | G | -0.725 | 0.066 | 0.158 | 0.026 | 4.18E-06 | 0.013 | FALSE | 21 |
|  | rs12669698 | C | T | 1.127 | 0.019 | 0.255 | 0.041 | 9.73E-06 | 0.635 | FALSE | 20 |
|  | rs17145573 | A | G | 1.708 | 0.033 | 0.365 | 0.042 | 2.87E-06 | 0.423 | FALSE | 22 |
|  | rs389558 | T | C | -0.783 | -0.044 | 0.160 | 0.026 | 9.74E-07 | 0.089 | FALSE | 24 |
|  | rs491806 | C | A | -0.948 | -0.018 | 0.211 | 0.031 | 6.92E-06 | 0.570 | FALSE | 20 |
|  | rs529324 | A | G | -0.826 | -0.035 | 0.179 | 0.027 | 4.16E-06 | 0.195 | FALSE | 21 |
|  | rs7778345 | G | A | 0.730 | -0.021 | 0.163 | 0.025 | 7.05E-06 | 0.410 | FALSE | 20 |
| genus.Senegalimassilia(id.11160) | rs10513202 | G | A | 1.427 | 0.005 | 0.321 | 0.028 | 8.58E-06 | 0.847 | FALSE | 20 |
|  | rs11090513 | T | G | 0.798 | 0.034 | 0.175 | 0.020 | 5.00E-06 | 0.085 | FALSE | 21 |
|  | rs11230859 | A | G | -0.725 | 0.012 | 0.158 | 0.018 | 4.18E-06 | 0.498 | FALSE | 21 |
|  | rs12669698 | C | T | 1.127 | 0.047 | 0.255 | 0.027 | 9.73E-06 | 0.087 | FALSE | 20 |
|  | rs17145573 | A | G | 1.708 | 0.001 | 0.365 | 0.027 | 2.87E-06 | 0.965 | FALSE | 22 |
|  | rs389558 | T | C | -0.783 | -0.005 | 0.160 | 0.017 | 9.74E-07 | 0.787 | FALSE | 24 |
|  | rs491806 | C | A | -0.948 | -0.013 | 0.211 | 0.021 | 6.92E-06 | 0.522 | FALSE | 20 |
|  | rs529324 | A | G | -0.826 | -0.005 | 0.179 | 0.018 | 4.16E-06 | 0.795 | FALSE | 21 |
|  | rs7778345 | G | A | 0.730 | 0.025 | 0.163 | 0.017 | 7.05E-06 | 0.152 | FALSE | 20 |
| genus.Slackia(id.825) | rs10513202 | G | A | 1.427 | -0.009 | 0.321 | 0.029 | 8.58E-06 | 0.765 | FALSE | 20 |
|  | rs11090513 | T | G | 0.798 | -0.019 | 0.175 | 0.021 | 5.00E-06 | 0.360 | FALSE | 21 |
|  | rs11230859 | A | G | -0.725 | -0.023 | 0.158 | 0.019 | 4.18E-06 | 0.223 | FALSE | 21 |
|  | rs12669698 | C | T | 1.127 | 0.047 | 0.255 | 0.029 | 9.73E-06 | 0.109 | FALSE | 20 |
|  | rs17145573 | A | G | 1.708 | 0.006 | 0.365 | 0.030 | 2.87E-06 | 0.828 | FALSE | 22 |
|  | rs389558 | T | C | -0.783 | -0.022 | 0.160 | 0.018 | 9.74E-07 | 0.231 | FALSE | 24 |
|  | rs491806 | C | A | -0.948 | -0.037 | 0.211 | 0.022 | 6.92E-06 | 0.098 | FALSE | 20 |
|  | rs529324 | A | G | -0.826 | 0.031 | 0.179 | 0.020 | 4.16E-06 | 0.114 | FALSE | 21 |
|  | rs7778345 | G | A | 0.730 | 0.007 | 0.163 | 0.018 | 7.05E-06 | 0.687 | FALSE | 20 |
| genus.Streptococcus(id.1853) | rs10513202 | G | A | 1.427 | -0.029 | 0.321 | 0.018 | 8.58E-06 | 0.107 | FALSE | 20 |
|  | rs11090513 | T | G | 0.798 | 0.020 | 0.175 | 0.013 | 5.00E-06 | 0.127 | FALSE | 21 |
|  | rs11230859 | A | G | -0.725 | 0.003 | 0.158 | 0.012 | 4.18E-06 | 0.789 | FALSE | 21 |
|  | rs12669698 | C | T | 1.127 | 0.028 | 0.255 | 0.017 | 9.73E-06 | 0.102 | FALSE | 20 |
|  | rs17145573 | A | G | 1.708 | 0.003 | 0.365 | 0.018 | 2.87E-06 | 0.865 | FALSE | 22 |
|  | rs389558 | T | C | -0.783 | -0.018 | 0.160 | 0.011 | 9.74E-07 | 0.115 | FALSE | 24 |
|  | rs491806 | C | A | -0.948 | 0.005 | 0.211 | 0.014 | 6.92E-06 | 0.717 | FALSE | 20 |
|  | rs529324 | A | G | -0.826 | 0.006 | 0.179 | 0.012 | 4.16E-06 | 0.641 | FALSE | 21 |
|  | rs7778345 | G | A | 0.730 | -0.001 | 0.163 | 0.011 | 7.05E-06 | 0.916 | FALSE | 20 |
| genus.Subdoligranulum(id.2070) | rs10513202 | G | A | 1.427 | 0.004 | 0.321 | 0.017 | 8.58E-06 | 0.809 | FALSE | 20 |
|  | rs11090513 | T | G | 0.798 | 0.008 | 0.175 | 0.013 | 5.00E-06 | 0.502 | FALSE | 21 |
|  | rs11230859 | A | G | -0.725 | -0.008 | 0.158 | 0.011 | 4.18E-06 | 0.498 | FALSE | 21 |
|  | rs12669698 | C | T | 1.127 | 0.006 | 0.255 | 0.017 | 9.73E-06 | 0.716 | FALSE | 20 |
|  | rs17145573 | A | G | 1.708 | 0.048 | 0.365 | 0.017 | 2.87E-06 | 0.005 | FALSE | 22 |
|  | rs389558 | T | C | -0.783 | 0.002 | 0.160 | 0.011 | 9.74E-07 | 0.876 | FALSE | 24 |
|  | rs491806 | C | A | -0.948 | 0.004 | 0.211 | 0.013 | 6.92E-06 | 0.741 | FALSE | 20 |
|  | rs529324 | A | G | -0.826 | -0.007 | 0.179 | 0.012 | 4.16E-06 | 0.549 | FALSE | 21 |
|  | rs7778345 | G | A | 0.730 | 0.026 | 0.163 | 0.011 | 7.05E-06 | 0.018 | FALSE | 20 |
| genus.Sutterella(id.2896) | rs10513202 | G | A | 1.427 | 0.016 | 0.321 | 0.020 | 8.58E-06 | 0.427 | FALSE | 20 |
|  | rs11090513 | T | G | 0.798 | 0.003 | 0.175 | 0.015 | 5.00E-06 | 0.860 | FALSE | 21 |
|  | rs11230859 | A | G | -0.725 | 0.000 | 0.158 | 0.013 | 4.18E-06 | 0.983 | FALSE | 21 |
|  | rs12669698 | C | T | 1.127 | -0.010 | 0.255 | 0.019 | 9.73E-06 | 0.615 | FALSE | 20 |
|  | rs17145573 | A | G | 1.708 | -0.013 | 0.365 | 0.020 | 2.87E-06 | 0.513 | FALSE | 22 |
|  | rs389558 | T | C | -0.783 | -0.011 | 0.160 | 0.013 | 9.74E-07 | 0.393 | FALSE | 24 |
|  | rs491806 | C | A | -0.948 | -0.006 | 0.211 | 0.015 | 6.92E-06 | 0.700 | FALSE | 20 |
|  | rs529324 | A | G | -0.826 | -0.001 | 0.179 | 0.013 | 4.16E-06 | 0.947 | FALSE | 21 |
|  | rs7778345 | G | A | 0.730 | -0.003 | 0.163 | 0.013 | 7.05E-06 | 0.826 | FALSE | 20 |
| genus.Terrisporobacter(id.11348) | rs10513202 | G | A | 1.427 | -0.024 | 0.321 | 0.028 | 8.58E-06 | 0.381 | FALSE | 20 |
|  | rs11090513 | T | G | 0.798 | 0.020 | 0.175 | 0.021 | 5.00E-06 | 0.329 | FALSE | 21 |
|  | rs11230859 | A | G | -0.725 | 0.010 | 0.158 | 0.018 | 4.18E-06 | 0.590 | FALSE | 21 |
|  | rs12669698 | C | T | 1.127 | 0.066 | 0.255 | 0.028 | 9.73E-06 | 0.019 | FALSE | 20 |
|  | rs17145573 | A | G | 1.708 | 0.036 | 0.365 | 0.028 | 2.87E-06 | 0.200 | FALSE | 22 |
|  | rs389558 | T | C | -0.783 | 0.038 | 0.160 | 0.018 | 9.74E-07 | 0.036 | FALSE | 24 |
|  | rs491806 | C | A | -0.948 | 0.009 | 0.211 | 0.021 | 6.92E-06 | 0.681 | FALSE | 20 |
|  | rs529324 | A | G | -0.826 | 0.020 | 0.179 | 0.019 | 4.16E-06 | 0.293 | FALSE | 21 |
|  | rs7778345 | G | A | 0.730 | 0.009 | 0.163 | 0.018 | 7.05E-06 | 0.624 | FALSE | 20 |
| genus.Turicibacter(id.2162) | rs10513202 | G | A | 1.427 | 0.004 | 0.321 | 0.024 | 8.58E-06 | 0.857 | FALSE | 20 |
|  | rs11090513 | T | G | 0.798 | 0.000 | 0.175 | 0.018 | 5.00E-06 | 0.980 | FALSE | 21 |
|  | rs11230859 | A | G | -0.725 | -0.006 | 0.158 | 0.016 | 4.18E-06 | 0.705 | FALSE | 21 |
|  | rs12669698 | C | T | 1.127 | 0.033 | 0.255 | 0.024 | 9.73E-06 | 0.173 | FALSE | 20 |
|  | rs17145573 | A | G | 1.708 | 0.015 | 0.365 | 0.024 | 2.87E-06 | 0.534 | FALSE | 22 |
|  | rs389558 | T | C | -0.783 | -0.005 | 0.160 | 0.015 | 9.74E-07 | 0.741 | FALSE | 24 |
|  | rs491806 | C | A | -0.948 | 0.008 | 0.211 | 0.018 | 6.92E-06 | 0.661 | FALSE | 20 |
|  | rs529324 | A | G | -0.826 | -0.022 | 0.179 | 0.016 | 4.16E-06 | 0.179 | FALSE | 21 |
|  | rs7778345 | G | A | 0.730 | 0.004 | 0.163 | 0.015 | 7.05E-06 | 0.804 | FALSE | 20 |
| genus.Tyzzerella3(id.11335) | rs10513202 | G | A | 1.427 | 0.034 | 0.321 | 0.032 | 8.58E-06 | 0.287 | FALSE | 20 |
|  | rs11090513 | T | G | 0.798 | -0.041 | 0.175 | 0.023 | 5.00E-06 | 0.080 | FALSE | 21 |
|  | rs11230859 | A | G | -0.725 | -0.023 | 0.158 | 0.020 | 4.18E-06 | 0.243 | FALSE | 21 |
|  | rs12669698 | C | T | 1.127 | 0.010 | 0.255 | 0.031 | 9.73E-06 | 0.751 | FALSE | 20 |
|  | rs17145573 | A | G | 1.708 | 0.025 | 0.365 | 0.031 | 2.87E-06 | 0.424 | FALSE | 22 |
|  | rs389558 | T | C | -0.783 | 0.032 | 0.160 | 0.020 | 9.74E-07 | 0.108 | FALSE | 24 |
|  | rs491806 | C | A | -0.948 | 0.024 | 0.211 | 0.024 | 6.92E-06 | 0.315 | FALSE | 20 |
|  | rs529324 | A | G | -0.826 | -0.007 | 0.179 | 0.021 | 4.16E-06 | 0.745 | FALSE | 21 |
|  | rs7778345 | G | A | 0.730 | 0.013 | 0.163 | 0.020 | 7.05E-06 | 0.514 | FALSE | 20 |
| genus.unknowngenus(id.826) | rs10513202 | G | A | 1.427 | -0.003 | 0.321 | 0.020 | 8.58E-06 | 0.865 | FALSE | 20 |
|  | rs11090513 | T | G | 0.798 | -0.024 | 0.175 | 0.014 | 5.00E-06 | 0.094 | FALSE | 21 |
|  | rs11230859 | A | G | -0.725 | -0.024 | 0.158 | 0.013 | 4.18E-06 | 0.064 | FALSE | 21 |
|  | rs12669698 | C | T | 1.127 | 0.026 | 0.255 | 0.020 | 9.73E-06 | 0.186 | FALSE | 20 |
|  | rs17145573 | A | G | 1.708 | -0.003 | 0.365 | 0.020 | 2.87E-06 | 0.877 | FALSE | 22 |
|  | rs389558 | T | C | -0.783 | -0.005 | 0.160 | 0.012 | 9.74E-07 | 0.695 | FALSE | 24 |
|  | rs491806 | C | A | -0.948 | 0.016 | 0.211 | 0.015 | 6.92E-06 | 0.286 | FALSE | 20 |
|  | rs529324 | A | G | -0.826 | -0.018 | 0.179 | 0.013 | 4.16E-06 | 0.167 | FALSE | 21 |
|  | rs7778345 | G | A | 0.730 | -0.010 | 0.163 | 0.013 | 7.05E-06 | 0.405 | FALSE | 20 |
| genus.unknowngenus(id.959) | rs10513202 | G | A | 1.427 | -0.030 | 0.321 | 0.033 | 8.58E-06 | 0.366 | FALSE | 20 |
|  | rs11090513 | T | G | 0.798 | 0.010 | 0.175 | 0.025 | 5.00E-06 | 0.692 | FALSE | 21 |
|  | rs11230859 | A | G | -0.725 | -0.009 | 0.158 | 0.021 | 4.18E-06 | 0.688 | FALSE | 21 |
|  | rs12669698 | C | T | 1.127 | -0.002 | 0.255 | 0.031 | 9.73E-06 | 0.957 | FALSE | 20 |
|  | rs17145573 | A | G | 1.708 | -0.037 | 0.365 | 0.032 | 2.87E-06 | 0.244 | FALSE | 22 |
|  | rs389558 | T | C | -0.783 | -0.027 | 0.160 | 0.021 | 9.74E-07 | 0.197 | FALSE | 24 |
|  | rs491806 | C | A | -0.948 | -0.022 | 0.211 | 0.025 | 6.92E-06 | 0.378 | FALSE | 20 |
|  | rs529324 | A | G | -0.826 | 0.042 | 0.179 | 0.022 | 4.16E-06 | 0.052 | FALSE | 21 |
|  | rs7778345 | G | A | 0.730 | 0.001 | 0.163 | 0.021 | 7.05E-06 | 0.977 | FALSE | 20 |
| family.Acidaminococcaceae(id.2166) | rs10513202 | G | A | 1.427 | 0.027 | 0.321 | 0.020 | 8.58E-06 | 0.183 | FALSE | 20 |
|  | rs11090513 | T | G | 0.798 | -0.003 | 0.175 | 0.015 | 5.00E-06 | 0.857 | FALSE | 21 |
|  | rs11230859 | A | G | -0.725 | 0.000 | 0.158 | 0.013 | 4.18E-06 | 0.978 | FALSE | 21 |
|  | rs12669698 | C | T | 1.127 | 0.014 | 0.255 | 0.020 | 9.73E-06 | 0.461 | FALSE | 20 |
|  | rs17145573 | A | G | 1.708 | -0.035 | 0.365 | 0.020 | 2.87E-06 | 0.086 | FALSE | 22 |
|  | rs389558 | T | C | -0.783 | -0.008 | 0.160 | 0.013 | 9.74E-07 | 0.562 | FALSE | 24 |
|  | rs491806 | C | A | -0.948 | -0.019 | 0.211 | 0.016 | 6.92E-06 | 0.218 | FALSE | 20 |
|  | rs529324 | A | G | -0.826 | 0.004 | 0.179 | 0.014 | 4.16E-06 | 0.778 | FALSE | 21 |
|  | rs7778345 | G | A | 0.730 | 0.008 | 0.163 | 0.013 | 7.05E-06 | 0.527 | FALSE | 20 |
| family.Actinomycetaceae(id.421) | rs10513202 | G | A | 1.427 | 0.002 | 0.321 | 0.026 | 8.58E-06 | 0.926 | FALSE | 20 |
|  | rs11090513 | T | G | 0.798 | -0.032 | 0.175 | 0.019 | 5.00E-06 | 0.088 | FALSE | 21 |
|  | rs11230859 | A | G | -0.725 | -0.008 | 0.158 | 0.017 | 4.18E-06 | 0.625 | FALSE | 21 |
|  | rs12669698 | C | T | 1.127 | 0.017 | 0.255 | 0.026 | 9.73E-06 | 0.521 | FALSE | 20 |
|  | rs17145573 | A | G | 1.708 | -0.017 | 0.365 | 0.026 | 2.87E-06 | 0.516 | FALSE | 22 |
|  | rs389558 | T | C | -0.783 | -0.001 | 0.160 | 0.016 | 9.74E-07 | 0.931 | FALSE | 24 |
|  | rs491806 | C | A | -0.948 | -0.008 | 0.211 | 0.020 | 6.92E-06 | 0.683 | FALSE | 20 |
|  | rs529324 | A | G | -0.826 | -0.017 | 0.179 | 0.017 | 4.16E-06 | 0.338 | FALSE | 21 |
|  | rs7778345 | G | A | 0.730 | -0.018 | 0.163 | 0.016 | 7.05E-06 | 0.262 | FALSE | 20 |
| family.Alcaligenaceae(id.2875) | rs10513202 | G | A | 1.427 | 0.016 | 0.321 | 0.018 | 8.58E-06 | 0.371 | FALSE | 20 |
|  | rs11090513 | T | G | 0.798 | -0.011 | 0.175 | 0.013 | 5.00E-06 | 0.397 | FALSE | 21 |
|  | rs11230859 | A | G | -0.725 | 0.004 | 0.158 | 0.011 | 4.18E-06 | 0.735 | FALSE | 21 |
|  | rs12669698 | C | T | 1.127 | 0.021 | 0.255 | 0.017 | 9.73E-06 | 0.216 | FALSE | 20 |
|  | rs17145573 | A | G | 1.708 | 0.004 | 0.365 | 0.017 | 2.87E-06 | 0.824 | FALSE | 22 |
|  | rs389558 | T | C | -0.783 | -0.014 | 0.160 | 0.011 | 9.74E-07 | 0.196 | FALSE | 24 |
|  | rs491806 | C | A | -0.948 | -0.014 | 0.211 | 0.013 | 6.92E-06 | 0.300 | FALSE | 20 |
|  | rs529324 | A | G | -0.826 | -0.004 | 0.179 | 0.012 | 4.16E-06 | 0.711 | FALSE | 21 |
|  | rs7778345 | G | A | 0.730 | -0.005 | 0.163 | 0.011 | 7.05E-06 | 0.651 | FALSE | 20 |
| family.Bacteroidaceae(id.917) | rs10513202 | G | A | 1.427 | -0.013 | 0.321 | 0.017 | 8.58E-06 | 0.424 | FALSE | 20 |
|  | rs11090513 | T | G | 0.798 | -0.013 | 0.175 | 0.012 | 5.00E-06 | 0.310 | FALSE | 21 |
|  | rs11230859 | A | G | -0.725 | -0.009 | 0.158 | 0.011 | 4.18E-06 | 0.391 | FALSE | 21 |
|  | rs12669698 | C | T | 1.127 | 0.009 | 0.255 | 0.016 | 9.73E-06 | 0.570 | FALSE | 20 |
|  | rs17145573 | A | G | 1.708 | -0.020 | 0.365 | 0.017 | 2.87E-06 | 0.244 | FALSE | 22 |
|  | rs389558 | T | C | -0.783 | -0.004 | 0.160 | 0.011 | 9.74E-07 | 0.743 | FALSE | 24 |
|  | rs491806 | C | A | -0.948 | -0.023 | 0.211 | 0.013 | 6.92E-06 | 0.076 | FALSE | 20 |
|  | rs529324 | A | G | -0.826 | -0.012 | 0.179 | 0.011 | 4.16E-06 | 0.307 | FALSE | 21 |
|  | rs7778345 | G | A | 0.730 | -0.003 | 0.163 | 0.011 | 7.05E-06 | 0.768 | FALSE | 20 |
| family.BacteroidalesS24.7group(id.11173) | rs10513202 | G | A | 1.427 | -0.010 | 0.321 | 0.026 | 8.58E-06 | 0.699 | FALSE | 20 |
|  | rs11090513 | T | G | 0.798 | 0.007 | 0.175 | 0.019 | 5.00E-06 | 0.708 | FALSE | 21 |
|  | rs11230859 | A | G | -0.725 | 0.006 | 0.158 | 0.017 | 4.18E-06 | 0.729 | FALSE | 21 |
|  | rs12669698 | C | T | 1.127 | -0.010 | 0.255 | 0.025 | 9.73E-06 | 0.679 | FALSE | 20 |
|  | rs17145573 | A | G | 1.708 | -0.029 | 0.365 | 0.026 | 2.87E-06 | 0.261 | FALSE | 22 |
|  | rs389558 | T | C | -0.783 | -0.016 | 0.160 | 0.016 | 9.74E-07 | 0.313 | FALSE | 24 |
|  | rs491806 | C | A | -0.948 | 0.018 | 0.211 | 0.020 | 6.92E-06 | 0.364 | FALSE | 20 |
|  | rs529324 | A | G | -0.826 | 0.012 | 0.179 | 0.017 | 4.16E-06 | 0.478 | FALSE | 21 |
|  | rs7778345 | G | A | 0.730 | -0.004 | 0.163 | 0.016 | 7.05E-06 | 0.824 | FALSE | 20 |
| family.Bifidobacteriaceae(id.433) | rs10513202 | G | A | 1.427 | 0.005 | 0.321 | 0.019 | 8.58E-06 | 0.791 | FALSE | 20 |
|  | rs11090513 | T | G | 0.798 | -0.017 | 0.175 | 0.014 | 5.00E-06 | 0.205 | FALSE | 21 |
|  | rs11230859 | A | G | -0.725 | -0.005 | 0.158 | 0.012 | 4.18E-06 | 0.691 | FALSE | 21 |
|  | rs12669698 | C | T | 1.127 | -0.024 | 0.255 | 0.018 | 9.73E-06 | 0.175 | FALSE | 20 |
|  | rs17145573 | A | G | 1.708 | 0.033 | 0.365 | 0.018 | 2.87E-06 | 0.078 | FALSE | 22 |
|  | rs389558 | T | C | -0.783 | -0.003 | 0.160 | 0.012 | 9.74E-07 | 0.783 | FALSE | 24 |
|  | rs491806 | C | A | -0.948 | -0.007 | 0.211 | 0.014 | 6.92E-06 | 0.632 | FALSE | 20 |
|  | rs529324 | A | G | -0.826 | -0.031 | 0.179 | 0.013 | 4.16E-06 | 0.015 | FALSE | 21 |
|  | rs7778345 | G | A | 0.730 | -0.005 | 0.163 | 0.012 | 7.05E-06 | 0.703 | FALSE | 20 |
| family.Christensenellaceae(id.1866) | rs10513202 | G | A | 1.427 | -0.015 | 0.321 | 0.018 | 8.58E-06 | 0.408 | FALSE | 20 |
|  | rs11090513 | T | G | 0.798 | 0.011 | 0.175 | 0.013 | 5.00E-06 | 0.387 | FALSE | 21 |
|  | rs11230859 | A | G | -0.725 | 0.013 | 0.158 | 0.011 | 4.18E-06 | 0.248 | FALSE | 21 |
|  | rs12669698 | C | T | 1.127 | -0.003 | 0.255 | 0.017 | 9.73E-06 | 0.855 | FALSE | 20 |
|  | rs17145573 | A | G | 1.708 | -0.012 | 0.365 | 0.018 | 2.87E-06 | 0.500 | FALSE | 22 |
|  | rs389558 | T | C | -0.783 | -0.012 | 0.160 | 0.011 | 9.74E-07 | 0.292 | FALSE | 24 |
|  | rs491806 | C | A | -0.948 | 0.002 | 0.211 | 0.013 | 6.92E-06 | 0.908 | FALSE | 20 |
|  | rs529324 | A | G | -0.826 | 0.007 | 0.179 | 0.012 | 4.16E-06 | 0.530 | FALSE | 21 |
|  | rs7778345 | G | A | 0.730 | 0.007 | 0.163 | 0.011 | 7.05E-06 | 0.518 | FALSE | 20 |
| family.Clostridiaceae1(id.1869) | rs10513202 | G | A | 1.427 | -0.002 | 0.321 | 0.019 | 8.58E-06 | 0.925 | FALSE | 20 |
|  | rs11090513 | T | G | 0.798 | -0.006 | 0.175 | 0.014 | 5.00E-06 | 0.693 | FALSE | 21 |
|  | rs11230859 | A | G | -0.725 | -0.002 | 0.158 | 0.012 | 4.18E-06 | 0.858 | FALSE | 21 |
|  | rs12669698 | C | T | 1.127 | -0.011 | 0.255 | 0.019 | 9.73E-06 | 0.537 | FALSE | 20 |
|  | rs17145573 | A | G | 1.708 | -0.024 | 0.365 | 0.019 | 2.87E-06 | 0.198 | FALSE | 22 |
|  | rs389558 | T | C | -0.783 | 0.006 | 0.160 | 0.012 | 9.74E-07 | 0.594 | FALSE | 24 |
|  | rs491806 | C | A | -0.948 | 0.017 | 0.211 | 0.015 | 6.92E-06 | 0.241 | FALSE | 20 |
|  | rs529324 | A | G | -0.826 | -0.001 | 0.179 | 0.013 | 4.16E-06 | 0.965 | FALSE | 21 |
|  | rs7778345 | G | A | 0.730 | 0.014 | 0.163 | 0.012 | 7.05E-06 | 0.246 | FALSE | 20 |
| family.ClostridialesvadinBB60group(id.11286) | rs10513202 | G | A | 1.427 | 0.007 | 0.321 | 0.022 | 8.58E-06 | 0.754 | FALSE | 20 |
|  | rs11090513 | T | G | 0.798 | 0.043 | 0.175 | 0.016 | 5.00E-06 | 0.008 | FALSE | 21 |
|  | rs11230859 | A | G | -0.725 | -0.005 | 0.158 | 0.014 | 4.18E-06 | 0.739 | FALSE | 21 |
|  | rs12669698 | C | T | 1.127 | -0.003 | 0.255 | 0.022 | 9.73E-06 | 0.905 | FALSE | 20 |
|  | rs17145573 | A | G | 1.708 | -0.063 | 0.365 | 0.022 | 2.87E-06 | 0.005 | FALSE | 22 |
|  | rs389558 | T | C | -0.783 | 0.025 | 0.160 | 0.014 | 9.74E-07 | 0.081 | FALSE | 24 |
|  | rs491806 | C | A | -0.948 | 0.009 | 0.211 | 0.017 | 6.92E-06 | 0.612 | FALSE | 20 |
|  | rs529324 | A | G | -0.826 | -0.007 | 0.179 | 0.015 | 4.16E-06 | 0.649 | FALSE | 21 |
|  | rs7778345 | G | A | 0.730 | 0.020 | 0.163 | 0.014 | 7.05E-06 | 0.150 | FALSE | 20 |
| family.Coriobacteriaceae(id.811) | rs10513202 | G | A | 1.427 | -0.017 | 0.321 | 0.017 | 8.58E-06 | 0.334 | FALSE | 20 |
|  | rs11090513 | T | G | 0.798 | -0.017 | 0.175 | 0.012 | 5.00E-06 | 0.176 | FALSE | 21 |
|  | rs11230859 | A | G | -0.725 | -0.001 | 0.158 | 0.011 | 4.18E-06 | 0.916 | FALSE | 21 |
|  | rs12669698 | C | T | 1.127 | 0.014 | 0.255 | 0.017 | 9.73E-06 | 0.387 | FALSE | 20 |
|  | rs17145573 | A | G | 1.708 | 0.022 | 0.365 | 0.017 | 2.87E-06 | 0.190 | FALSE | 22 |
|  | rs389558 | T | C | -0.783 | 0.002 | 0.160 | 0.011 | 9.74E-07 | 0.878 | FALSE | 24 |
|  | rs491806 | C | A | -0.948 | 0.011 | 0.211 | 0.013 | 6.92E-06 | 0.417 | FALSE | 20 |
|  | rs529324 | A | G | -0.826 | 0.012 | 0.179 | 0.012 | 4.16E-06 | 0.300 | FALSE | 21 |
|  | rs7778345 | G | A | 0.730 | 0.002 | 0.163 | 0.011 | 7.05E-06 | 0.829 | FALSE | 20 |
| family.Defluviitaleaceae(id.1924) | rs10513202 | G | A | 1.427 | -0.039 | 0.321 | 0.025 | 8.58E-06 | 0.126 | FALSE | 20 |
|  | rs11090513 | T | G | 0.798 | 0.014 | 0.175 | 0.018 | 5.00E-06 | 0.436 | FALSE | 21 |
|  | rs11230859 | A | G | -0.725 | 0.045 | 0.158 | 0.016 | 4.18E-06 | 0.006 | FALSE | 21 |
|  | rs12669698 | C | T | 1.127 | -0.012 | 0.255 | 0.025 | 9.73E-06 | 0.630 | FALSE | 20 |
|  | rs17145573 | A | G | 1.708 | -0.016 | 0.365 | 0.026 | 2.87E-06 | 0.532 | FALSE | 22 |
|  | rs389558 | T | C | -0.783 | -0.020 | 0.160 | 0.016 | 9.74E-07 | 0.213 | FALSE | 24 |
|  | rs491806 | C | A | -0.948 | 0.034 | 0.211 | 0.019 | 6.92E-06 | 0.079 | FALSE | 20 |
|  | rs529324 | A | G | -0.826 | -0.034 | 0.179 | 0.017 | 4.16E-06 | 0.048 | FALSE | 21 |
|  | rs7778345 | G | A | 0.730 | -0.006 | 0.163 | 0.016 | 7.05E-06 | 0.683 | FALSE | 20 |
| family.Desulfovibrionaceae(id.3169) | rs10513202 | G | A | 1.427 | 0.018 | 0.321 | 0.019 | 8.58E-06 | 0.319 | FALSE | 20 |
|  | rs11090513 | T | G | 0.798 | -0.011 | 0.175 | 0.014 | 5.00E-06 | 0.411 | FALSE | 21 |
|  | rs11230859 | A | G | -0.725 | 0.011 | 0.158 | 0.012 | 4.18E-06 | 0.347 | FALSE | 21 |
|  | rs12669698 | C | T | 1.127 | 0.013 | 0.255 | 0.018 | 9.73E-06 | 0.466 | FALSE | 20 |
|  | rs17145573 | A | G | 1.708 | -0.016 | 0.365 | 0.018 | 2.87E-06 | 0.373 | FALSE | 22 |
|  | rs389558 | T | C | -0.783 | 0.008 | 0.160 | 0.012 | 9.74E-07 | 0.507 | FALSE | 24 |
|  | rs491806 | C | A | -0.948 | 0.016 | 0.211 | 0.014 | 6.92E-06 | 0.252 | FALSE | 20 |
|  | rs529324 | A | G | -0.826 | 0.004 | 0.179 | 0.012 | 4.16E-06 | 0.762 | FALSE | 21 |
|  | rs7778345 | G | A | 0.730 | 0.015 | 0.163 | 0.012 | 7.05E-06 | 0.204 | FALSE | 20 |
| family.Enterobacteriaceae(id.3469) | rs10513202 | G | A | 1.427 | 0.004 | 0.321 | 0.020 | 8.58E-06 | 0.820 | FALSE | 20 |
|  | rs11090513 | T | G | 0.798 | 0.005 | 0.175 | 0.014 | 5.00E-06 | 0.745 | FALSE | 21 |
|  | rs11230859 | A | G | -0.725 | 0.036 | 0.158 | 0.013 | 4.18E-06 | 0.004 | FALSE | 21 |
|  | rs12669698 | C | T | 1.127 | -0.004 | 0.255 | 0.019 | 9.73E-06 | 0.843 | FALSE | 20 |
|  | rs17145573 | A | G | 1.708 | 0.026 | 0.365 | 0.019 | 2.87E-06 | 0.179 | FALSE | 22 |
|  | rs389558 | T | C | -0.783 | 0.004 | 0.160 | 0.012 | 9.74E-07 | 0.749 | FALSE | 24 |
|  | rs491806 | C | A | -0.948 | -0.005 | 0.211 | 0.015 | 6.92E-06 | 0.755 | FALSE | 20 |
|  | rs529324 | A | G | -0.826 | 0.016 | 0.179 | 0.013 | 4.16E-06 | 0.215 | FALSE | 21 |
|  | rs7778345 | G | A | 0.730 | 0.014 | 0.163 | 0.013 | 7.05E-06 | 0.265 | FALSE | 20 |
| family.Erysipelotrichaceae(id.2149) | rs10513202 | G | A | 1.427 | -0.015 | 0.321 | 0.017 | 8.58E-06 | 0.389 | FALSE | 20 |
|  | rs11090513 | T | G | 0.798 | 0.001 | 0.175 | 0.012 | 5.00E-06 | 0.945 | FALSE | 21 |
|  | rs11230859 | A | G | -0.725 | -0.015 | 0.158 | 0.011 | 4.18E-06 | 0.168 | FALSE | 21 |
|  | rs12669698 | C | T | 1.127 | 0.016 | 0.255 | 0.016 | 9.73E-06 | 0.324 | FALSE | 20 |
|  | rs17145573 | A | G | 1.708 | 0.022 | 0.365 | 0.017 | 2.87E-06 | 0.193 | FALSE | 22 |
|  | rs389558 | T | C | -0.783 | 0.002 | 0.160 | 0.011 | 9.74E-07 | 0.866 | FALSE | 24 |
|  | rs491806 | C | A | -0.948 | -0.004 | 0.211 | 0.013 | 6.92E-06 | 0.773 | FALSE | 20 |
|  | rs529324 | A | G | -0.826 | -0.012 | 0.179 | 0.011 | 4.16E-06 | 0.277 | FALSE | 21 |
|  | rs7778345 | G | A | 0.730 | 0.009 | 0.163 | 0.011 | 7.05E-06 | 0.415 | FALSE | 20 |
| family.FamilyXI(id.1936) | rs10513202 | G | A | 1.427 | 0.003 | 0.321 | 0.040 | 8.58E-06 | 0.940 | FALSE | 20 |
|  | rs11230859 | A | G | -0.725 | 0.033 | 0.158 | 0.026 | 4.18E-06 | 0.217 | FALSE | 21 |
|  | rs12669698 | C | T | 1.127 | -0.010 | 0.255 | 0.040 | 9.73E-06 | 0.808 | FALSE | 20 |
|  | rs17145573 | A | G | 1.708 | 0.019 | 0.365 | 0.042 | 2.87E-06 | 0.657 | FALSE | 22 |
|  | rs491806 | C | A | -0.948 | -0.001 | 0.211 | 0.031 | 6.92E-06 | 0.980 | FALSE | 20 |
|  | rs529324 | A | G | -0.826 | 0.000 | 0.179 | 0.027 | 4.16E-06 | 0.994 | FALSE | 21 |
|  | rs7778345 | G | A | 0.730 | -0.006 | 0.163 | 0.025 | 7.05E-06 | 0.824 | FALSE | 20 |
| family.FamilyXIII(id.1957) | rs10513202 | G | A | 1.427 | -0.007 | 0.321 | 0.018 | 8.58E-06 | 0.689 | FALSE | 20 |
|  | rs11090513 | T | G | 0.798 | 0.018 | 0.175 | 0.013 | 5.00E-06 | 0.155 | FALSE | 21 |
|  | rs11230859 | A | G | -0.725 | 0.006 | 0.158 | 0.011 | 4.18E-06 | 0.611 | FALSE | 21 |
|  | rs12669698 | C | T | 1.127 | -0.016 | 0.255 | 0.017 | 9.73E-06 | 0.344 | FALSE | 20 |
|  | rs17145573 | A | G | 1.708 | 0.021 | 0.365 | 0.018 | 2.87E-06 | 0.231 | FALSE | 22 |
|  | rs389558 | T | C | -0.783 | -0.002 | 0.160 | 0.011 | 9.74E-07 | 0.892 | FALSE | 24 |
|  | rs491806 | C | A | -0.948 | 0.029 | 0.211 | 0.013 | 6.92E-06 | 0.030 | FALSE | 20 |
|  | rs529324 | A | G | -0.826 | 0.018 | 0.179 | 0.012 | 4.16E-06 | 0.130 | FALSE | 21 |
|  | rs7778345 | G | A | 0.730 | 0.015 | 0.163 | 0.011 | 7.05E-06 | 0.193 | FALSE | 20 |
| family.Lachnospiraceae(id.1987) | rs10513202 | G | A | 1.427 | -0.003 | 0.321 | 0.017 | 8.58E-06 | 0.860 | FALSE | 20 |
|  | rs11090513 | T | G | 0.798 | -0.009 | 0.175 | 0.012 | 5.00E-06 | 0.469 | FALSE | 21 |
|  | rs11230859 | A | G | -0.725 | -0.010 | 0.158 | 0.011 | 4.18E-06 | 0.344 | FALSE | 21 |
|  | rs12669698 | C | T | 1.127 | 0.001 | 0.255 | 0.016 | 9.73E-06 | 0.953 | FALSE | 20 |
|  | rs17145573 | A | G | 1.708 | 0.002 | 0.365 | 0.017 | 2.87E-06 | 0.910 | FALSE | 22 |
|  | rs389558 | T | C | -0.783 | 0.002 | 0.160 | 0.011 | 9.74E-07 | 0.877 | FALSE | 24 |
|  | rs491806 | C | A | -0.948 | 0.004 | 0.211 | 0.013 | 6.92E-06 | 0.781 | FALSE | 20 |
|  | rs529324 | A | G | -0.826 | 0.005 | 0.179 | 0.011 | 4.16E-06 | 0.684 | FALSE | 21 |
|  | rs7778345 | G | A | 0.730 | 0.003 | 0.163 | 0.011 | 7.05E-06 | 0.773 | FALSE | 20 |
| family.Lactobacillaceae(id.1836) | rs10513202 | G | A | 1.427 | 0.048 | 0.321 | 0.027 | 8.58E-06 | 0.081 | FALSE | 20 |
|  | rs11090513 | T | G | 0.798 | -0.005 | 0.175 | 0.020 | 5.00E-06 | 0.809 | FALSE | 21 |
|  | rs11230859 | A | G | -0.725 | -0.006 | 0.158 | 0.018 | 4.18E-06 | 0.713 | FALSE | 21 |
|  | rs12669698 | C | T | 1.127 | 0.004 | 0.255 | 0.027 | 9.73E-06 | 0.871 | FALSE | 20 |
|  | rs17145573 | A | G | 1.708 | 0.033 | 0.365 | 0.027 | 2.87E-06 | 0.222 | FALSE | 22 |
|  | rs389558 | T | C | -0.783 | 0.006 | 0.160 | 0.017 | 9.74E-07 | 0.727 | FALSE | 24 |
|  | rs491806 | C | A | -0.948 | -0.016 | 0.211 | 0.021 | 6.92E-06 | 0.434 | FALSE | 20 |
|  | rs529324 | A | G | -0.826 | 0.006 | 0.179 | 0.018 | 4.16E-06 | 0.734 | FALSE | 21 |
|  | rs7778345 | G | A | 0.730 | -0.009 | 0.163 | 0.017 | 7.05E-06 | 0.605 | FALSE | 20 |
| family.Methanobacteriaceae(id.121) | rs10513202 | G | A | 1.427 | 0.025 | 0.321 | 0.037 | 8.58E-06 | 0.491 | FALSE | 20 |
|  | rs11090513 | T | G | 0.798 | -0.006 | 0.175 | 0.029 | 5.00E-06 | 0.832 | FALSE | 21 |
|  | rs11230859 | A | G | -0.725 | 0.005 | 0.158 | 0.024 | 4.18E-06 | 0.849 | FALSE | 21 |
|  | rs12669698 | C | T | 1.127 | -0.032 | 0.255 | 0.037 | 9.73E-06 | 0.386 | FALSE | 20 |
|  | rs17145573 | A | G | 1.708 | -0.040 | 0.365 | 0.038 | 2.87E-06 | 0.286 | FALSE | 22 |
|  | rs389558 | T | C | -0.783 | 0.029 | 0.160 | 0.025 | 9.74E-07 | 0.245 | FALSE | 24 |
|  | rs491806 | C | A | -0.948 | 0.023 | 0.211 | 0.029 | 6.92E-06 | 0.429 | FALSE | 20 |
|  | rs529324 | A | G | -0.826 | -0.001 | 0.179 | 0.025 | 4.16E-06 | 0.983 | FALSE | 21 |
|  | rs7778345 | G | A | 0.730 | -0.030 | 0.163 | 0.024 | 7.05E-06 | 0.203 | FALSE | 20 |
| family.Oxalobacteraceae(id.2966) | rs10513202 | G | A | 1.427 | 0.023 | 0.321 | 0.032 | 8.58E-06 | 0.482 | FALSE | 20 |
|  | rs11090513 | T | G | 0.798 | 0.050 | 0.175 | 0.023 | 5.00E-06 | 0.031 | FALSE | 21 |
|  | rs11230859 | A | G | -0.725 | 0.004 | 0.158 | 0.021 | 4.18E-06 | 0.846 | FALSE | 21 |
|  | rs12669698 | C | T | 1.127 | -0.027 | 0.255 | 0.032 | 9.73E-06 | 0.389 | FALSE | 20 |
|  | rs17145573 | A | G | 1.708 | 0.031 | 0.365 | 0.032 | 2.87E-06 | 0.338 | FALSE | 22 |
|  | rs389558 | T | C | -0.783 | 0.015 | 0.160 | 0.020 | 9.74E-07 | 0.466 | FALSE | 24 |
|  | rs491806 | C | A | -0.948 | 0.028 | 0.211 | 0.024 | 6.92E-06 | 0.246 | FALSE | 20 |
|  | rs529324 | A | G | -0.826 | 0.025 | 0.179 | 0.021 | 4.16E-06 | 0.233 | FALSE | 21 |
|  | rs7778345 | G | A | 0.730 | 0.010 | 0.163 | 0.020 | 7.05E-06 | 0.608 | FALSE | 20 |
| family.Pasteurellaceae(id.3689) | rs10513202 | G | A | 1.427 | 0.014 | 0.321 | 0.024 | 8.58E-06 | 0.541 | FALSE | 20 |
|  | rs11090513 | T | G | 0.798 | 0.001 | 0.175 | 0.017 | 5.00E-06 | 0.972 | FALSE | 21 |
|  | rs11230859 | A | G | -0.725 | 0.000 | 0.158 | 0.015 | 4.18E-06 | 0.978 | FALSE | 21 |
|  | rs12669698 | C | T | 1.127 | 0.020 | 0.255 | 0.022 | 9.73E-06 | 0.372 | FALSE | 20 |
|  | rs17145573 | A | G | 1.708 | -0.009 | 0.365 | 0.023 | 2.87E-06 | 0.715 | FALSE | 22 |
|  | rs389558 | T | C | -0.783 | -0.025 | 0.160 | 0.015 | 9.74E-07 | 0.094 | FALSE | 24 |
|  | rs491806 | C | A | -0.948 | 0.017 | 0.211 | 0.018 | 6.92E-06 | 0.340 | FALSE | 20 |
|  | rs529324 | A | G | -0.826 | 0.020 | 0.179 | 0.016 | 4.16E-06 | 0.212 | FALSE | 21 |
|  | rs7778345 | G | A | 0.730 | -0.004 | 0.163 | 0.015 | 7.05E-06 | 0.786 | FALSE | 20 |
| family.Peptococcaceae(id.2024) | rs10513202 | G | A | 1.427 | -0.032 | 0.321 | 0.022 | 8.58E-06 | 0.144 | FALSE | 20 |
|  | rs11090513 | T | G | 0.798 | 0.017 | 0.175 | 0.016 | 5.00E-06 | 0.278 | FALSE | 21 |
|  | rs11230859 | A | G | -0.725 | -0.020 | 0.158 | 0.014 | 4.18E-06 | 0.161 | FALSE | 21 |
|  | rs12669698 | C | T | 1.127 | -0.002 | 0.255 | 0.022 | 9.73E-06 | 0.932 | FALSE | 20 |
|  | rs17145573 | A | G | 1.708 | -0.029 | 0.365 | 0.022 | 2.87E-06 | 0.187 | FALSE | 22 |
|  | rs389558 | T | C | -0.783 | -0.001 | 0.160 | 0.014 | 9.74E-07 | 0.937 | FALSE | 24 |
|  | rs491806 | C | A | -0.948 | -0.012 | 0.211 | 0.017 | 6.92E-06 | 0.462 | FALSE | 20 |
|  | rs529324 | A | G | -0.826 | -0.015 | 0.179 | 0.015 | 4.16E-06 | 0.299 | FALSE | 21 |
|  | rs7778345 | G | A | 0.730 | -0.005 | 0.163 | 0.014 | 7.05E-06 | 0.719 | FALSE | 20 |
| family.Peptostreptococcaceae(id.2042) | rs10513202 | G | A | 1.427 | 0.002 | 0.321 | 0.018 | 8.58E-06 | 0.893 | FALSE | 20 |
|  | rs11090513 | T | G | 0.798 | -0.014 | 0.175 | 0.013 | 5.00E-06 | 0.294 | FALSE | 21 |
|  | rs11230859 | A | G | -0.725 | -0.004 | 0.158 | 0.012 | 4.18E-06 | 0.751 | FALSE | 21 |
|  | rs12669698 | C | T | 1.127 | 0.012 | 0.255 | 0.017 | 9.73E-06 | 0.488 | FALSE | 20 |
|  | rs17145573 | A | G | 1.708 | 0.013 | 0.365 | 0.018 | 2.87E-06 | 0.454 | FALSE | 22 |
|  | rs389558 | T | C | -0.783 | 0.017 | 0.160 | 0.011 | 9.74E-07 | 0.140 | FALSE | 24 |
|  | rs491806 | C | A | -0.948 | 0.001 | 0.211 | 0.014 | 6.92E-06 | 0.956 | FALSE | 20 |
|  | rs529324 | A | G | -0.826 | 0.009 | 0.179 | 0.012 | 4.16E-06 | 0.453 | FALSE | 21 |
|  | rs7778345 | G | A | 0.730 | 0.019 | 0.163 | 0.011 | 7.05E-06 | 0.095 | FALSE | 20 |
| family.Porphyromonadaceae(id.943) | rs10513202 | G | A | 1.427 | -0.007 | 0.321 | 0.017 | 8.58E-06 | 0.683 | FALSE | 20 |
|  | rs11090513 | T | G | 0.798 | 0.001 | 0.175 | 0.012 | 5.00E-06 | 0.944 | FALSE | 21 |
|  | rs11230859 | A | G | -0.725 | 0.001 | 0.158 | 0.011 | 4.18E-06 | 0.947 | FALSE | 21 |
|  | rs12669698 | C | T | 1.127 | 0.016 | 0.255 | 0.016 | 9.73E-06 | 0.343 | FALSE | 20 |
|  | rs17145573 | A | G | 1.708 | 0.007 | 0.365 | 0.017 | 2.87E-06 | 0.662 | FALSE | 22 |
|  | rs389558 | T | C | -0.783 | 0.008 | 0.160 | 0.011 | 9.74E-07 | 0.447 | FALSE | 24 |
|  | rs491806 | C | A | -0.948 | -0.001 | 0.211 | 0.013 | 6.92E-06 | 0.917 | FALSE | 20 |
|  | rs529324 | A | G | -0.826 | 0.005 | 0.179 | 0.011 | 4.16E-06 | 0.669 | FALSE | 21 |
|  | rs7778345 | G | A | 0.730 | -0.007 | 0.163 | 0.011 | 7.05E-06 | 0.526 | FALSE | 20 |
| family.Prevotellaceae(id.960) | rs10513202 | G | A | 1.427 | 0.012 | 0.321 | 0.019 | 8.58E-06 | 0.532 | FALSE | 20 |
|  | rs11090513 | T | G | 0.798 | 0.009 | 0.175 | 0.014 | 5.00E-06 | 0.510 | FALSE | 21 |
|  | rs11230859 | A | G | -0.725 | -0.016 | 0.158 | 0.012 | 4.18E-06 | 0.192 | FALSE | 21 |
|  | rs12669698 | C | T | 1.127 | -0.023 | 0.255 | 0.018 | 9.73E-06 | 0.195 | FALSE | 20 |
|  | rs17145573 | A | G | 1.708 | -0.011 | 0.365 | 0.019 | 2.87E-06 | 0.560 | FALSE | 22 |
|  | rs389558 | T | C | -0.783 | 0.007 | 0.160 | 0.012 | 9.74E-07 | 0.539 | FALSE | 24 |
|  | rs491806 | C | A | -0.948 | 0.003 | 0.211 | 0.014 | 6.92E-06 | 0.833 | FALSE | 20 |
|  | rs529324 | A | G | -0.826 | 0.015 | 0.179 | 0.013 | 4.16E-06 | 0.224 | FALSE | 21 |
|  | rs7778345 | G | A | 0.730 | 0.008 | 0.163 | 0.012 | 7.05E-06 | 0.530 | FALSE | 20 |
| family.Rhodospirillaceae(id.2717) | rs10513202 | G | A | 1.427 | 0.024 | 0.321 | 0.025 | 8.58E-06 | 0.322 | FALSE | 20 |
|  | rs11090513 | T | G | 0.798 | -0.010 | 0.175 | 0.018 | 5.00E-06 | 0.588 | FALSE | 21 |
|  | rs11230859 | A | G | -0.725 | 0.002 | 0.158 | 0.016 | 4.18E-06 | 0.890 | FALSE | 21 |
|  | rs12669698 | C | T | 1.127 | 0.030 | 0.255 | 0.025 | 9.73E-06 | 0.219 | FALSE | 20 |
|  | rs17145573 | A | G | 1.708 | 0.011 | 0.365 | 0.025 | 2.87E-06 | 0.667 | FALSE | 22 |
|  | rs389558 | T | C | -0.783 | -0.006 | 0.160 | 0.016 | 9.74E-07 | 0.725 | FALSE | 24 |
|  | rs491806 | C | A | -0.948 | 0.000 | 0.211 | 0.019 | 6.92E-06 | 0.997 | FALSE | 20 |
|  | rs529324 | A | G | -0.826 | 0.007 | 0.179 | 0.017 | 4.16E-06 | 0.673 | FALSE | 21 |
|  | rs7778345 | G | A | 0.730 | -0.007 | 0.163 | 0.015 | 7.05E-06 | 0.654 | FALSE | 20 |
| family.Rikenellaceae(id.967) | rs10513202 | G | A | 1.427 | -0.011 | 0.321 | 0.017 | 8.58E-06 | 0.507 | FALSE | 20 |
|  | rs11090513 | T | G | 0.798 | -0.002 | 0.175 | 0.013 | 5.00E-06 | 0.882 | FALSE | 21 |
|  | rs11230859 | A | G | -0.725 | -0.007 | 0.158 | 0.011 | 4.18E-06 | 0.529 | FALSE | 21 |
|  | rs12669698 | C | T | 1.127 | 0.014 | 0.255 | 0.017 | 9.73E-06 | 0.390 | FALSE | 20 |
|  | rs17145573 | A | G | 1.708 | -0.021 | 0.365 | 0.017 | 2.87E-06 | 0.214 | FALSE | 22 |
|  | rs389558 | T | C | -0.783 | 0.003 | 0.160 | 0.011 | 9.74E-07 | 0.788 | FALSE | 24 |
|  | rs491806 | C | A | -0.948 | 0.000 | 0.211 | 0.013 | 6.92E-06 | 0.984 | FALSE | 20 |
|  | rs529324 | A | G | -0.826 | 0.009 | 0.179 | 0.012 | 4.16E-06 | 0.449 | FALSE | 21 |
|  | rs7778345 | G | A | 0.730 | -0.007 | 0.163 | 0.011 | 7.05E-06 | 0.506 | FALSE | 20 |
| family.Ruminococcaceae(id.2050) | rs10513202 | G | A | 1.427 | -0.017 | 0.321 | 0.017 | 8.58E-06 | 0.321 | FALSE | 20 |
|  | rs11090513 | T | G | 0.798 | 0.007 | 0.175 | 0.012 | 5.00E-06 | 0.593 | FALSE | 21 |
|  | rs11230859 | A | G | -0.725 | -0.004 | 0.158 | 0.011 | 4.18E-06 | 0.715 | FALSE | 21 |
|  | rs12669698 | C | T | 1.127 | -0.017 | 0.255 | 0.016 | 9.73E-06 | 0.281 | FALSE | 20 |
|  | rs17145573 | A | G | 1.708 | -0.005 | 0.365 | 0.017 | 2.87E-06 | 0.767 | FALSE | 22 |
|  | rs389558 | T | C | -0.783 | -0.003 | 0.160 | 0.011 | 9.74E-07 | 0.787 | FALSE | 24 |
|  | rs491806 | C | A | -0.948 | 0.011 | 0.211 | 0.013 | 6.92E-06 | 0.399 | FALSE | 20 |
|  | rs529324 | A | G | -0.826 | -0.002 | 0.179 | 0.011 | 4.16E-06 | 0.857 | FALSE | 21 |
|  | rs7778345 | G | A | 0.730 | 0.000 | 0.163 | 0.011 | 7.05E-06 | 1.000 | FALSE | 20 |
| family.Streptococcaceae(id.1850) | rs10513202 | G | A | 1.427 | -0.034 | 0.321 | 0.018 | 8.58E-06 | 0.052 | FALSE | 20 |
|  | rs11090513 | T | G | 0.798 | 0.018 | 0.175 | 0.013 | 5.00E-06 | 0.165 | FALSE | 21 |
|  | rs11230859 | A | G | -0.725 | 0.003 | 0.158 | 0.011 | 4.18E-06 | 0.822 | FALSE | 21 |
|  | rs12669698 | C | T | 1.127 | 0.030 | 0.255 | 0.017 | 9.73E-06 | 0.084 | FALSE | 20 |
|  | rs17145573 | A | G | 1.708 | 0.003 | 0.365 | 0.018 | 2.87E-06 | 0.864 | FALSE | 22 |
|  | rs389558 | T | C | -0.783 | -0.021 | 0.160 | 0.011 | 9.74E-07 | 0.060 | FALSE | 24 |
|  | rs491806 | C | A | -0.948 | 0.011 | 0.211 | 0.013 | 6.92E-06 | 0.433 | FALSE | 20 |
|  | rs529324 | A | G | -0.826 | 0.009 | 0.179 | 0.012 | 4.16E-06 | 0.452 | FALSE | 21 |
|  | rs7778345 | G | A | 0.730 | 0.002 | 0.163 | 0.011 | 7.05E-06 | 0.885 | FALSE | 20 |
| family.unknownfamily(id.1000001214) | rs10513202 | G | A | 1.427 | 0.037 | 0.321 | 0.030 | 8.58E-06 | 0.207 | FALSE | 20 |
|  | rs11090513 | T | G | 0.798 | -0.013 | 0.175 | 0.022 | 5.00E-06 | 0.558 | FALSE | 21 |
|  | rs11230859 | A | G | -0.725 | 0.017 | 0.158 | 0.019 | 4.18E-06 | 0.382 | FALSE | 21 |
|  | rs12669698 | C | T | 1.127 | 0.002 | 0.255 | 0.030 | 9.73E-06 | 0.946 | FALSE | 20 |
|  | rs17145573 | A | G | 1.708 | 0.008 | 0.365 | 0.030 | 2.87E-06 | 0.787 | FALSE | 22 |
|  | rs389558 | T | C | -0.783 | 0.006 | 0.160 | 0.019 | 9.74E-07 | 0.761 | FALSE | 24 |
|  | rs491806 | C | A | -0.948 | 0.013 | 0.211 | 0.022 | 6.92E-06 | 0.573 | FALSE | 20 |
|  | rs529324 | A | G | -0.826 | 0.005 | 0.179 | 0.020 | 4.16E-06 | 0.794 | FALSE | 21 |
|  | rs7778345 | G | A | 0.730 | -0.013 | 0.163 | 0.019 | 7.05E-06 | 0.498 | FALSE | 20 |
| family.unknownfamily(id.1000005471) | rs10513202 | G | A | 1.427 | 0.017 | 0.321 | 0.022 | 8.58E-06 | 0.451 | FALSE | 20 |
|  | rs11090513 | T | G | 0.798 | 0.012 | 0.175 | 0.016 | 5.00E-06 | 0.463 | FALSE | 21 |
|  | rs11230859 | A | G | -0.725 | -0.006 | 0.158 | 0.015 | 4.18E-06 | 0.685 | FALSE | 21 |
|  | rs12669698 | C | T | 1.127 | 0.000 | 0.255 | 0.022 | 9.73E-06 | 0.997 | FALSE | 20 |
|  | rs17145573 | A | G | 1.708 | 0.004 | 0.365 | 0.022 | 2.87E-06 | 0.857 | FALSE | 22 |
|  | rs389558 | T | C | -0.783 | -0.005 | 0.160 | 0.014 | 9.74E-07 | 0.702 | FALSE | 24 |
|  | rs491806 | C | A | -0.948 | 0.013 | 0.211 | 0.017 | 6.92E-06 | 0.435 | FALSE | 20 |
|  | rs529324 | A | G | -0.826 | 0.015 | 0.179 | 0.015 | 4.16E-06 | 0.308 | FALSE | 21 |
|  | rs7778345 | G | A | 0.730 | 0.017 | 0.163 | 0.014 | 7.05E-06 | 0.226 | FALSE | 20 |
| family.unknownfamily(id.1000006161) | rs10513202 | G | A | 1.427 | 0.046 | 0.321 | 0.032 | 8.58E-06 | 0.146 | FALSE | 20 |
|  | rs11090513 | T | G | 0.798 | 0.038 | 0.175 | 0.023 | 5.00E-06 | 0.103 | FALSE | 21 |
|  | rs11230859 | A | G | -0.725 | 0.005 | 0.158 | 0.021 | 4.18E-06 | 0.818 | FALSE | 21 |
|  | rs12669698 | C | T | 1.127 | -0.038 | 0.255 | 0.032 | 9.73E-06 | 0.229 | FALSE | 20 |
|  | rs17145573 | A | G | 1.708 | 0.022 | 0.365 | 0.033 | 2.87E-06 | 0.506 | FALSE | 22 |
|  | rs389558 | T | C | -0.783 | 0.008 | 0.160 | 0.021 | 9.74E-07 | 0.701 | FALSE | 24 |
|  | rs491806 | C | A | -0.948 | -0.025 | 0.211 | 0.024 | 6.92E-06 | 0.292 | FALSE | 20 |
|  | rs529324 | A | G | -0.826 | 0.016 | 0.179 | 0.022 | 4.16E-06 | 0.469 | FALSE | 21 |
|  | rs7778345 | G | A | 0.730 | 0.025 | 0.163 | 0.020 | 7.05E-06 | 0.219 | FALSE | 20 |
| family.Veillonellaceae(id.2172) | rs10513202 | G | A | 1.427 | 0.003 | 0.321 | 0.018 | 8.58E-06 | 0.871 | FALSE | 20 |
|  | rs11090513 | T | G | 0.798 | -0.016 | 0.175 | 0.013 | 5.00E-06 | 0.216 | FALSE | 21 |
|  | rs11230859 | A | G | -0.725 | 0.009 | 0.158 | 0.012 | 4.18E-06 | 0.451 | FALSE | 21 |
|  | rs12669698 | C | T | 1.127 | -0.033 | 0.255 | 0.017 | 9.73E-06 | 0.055 | FALSE | 20 |
|  | rs17145573 | A | G | 1.708 | -0.010 | 0.365 | 0.018 | 2.87E-06 | 0.578 | FALSE | 22 |
|  | rs389558 | T | C | -0.783 | 0.024 | 0.160 | 0.011 | 9.74E-07 | 0.034 | FALSE | 24 |
|  | rs491806 | C | A | -0.948 | 0.011 | 0.211 | 0.014 | 6.92E-06 | 0.420 | FALSE | 20 |
|  | rs529324 | A | G | -0.826 | 0.007 | 0.179 | 0.012 | 4.16E-06 | 0.574 | FALSE | 21 |
|  | rs7778345 | G | A | 0.730 | -0.004 | 0.163 | 0.012 | 7.05E-06 | 0.740 | FALSE | 20 |
| family.Verrucomicrobiaceae(id.4036) | rs10513202 | G | A | 1.427 | -0.016 | 0.321 | 0.021 | 8.58E-06 | 0.449 | FALSE | 20 |
|  | rs11090513 | T | G | 0.798 | 0.013 | 0.175 | 0.015 | 5.00E-06 | 0.406 | FALSE | 21 |
|  | rs11230859 | A | G | -0.725 | 0.015 | 0.158 | 0.014 | 4.18E-06 | 0.285 | FALSE | 21 |
|  | rs12669698 | C | T | 1.127 | 0.003 | 0.255 | 0.020 | 9.73E-06 | 0.867 | FALSE | 20 |
|  | rs17145573 | A | G | 1.708 | 0.003 | 0.365 | 0.021 | 2.87E-06 | 0.870 | FALSE | 22 |
|  | rs389558 | T | C | -0.783 | 0.005 | 0.160 | 0.013 | 9.74E-07 | 0.684 | FALSE | 24 |
|  | rs491806 | C | A | -0.948 | 0.013 | 0.211 | 0.016 | 6.92E-06 | 0.400 | FALSE | 20 |
|  | rs529324 | A | G | -0.826 | -0.004 | 0.179 | 0.014 | 4.16E-06 | 0.756 | FALSE | 21 |
|  | rs7778345 | G | A | 0.730 | 0.012 | 0.163 | 0.013 | 7.05E-06 | 0.375 | FALSE | 20 |
| family.Victivallaceae(id.2255) | rs10513202 | G | A | 1.427 | 0.073 | 0.321 | 0.036 | 8.58E-06 | 0.041 | FALSE | 20 |
|  | rs11090513 | T | G | 0.798 | 0.038 | 0.175 | 0.027 | 5.00E-06 | 0.164 | FALSE | 21 |
|  | rs11230859 | A | G | -0.725 | 0.005 | 0.158 | 0.024 | 4.18E-06 | 0.820 | FALSE | 21 |
|  | rs12669698 | C | T | 1.127 | 0.003 | 0.255 | 0.035 | 9.73E-06 | 0.921 | FALSE | 20 |
|  | rs17145573 | A | G | 1.708 | -0.016 | 0.365 | 0.037 | 2.87E-06 | 0.670 | FALSE | 22 |
|  | rs389558 | T | C | -0.783 | -0.012 | 0.160 | 0.024 | 9.74E-07 | 0.622 | FALSE | 24 |
|  | rs491806 | C | A | -0.948 | -0.015 | 0.211 | 0.027 | 6.92E-06 | 0.590 | FALSE | 20 |
|  | rs529324 | A | G | -0.826 | 0.011 | 0.179 | 0.024 | 4.16E-06 | 0.667 | FALSE | 21 |
|  | rs7778345 | G | A | 0.730 | 0.014 | 0.163 | 0.023 | 7.05E-06 | 0.530 | FALSE | 20 |
| order.Actinomycetales(id.420) | rs10513202 | G | A | 1.427 | 0.001 | 0.321 | 0.026 | 8.58E-06 | 0.959 | FALSE | 20 |
|  | rs11090513 | T | G | 0.798 | -0.031 | 0.175 | 0.019 | 5.00E-06 | 0.093 | FALSE | 21 |
|  | rs11230859 | A | G | -0.725 | -0.008 | 0.158 | 0.017 | 4.18E-06 | 0.636 | FALSE | 21 |
|  | rs12669698 | C | T | 1.127 | 0.017 | 0.255 | 0.026 | 9.73E-06 | 0.514 | FALSE | 20 |
|  | rs17145573 | A | G | 1.708 | -0.017 | 0.365 | 0.026 | 2.87E-06 | 0.521 | FALSE | 22 |
|  | rs389558 | T | C | -0.783 | -0.001 | 0.160 | 0.016 | 9.74E-07 | 0.947 | FALSE | 24 |
|  | rs491806 | C | A | -0.948 | -0.008 | 0.211 | 0.020 | 6.92E-06 | 0.671 | FALSE | 20 |
|  | rs529324 | A | G | -0.826 | -0.016 | 0.179 | 0.017 | 4.16E-06 | 0.349 | FALSE | 21 |
|  | rs7778345 | G | A | 0.730 | -0.018 | 0.163 | 0.016 | 7.05E-06 | 0.261 | FALSE | 20 |
| order.Bacillales(id.1674) | rs10513202 | G | A | 1.427 | 0.048 | 0.321 | 0.040 | 8.58E-06 | 0.230 | FALSE | 20 |
|  | rs11090513 | T | G | 0.798 | 0.002 | 0.175 | 0.030 | 5.00E-06 | 0.957 | FALSE | 21 |
|  | rs11230859 | A | G | -0.725 | 0.000 | 0.158 | 0.026 | 4.18E-06 | 0.986 | FALSE | 21 |
|  | rs12669698 | C | T | 1.127 | -0.040 | 0.255 | 0.039 | 9.73E-06 | 0.311 | FALSE | 20 |
|  | rs17145573 | A | G | 1.708 | -0.008 | 0.365 | 0.040 | 2.87E-06 | 0.837 | FALSE | 22 |
|  | rs389558 | T | C | -0.783 | -0.010 | 0.160 | 0.026 | 9.74E-07 | 0.710 | FALSE | 24 |
|  | rs491806 | C | A | -0.948 | -0.036 | 0.211 | 0.031 | 6.92E-06 | 0.247 | FALSE | 20 |
|  | rs529324 | A | G | -0.826 | 0.017 | 0.179 | 0.027 | 4.16E-06 | 0.517 | FALSE | 21 |
|  | rs7778345 | G | A | 0.730 | -0.021 | 0.163 | 0.025 | 7.05E-06 | 0.414 | FALSE | 20 |
| order.Bacteroidales(id.913) | rs10513202 | G | A | 1.427 | 0.001 | 0.321 | 0.017 | 8.58E-06 | 0.936 | FALSE | 20 |
|  | rs11090513 | T | G | 0.798 | -0.012 | 0.175 | 0.012 | 5.00E-06 | 0.331 | FALSE | 21 |
|  | rs11230859 | A | G | -0.725 | -0.003 | 0.158 | 0.011 | 4.18E-06 | 0.778 | FALSE | 21 |
|  | rs12669698 | C | T | 1.127 | -0.008 | 0.255 | 0.016 | 9.73E-06 | 0.638 | FALSE | 20 |
|  | rs17145573 | A | G | 1.708 | -0.025 | 0.365 | 0.017 | 2.87E-06 | 0.131 | FALSE | 22 |
|  | rs389558 | T | C | -0.783 | 0.005 | 0.160 | 0.011 | 9.74E-07 | 0.647 | FALSE | 24 |
|  | rs491806 | C | A | -0.948 | -0.017 | 0.211 | 0.013 | 6.92E-06 | 0.194 | FALSE | 20 |
|  | rs529324 | A | G | -0.826 | 0.002 | 0.179 | 0.011 | 4.16E-06 | 0.836 | FALSE | 21 |
|  | rs7778345 | G | A | 0.730 | 0.001 | 0.163 | 0.011 | 7.05E-06 | 0.950 | FALSE | 20 |
| order.Bifidobacteriales(id.432) | rs10513202 | G | A | 1.427 | 0.005 | 0.321 | 0.019 | 8.58E-06 | 0.791 | FALSE | 20 |
|  | rs11090513 | T | G | 0.798 | -0.017 | 0.175 | 0.014 | 5.00E-06 | 0.205 | FALSE | 21 |
|  | rs11230859 | A | G | -0.725 | -0.005 | 0.158 | 0.012 | 4.18E-06 | 0.691 | FALSE | 21 |
|  | rs12669698 | C | T | 1.127 | -0.024 | 0.255 | 0.018 | 9.73E-06 | 0.175 | FALSE | 20 |
|  | rs17145573 | A | G | 1.708 | 0.033 | 0.365 | 0.018 | 2.87E-06 | 0.078 | FALSE | 22 |
|  | rs389558 | T | C | -0.783 | -0.003 | 0.160 | 0.012 | 9.74E-07 | 0.783 | FALSE | 24 |
|  | rs491806 | C | A | -0.948 | -0.007 | 0.211 | 0.014 | 6.92E-06 | 0.632 | FALSE | 20 |
|  | rs529324 | A | G | -0.826 | -0.031 | 0.179 | 0.013 | 4.16E-06 | 0.015 | FALSE | 21 |
|  | rs7778345 | G | A | 0.730 | -0.005 | 0.163 | 0.012 | 7.05E-06 | 0.703 | FALSE | 20 |
| order.Burkholderiales(id.2874) | rs10513202 | G | A | 1.427 | 0.024 | 0.321 | 0.017 | 8.58E-06 | 0.172 | FALSE | 20 |
|  | rs11090513 | T | G | 0.798 | -0.007 | 0.175 | 0.013 | 5.00E-06 | 0.599 | FALSE | 21 |
|  | rs11230859 | A | G | -0.725 | 0.001 | 0.158 | 0.011 | 4.18E-06 | 0.941 | FALSE | 21 |
|  | rs12669698 | C | T | 1.127 | 0.018 | 0.255 | 0.017 | 9.73E-06 | 0.281 | FALSE | 20 |
|  | rs17145573 | A | G | 1.708 | 0.000 | 0.365 | 0.017 | 2.87E-06 | 0.980 | FALSE | 22 |
|  | rs389558 | T | C | -0.783 | -0.013 | 0.160 | 0.011 | 9.74E-07 | 0.245 | FALSE | 24 |
|  | rs491806 | C | A | -0.948 | -0.010 | 0.211 | 0.013 | 6.92E-06 | 0.473 | FALSE | 20 |
|  | rs529324 | A | G | -0.826 | -0.005 | 0.179 | 0.012 | 4.16E-06 | 0.640 | FALSE | 21 |
|  | rs7778345 | G | A | 0.730 | -0.008 | 0.163 | 0.011 | 7.05E-06 | 0.490 | FALSE | 20 |
| order.Clostridiales(id.1863) | rs10513202 | G | A | 1.427 | -0.022 | 0.321 | 0.017 | 8.58E-06 | 0.194 | FALSE | 20 |
|  | rs11090513 | T | G | 0.798 | 0.015 | 0.175 | 0.012 | 5.00E-06 | 0.212 | FALSE | 21 |
|  | rs11230859 | A | G | -0.725 | -0.013 | 0.158 | 0.011 | 4.18E-06 | 0.232 | FALSE | 21 |
|  | rs12669698 | C | T | 1.127 | -0.005 | 0.255 | 0.016 | 9.73E-06 | 0.779 | FALSE | 20 |
|  | rs17145573 | A | G | 1.708 | 0.013 | 0.365 | 0.017 | 2.87E-06 | 0.451 | FALSE | 22 |
|  | rs389558 | T | C | -0.783 | -0.001 | 0.160 | 0.011 | 9.74E-07 | 0.895 | FALSE | 24 |
|  | rs491806 | C | A | -0.948 | 0.018 | 0.211 | 0.013 | 6.92E-06 | 0.162 | FALSE | 20 |
|  | rs529324 | A | G | -0.826 | -0.003 | 0.179 | 0.011 | 4.16E-06 | 0.810 | FALSE | 21 |
|  | rs7778345 | G | A | 0.730 | 0.005 | 0.163 | 0.011 | 7.05E-06 | 0.625 | FALSE | 20 |
| order.Coriobacteriales(id.810) | rs10513202 | G | A | 1.427 | -0.017 | 0.321 | 0.017 | 8.58E-06 | 0.334 | FALSE | 20 |
|  | rs11090513 | T | G | 0.798 | -0.017 | 0.175 | 0.012 | 5.00E-06 | 0.176 | FALSE | 21 |
|  | rs11230859 | A | G | -0.725 | -0.001 | 0.158 | 0.011 | 4.18E-06 | 0.916 | FALSE | 21 |
|  | rs12669698 | C | T | 1.127 | 0.014 | 0.255 | 0.017 | 9.73E-06 | 0.387 | FALSE | 20 |
|  | rs17145573 | A | G | 1.708 | 0.022 | 0.365 | 0.017 | 2.87E-06 | 0.190 | FALSE | 22 |
|  | rs389558 | T | C | -0.783 | 0.002 | 0.160 | 0.011 | 9.74E-07 | 0.878 | FALSE | 24 |
|  | rs491806 | C | A | -0.948 | 0.011 | 0.211 | 0.013 | 6.92E-06 | 0.417 | FALSE | 20 |
|  | rs529324 | A | G | -0.826 | 0.012 | 0.179 | 0.012 | 4.16E-06 | 0.300 | FALSE | 21 |
|  | rs7778345 | G | A | 0.730 | 0.002 | 0.163 | 0.011 | 7.05E-06 | 0.829 | FALSE | 20 |
| order.Desulfovibrionales(id.3156) | rs10513202 | G | A | 1.427 | 0.019 | 0.321 | 0.019 | 8.58E-06 | 0.317 | FALSE | 20 |
|  | rs11090513 | T | G | 0.798 | -0.011 | 0.175 | 0.014 | 5.00E-06 | 0.411 | FALSE | 21 |
|  | rs11230859 | A | G | -0.725 | 0.012 | 0.158 | 0.012 | 4.18E-06 | 0.328 | FALSE | 21 |
|  | rs12669698 | C | T | 1.127 | 0.013 | 0.255 | 0.018 | 9.73E-06 | 0.460 | FALSE | 20 |
|  | rs17145573 | A | G | 1.708 | -0.017 | 0.365 | 0.018 | 2.87E-06 | 0.364 | FALSE | 22 |
|  | rs389558 | T | C | -0.783 | 0.007 | 0.160 | 0.012 | 9.74E-07 | 0.534 | FALSE | 24 |
|  | rs491806 | C | A | -0.948 | 0.016 | 0.211 | 0.014 | 6.92E-06 | 0.263 | FALSE | 20 |
|  | rs529324 | A | G | -0.826 | 0.004 | 0.179 | 0.012 | 4.16E-06 | 0.737 | FALSE | 21 |
|  | rs7778345 | G | A | 0.730 | 0.015 | 0.163 | 0.012 | 7.05E-06 | 0.200 | FALSE | 20 |
| order.Enterobacteriales(id.3468) | rs10513202 | G | A | 1.427 | 0.004 | 0.321 | 0.020 | 8.58E-06 | 0.820 | FALSE | 20 |
|  | rs11090513 | T | G | 0.798 | 0.005 | 0.175 | 0.014 | 5.00E-06 | 0.745 | FALSE | 21 |
|  | rs11230859 | A | G | -0.725 | 0.036 | 0.158 | 0.013 | 4.18E-06 | 0.004 | FALSE | 21 |
|  | rs12669698 | C | T | 1.127 | -0.004 | 0.255 | 0.019 | 9.73E-06 | 0.843 | FALSE | 20 |
|  | rs17145573 | A | G | 1.708 | 0.026 | 0.365 | 0.019 | 2.87E-06 | 0.179 | FALSE | 22 |
|  | rs389558 | T | C | -0.783 | 0.004 | 0.160 | 0.012 | 9.74E-07 | 0.749 | FALSE | 24 |
|  | rs491806 | C | A | -0.948 | -0.005 | 0.211 | 0.015 | 6.92E-06 | 0.755 | FALSE | 20 |
|  | rs529324 | A | G | -0.826 | 0.016 | 0.179 | 0.013 | 4.16E-06 | 0.215 | FALSE | 21 |
|  | rs7778345 | G | A | 0.730 | 0.014 | 0.163 | 0.013 | 7.05E-06 | 0.265 | FALSE | 20 |
| order.Erysipelotrichales(id.2148) | rs10513202 | G | A | 1.427 | -0.015 | 0.321 | 0.017 | 8.58E-06 | 0.389 | FALSE | 20 |
|  | rs11090513 | T | G | 0.798 | 0.001 | 0.175 | 0.012 | 5.00E-06 | 0.945 | FALSE | 21 |
|  | rs11230859 | A | G | -0.725 | -0.015 | 0.158 | 0.011 | 4.18E-06 | 0.168 | FALSE | 21 |
|  | rs12669698 | C | T | 1.127 | 0.016 | 0.255 | 0.016 | 9.73E-06 | 0.324 | FALSE | 20 |
|  | rs17145573 | A | G | 1.708 | 0.022 | 0.365 | 0.017 | 2.87E-06 | 0.193 | FALSE | 22 |
|  | rs389558 | T | C | -0.783 | 0.002 | 0.160 | 0.011 | 9.74E-07 | 0.866 | FALSE | 24 |
|  | rs491806 | C | A | -0.948 | -0.004 | 0.211 | 0.013 | 6.92E-06 | 0.773 | FALSE | 20 |
|  | rs529324 | A | G | -0.826 | -0.012 | 0.179 | 0.011 | 4.16E-06 | 0.277 | FALSE | 21 |
|  | rs7778345 | G | A | 0.730 | 0.009 | 0.163 | 0.011 | 7.05E-06 | 0.415 | FALSE | 20 |
| order.Gastranaerophilales(id.1591) | rs10513202 | G | A | 1.427 | 0.037 | 0.321 | 0.030 | 8.58E-06 | 0.207 | FALSE | 20 |
|  | rs11090513 | T | G | 0.798 | -0.013 | 0.175 | 0.022 | 5.00E-06 | 0.558 | FALSE | 21 |
|  | rs11230859 | A | G | -0.725 | 0.017 | 0.158 | 0.019 | 4.18E-06 | 0.382 | FALSE | 21 |
|  | rs12669698 | C | T | 1.127 | 0.002 | 0.255 | 0.030 | 9.73E-06 | 0.946 | FALSE | 20 |
|  | rs17145573 | A | G | 1.708 | 0.008 | 0.365 | 0.030 | 2.87E-06 | 0.787 | FALSE | 22 |
|  | rs389558 | T | C | -0.783 | 0.006 | 0.160 | 0.019 | 9.74E-07 | 0.761 | FALSE | 24 |
|  | rs491806 | C | A | -0.948 | 0.013 | 0.211 | 0.022 | 6.92E-06 | 0.573 | FALSE | 20 |
|  | rs529324 | A | G | -0.826 | 0.005 | 0.179 | 0.020 | 4.16E-06 | 0.794 | FALSE | 21 |
|  | rs7778345 | G | A | 0.730 | -0.013 | 0.163 | 0.019 | 7.05E-06 | 0.498 | FALSE | 20 |
| order.Lactobacillales(id.1800) | rs10513202 | G | A | 1.427 | -0.045 | 0.321 | 0.017 | 8.58E-06 | 0.009 | FALSE | 20 |
|  | rs11090513 | T | G | 0.798 | 0.007 | 0.175 | 0.013 | 5.00E-06 | 0.556 | FALSE | 21 |
|  | rs11230859 | A | G | -0.725 | -0.002 | 0.158 | 0.011 | 4.18E-06 | 0.860 | FALSE | 21 |
|  | rs12669698 | C | T | 1.127 | 0.027 | 0.255 | 0.017 | 9.73E-06 | 0.105 | FALSE | 20 |
|  | rs17145573 | A | G | 1.708 | 0.009 | 0.365 | 0.017 | 2.87E-06 | 0.606 | FALSE | 22 |
|  | rs389558 | T | C | -0.783 | -0.020 | 0.160 | 0.011 | 9.74E-07 | 0.065 | FALSE | 24 |
|  | rs491806 | C | A | -0.948 | 0.008 | 0.211 | 0.013 | 6.92E-06 | 0.533 | FALSE | 20 |
|  | rs529324 | A | G | -0.826 | 0.009 | 0.179 | 0.012 | 4.16E-06 | 0.463 | FALSE | 21 |
|  | rs7778345 | G | A | 0.730 | 0.003 | 0.163 | 0.011 | 7.05E-06 | 0.808 | FALSE | 20 |
| order.Methanobacteriales(id.120) | rs10513202 | G | A | 1.427 | 0.025 | 0.321 | 0.037 | 8.58E-06 | 0.491 | FALSE | 20 |
|  | rs11090513 | T | G | 0.798 | -0.006 | 0.175 | 0.029 | 5.00E-06 | 0.832 | FALSE | 21 |
|  | rs11230859 | A | G | -0.725 | 0.005 | 0.158 | 0.024 | 4.18E-06 | 0.849 | FALSE | 21 |
|  | rs12669698 | C | T | 1.127 | -0.032 | 0.255 | 0.037 | 9.73E-06 | 0.386 | FALSE | 20 |
|  | rs17145573 | A | G | 1.708 | -0.040 | 0.365 | 0.038 | 2.87E-06 | 0.286 | FALSE | 22 |
|  | rs389558 | T | C | -0.783 | 0.029 | 0.160 | 0.025 | 9.74E-07 | 0.245 | FALSE | 24 |
|  | rs491806 | C | A | -0.948 | 0.023 | 0.211 | 0.029 | 6.92E-06 | 0.429 | FALSE | 20 |
|  | rs529324 | A | G | -0.826 | -0.001 | 0.179 | 0.025 | 4.16E-06 | 0.983 | FALSE | 21 |
|  | rs7778345 | G | A | 0.730 | -0.030 | 0.163 | 0.024 | 7.05E-06 | 0.203 | FALSE | 20 |
| order.MollicutesRF9(id.11579) | rs10513202 | G | A | 1.427 | 0.017 | 0.321 | 0.022 | 8.58E-06 | 0.451 | FALSE | 20 |
|  | rs11090513 | T | G | 0.798 | 0.012 | 0.175 | 0.016 | 5.00E-06 | 0.463 | FALSE | 21 |
|  | rs11230859 | A | G | -0.725 | -0.006 | 0.158 | 0.015 | 4.18E-06 | 0.685 | FALSE | 21 |
|  | rs12669698 | C | T | 1.127 | 0.000 | 0.255 | 0.022 | 9.73E-06 | 0.997 | FALSE | 20 |
|  | rs17145573 | A | G | 1.708 | 0.004 | 0.365 | 0.022 | 2.87E-06 | 0.857 | FALSE | 22 |
|  | rs389558 | T | C | -0.783 | -0.005 | 0.160 | 0.014 | 9.74E-07 | 0.702 | FALSE | 24 |
|  | rs491806 | C | A | -0.948 | 0.013 | 0.211 | 0.017 | 6.92E-06 | 0.435 | FALSE | 20 |
|  | rs529324 | A | G | -0.826 | 0.015 | 0.179 | 0.015 | 4.16E-06 | 0.308 | FALSE | 21 |
|  | rs7778345 | G | A | 0.730 | 0.017 | 0.163 | 0.014 | 7.05E-06 | 0.226 | FALSE | 20 |
| order.NB1n(id.3953) | rs10513202 | G | A | 1.427 | 0.046 | 0.321 | 0.032 | 8.58E-06 | 0.146 | FALSE | 20 |
|  | rs11090513 | T | G | 0.798 | 0.038 | 0.175 | 0.023 | 5.00E-06 | 0.103 | FALSE | 21 |
|  | rs11230859 | A | G | -0.725 | 0.005 | 0.158 | 0.021 | 4.18E-06 | 0.818 | FALSE | 21 |
|  | rs12669698 | C | T | 1.127 | -0.038 | 0.255 | 0.032 | 9.73E-06 | 0.229 | FALSE | 20 |
|  | rs17145573 | A | G | 1.708 | 0.022 | 0.365 | 0.033 | 2.87E-06 | 0.506 | FALSE | 22 |
|  | rs389558 | T | C | -0.783 | 0.008 | 0.160 | 0.021 | 9.74E-07 | 0.701 | FALSE | 24 |
|  | rs491806 | C | A | -0.948 | -0.025 | 0.211 | 0.024 | 6.92E-06 | 0.292 | FALSE | 20 |
|  | rs529324 | A | G | -0.826 | 0.016 | 0.179 | 0.022 | 4.16E-06 | 0.469 | FALSE | 21 |
|  | rs7778345 | G | A | 0.730 | 0.025 | 0.163 | 0.020 | 7.05E-06 | 0.219 | FALSE | 20 |
| order.Pasteurellales(id.3688) | rs10513202 | G | A | 1.427 | 0.014 | 0.321 | 0.024 | 8.58E-06 | 0.541 | FALSE | 20 |
|  | rs11090513 | T | G | 0.798 | 0.001 | 0.175 | 0.017 | 5.00E-06 | 0.972 | FALSE | 21 |
|  | rs11230859 | A | G | -0.725 | 0.000 | 0.158 | 0.015 | 4.18E-06 | 0.978 | FALSE | 21 |
|  | rs12669698 | C | T | 1.127 | 0.020 | 0.255 | 0.022 | 9.73E-06 | 0.372 | FALSE | 20 |
|  | rs17145573 | A | G | 1.708 | -0.009 | 0.365 | 0.023 | 2.87E-06 | 0.715 | FALSE | 22 |
|  | rs389558 | T | C | -0.783 | -0.025 | 0.160 | 0.015 | 9.74E-07 | 0.094 | FALSE | 24 |
|  | rs491806 | C | A | -0.948 | 0.017 | 0.211 | 0.018 | 6.92E-06 | 0.340 | FALSE | 20 |
|  | rs529324 | A | G | -0.826 | 0.020 | 0.179 | 0.016 | 4.16E-06 | 0.212 | FALSE | 21 |
|  | rs7778345 | G | A | 0.730 | -0.004 | 0.163 | 0.015 | 7.05E-06 | 0.786 | FALSE | 20 |
| order.Rhodospirillales(id.2667) | rs10513202 | G | A | 1.427 | 0.028 | 0.321 | 0.025 | 8.58E-06 | 0.258 | FALSE | 20 |
|  | rs11090513 | T | G | 0.798 | -0.012 | 0.175 | 0.018 | 5.00E-06 | 0.518 | FALSE | 21 |
|  | rs11230859 | A | G | -0.725 | 0.000 | 0.158 | 0.016 | 4.18E-06 | 0.998 | FALSE | 21 |
|  | rs12669698 | C | T | 1.127 | 0.029 | 0.255 | 0.024 | 9.73E-06 | 0.235 | FALSE | 20 |
|  | rs17145573 | A | G | 1.708 | 0.007 | 0.365 | 0.025 | 2.87E-06 | 0.786 | FALSE | 22 |
|  | rs389558 | T | C | -0.783 | -0.008 | 0.160 | 0.016 | 9.74E-07 | 0.633 | FALSE | 24 |
|  | rs491806 | C | A | -0.948 | -0.001 | 0.211 | 0.019 | 6.92E-06 | 0.937 | FALSE | 20 |
|  | rs529324 | A | G | -0.826 | 0.006 | 0.179 | 0.017 | 4.16E-06 | 0.712 | FALSE | 21 |
|  | rs7778345 | G | A | 0.730 | -0.007 | 0.163 | 0.015 | 7.05E-06 | 0.666 | FALSE | 20 |
| order.Selenomonadales(id.2165) | rs10513202 | G | A | 1.427 | 0.005 | 0.321 | 0.017 | 8.58E-06 | 0.771 | FALSE | 20 |
|  | rs11090513 | T | G | 0.798 | -0.018 | 0.175 | 0.012 | 5.00E-06 | 0.139 | FALSE | 21 |
|  | rs11230859 | A | G | -0.725 | 0.014 | 0.158 | 0.011 | 4.18E-06 | 0.196 | FALSE | 21 |
|  | rs12669698 | C | T | 1.127 | -0.009 | 0.255 | 0.016 | 9.73E-06 | 0.566 | FALSE | 20 |
|  | rs17145573 | A | G | 1.708 | -0.012 | 0.365 | 0.017 | 2.87E-06 | 0.487 | FALSE | 22 |
|  | rs389558 | T | C | -0.783 | 0.005 | 0.160 | 0.011 | 9.74E-07 | 0.663 | FALSE | 24 |
|  | rs491806 | C | A | -0.948 | 0.007 | 0.211 | 0.013 | 6.92E-06 | 0.584 | FALSE | 20 |
|  | rs529324 | A | G | -0.826 | 0.008 | 0.179 | 0.011 | 4.16E-06 | 0.461 | FALSE | 21 |
|  | rs7778345 | G | A | 0.730 | 0.000 | 0.163 | 0.011 | 7.05E-06 | 0.968 | FALSE | 20 |
| order.Verrucomicrobiales(id.4030) | rs10513202 | G | A | 1.427 | -0.016 | 0.321 | 0.021 | 8.58E-06 | 0.451 | FALSE | 20 |
|  | rs11090513 | T | G | 0.798 | 0.013 | 0.175 | 0.015 | 5.00E-06 | 0.408 | FALSE | 21 |
|  | rs11230859 | A | G | -0.725 | 0.014 | 0.158 | 0.014 | 4.18E-06 | 0.287 | FALSE | 21 |
|  | rs12669698 | C | T | 1.127 | 0.003 | 0.255 | 0.020 | 9.73E-06 | 0.866 | FALSE | 20 |
|  | rs17145573 | A | G | 1.708 | 0.003 | 0.365 | 0.021 | 2.87E-06 | 0.878 | FALSE | 22 |
|  | rs389558 | T | C | -0.783 | 0.005 | 0.160 | 0.013 | 9.74E-07 | 0.686 | FALSE | 24 |
|  | rs491806 | C | A | -0.948 | 0.013 | 0.211 | 0.016 | 6.92E-06 | 0.402 | FALSE | 20 |
|  | rs529324 | A | G | -0.826 | -0.004 | 0.179 | 0.014 | 4.16E-06 | 0.756 | FALSE | 21 |
|  | rs7778345 | G | A | 0.730 | 0.012 | 0.163 | 0.013 | 7.05E-06 | 0.374 | FALSE | 20 |
| order.Victivallales(id.2254) | rs10513202 | G | A | 1.427 | 0.026 | 0.321 | 0.033 | 8.58E-06 | 0.430 | FALSE | 20 |
|  | rs11090513 | T | G | 0.798 | 0.023 | 0.175 | 0.025 | 5.00E-06 | 0.353 | FALSE | 21 |
|  | rs11230859 | A | G | -0.725 | -0.001 | 0.158 | 0.021 | 4.18E-06 | 0.947 | FALSE | 21 |
|  | rs12669698 | C | T | 1.127 | 0.016 | 0.255 | 0.032 | 9.73E-06 | 0.611 | FALSE | 20 |
|  | rs17145573 | A | G | 1.708 | -0.027 | 0.365 | 0.033 | 2.87E-06 | 0.412 | FALSE | 22 |
|  | rs389558 | T | C | -0.783 | -0.004 | 0.160 | 0.022 | 9.74E-07 | 0.843 | FALSE | 24 |
|  | rs491806 | C | A | -0.948 | -0.029 | 0.211 | 0.025 | 6.92E-06 | 0.251 | FALSE | 20 |
|  | rs529324 | A | G | -0.826 | 0.006 | 0.179 | 0.022 | 4.16E-06 | 0.782 | FALSE | 21 |
|  | rs7778345 | G | A | 0.730 | 0.018 | 0.163 | 0.021 | 7.05E-06 | 0.392 | FALSE | 20 |
| class.Actinobacteria(id.419) | rs10513202 | G | A | 1.427 | 0.004 | 0.321 | 0.018 | 8.58E-06 | 0.831 | FALSE | 20 |
|  | rs11090513 | T | G | 0.798 | -0.023 | 0.175 | 0.013 | 5.00E-06 | 0.081 | FALSE | 21 |
|  | rs11230859 | A | G | -0.725 | -0.002 | 0.158 | 0.012 | 4.18E-06 | 0.851 | FALSE | 21 |
|  | rs12669698 | C | T | 1.127 | -0.024 | 0.255 | 0.017 | 9.73E-06 | 0.166 | FALSE | 20 |
|  | rs17145573 | A | G | 1.708 | 0.032 | 0.365 | 0.018 | 2.87E-06 | 0.073 | FALSE | 22 |
|  | rs389558 | T | C | -0.783 | -0.006 | 0.160 | 0.011 | 9.74E-07 | 0.624 | FALSE | 24 |
|  | rs491806 | C | A | -0.948 | 0.000 | 0.211 | 0.014 | 6.92E-06 | 0.996 | FALSE | 20 |
|  | rs529324 | A | G | -0.826 | -0.022 | 0.179 | 0.012 | 4.16E-06 | 0.069 | FALSE | 21 |
|  | rs7778345 | G | A | 0.730 | 0.003 | 0.163 | 0.011 | 7.05E-06 | 0.795 | FALSE | 20 |
| class.Alphaproteobacteria(id.2379) | rs10513202 | G | A | 1.427 | 0.016 | 0.321 | 0.023 | 8.58E-06 | 0.482 | FALSE | 20 |
|  | rs11090513 | T | G | 0.798 | -0.005 | 0.175 | 0.017 | 5.00E-06 | 0.764 | FALSE | 21 |
|  | rs11230859 | A | G | -0.725 | 0.007 | 0.158 | 0.015 | 4.18E-06 | 0.650 | FALSE | 21 |
|  | rs12669698 | C | T | 1.127 | 0.016 | 0.255 | 0.023 | 9.73E-06 | 0.497 | FALSE | 20 |
|  | rs17145573 | A | G | 1.708 | 0.005 | 0.365 | 0.024 | 2.87E-06 | 0.832 | FALSE | 22 |
|  | rs389558 | T | C | -0.783 | -0.007 | 0.160 | 0.015 | 9.74E-07 | 0.649 | FALSE | 24 |
|  | rs491806 | C | A | -0.948 | 0.008 | 0.211 | 0.018 | 6.92E-06 | 0.662 | FALSE | 20 |
|  | rs529324 | A | G | -0.826 | 0.010 | 0.179 | 0.016 | 4.16E-06 | 0.524 | FALSE | 21 |
|  | rs7778345 | G | A | 0.730 | -0.005 | 0.163 | 0.015 | 7.05E-06 | 0.744 | FALSE | 20 |
| class.Bacilli(id.1673) | rs10513202 | G | A | 1.427 | -0.046 | 0.321 | 0.017 | 8.58E-06 | 0.008 | FALSE | 20 |
|  | rs11090513 | T | G | 0.798 | 0.010 | 0.175 | 0.013 | 5.00E-06 | 0.430 | FALSE | 21 |
|  | rs11230859 | A | G | -0.725 | -0.003 | 0.158 | 0.011 | 4.18E-06 | 0.804 | FALSE | 21 |
|  | rs12669698 | C | T | 1.127 | 0.023 | 0.255 | 0.017 | 9.73E-06 | 0.168 | FALSE | 20 |
|  | rs17145573 | A | G | 1.708 | 0.010 | 0.365 | 0.017 | 2.87E-06 | 0.560 | FALSE | 22 |
|  | rs389558 | T | C | -0.783 | -0.021 | 0.160 | 0.011 | 9.74E-07 | 0.054 | FALSE | 24 |
|  | rs491806 | C | A | -0.948 | 0.008 | 0.211 | 0.013 | 6.92E-06 | 0.532 | FALSE | 20 |
|  | rs529324 | A | G | -0.826 | 0.009 | 0.179 | 0.012 | 4.16E-06 | 0.463 | FALSE | 21 |
|  | rs7778345 | G | A | 0.730 | 0.001 | 0.163 | 0.011 | 7.05E-06 | 0.917 | FALSE | 20 |
| class.Bacteroidia(id.912) | rs10513202 | G | A | 1.427 | 0.001 | 0.321 | 0.017 | 8.58E-06 | 0.936 | FALSE | 20 |
|  | rs11090513 | T | G | 0.798 | -0.012 | 0.175 | 0.012 | 5.00E-06 | 0.331 | FALSE | 21 |
|  | rs11230859 | A | G | -0.725 | -0.003 | 0.158 | 0.011 | 4.18E-06 | 0.778 | FALSE | 21 |
|  | rs12669698 | C | T | 1.127 | -0.008 | 0.255 | 0.016 | 9.73E-06 | 0.638 | FALSE | 20 |
|  | rs17145573 | A | G | 1.708 | -0.025 | 0.365 | 0.017 | 2.87E-06 | 0.131 | FALSE | 22 |
|  | rs389558 | T | C | -0.783 | 0.005 | 0.160 | 0.011 | 9.74E-07 | 0.647 | FALSE | 24 |
|  | rs491806 | C | A | -0.948 | -0.017 | 0.211 | 0.013 | 6.92E-06 | 0.194 | FALSE | 20 |
|  | rs529324 | A | G | -0.826 | 0.002 | 0.179 | 0.011 | 4.16E-06 | 0.836 | FALSE | 21 |
|  | rs7778345 | G | A | 0.730 | 0.001 | 0.163 | 0.011 | 7.05E-06 | 0.950 | FALSE | 20 |
| class.Betaproteobacteria(id.2867) | rs10513202 | G | A | 1.427 | 0.022 | 0.321 | 0.017 | 8.58E-06 | 0.203 | FALSE | 20 |
|  | rs11090513 | T | G | 0.798 | -0.009 | 0.175 | 0.013 | 5.00E-06 | 0.492 | FALSE | 21 |
|  | rs11230859 | A | G | -0.725 | 0.002 | 0.158 | 0.011 | 4.18E-06 | 0.874 | FALSE | 21 |
|  | rs12669698 | C | T | 1.127 | 0.017 | 0.255 | 0.017 | 9.73E-06 | 0.302 | FALSE | 20 |
|  | rs17145573 | A | G | 1.708 | -0.001 | 0.365 | 0.017 | 2.87E-06 | 0.942 | FALSE | 22 |
|  | rs389558 | T | C | -0.783 | -0.012 | 0.160 | 0.011 | 9.74E-07 | 0.294 | FALSE | 24 |
|  | rs491806 | C | A | -0.948 | -0.012 | 0.211 | 0.013 | 6.92E-06 | 0.368 | FALSE | 20 |
|  | rs529324 | A | G | -0.826 | -0.002 | 0.179 | 0.012 | 4.16E-06 | 0.879 | FALSE | 21 |
|  | rs7778345 | G | A | 0.730 | -0.008 | 0.163 | 0.011 | 7.05E-06 | 0.499 | FALSE | 20 |
| class.Clostridia(id.1859) | rs10513202 | G | A | 1.427 | -0.021 | 0.321 | 0.017 | 8.58E-06 | 0.209 | FALSE | 20 |
|  | rs11090513 | T | G | 0.798 | 0.016 | 0.175 | 0.012 | 5.00E-06 | 0.203 | FALSE | 21 |
|  | rs11230859 | A | G | -0.725 | -0.013 | 0.158 | 0.011 | 4.18E-06 | 0.219 | FALSE | 21 |
|  | rs12669698 | C | T | 1.127 | -0.005 | 0.255 | 0.016 | 9.73E-06 | 0.769 | FALSE | 20 |
|  | rs17145573 | A | G | 1.708 | 0.013 | 0.365 | 0.017 | 2.87E-06 | 0.437 | FALSE | 22 |
|  | rs389558 | T | C | -0.783 | -0.001 | 0.160 | 0.011 | 9.74E-07 | 0.891 | FALSE | 24 |
|  | rs491806 | C | A | -0.948 | 0.018 | 0.211 | 0.013 | 6.92E-06 | 0.158 | FALSE | 20 |
|  | rs529324 | A | G | -0.826 | -0.002 | 0.179 | 0.011 | 4.16E-06 | 0.829 | FALSE | 21 |
|  | rs7778345 | G | A | 0.730 | 0.005 | 0.163 | 0.011 | 7.05E-06 | 0.610 | FALSE | 20 |
| class.Coriobacteriia(id.809) | rs10513202 | G | A | 1.427 | -0.017 | 0.321 | 0.017 | 8.58E-06 | 0.334 | FALSE | 20 |
|  | rs11090513 | T | G | 0.798 | -0.017 | 0.175 | 0.012 | 5.00E-06 | 0.176 | FALSE | 21 |
|  | rs11230859 | A | G | -0.725 | -0.001 | 0.158 | 0.011 | 4.18E-06 | 0.916 | FALSE | 21 |
|  | rs12669698 | C | T | 1.127 | 0.014 | 0.255 | 0.017 | 9.73E-06 | 0.387 | FALSE | 20 |
|  | rs17145573 | A | G | 1.708 | 0.022 | 0.365 | 0.017 | 2.87E-06 | 0.190 | FALSE | 22 |
|  | rs389558 | T | C | -0.783 | 0.002 | 0.160 | 0.011 | 9.74E-07 | 0.878 | FALSE | 24 |
|  | rs491806 | C | A | -0.948 | 0.011 | 0.211 | 0.013 | 6.92E-06 | 0.417 | FALSE | 20 |
|  | rs529324 | A | G | -0.826 | 0.012 | 0.179 | 0.012 | 4.16E-06 | 0.300 | FALSE | 21 |
|  | rs7778345 | G | A | 0.730 | 0.002 | 0.163 | 0.011 | 7.05E-06 | 0.829 | FALSE | 20 |
| class.Deltaproteobacteria(id.3087) | rs10513202 | G | A | 1.427 | 0.019 | 0.321 | 0.019 | 8.58E-06 | 0.305 | FALSE | 20 |
|  | rs11090513 | T | G | 0.798 | -0.009 | 0.175 | 0.013 | 5.00E-06 | 0.489 | FALSE | 21 |
|  | rs11230859 | A | G | -0.725 | 0.013 | 0.158 | 0.012 | 4.18E-06 | 0.297 | FALSE | 21 |
|  | rs12669698 | C | T | 1.127 | 0.015 | 0.255 | 0.018 | 9.73E-06 | 0.402 | FALSE | 20 |
|  | rs17145573 | A | G | 1.708 | -0.017 | 0.365 | 0.018 | 2.87E-06 | 0.356 | FALSE | 22 |
|  | rs389558 | T | C | -0.783 | 0.007 | 0.160 | 0.012 | 9.74E-07 | 0.561 | FALSE | 24 |
|  | rs491806 | C | A | -0.948 | 0.015 | 0.211 | 0.014 | 6.92E-06 | 0.281 | FALSE | 20 |
|  | rs529324 | A | G | -0.826 | 0.005 | 0.179 | 0.012 | 4.16E-06 | 0.690 | FALSE | 21 |
|  | rs7778345 | G | A | 0.730 | 0.014 | 0.163 | 0.012 | 7.05E-06 | 0.247 | FALSE | 20 |
| class.Erysipelotrichia(id.2147) | rs10513202 | G | A | 1.427 | -0.015 | 0.321 | 0.017 | 8.58E-06 | 0.389 | FALSE | 20 |
|  | rs11090513 | T | G | 0.798 | 0.001 | 0.175 | 0.012 | 5.00E-06 | 0.945 | FALSE | 21 |
|  | rs11230859 | A | G | -0.725 | -0.015 | 0.158 | 0.011 | 4.18E-06 | 0.168 | FALSE | 21 |
|  | rs12669698 | C | T | 1.127 | 0.016 | 0.255 | 0.016 | 9.73E-06 | 0.324 | FALSE | 20 |
|  | rs17145573 | A | G | 1.708 | 0.022 | 0.365 | 0.017 | 2.87E-06 | 0.193 | FALSE | 22 |
|  | rs389558 | T | C | -0.783 | 0.002 | 0.160 | 0.011 | 9.74E-07 | 0.866 | FALSE | 24 |
|  | rs491806 | C | A | -0.948 | -0.004 | 0.211 | 0.013 | 6.92E-06 | 0.773 | FALSE | 20 |
|  | rs529324 | A | G | -0.826 | -0.012 | 0.179 | 0.011 | 4.16E-06 | 0.277 | FALSE | 21 |
|  | rs7778345 | G | A | 0.730 | 0.009 | 0.163 | 0.011 | 7.05E-06 | 0.415 | FALSE | 20 |
| class.Gammaproteobacteria(id.3303) | rs10513202 | G | A | 1.427 | 0.003 | 0.321 | 0.018 | 8.58E-06 | 0.869 | FALSE | 20 |
|  | rs11090513 | T | G | 0.798 | -0.008 | 0.175 | 0.013 | 5.00E-06 | 0.556 | FALSE | 21 |
|  | rs11230859 | A | G | -0.725 | 0.022 | 0.158 | 0.012 | 4.18E-06 | 0.055 | FALSE | 21 |
|  | rs12669698 | C | T | 1.127 | 0.022 | 0.255 | 0.017 | 9.73E-06 | 0.200 | FALSE | 20 |
|  | rs17145573 | A | G | 1.708 | 0.017 | 0.365 | 0.018 | 2.87E-06 | 0.356 | FALSE | 22 |
|  | rs389558 | T | C | -0.783 | -0.004 | 0.160 | 0.011 | 9.74E-07 | 0.715 | FALSE | 24 |
|  | rs491806 | C | A | -0.948 | -0.002 | 0.211 | 0.014 | 6.92E-06 | 0.872 | FALSE | 20 |
|  | rs529324 | A | G | -0.826 | 0.019 | 0.179 | 0.012 | 4.16E-06 | 0.125 | FALSE | 21 |
|  | rs7778345 | G | A | 0.730 | 0.001 | 0.163 | 0.012 | 7.05E-06 | 0.952 | FALSE | 20 |
| class.Lentisphaeria(id.2250) | rs10513202 | G | A | 1.427 | 0.026 | 0.321 | 0.033 | 8.58E-06 | 0.430 | FALSE | 20 |
|  | rs11090513 | T | G | 0.798 | 0.023 | 0.175 | 0.025 | 5.00E-06 | 0.353 | FALSE | 21 |
|  | rs11230859 | A | G | -0.725 | -0.001 | 0.158 | 0.021 | 4.18E-06 | 0.947 | FALSE | 21 |
|  | rs12669698 | C | T | 1.127 | 0.016 | 0.255 | 0.032 | 9.73E-06 | 0.611 | FALSE | 20 |
|  | rs17145573 | A | G | 1.708 | -0.027 | 0.365 | 0.033 | 2.87E-06 | 0.412 | FALSE | 22 |
|  | rs389558 | T | C | -0.783 | -0.004 | 0.160 | 0.022 | 9.74E-07 | 0.843 | FALSE | 24 |
|  | rs491806 | C | A | -0.948 | -0.029 | 0.211 | 0.025 | 6.92E-06 | 0.251 | FALSE | 20 |
|  | rs529324 | A | G | -0.826 | 0.006 | 0.179 | 0.022 | 4.16E-06 | 0.782 | FALSE | 21 |
|  | rs7778345 | G | A | 0.730 | 0.018 | 0.163 | 0.021 | 7.05E-06 | 0.392 | FALSE | 20 |
| class.Melainabacteria(id.1589) | rs10513202 | G | A | 1.427 | 0.037 | 0.321 | 0.030 | 8.58E-06 | 0.212 | FALSE | 20 |
|  | rs11090513 | T | G | 0.798 | -0.013 | 0.175 | 0.022 | 5.00E-06 | 0.572 | FALSE | 21 |
|  | rs11230859 | A | G | -0.725 | 0.018 | 0.158 | 0.019 | 4.18E-06 | 0.350 | FALSE | 21 |
|  | rs12669698 | C | T | 1.127 | 0.005 | 0.255 | 0.029 | 9.73E-06 | 0.863 | FALSE | 20 |
|  | rs17145573 | A | G | 1.708 | 0.009 | 0.365 | 0.030 | 2.87E-06 | 0.771 | FALSE | 22 |
|  | rs389558 | T | C | -0.783 | 0.005 | 0.160 | 0.019 | 9.74E-07 | 0.796 | FALSE | 24 |
|  | rs491806 | C | A | -0.948 | 0.012 | 0.211 | 0.022 | 6.92E-06 | 0.605 | FALSE | 20 |
|  | rs529324 | A | G | -0.826 | 0.005 | 0.179 | 0.020 | 4.16E-06 | 0.812 | FALSE | 21 |
|  | rs7778345 | G | A | 0.730 | -0.012 | 0.163 | 0.019 | 7.05E-06 | 0.514 | FALSE | 20 |
| class.Methanobacteria(id.119) | rs10513202 | G | A | 1.427 | 0.025 | 0.321 | 0.037 | 8.58E-06 | 0.491 | FALSE | 20 |
|  | rs11090513 | T | G | 0.798 | -0.006 | 0.175 | 0.029 | 5.00E-06 | 0.832 | FALSE | 21 |
|  | rs11230859 | A | G | -0.725 | 0.005 | 0.158 | 0.024 | 4.18E-06 | 0.849 | FALSE | 21 |
|  | rs12669698 | C | T | 1.127 | -0.032 | 0.255 | 0.037 | 9.73E-06 | 0.386 | FALSE | 20 |
|  | rs17145573 | A | G | 1.708 | -0.040 | 0.365 | 0.038 | 2.87E-06 | 0.286 | FALSE | 22 |
|  | rs389558 | T | C | -0.783 | 0.029 | 0.160 | 0.025 | 9.74E-07 | 0.245 | FALSE | 24 |
|  | rs491806 | C | A | -0.948 | 0.023 | 0.211 | 0.029 | 6.92E-06 | 0.429 | FALSE | 20 |
|  | rs529324 | A | G | -0.826 | -0.001 | 0.179 | 0.025 | 4.16E-06 | 0.983 | FALSE | 21 |
|  | rs7778345 | G | A | 0.730 | -0.030 | 0.163 | 0.024 | 7.05E-06 | 0.203 | FALSE | 20 |
| class.Mollicutes(id.3920) | rs10513202 | G | A | 1.427 | 0.014 | 0.321 | 0.021 | 8.58E-06 | 0.500 | FALSE | 20 |
|  | rs11090513 | T | G | 0.798 | 0.012 | 0.175 | 0.016 | 5.00E-06 | 0.449 | FALSE | 21 |
|  | rs11230859 | A | G | -0.725 | -0.002 | 0.158 | 0.014 | 4.18E-06 | 0.866 | FALSE | 21 |
|  | rs12669698 | C | T | 1.127 | 0.009 | 0.255 | 0.021 | 9.73E-06 | 0.666 | FALSE | 20 |
|  | rs17145573 | A | G | 1.708 | 0.005 | 0.365 | 0.021 | 2.87E-06 | 0.814 | FALSE | 22 |
|  | rs389558 | T | C | -0.783 | 0.003 | 0.160 | 0.014 | 9.74E-07 | 0.800 | FALSE | 24 |
|  | rs491806 | C | A | -0.948 | 0.007 | 0.211 | 0.016 | 6.92E-06 | 0.650 | FALSE | 20 |
|  | rs529324 | A | G | -0.826 | 0.007 | 0.179 | 0.014 | 4.16E-06 | 0.614 | FALSE | 21 |
|  | rs7778345 | G | A | 0.730 | 0.005 | 0.163 | 0.014 | 7.05E-06 | 0.708 | FALSE | 20 |
| class.Negativicutes(id.2164) | rs10513202 | G | A | 1.427 | 0.005 | 0.321 | 0.017 | 8.58E-06 | 0.771 | FALSE | 20 |
|  | rs11090513 | T | G | 0.798 | -0.018 | 0.175 | 0.012 | 5.00E-06 | 0.139 | FALSE | 21 |
|  | rs11230859 | A | G | -0.725 | 0.014 | 0.158 | 0.011 | 4.18E-06 | 0.196 | FALSE | 21 |
|  | rs12669698 | C | T | 1.127 | -0.009 | 0.255 | 0.016 | 9.73E-06 | 0.566 | FALSE | 20 |
|  | rs17145573 | A | G | 1.708 | -0.012 | 0.365 | 0.017 | 2.87E-06 | 0.487 | FALSE | 22 |
|  | rs389558 | T | C | -0.783 | 0.005 | 0.160 | 0.011 | 9.74E-07 | 0.663 | FALSE | 24 |
|  | rs491806 | C | A | -0.948 | 0.007 | 0.211 | 0.013 | 6.92E-06 | 0.584 | FALSE | 20 |
|  | rs529324 | A | G | -0.826 | 0.008 | 0.179 | 0.011 | 4.16E-06 | 0.461 | FALSE | 21 |
|  | rs7778345 | G | A | 0.730 | 0.000 | 0.163 | 0.011 | 7.05E-06 | 0.968 | FALSE | 20 |
| class.Verrucomicrobiae(id.4029) | rs10513202 | G | A | 1.427 | -0.016 | 0.321 | 0.021 | 8.58E-06 | 0.451 | FALSE | 20 |
|  | rs11090513 | T | G | 0.798 | 0.013 | 0.175 | 0.015 | 5.00E-06 | 0.408 | FALSE | 21 |
|  | rs11230859 | A | G | -0.725 | 0.014 | 0.158 | 0.014 | 4.18E-06 | 0.287 | FALSE | 21 |
|  | rs12669698 | C | T | 1.127 | 0.003 | 0.255 | 0.020 | 9.73E-06 | 0.866 | FALSE | 20 |
|  | rs17145573 | A | G | 1.708 | 0.003 | 0.365 | 0.021 | 2.87E-06 | 0.878 | FALSE | 22 |
|  | rs389558 | T | C | -0.783 | 0.005 | 0.160 | 0.013 | 9.74E-07 | 0.686 | FALSE | 24 |
|  | rs491806 | C | A | -0.948 | 0.013 | 0.211 | 0.016 | 6.92E-06 | 0.402 | FALSE | 20 |
|  | rs529324 | A | G | -0.826 | -0.004 | 0.179 | 0.014 | 4.16E-06 | 0.756 | FALSE | 21 |
|  | rs7778345 | G | A | 0.730 | 0.012 | 0.163 | 0.013 | 7.05E-06 | 0.374 | FALSE | 20 |
| phylum.Actinobacteria(id.400) | rs10513202 | G | A | 1.427 | -0.010 | 0.321 | 0.017 | 8.58E-06 | 0.558 | FALSE | 20 |
|  | rs11090513 | T | G | 0.798 | -0.022 | 0.175 | 0.012 | 5.00E-06 | 0.081 | FALSE | 21 |
|  | rs11230859 | A | G | -0.725 | 0.002 | 0.158 | 0.011 | 4.18E-06 | 0.851 | FALSE | 21 |
|  | rs12669698 | C | T | 1.127 | 0.014 | 0.255 | 0.016 | 9.73E-06 | 0.380 | FALSE | 20 |
|  | rs17145573 | A | G | 1.708 | 0.029 | 0.365 | 0.017 | 2.87E-06 | 0.082 | FALSE | 22 |
|  | rs389558 | T | C | -0.783 | -0.010 | 0.160 | 0.011 | 9.74E-07 | 0.347 | FALSE | 24 |
|  | rs491806 | C | A | -0.948 | 0.015 | 0.211 | 0.013 | 6.92E-06 | 0.248 | FALSE | 20 |
|  | rs529324 | A | G | -0.826 | -0.008 | 0.179 | 0.011 | 4.16E-06 | 0.511 | FALSE | 21 |
|  | rs7778345 | G | A | 0.730 | 0.009 | 0.163 | 0.011 | 7.05E-06 | 0.406 | FALSE | 20 |
| phylum.Bacteroidetes(id.905) | rs10513202 | G | A | 1.427 | 0.000 | 0.321 | 0.017 | 8.58E-06 | 0.992 | FALSE | 20 |
|  | rs11090513 | T | G | 0.798 | -0.014 | 0.175 | 0.012 | 5.00E-06 | 0.273 | FALSE | 21 |
|  | rs11230859 | A | G | -0.725 | -0.004 | 0.158 | 0.011 | 4.18E-06 | 0.728 | FALSE | 21 |
|  | rs12669698 | C | T | 1.127 | -0.008 | 0.255 | 0.016 | 9.73E-06 | 0.632 | FALSE | 20 |
|  | rs17145573 | A | G | 1.708 | -0.028 | 0.365 | 0.017 | 2.87E-06 | 0.100 | FALSE | 22 |
|  | rs389558 | T | C | -0.783 | 0.005 | 0.160 | 0.011 | 9.74E-07 | 0.640 | FALSE | 24 |
|  | rs491806 | C | A | -0.948 | -0.016 | 0.211 | 0.013 | 6.92E-06 | 0.213 | FALSE | 20 |
|  | rs529324 | A | G | -0.826 | 0.004 | 0.179 | 0.011 | 4.16E-06 | 0.733 | FALSE | 21 |
|  | rs7778345 | G | A | 0.730 | -0.001 | 0.163 | 0.011 | 7.05E-06 | 0.951 | FALSE | 20 |
| phylum.Cyanobacteria(id.1500) | rs10513202 | G | A | 1.427 | 0.032 | 0.321 | 0.027 | 8.58E-06 | 0.236 | FALSE | 20 |
|  | rs11090513 | T | G | 0.798 | 0.004 | 0.175 | 0.020 | 5.00E-06 | 0.855 | FALSE | 21 |
|  | rs11230859 | A | G | -0.725 | 0.015 | 0.158 | 0.018 | 4.18E-06 | 0.394 | FALSE | 21 |
|  | rs12669698 | C | T | 1.127 | -0.004 | 0.255 | 0.027 | 9.73E-06 | 0.884 | FALSE | 20 |
|  | rs17145573 | A | G | 1.708 | 0.015 | 0.365 | 0.027 | 2.87E-06 | 0.589 | FALSE | 22 |
|  | rs389558 | T | C | -0.783 | 0.006 | 0.160 | 0.017 | 9.74E-07 | 0.712 | FALSE | 24 |
|  | rs491806 | C | A | -0.948 | 0.008 | 0.211 | 0.020 | 6.92E-06 | 0.712 | FALSE | 20 |
|  | rs529324 | A | G | -0.826 | -0.004 | 0.179 | 0.018 | 4.16E-06 | 0.822 | FALSE | 21 |
|  | rs7778345 | G | A | 0.730 | -0.004 | 0.163 | 0.017 | 7.05E-06 | 0.817 | FALSE | 20 |
| phylum.Euryarchaeota(id.55) | rs10513202 | G | A | 1.427 | 0.029 | 0.321 | 0.036 | 8.58E-06 | 0.419 | FALSE | 20 |
|  | rs11090513 | T | G | 0.798 | 0.003 | 0.175 | 0.028 | 5.00E-06 | 0.917 | FALSE | 21 |
|  | rs11230859 | A | G | -0.725 | 0.006 | 0.158 | 0.024 | 4.18E-06 | 0.810 | FALSE | 21 |
|  | rs12669698 | C | T | 1.127 | -0.019 | 0.255 | 0.036 | 9.73E-06 | 0.600 | FALSE | 20 |
|  | rs17145573 | A | G | 1.708 | -0.028 | 0.365 | 0.037 | 2.87E-06 | 0.456 | FALSE | 22 |
|  | rs389558 | T | C | -0.783 | 0.049 | 0.160 | 0.024 | 9.74E-07 | 0.043 | FALSE | 24 |
|  | rs491806 | C | A | -0.948 | 0.029 | 0.211 | 0.028 | 6.92E-06 | 0.299 | FALSE | 20 |
|  | rs529324 | A | G | -0.826 | 0.002 | 0.179 | 0.025 | 4.16E-06 | 0.938 | FALSE | 21 |
|  | rs7778345 | G | A | 0.730 | -0.035 | 0.163 | 0.023 | 7.05E-06 | 0.132 | FALSE | 20 |
| phylum.Firmicutes(id.1672) | rs10513202 | G | A | 1.427 | -0.027 | 0.321 | 0.017 | 8.58E-06 | 0.108 | FALSE | 20 |
|  | rs11090513 | T | G | 0.798 | 0.011 | 0.175 | 0.012 | 5.00E-06 | 0.354 | FALSE | 21 |
|  | rs11230859 | A | G | -0.725 | -0.012 | 0.158 | 0.011 | 4.18E-06 | 0.264 | FALSE | 21 |
|  | rs12669698 | C | T | 1.127 | -0.010 | 0.255 | 0.016 | 9.73E-06 | 0.519 | FALSE | 20 |
|  | rs17145573 | A | G | 1.708 | 0.012 | 0.365 | 0.017 | 2.87E-06 | 0.482 | FALSE | 22 |
|  | rs389558 | T | C | -0.783 | -0.001 | 0.160 | 0.011 | 9.74E-07 | 0.950 | FALSE | 24 |
|  | rs491806 | C | A | -0.948 | 0.017 | 0.211 | 0.013 | 6.92E-06 | 0.192 | FALSE | 20 |
|  | rs529324 | A | G | -0.826 | -0.001 | 0.179 | 0.011 | 4.16E-06 | 0.947 | FALSE | 21 |
|  | rs7778345 | G | A | 0.730 | 0.010 | 0.163 | 0.011 | 7.05E-06 | 0.342 | FALSE | 20 |
| phylum.Lentisphaerae(id.2238) | rs10513202 | G | A | 1.427 | 0.024 | 0.321 | 0.033 | 8.58E-06 | 0.454 | FALSE | 20 |
|  | rs11090513 | T | G | 0.798 | 0.026 | 0.175 | 0.025 | 5.00E-06 | 0.286 | FALSE | 21 |
|  | rs11230859 | A | G | -0.725 | -0.004 | 0.158 | 0.021 | 4.18E-06 | 0.848 | FALSE | 21 |
|  | rs12669698 | C | T | 1.127 | 0.014 | 0.255 | 0.032 | 9.73E-06 | 0.664 | FALSE | 20 |
|  | rs17145573 | A | G | 1.708 | -0.028 | 0.365 | 0.033 | 2.87E-06 | 0.395 | FALSE | 22 |
|  | rs389558 | T | C | -0.783 | -0.005 | 0.160 | 0.022 | 9.74E-07 | 0.807 | FALSE | 24 |
|  | rs491806 | C | A | -0.948 | -0.030 | 0.211 | 0.025 | 6.92E-06 | 0.223 | FALSE | 20 |
|  | rs529324 | A | G | -0.826 | 0.005 | 0.179 | 0.022 | 4.16E-06 | 0.806 | FALSE | 21 |
|  | rs7778345 | G | A | 0.730 | 0.018 | 0.163 | 0.021 | 7.05E-06 | 0.392 | FALSE | 20 |
| phylum.Proteobacteria(id.2375) | rs10513202 | G | A | 1.427 | 0.037 | 0.321 | 0.017 | 8.58E-06 | 0.028 | FALSE | 20 |
|  | rs11090513 | T | G | 0.798 | -0.001 | 0.175 | 0.012 | 5.00E-06 | 0.949 | FALSE | 21 |
|  | rs11230859 | A | G | -0.725 | 0.016 | 0.158 | 0.011 | 4.18E-06 | 0.142 | FALSE | 21 |
|  | rs12669698 | C | T | 1.127 | 0.026 | 0.255 | 0.016 | 9.73E-06 | 0.116 | FALSE | 20 |
|  | rs17145573 | A | G | 1.708 | -0.006 | 0.365 | 0.017 | 2.87E-06 | 0.740 | FALSE | 22 |
|  | rs389558 | T | C | -0.783 | -0.005 | 0.160 | 0.011 | 9.74E-07 | 0.649 | FALSE | 24 |
|  | rs491806 | C | A | -0.948 | 0.006 | 0.211 | 0.013 | 6.92E-06 | 0.639 | FALSE | 20 |
|  | rs529324 | A | G | -0.826 | 0.008 | 0.179 | 0.011 | 4.16E-06 | 0.470 | FALSE | 21 |
|  | rs7778345 | G | A | 0.730 | 0.002 | 0.163 | 0.011 | 7.05E-06 | 0.854 | FALSE | 20 |
| phylum.Tenericutes(id.3919) | rs10513202 | G | A | 1.427 | 0.014 | 0.321 | 0.021 | 8.58E-06 | 0.500 | FALSE | 20 |
|  | rs11090513 | T | G | 0.798 | 0.012 | 0.175 | 0.016 | 5.00E-06 | 0.449 | FALSE | 21 |
|  | rs11230859 | A | G | -0.725 | -0.002 | 0.158 | 0.014 | 4.18E-06 | 0.866 | FALSE | 21 |
|  | rs12669698 | C | T | 1.127 | 0.009 | 0.255 | 0.021 | 9.73E-06 | 0.666 | FALSE | 20 |
|  | rs17145573 | A | G | 1.708 | 0.005 | 0.365 | 0.021 | 2.87E-06 | 0.814 | FALSE | 22 |
|  | rs389558 | T | C | -0.783 | 0.003 | 0.160 | 0.014 | 9.74E-07 | 0.800 | FALSE | 24 |
|  | rs491806 | C | A | -0.948 | 0.007 | 0.211 | 0.016 | 6.92E-06 | 0.650 | FALSE | 20 |
|  | rs529324 | A | G | -0.826 | 0.007 | 0.179 | 0.014 | 4.16E-06 | 0.614 | FALSE | 21 |
|  | rs7778345 | G | A | 0.730 | 0.005 | 0.163 | 0.014 | 7.05E-06 | 0.708 | FALSE | 20 |
| phylum.Verrucomicrobia(id.3982) | rs10513202 | G | A | 1.427 | -0.007 | 0.321 | 0.020 | 8.58E-06 | 0.744 | FALSE | 20 |
|  | rs11090513 | T | G | 0.798 | 0.014 | 0.175 | 0.015 | 5.00E-06 | 0.333 | FALSE | 21 |
|  | rs11230859 | A | G | -0.725 | 0.009 | 0.158 | 0.013 | 4.18E-06 | 0.504 | FALSE | 21 |
|  | rs12669698 | C | T | 1.127 | 0.009 | 0.255 | 0.020 | 9.73E-06 | 0.643 | FALSE | 20 |
|  | rs17145573 | A | G | 1.708 | 0.007 | 0.365 | 0.021 | 2.87E-06 | 0.730 | FALSE | 22 |
|  | rs389558 | T | C | -0.783 | 0.013 | 0.160 | 0.013 | 9.74E-07 | 0.303 | FALSE | 24 |
|  | rs491806 | C | A | -0.948 | 0.009 | 0.211 | 0.016 | 6.92E-06 | 0.574 | FALSE | 20 |
|  | rs529324 | A | G | -0.826 | -0.006 | 0.179 | 0.014 | 4.16E-06 | 0.677 | FALSE | 21 |
|  | rs7778345 | G | A | 0.730 | 0.011 | 0.163 | 0.013 | 7.05E-06 | 0.407 | FALSE | 20 |
